# A Highly Selective Cobalt Catalyst for Primary Amine Synthesis from Carboxylic Acids, Esters, and Vegetable Oils

**DOI:** 10.1021/jacs.5c10097

**Published:** 2025-10-15

**Authors:** Fairoosa Poovan, Vishwas G. Chandrashekhar, Dilver Peña Fuentes, Thanh Huyen Vuong, Ralf Jackstell, Jabor Rabeah, Rajenahally V. Jagadeesh, Matthias Beller

**Affiliations:** † 28392 Leibniz-Institut für Katalyse e.V, Albert-Einstein-Straße 29a, Rostock 18059, Germany; ‡ Nanotechnology Centre, Centre for Energy and Environmental Technologies, VŠB-Technical University of Ostrava, Ostrava-Poruba 70800, Czech Republic; § State Key Laboratory of Low Carbon Catalysis and Carbon Dioxide Utilization, Lanzhou Institute of Chemical Physics (LICP), Chinese Academy of Sciences, Lanzhou 730000, China

## Abstract

The prevalence of carboxylic acids and esters in nature renders them diverse and conveniently accessible building blocks for chemical synthesis. In this Letter, we present a straightforward cobalt-based catalytic system for the selective synthesis of primary amines from carboxylic acid derivatives and ammonia. The current system has been demonstrated to provide an efficient synthesis route for various amine derivatives and exhibits excellent chemoselectivity despite the complex reaction network. The optimal catalyst offers broad functional group tolerance, with both (hetero)­aromatic and aliphatic substrates yielding the desired primary amines in good to excellent yields. The sophisticated Co-triphos^(p‑anisole)^ system has also been demonstrated to be efficacious in the direct synthesis of fatty amines from a variety of vegetable oils. In comparison with the industrially applied fatty amine process, the approach of one-pot fatty amine synthesis presented here represents a significant advancement in the field. This approach not only overcomes the inherent challenges but also aligns with sustainability goals by utilizing renewable feedstocks.

## Graphical abstract

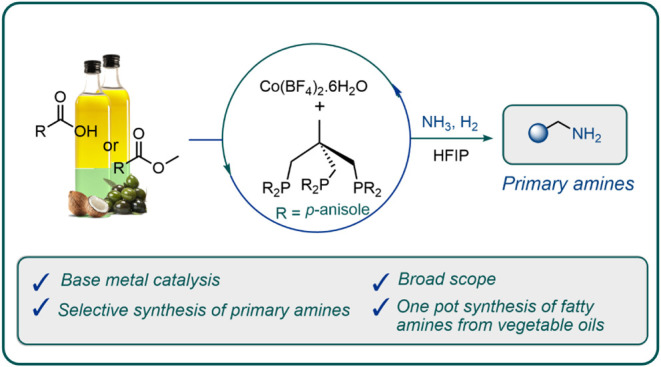

## Introduction

Primary amine compounds play a pivotal role among the various known amine compounds. They offer a wide range of further transformations and are utilized extensively as starting materials and intermediates in chemical synthesis. As indicated in Scheme 1a, they are a crucial structural motif in active pharmaceutical ingredients (APIs), ligand libraries, CO_2_ capturing agents, dyes, and polymers.^1–9^ Apart from traditional stoichiometric methods such as Gabriel synthesis, Curtius rearrangement, and the well-established reduction of nitrile or nitro groups, various catalytic protocols to synthesize primary amines have been developed.^10^

**Scheme 1:**
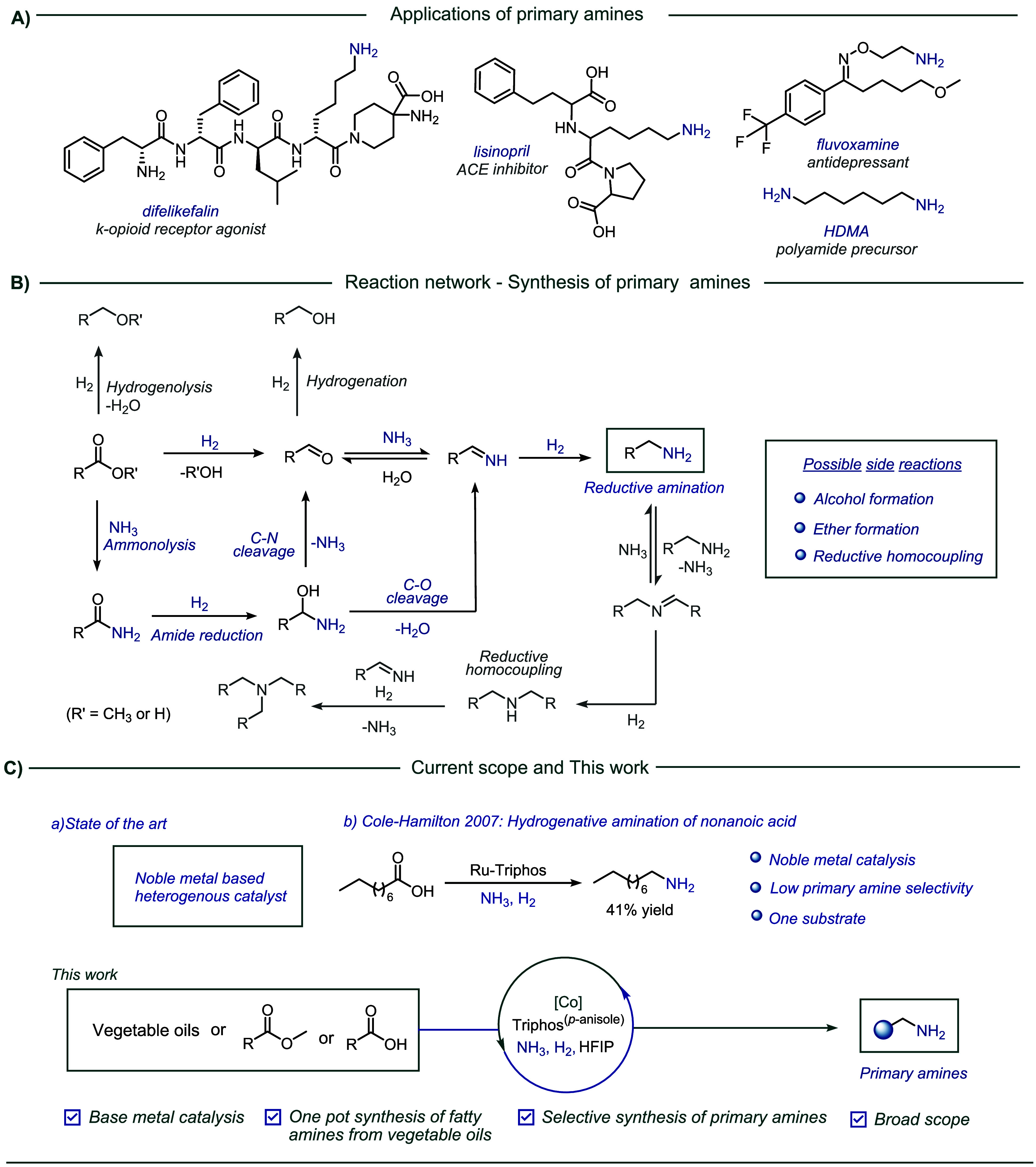
Overview: Cobalt Catalyzed Hydrogenative Amination. (A) Applications of Primary Amines; (B) Reaction Network; (C) Current Scope and this Work

Even though there are many protocols available, most C–N bond formations in the chemical industry are achieved using only a few well-known synthetic methods. In this respect, one of the most important chemical reactions in the industry for forming C–N bonds is the catalytic reduction of aldehydes or ketones using ammonia.^11^ However, the sensitivity, high reactivity, and limited availability of aldehydes often impede their application in advanced chemical synthesis. In general, the synthesis of amines from more stable carboxylic acids and esters presents a viable alternative to the established approach.^12–14^ Favorably, these substrates are more abundant in nature than olefins or aldehydes, which are derived from classic petrochemical feedstocks. Furthermore, the Haber–Bosch process, which utilizes green hydrogen, is anticipated to produce “green” ammonia in the forthcoming years and is expected to be a readily available feedstock.^15,16^ Consequently, the development of a synthetic toolbox for primary amines from carboxylic acid derivatives, hydrogen, and ammonia can be a significant and sustainable approach.

Despite the steady progress in transition metal catalysis over the past decade, with ammonia serving as a nitrogen source,^17–20^ the selective formation of primary amines under mild conditions remains a formidable challenge. The deactivation of the catalyst due to the formation of stable transition metal-ammonia complexes leads to less reactive metal-amine species,^21^ and chemoselectivity issues arising from the reactivity of the initially generated primary amines compared to ammonia—which leads to secondary or tertiary amines—are inherent obstacles in this field. Additionally, the control of chemoselectivity in reductive aminations employing carboxylic acids or esters is a challenging endeavor. The presence of multiple intermediates, along with the potential for side reactions stemming from the intrinsic reactivity of these intermediates, contributes to the complexity of the aforementioned reaction network (Scheme 1b). For instance, the partial reduction of esters or carboxylic acids to aldehydes is a slow process, and the generated aldehydes are more prone to reduction. Likewise, the presence of etherification and amide formation reactions complicates the selectivity to a considerable extent. Moreover, the primary amine selectivity can be decreased via reductive homocouplings, which result in the formation of secondary or tertiary amines.

In the year 1994, Barrault et al. reported a CuCrO_2_-based heterogeneous catalyst for the reductive amination of carboxylic acids and esters to primary amines. The reaction, using methyl dodecanoate and dodecanoic acid in the presence of ammonia and hydrogen, yielded up to 27% primary amines at 300 °C.^22^ Further catalysts developed to date for the formation of primary amines from acids or esters are mostly heterogeneous catalysts based on Ru, Au, Ag, or Pt, which require very high reaction temperatures (>200 °C), similar to the seminal work.^23,24^ Moreover, in the case of Ru-based catalytic systems, it was difficult to prevent hydrogenation of the aromatic ring when employing aromatic substrates.^23^ The first and only homogeneous catalyst developed for the conversion of carboxylic acids to primary amines was disclosed by Cole-Hamilton and coworkers.^25^ In this study, the ruthenium-1,1,1-tris­(diphenylphosphinomethyl)­ethane (tripodal-triphos) system was employed for the amination of nonanoic acid (studied as the only substrate) with ammonia. Unfortunately, the selectivity exhibited toward primary amines was found to be minimal.

In the past decade, a substantial number of studies in the area of homogeneous catalytic hydrogenation reactions have focused on the use of non-noble metal-based catalysts.^26^ In light of our long-standing interest in this area,^27–29^ a novel amination of polymers and (bio)­waste was achieved recently.^30^ Based on the realization of this waste-to-value concept, we present here a general hydrogenative amination of all kinds of carboxylic acids and esters, including one-pot fatty amine synthesis from vegetable oils and ammonia (Scheme 1c).

## Results and Discussion

### Catalyst Development and Optimization of the Reaction System

In order to develop a general synthesis methodology for primary amines from readily accessible esters and acids, the direct amination of methyl benzoate with ammonia in the presence of molecular hydrogen was utilized as a model reaction. Traditional reductive aminations of carbonyl compounds have predominantly been carried out by employing heterogeneous noble metal-based materials.^17,19,31^ However, over the past two decades, there has been a rapid emergence of molecularly defined 3d metal catalysts for a wide range of reductive transformations.^32–34^ An illustrative and elegant example is the work of the groups of de Bruin and Elsevier, which described a Co-triphos complex for the challenging hydrogenation of carboxylic acids .^35^ A critical factor for the application of this and related catalysts is the stability of the active metal complex,^28,29^ which can be achieved using multidentate ligands. Therefore, the present investigation was initiated using a model system with cationic Fe, Mn, Co, Ni, and Cu salts in the presence of various bi-, tri-, and tetradentate ligands, specifically dppe (1,2-bis­(diphenylphosphino)­ethane; **L1**) tetraphos (tris­[2-(diphenylphosphino)­ethyl]­phosphine; **L2**), and tripodal-triphos (tri-(1,1,1-tris­(diphenylphosphinomethyl)­ethane; **L3)** (Tables 1 and S1). To identify potential catalysts in a fast and reliable manner, *in situ*-generated complexes were applied. Notably, in the hydrogenative amination of methyl benzoate **1a** with ammonia, in addition to the desired benzylamine **2b**, other byproducts such as benzamide **3b** and benzyl alcohol **4b** can be anticipated as side products. As shown in Table S1, except for the triphos-ligated Co catalyst (Co-**L3**), the tested complexes did not promote the formation of **2b** but provided only **3b** (47–85%) and **4b** (8–24%). In contrast, applying Co-**L3** resulted in the detection of 25% of the desired product **2b,** accompanied by the formation of benzamide **3b** and benzyl alcohol **4b,** as provided in Table S1.

**Table 1:**
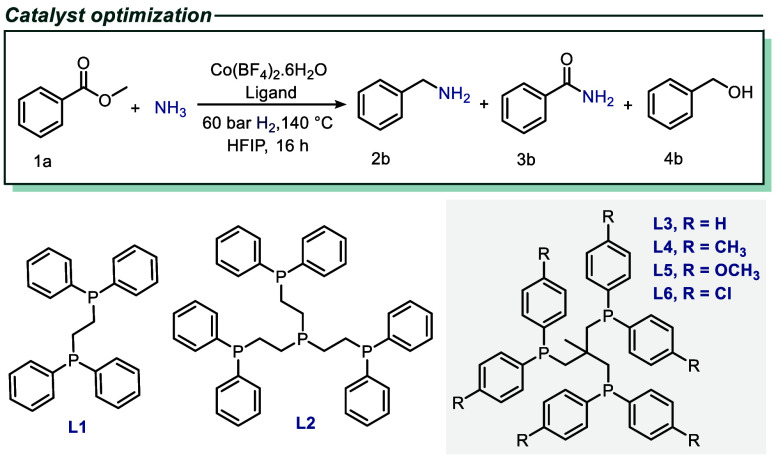
Co-Catalyzed Hydrogenative Amination Testing of Different Phosphine Ligands^a^

Entry	Co-Ligand	Conv. [%]	2b [%]	3b [%]	4b [%]
1	Co-L1	85	-	60	24
2	Co-L2	78	-	58	19
3	Co-L3	89	25	55	8
4	Co-L4	85	65	12	7
5	Co-L5	98	79	11	4
6	Co-L6	70	10	48	10
7	Co-L5 (1:1.1)	54	11	39	2
8	Co-(L5) (1:2.1)	>99	82	10	5
9	Co-(L5)^b^ (1:2.1)	>99	92	5	2

a Reaction conditions: 0.3 mmol methyl benzoate, 5 bar NH_3_, 8 mol % Co­(BF_4_)_2_·6H_2_O, 16 mol % ligand, 60 bar H_2_, 1 mL HFIP, 140 °C, 16 h.

b Similar conditions with 20 mol % Al­(O-*i*Pr)_3_. Conversion and yields were determined by GC using mesitylene as standard.

In order to enhance the selectivity of the process, three electronically distinct substituted triphos ligands (**L4**–**L6**) were prepared (Supporting Information 2) and the corresponding *in situ-*generated cobalt complexes were tested in the benchmark reaction (Tables 1 and S2). Here, triphos-substituted ligands with electron-donating groups (methyl, methoxy, **L4**–**L5**) displayed an increase in activity and selectivity for the formation of the desired product 2 (see Tables 1 and S2, entries 2–3 for further details). Conversely, the chloro-substituted ligand **L6** exhibited diminished activity and selectivity (see Tables 1 and S2). As demonstrated in Table 1 (entry 8), using a 1:2.1 Co-**L5** ratio, the yield of **2b** was increased to 82%. In light of the elementary reaction steps in the hydrogenative amination of methyl benzoate, we assumed that employing acidic or basic additives could enhance the overall transformation. Indeed, higher yields of the desired benzylamine **2b** were obtained in the presence of acidic cocatalysts (Table S3). Among these, inexpensive aluminum isopropoxide (Al­(O-iPr)_3_) demonstrated the most favorable outcome, with a 92% yield of the desired product (Table 1, entry 9).

Testing different solvents revealed that fluorinated ones, especially HFIP, showed the best activity and selectivity in the benchmark reaction (Table S4). Similar positive effects of fluorinated solvents have been observed in conventional reductive aminations,^32,33^ as well as other reactions in organic synthesis.^36–39^ At this point, it should be noted that the HFIP solvent can be conveniently recycled and reused, as demonstrated (Figure S2).^40^

Additionally, the thermal stability of the cobalt system was investigated. It is noteworthy that the majority of 3d-metal phosphine complexes exhibit heightened sensitivity in comparison to their noble congeners. Nevertheless, the catalyst demonstrated no deactivation in the model reaction at 180 °C and exhibited remarkable chemoselectivity (see Figure S3).

### Mechanistic Investigation

As illustrated in Scheme 1, the reaction of **1a** with ammonia and hydrogen can result in the formation of multiple products. Achieving chemoselectivity necessitates control of this intricate reaction network. From a mechanistic perspective, two general pathways exist for achieving the desired transformation: A. The catalytic hydrogenation of **1a** results in the formation of benzaldehyde, which reacts with ammonia to form an imine intermediate. Subsequent catalytic hydrogenation yields the primary amine **2b**. B. Alternatively, the primary amide **3b** could be formed first, followed by hydrogenation to give the desired amine **2b**. A series of control experiments were conducted to differentiate between the two reaction pathways, and the progression of the reaction and the intermediates was meticulously monitored (Scheme 2 and see Section S13 for more details).

**Scheme 2:**
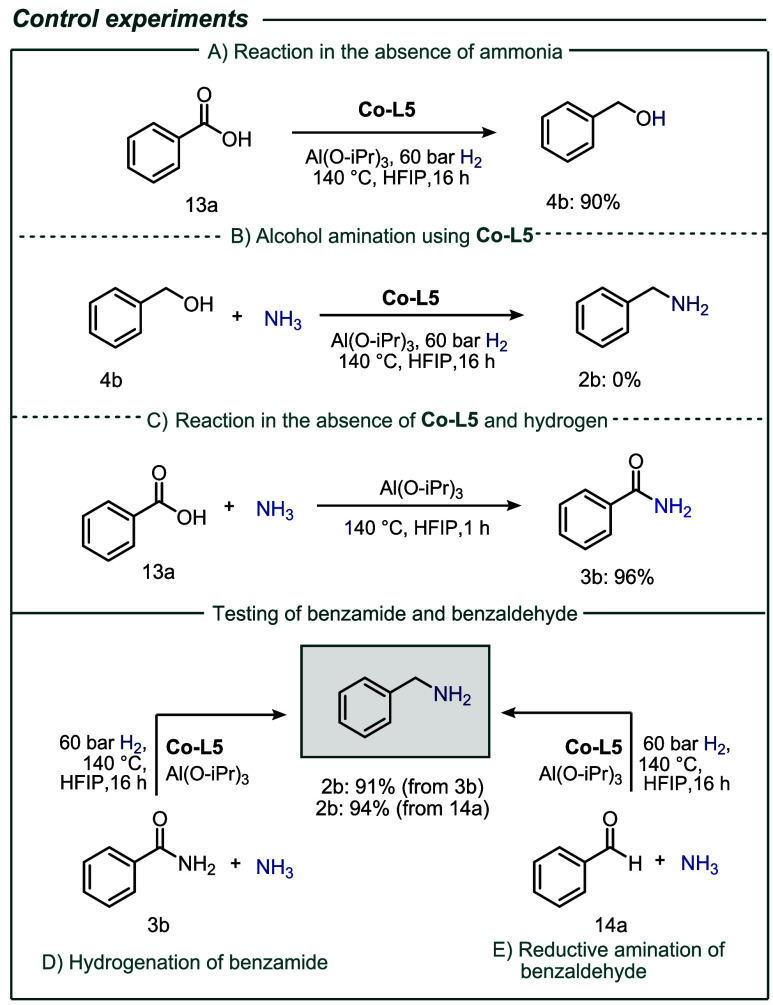
Mechanistic Studies: Control Experiments^a^

a Reaction conditions: 0.3 mmol substrate, 8 mol % Co­(BF_4_)_2_.6H_2_O, 2.1 equiv of **L5** to [Co], 5 bar NH_3_, 20 mol % Al­(O-iPr)_3_, 60 bar H_2_, 1 mL HFIP, 140 °C, 16 h. GC yields were determined using mesitylene as standard.

Under optimized reaction conditions excluding ammonia, **13a** yielded benzyl alcohol **4b** in 90% yield (Scheme 2a). It is evident that, under these conditions, the transformation of **4b** to **2b** is not possible (Scheme 2b), thereby precluding the possibility of an alcohol amination mechanism. Conversely, the hydrogenation of benzamide **3b** also occurred under these conditions (Scheme 2d). As anticipated, the reductive amination of benzaldehyde **13a** proceeded satisfactorily under these conditions, yielding 94% of the desired product, benzylamine **2b** (Scheme 2e). Notably, the Lewis acid-catalyzed formation of amide **3b** took place within 1 h under standard reaction conditions (Scheme 2c) and this latter finding suggests that the hydrogenative amination of esters and acids likely proceeds through a reaction pathway involving the corresponding amide as a major intermediate. To determine whether a reductive amination sequence is possible directly from carboxylic acids and esters under the employed conditions, the reaction system was investigated in more detail using further studies. In this respect, high-pressure, *in situ* NMR spectroscopy was performed at varying temperatures to gain insight into the catalytic species involved in the reaction (see Section S8 for details).^41–43^

Since the NMR measurements pointed to the presence of paramagnetic species, we further explored the reaction mechanism using EPR spectroscopy. The EPR analysis of the *in situ* Co catalyst (metal-to-ligand ratio 1:2) in HFIP at 95 K exhibits an EPR signal of rhombic symmetry at g_1_ = 2.250, g_2_ = 2.070, g_3_ = 1.978, and A_1_ = 174 MHz, A_2_ = 148 MHz, A_3_ = 148 MHz (estimated from EPR simulation, Figure 1) with a complex hyperfine splitting due to the coupling of the unpaired electron (S = 1/2) with ^59^Co nuclear spins (I = 7/2, 100%). The eight-line hyperfine structure of the second component is more clearly visible in the second derivative EPR spectra (Figure S6a).

**Figure 1:**
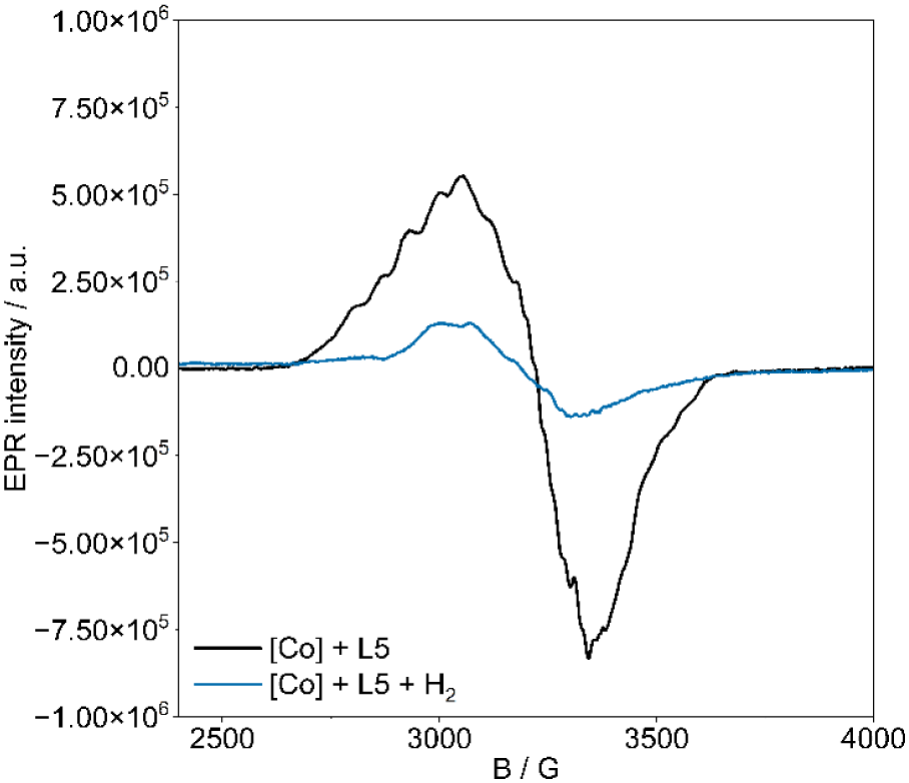
EPR spectra of **Co-L5** in the absence and presence of hydrogen in HFIP were measured at 95 K.

Subsequently, we investigated the *in situ*-prepared [Co] catalyst under a hydrogen atmosphere. The obtained EPR spectra showed a single broad signal at *g* = 2.1, which was lower than the initial one and lacked hyperfine structure (Figure 1). Therefore, we assume that the initial [Co] complex’s undergoes structural reorganization during the reaction. Moreover, the EPR analysis of the [Co] catalyst with BzOH in HFIP (Figure 2) showed an EPR signal of rhombic symmetry with minor differences in parameters at *g*_1_ = 2.205, *g*_2_ = 2.006, *g*_3_ = 1.983, and *A*_1_ = 174 MHz, *A*_2_ = 147 MHz, *A*_3_ = 162 MHz, along with a well-resolved eight-line hyperfine structure (Figure S6b). Additional splitting of the hyperfine interaction with the three ^31^P nucleus (I = 1/2) was observed in the second derivative EPR spectra (Figure S6b). These Hamiltonian parameters are similar to those of Co phosphine complexes with square-planar or square-pyramidal geometries of low-spin Co­(II) ions.^35,41,44^

**Figure 2:**
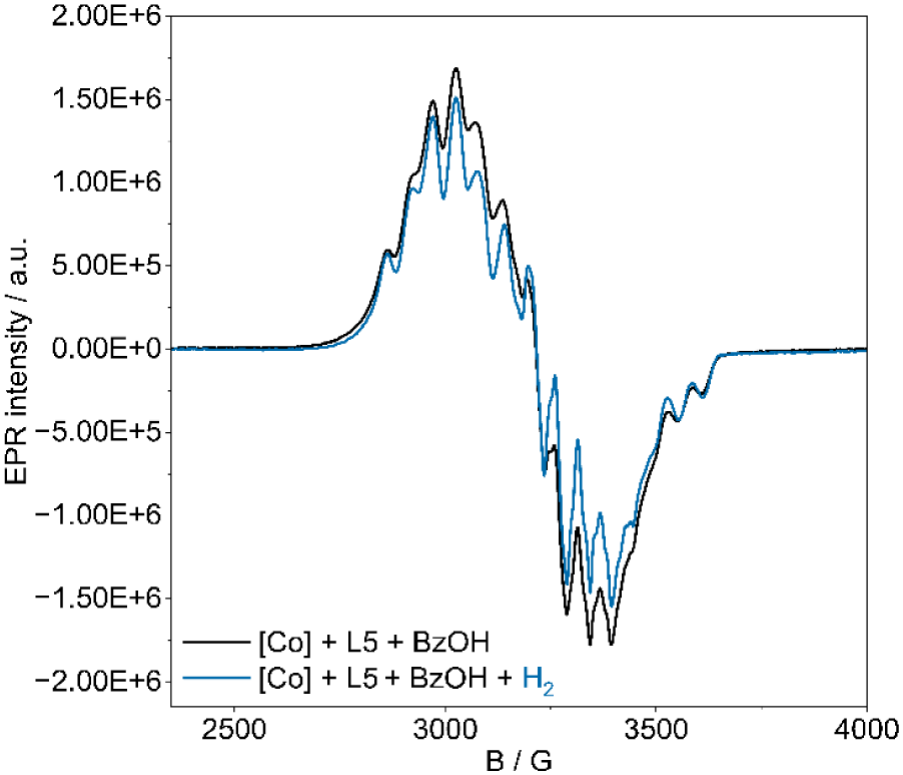
EPR spectra of **Co-L5** and benzoic acid in the absence and presence of hydrogen in HFIP were also measured at 95 K.

To learn more about the active cobalt species, we performed EPR measurements on the reaction mixture after catalysis. The resulting reaction mixture showed a Co­(II) signal similar to that shown in Figure 1 but with a slightly lower intensity. Furthermore, these results suggest the presence of a cobalt–substrate complex, [Co-triphos­(p-anisole)­benzoate]^+^, which was confirmed by ESI-MS (Figure S7). Additionally, no radical intermediate signal was detected during further control measurements, which excludes a radical mechanism.

A mercury poisoning test was conducted with the **Co-L5** catalyst, and no considerable inhibition of catalysis was observed, confirming that the reaction proceeds via homogeneous catalysis and that the formation of Co nanoparticles is not likely to occur (see Figure S8). Based on all of the experimental data obtained and the literature precedents, we propose the following mechanism. First, the formation of Co­(triphos­(p-anisole))­(alkanoate) species takes place, followed by heterolytic hydrogen splitting, leading to a Co–H complex. After hydride addition to the carbonyl carbon, a subsequent second heterolytic hydrogen splitting step takes place. After one equivalent of water is expelled, a [Co­(triphos^(p‑anisole)^)­(H)­(aldehyde)]^+^ species is formed. Next, the primary imine formed from the aldehyde in the presence of ammonia generates the Co-imine complex. Finally, after β-hydride addition and H_2_ coordination, followed by hydrogenolysis, the primary amine is released, and the catalytically active species is regenerated (Scheme 3).

**Scheme 3:**
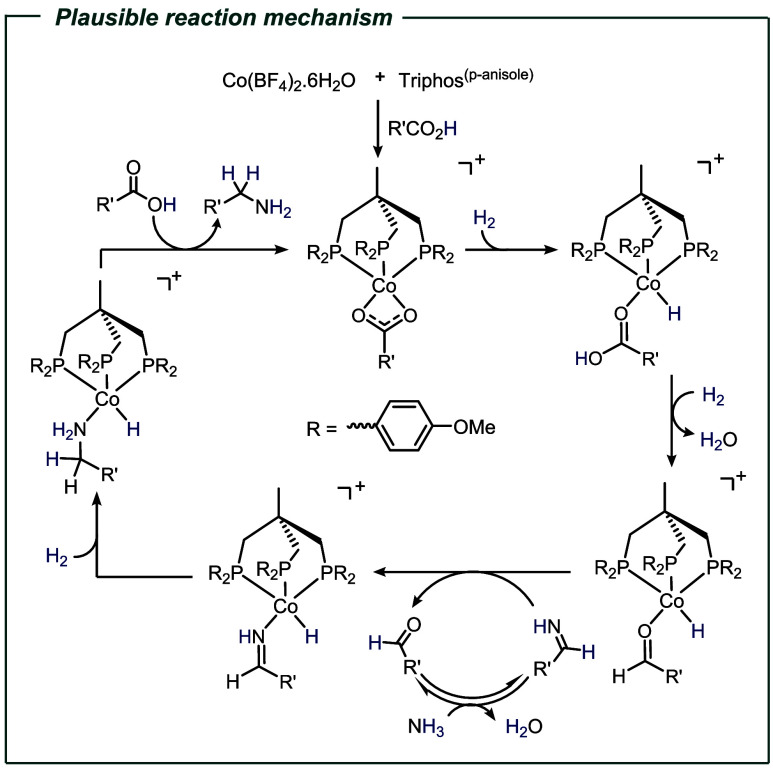
Mechanistic Proposal for the “Non-Amide” Involved Reaction Pathway

### Synthesis of Primary Amines from Esters and Carboxylic Acids: Scope and Applications

In order to illustrate the synthetic utility of the presented catalyst system, an investigation was conducted into the applicability of the **Co-L5** system for the hydrogenative amination of diverse esters and carboxylic acids (Scheme 4). As demonstrated in Scheme 4, a range of carboxylic acids, including aromatic, heterocyclic, and aliphatic derivatives, along with esters, were subjected to amination reactions with ammonia. This process generated primary amines in good to excellent yields. The amination of methyl esters and the corresponding carboxylic acids showcased similar activity and selectivity during the process. In addition to conventional substrates, a number of functionalized substrates gave the corresponding primary amines in high yields. Halogenated benzylamines were obtained in yields of up to 90% (**12b**–**16b** and substrates containing methoxy-, benzyloxy-, trifluoromethoxy-, hydroxy-, and thioether groups yielded the corresponding benzylamines in up to 89% yield (**17b**–**26b**).

**Scheme 4:**
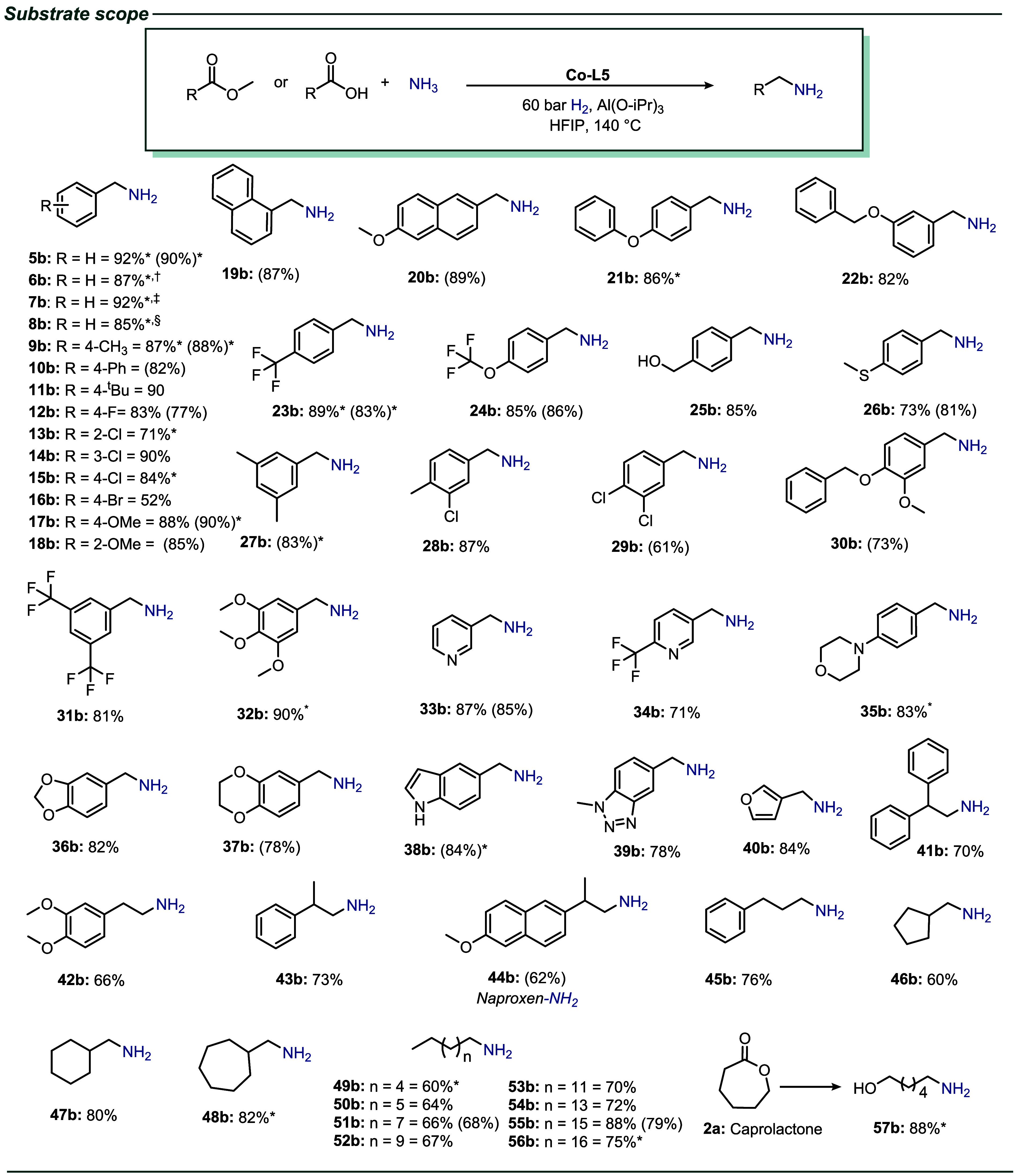
Substrate Scope – **Co-L5** Catalyzed Hydrogenative Amination of Various Carboxylic Acids and Esters[Fn sch4-fn2]

a Reaction conditions: 0.5 mmol ester or acid, 8 mol% Co­(BF_4_)_2_·6H_2_O, 2.1 equiv. of L5 to [Co], 5 bar NH_3_, 20 mol% Al­(O-iPr)_3_, 60 bar H_2_, 1 mL HFIP, 140 °C, 16–20 h, isolated yields. *GC yields using mesitylene as the standard. †From ethyl benzoate, ‡from butyl benzoate, §from tert-butyl benzoate. Note: Yields given in parentheses correspond to primary amines obtained from acids.

Additionally, the amination of di- and trisubstituted methyl benzoates and the corresponding acids was efficiently executed (**27b**–**32b**). Furthermore, a series of amines comprising significant heterocycles, including pyridine, furan, morpholine, benzodioxole, benzodioxane, indole, and benzotriazole, was synthesized from the corresponding heterocyclic esters and acids (**33b**–**40b**). In general, the amination of aryl-alkyl and aliphatic substrates is more challenging due to reduced selectivity and self-coupling reactions, which typically result in the formation of secondary amines. Nonetheless, these substrates were also selectively synthesized with up to 82% yield, representing a substantial advancement over the previously reported cobalt-catalyzed reductive amination of aldehydes (**41b**–**56b**).^31^ Apart from methyl esters, different kinds of aliphatic esters, e.g., ethyl, butyl, and tertiary butyl benzoates, provided the respective primary amines (**6b**–**8b**). In addition, ε-caprolactone was used as an example of lactones, which can be converted to the corresponding amino alcohol (**57b**). Amino alcohols are important structural motifs with a wide range of diverse pharmaceutical applications. As per FDA reports, the presence of amino alcohol residues has been detected in over 30% of small-molecule drugs, underscoring their importance in contemporary drug design.^45–47^ The developed protocol demonstrated broad applicability, providing products with yields ranging from good to excellent. Nevertheless, a few limitations were observed, e.g., in the case of halogenated substrates (**13b and 16b**), reductive dehalogenation of bromo and chloro substituents could be observed in varying amounts.

### Conversion of Renewable Vegetable Oils to Primary Fatty Amines

Among the array of renewable feedstocks, vegetable oils have emerged as the predominant biomass-based feedstock in terms of scale. Indeed, the annual production of vegetable oils exceeds 200 million metric tons and has a multitude of applications,^48,49^ including potential use in biorefinery concepts.^50,51^ These applications are relevant to the production of numerous everyday products, including soaps, candles, cosmetics, and food products.^52^

Notably, naturally occurring and commercially available vegetable oils constitute mixtures of different triglycerides and fatty acids. The valorization of these compounds typically necessitates a series of functionalization and purification steps. For instance, the predominant current production of primary fatty amines—a highly significant class of chemicals due to their wide applications in personal care products, household products, and allied industries,^53–56^ proceeds via the so-called “nitrile route” from vegetable oils. The process consists of three distinct steps (Scheme 5a): The first step in the synthesis of fatty amines involves the hydrogenation of the fatty acid alkyl chain, followed by the hydrolysis of vegetable oil to release fatty acids. The second step includes the amination-dehydration of fatty acids at high temperatures (>250 °C) in the presence of metal oxide catalysts, such as alumina or zinc oxide. The third step is a final catalytic hydrogenation of fatty nitriles, which results in the desired amine products.^57–60^ Despite being well-established, this method of fatty amine synthesis is characterized by several drawbacks, such as harsh reaction conditions and multiple steps. In addition, the formation of undesired mixtures of primary, secondary, and tertiary amines complicates the process, as their separation is difficult.

**Scheme 5:**
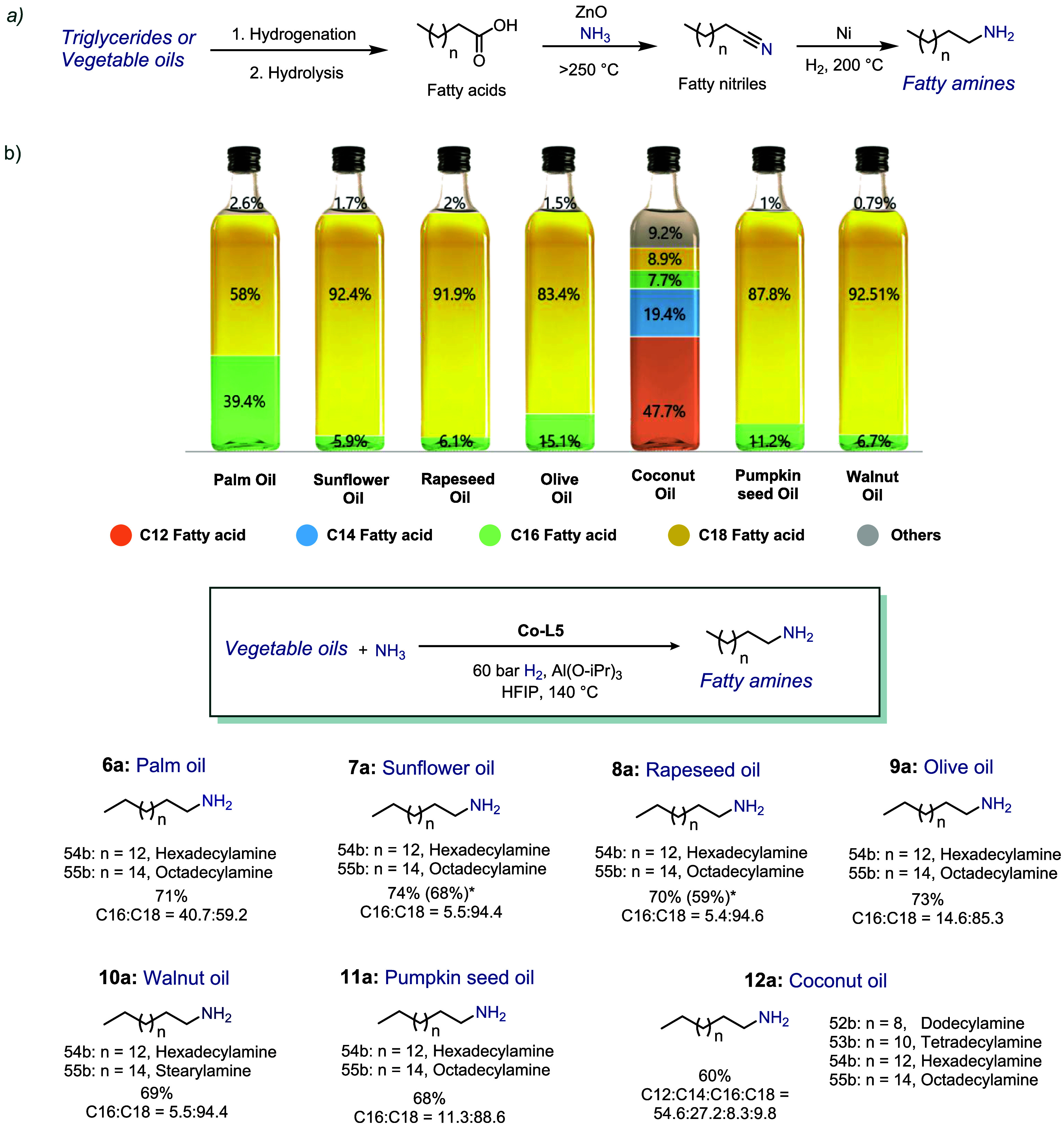
Straightforward Synthesis of Fatty Amines from Vegetable Oils[Fn sch5-fn3]

a Reaction conditions: 0.1 mmol vegetable oil, 6 mol % Co­(BF_4_)_2_.6H_2_O for each ester unit, 2.1 equiv of **L5** to [Co], 20 mol % Al­(O-iPr)_3_ for each ester unit, 5 bar NH_3_, 60 bar H_2_, 2 mL HFIP, 140 °C, 24 h. Yields correspond to the total yield of all fatty amines obtained after the reaction, GC yields were determined using mesitylene as the standard. *Isolated yield.

A more straightforward approach that can selectively transform vegetable oils into primary fatty amines would significantly minimize the aforementioned challenges. The first report of hydrogenative amination of triglycerides was presented in a patent application by Henkel, using ZnO-Al_2_O_3_ catalyst. The seminal work demonstrated excellent selectivity toward amines; however, significantly harsher reaction conditions (250 bar H_2_ and 310 °C) were required.^61^ In the year 2016, a homogeneous catalytic method for the direct synthesis of *N*-alkylated amines from triglycerides under hydrogen was reported by our group, utilizing the [Ru­(acac)_3_]/Triphos/HNTf_2_ catalytic system (acac—acetylacetonate; HNTf_2_ - triflimide). Although this study represented a significant step forward in this area, the catalytic system was not tested for primary amine synthesis.^13^ In 2019, Shimizu and coworkers demonstrated a platinum based heterogeneous catalyst for the direct conversion of triglycerides to fatty amines. The reactions performed using different triglycerides gave up to 51% dodecylamine and 32% didodecylamine.^62^ Although the system was tested with 6 different triglycerides, the long reaction time (96 h) and the high temperature (220 °C) constrained the applicability of this protocol.

During the preparation of this manuscript, Mizugaki and coworkers presented an interesting direct reductive amination of triglycerides to fatty amines using a titanium oxide-supported platinum-molybdenum (Pt–Mo/TiO_2_) catalyst. Reactions were performed under milder conditions (10 bar of H_2_, 180 °C, and 16–48 h) compared to previous reports. The reusability of the catalyst, as well as testing a wide variety of triglycerides for the synthesis of amines, are advantages of the developed system. However, employing ammonia for reductive amination yielded 74% of secondary amine instead of the primary fatty amine.^63^

Using our **Co-L5** system, different fatty amines can be conveniently obtained from pure triglycerides (Figure S9) as well as natural vegetable oils (Scheme 5b). For the initial screening, 3 diverse commercially available triglycerides were chosen (C7, C16, and C18). Under standard reaction conditions, the amination of commercially available triglyceride **5a** gave the corresponding primary fatty amines in lower yields, along with the generation of the corresponding fatty amide (Figure S9). Prolonging the reaction time to 24 h and using 6 mol % catalyst loading for each ester unit gave 86% yield of stearyl amine.

After triglyceride optimization, 7 distinct vegetable oils were chosen for hydrogenative amination. For ease of analytics, a fatty acid assay was generated after hydrolysis for each vegetable oil before the reaction (Scheme 5b and Table S6). For the majority of vegetable oils, characteristic C16 and C18 fatty acids were prominent, whereas coconut oil had a high composition of C12 fatty acid, and the presence of C14 fatty acid was also observed (Table S6). Palm oil **6a**, sunflower oil **7a**, rapeseed oil **8a**, olive oil **9a**, walnut oil **10a**, pumpkin seed oil **11a**, and coconut oil **12a** underwent reductive amination with ammonia and molecular hydrogen to give primary fatty amines C12–C18 in up to 74% yields (Scheme 5b, products **52b**–**55b**). In agreement with the results using triglycerides, the developed catalyst demonstrated a high degree of selectivity for the formation of primary amines, with no evidence of the homocoupling of primary amines. It is also noteworthy that hydrogenation of the double bond was observed in the case of all vegetable oils utilized in this study, which, in turn, simplifies downstream processing in fatty amine synthesis.

## Conclusion

In summary, a general catalytic route toward the synthesis of primary amines from carboxylic acid derivatives and ammonia in the presence of molecular hydrogen is presented. This approach is characterized by its selectivity as well as efficiency, making it a promising methodology for the preparation of a diverse class of valuable compounds. The success of this straightforward hydrogenative amination process is contingent upon the implementation of two critical components: the use of the stable and robust cobalt-based catalyst **Co-L5** and the employment of HFIP as a solvent. Among various triphos derivatives synthesized, the triphos^(p‑anisole)^ ligand demonstrated superior activity and selectivity. A broad variety of carboxylic acids and esters provided both (hetero)­aromatic and aliphatic primary amines in high to excellent yields. The advantages of this straightforward methodology are exemplified by the utilization of renewable feedstocks and vegetable oils for the one-pot synthesis of fatty amines. Starting from a series of seven distinct commercial-grade vegetable oils, each exhibiting a unique fatty acid composition, fatty amines were obtained in yields of up to 74% with excellent selectivity toward primary amines. Compared to the existing industrial synthesis of fatty amines, the current strategy of one-pot fatty amine synthesis presented herein represents a considerable step forward in this field.

## Supplementary Material



## Notes

Experimental procedures, mechanistic studies, and characterization of compounds (PDF)
