# Supplementary material for: A Highly Selective Cobalt Catalyst for Primary Amine Synthesis from Carboxylic Acids, Esters, and Vegetable Oils
Source: J Am Chem Soc. 2025 Oct 15;148(6):5966–75. doi: 10.1021/jacs.5c10097 (PMC12921917; doi:10.1021/jacs.5c10097)
Supplement: Supplementary file 1 [file ja5c10097_si_001.pdf]

Supporting Information

A highly selective cobalt catalyst for primary amine synthesis from carboxylic acids, esters and vegetable oils

Fairoosa Poovan[1], Vishwas G. Chandrashekhar[1], Dilver Peña Fuentes[1], Thanh Huyen Vuong[1], Ralf Jackstell[1], Jabor Rabeah[1,3], Rajenahally V. Jagadeesh[1,2]* and Matthias Beller[1]*

[1]Leibniz-Institut für Katalyse e.V., Albert-Einstein-Str. 29a, 18059 Rostock, Germany
[2]Nanotechnology Centre, Centre for Energy and Environmental Technologies, VŠB-Technical University of Ostrava, 70800, Ostrava-Poruba, Czech Republic

[3]State Key Laboratory of Low Carbon Catalysis and Carbon Dioxide Utilization, Lanzhou Institute of Chemical Physics (LICP), Chinese
Academy of Sciences, Lanzhou 730000, China

*Corresponding authors. E-mails: matthias.beller@catalysis.de ; jagadeesh.rajenahally@catalysis.de

Table of Contents

1. General information: Materials and methods 3
2. Preparation of triphos-ligands 5
3. General procedure for the hydrogenative amination of esters or carboxylic acids with ammonia......10
4. Hydrogenative amination of triglycerides and vegetable oils using ammonia 11
5. Reaction optimization 13
6. Recycling of HFIP solvent 16
7. Catalyst stability experiment 17
8. High pressure NMR experiments 17
9. EPR measurements 20
10. ESI-MS analysis 22
11. Catalyst poisoning experiment 22
12. Extended substrate scope for hydrogenative amination of triglycerides 23
13. Extended control experiments 23
14. Composition of vegetable oils 24
15. Analysis report of vegetable oils 26
16. NMR data 33
17. NMR spectra 44
18. References 79

1. General information: Materials and methods

Unless specified, all esters, carboxylic acids and amines were obtained commercially from various chemical companies and their purity has been checked before their use for the reactions. Cobalt(II) tetrafluoroborate hexahydrate (cat. no. PO7935-100002, 5G; 99.0%,) and hexaflouroisoproponol (HFIP; Cat. no. 003409, 1 Kg; 99.0%,) were purchased from Fluorochem Ltd. Tripodal triphos ligand (bis-(2-diphenylphosphinoethyl)-phenylphosphine; Lot #A0408544; Cat. no. 316820050, 5G; 97%) was obtained from Across Organics. Other commercial ligands were obtained from different chemical companies. $Al(O\text{-}iPr)_3$ (Lot. VWAFN-RR; Cat. no. A0246, 100 G; 98.0%(T)) was obtained from TCI chemicals. N-Methylamine (12 M in ethanol; Lot #BCCB0998; Cat. no. 534102-250ML), and N,N-dimethylamine (12 M in ethanol; Lot #BCBL6317V; Cat. no. 38950-250ML-F) was purchased from Sigma Aldrich and used as received. Vegetable oils were purchased from nearby stores (www.kaufland.com) and used as received. Analysis of purchased oils has been performed by Institute of Food Quality of LUFA Nord-West, Oldenburg, Germany.

For the ligand synthesis, 4-bromoanisole (Lot # STBJ3720; Cat. no. B56501, 500 G; ≥99.0%), diethyl phosphate (Lot # BCBW7242; Cat. no. D99234, 250 G; ≥99.0%), diisobutylaluminum hydride solution (1.0 M in hexanes, Lot # MFCD00008928; Cat. no. 190306, 800 mL G; ≥99.0%) and 1,1,1-tris(chloromethyl)ethane (Lot #0000139240; Cat. no. 380776, 25 mL; ≥99.0%) were purchased from Sigma Aldrich. 4-Bromotoluene (Lot #FCB109030; Cat. no. 001435, 25G; 98%) and 1-bromo-4-chlorobenzene (Lot #FCC3149076; Cat. no. 094835, 25G; 99%) were obtained from Fluorochem. KO^tBu (Lot #TAXCL-JW; Cat. no. P1008, 100 G; 99%) was received from TCI chemicals.

Ligands and Co-complexes were prepared using standard Schlenk and glovebox techniques. All catalytic reactions were carried out in 300 mL autoclave (PARR Instrument Company). In order to avoid unspecific reactions, catalytic reactions were carried out either in glass vials, which were placed inside the autoclave, or glass/Teflon vessel fitted autoclaves.

All organic solvents were distilled and stored in glass Schlenk flasks over molecular sieves (3 Å and 4 Å). Solvents were degassed by argon before using for the reaction.

^1H, ^{13}C NMR data were recorded on a Bruker Avance III HD 300 and 400 spectrometers using DMSO-d_6, $CDCl_3$, D_2O solvents.

GC conversion and yields were determined by GC-FID, HP6890 with FID detector, column HP530 m x 250 mm x 0.25 μm. GC-MS analysis of products was performed by Agilent, HP-5 MS, capillary column, 30 m × 0.25 mm × 0.25 μm.

High pressure NMR spectra were recorded on a Bruker Avance 400 spectrometer. HP-NMR-spectroscopic experiments were conducted using an NMR-cell setup from Daedalus (Aston, USA) with a 5 mm OD (3.4 mm ID) sapphire tube and a cell made of titanium.

https://daedalusinnovations.com/high-pressure-nmr/

NMR measurements at 0.1 MPa were carried out using a normal thin wall J. Young NMR tube. If ^{31}P served as the sensitive nucleus, proton decoupling was performed during the pulse sequence but not during the recycle delay ("inverse gated" to prevent dielectric heating); if ^{1}H served as sensitive nucleus, phosphorus was decoupled in a similar fashion.

The assessment of the NMR data was accomplished with TopSpin (Bruker) and Mestrenova (Mestrelab). The chemical shifts are reported relative to solvent signal (HFIP-d2: ^{1}H, δ(-CH-) = 4.39 ppm).

HRMS data were recorded on (1) LCMS-HR: Xevo G2-XS Tof /ESI-ES (Waters) and (2) EI-HRMS: Mass Spectrometer MAT 95XP (Thermo Electron), 70 eV.

EPR measurements were performed on a Bruker EMX CW-micro X-band spectrometer with a microwave power ≈ 6.9 mW, a modulation frequency of 100 kHz and modulation amplitude of 1 G. The EPR spectrometer is equipped with a variable temperature control unit including a liquid N_2 cryostat and a temperature controller for recording the EPR spectra at low temperature down to 95K. g values were calculated using the equation $h\nu = g\beta B_0$ with β, B_0 and ν being the Bohr magneton, resonance field and frequency, respectively. The DPPH standard (g = 2.0036 ± 0.0004) used as reference substance for calibration of the g values.

2. Preparation of triphos-ligands

Ligands **L4-L6** are prepared by modifying previously reported procedures[1-4].

General scheme

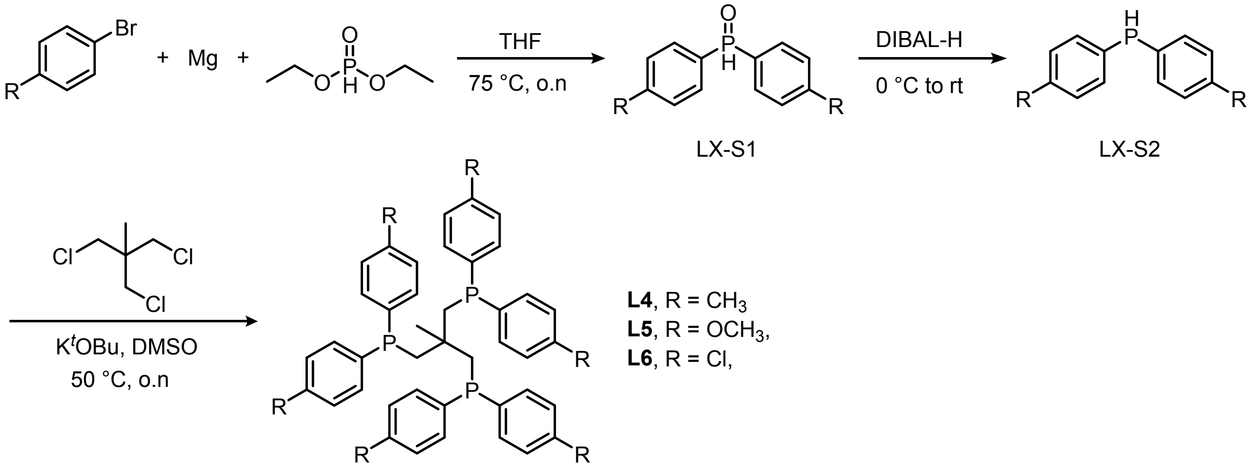

L4, R = CH_3
L5, R = OCH_3,
L6, R = Cl,

Synthesis of L4

Step-1. An oven dried 500 mL three-neck round bottom flask equipped with reflux condenser and addition funnel was charged with magnesium turnings (4.26 g, 175.4 mmol), I_2 (1 crystal) and THF (200 mL) under argon flow. To this pale brown mixture, 20 mL solution of 1-bromo-4-methylbenzene (25 g, 146.16 mmol, dissolved in 50 mL THF) was added dropwise via the addition funnel and the Grignard reagent formation was initiated by heating with a heat gun. Then the remaining 30 mL of 1-bromo-4-methylbenzene solution was added dropwise. After completing the addition, the reaction mixture was stirred at 75 °C for 3 h. Next, the reaction mixture was cooled to 0 °C and diethyl phosphite (7.06 g, 51.16 mmol) was added dropwise to the reaction mixture via addition funnel over 15 minutes. Then the reaction mixture was stirred overnight at ambient temperature. Afterwards, the reaction was quenched by adding 0.5 N HCl (200 mL) at 0 °C, and the mixture was extracted with EtOAc (100 mL x 2). The combined organic layers were washed with brine (200 mL) and dried over Na_2SO_4 and filtered. The solvent from the filtrate was removed in rotary evaporator, the resulting crude product was recrystallized from the mixture of 10% EtOAc in n-heptane to obtain the phosphine oxide as white solid (**L4-S1**) (7.9 g, 67% yield). ^{1}H NMR (300 MHz, $CDCl_3$) δ 8.01 (d, J = 478.1 Hz, 1H), 7.61 – 7.51 (m, 4H), 7.30 – 7.26 (m, 4H), 2.39 (s, 6H). ^{31}P(quant) NMR (122 MHz, $CDCl_3$) δ 21.60. EI-HRMS (m/z): Calculated for [$C_{14}H_{14}OP$]: 229.07768; found: 229.07780.

Step-2: To an oven dried 500 mL three-neck round bottom flask, which is equipped with an addition funnel and maintained under argon atmosphere, DIBAL-H (108 mL, 3.5equiv, 1.0 M in hexane) was transferred through cannula and the round bottom flask was cooled to 0 °C. Next the phosphine oxide (**L4-S1**) (7.9 g, 30.84 mmol) was added portion wise (Evolution of hydrogen was observed during the addition of phosphine oxide) followed by the addition of freshly degassed THF (70 mL) under argon atmosphere. After the addition of phosphine oxide and THF, the reaction mixture was stirred at ambient temperature for 3 h. Then the reaction mixture was cooled to 0 °C and freshly degassed Et_2O (50 mL) was added via addition funnel followed by the addition of 2 N aq. NaOH (120 mL) (freshly degassed) via additional funnel over 20 minutes (vigorous gas evolution). Subsequently, 40 mL of saturated aq. NaCl (freshly degassed) was added. The whole mixture was stirred for additional 20 minutes at ambient temperature. The organic phase was then transferred via cannula to a 500 mL dried round bottom flask which was pre-charged with $MgSO_4$ under argon. Then the aqueous phase was again extracted with freshly degassed Et_2O (70 mL) and the organic phase was transferred to the above mentioned round bottom flask and stirred for 5 minutes. The organic mixture was filtered through celite, and the solvent was removed under vacuum. The resulting semi-solid of phosphine hydride was confirmed by ^{31}P NMR and carried next step without further purification (**L4-S2**) (7 g, 95% yield).^{1}H NMR (300 MHz, CD_3CN) δ 7.40 – 7.34 (m, 4H), 7.16 – 7.12 (m, 4H), 5.14 (d, J = 218.5 Hz, 1H), 2.29 (s, 6H). ^{31}P NMR (122 MHz, CD_3CN) δ -42.61.

Step-3: An oven dried 500 mL two-neck round bottom flask was charged with di-p-tolylphosphane (**L4-S2**) (7 g, 32.67 mmol), KO^{t}Bu (3.66 g, 32.67 mmol) and freshly degassed DMSO (70 mL) under argon. The resulting dark red mixture was stirred for 30 minutes at room temperature. To this mixture, 1,1,1-tris(chloromethyl)ethane (1.43 g, 8.16 mmol) was added dropwise via syringe, and the reaction mixture was stirred at 60 °C for overnight. During this time, a discoloration of the dark red solution was observed. Then the reaction mixture was cooled to 0 °C, degassed water (80 mL, observed precipitation upon addition) was added dropwise and stirred for 30 minutes. The obtained solid was filtered under argon flow. The resulting compound was washed with H_2O (2 x 30 mL), MeOH (2 x 30 mL), followed by Et_2O (30 mL) and dried under vacuum to obtain white solid (**L4**) (5.2 g, 90% yield). ^{1}H NMR (400 MHz, $CDCl_3$) δ 7.30 – 7.24 (m, 12H), 7.11 – 7.06 (m, 12H), 2.42 (d, J = 3.1 Hz, 6H), 2.33 (s, 18H), 0.96 (s, 3H). ^{31}P NMR (162 MHz, $CDCl_3$) δ -27.99. EI-HRMS (m/z): Calculated for [$C_{47}H_{51}P_3$]: 708.31981; found: 708.31823.

Synthesis of L5

Step-1: An oven dried 1 L three-neck round bottom flask equipped with reflux condenser as well as addition funnel was charged with magnesium turnings (7.79 g, 320.79 mmol) and I_2 (2 crystal) and THF (400 mL) under argon flow. To this pale brown solution, 15 mL solution of 1-bromo-4-methoxybenzene (50 g, 267.32 mmol, dissolved in 50 mL THF) was added dropwise via the addition funnel and the Grignard reagent formation was initiated by heating with a heat gun. Subsequently, remaining 35 mL of 1-bromo-4-methoxybenzene solution was added dropwise and the reaction mixture was stirred at 75 °C for 3 hours. After 3 hours, the reaction mixture was cooled to 0 °C, diethyl phosphite (12.92 g, 93.56 mmol) was added dropwise to the reaction mixture via addition funnel over 20 minutes and stirred for overnight at ambient temperature. The reaction was quenched with 1N HCl (300 mL) at 0 °C and extracted with EtOAc (100 mL x 2). The combined organic layers were washed with brine (250 mL), dried over Na_2SO_4, and filtered. The solvent was removed in rotary evaporator and the resulting crude product was recrystallized from the mixture of 10% EtOAc in n-heptane to obtain the phosphine oxide as white solid (**L5-S1**) (22.15 g, 90% yield). ^{1}H NMR (300 MHz, $CDCl_3$) δ 8.02 (d, J = 477.3 Hz, 1H), 7.60 (dd, J = 13.1, 8.9 Hz, 4H), 6.98 (dd, J = 8.9, 2.2 Hz, 4H), 3.84 (s, 6H). ^{31}P NMR (122 MHz, $CDCl_3$) δ 20.59. EI-HRMS (m/z): Calculated for [$C_{14}H_{15}O_3P$]: 263.0837; found: 263.0844.

Step-2: To an oven dried 1 L three-neck round bottom flask equipped with an addition funnel, DIBAL-H (296 mL, 3.5equiv, 1.0 M in hexane) was transferred through cannula under argon atmosphere. The reaction mixture was cooled to 0 °C, and phosphine oxide (**L5-S1**) (22.15 g, 84.46 mmol) was added portion wise (with addition of each portion, H_2 evolution was observed) carefully, followed by the addition of freshly degassed THF (70 mL). After the addition, the reaction mixture was stirred at ambient temperature for 3 hours. Then, the reaction mixture was cooled to 0 °C and freshly degassed Et_2O (100 mL) was added to the reaction mixture via addition funnel. 2 N aq. NaOH (320 mL) (freshly degassed) was also added via additional funnel over 35 minutes (vigorous gas evolution), followed by the addition of 70 mL saturated aq. NaCl (freshly degassed). The mixture was stirred for additional 20 minutes at room temperature. The organic phase was then transferred via cannula to a 1 L dried round bottom flask which was pre-charged with $MgSO_4$ under argon. The aqueous phase was again extracted with freshly degassed Et_2O (150 mL) and the organic phase was transferred to the above mentioned round bottom flask and stirred for 5 minutes. The organic mixture was filtered through celite, and the solvent was removed in vacuum to obtain semi-solid, the phosphine hydride was confirmed by ^{31}P NMR and carried the next step without further purification (**L5-S2**) (20 g, 96% yield). ^{1}H NMR (300 MHz, CD_3CN) δ 7.57

– 7.48 (m, 4H), 7.09 – 6.95 (m, 4H), 5.24 (d, J = 218.7 Hz, 1H), 3.87 (s, 6H). ^{31}P NMR (122 MHz, CD_3CN) δ -45.22.

Step-3: To an oven dried 1 L three-neck round bottom flask, bis(4-methoxyphenyl)phosphane (**L5-S2**) (20 g, 81.22 mmol), KO^{t}Bu (9.11 g, 81.22 mmol) and freshly degassed DMSO (200 mL) were added under argon. The dark red mixture was stirred for 30 minutes at ambient temperature. To this mixture, 1,1,1-tris(chloromethyl)ethane (3.56 g, 20.30 mmol) was added dropwise via syringe, and the reaction mixture was stirred at 60 °C for overnight. Discoloration of the dark red solution was observed during course of the reaction. The reaction mixture was cooled to 0 °C, degassed water (200 mL) was added and stirred for 30 minutes. The water/DMSO mixture was extracted with EtOAc (100 mL x 3), the combined organic phase was washed with brine (150 mL), dried over Na_2SO_4, and filtered. The solvent from filtrate containing product was removed in vacuum and the resulting solid obtained was recrystallized from the mixture of EtOAc and MeOH as a white solid (**L5**) (14.5 g, 89% yield).
^{1}H NMR (300 MHz, $CDCl_3$) δ 7.27 (dd, J = 8.8, 7.1 Hz, 12H), 6.83 – 6.76 (m, 12H), 3.78 (s, 18H), 2.34 (d, J = 2.8 Hz, 6H), 0.93 (s, 3H). ^{31}P NMR (122 MHz, $CDCl_3$) δ -29.90. EI-HRMS (m/z): Calculated for [$C_{47}H_{51}O_6P_3$]: 804.28930; found: 804.29086.

Synthesis of L6

Step-1: An oven dried 500 mL three-neck round bottom flask equipped with reflux condenser and addition funnel was charged with magnesium turnings (3.81 g, 156.7 mmol) and I_2 (1 crystal) under argon flow and then THF (200 mL) was added. To this pale brown solution, 20 mL of 1-bromo-4-methoxybenzene (25 g, 130.50 mmol, dissolved in 50 mL THF) was added dropwise via the addition funnel and the Grignard reagent formation was initiated by heating with a heat gun. Subsequently, remaining 30 mL of 1-bromo-4-chlorobenzene solution was added dropwise and the reaction mixture was stirred at 75 °C for 3 hours. Then the reaction mixture was cooled to 0 °C and then diethyl phosphite (6.31 g, 45.70 mmol) was added dropwise to the reaction mixture via addition funnel over 15 minutes. The whole mixture was stirred for overnight at ambient temperature. The reaction was quenched with 0.5 N HCl (200 mL) at 0 °C and extracted with EtOAc (100 mL x 2). The combined organic layers were washed with brine (200 mL), dried over Na_2SO_4 and filtered. The solvent from the filtrate was removed in rotary evaporator and the resulting crude product was recrystallized with n-heptane to obtain the phosphine oxide as white solid (**L6-S1**) (9.9 g, 80% yield). ^{1}H NMR (300 MHz, $CDCl_3$) δ 8.05 (d, J =

487.3 Hz, 1H), 7.66 – 7.57 (m, 4H), 7.51 – 7.46 (m, 4H). ^{31}P NMR (122 MHz, $CDCl_3$) δ 18.90. EI-HRMS (m/z): Calculated for [$C_{12}H_9OCl_2P$]: 270.97640; found: 270.97664.

Step-2: DIBAL-H (128 mL, 3.5equiv, 1.0 M in hexane) was transferred through cannula to an oven dried 500 mL three-neck round bottom flask, which was equipped with an addition funnel and maintained under argon atmosphere. The reaction mixture was cooled to 0 °C, and phosphine oxide (**L6-S1**) (9.9 g, 36.52 mmol) was added portion wise (with addition of each portion H_2 evolution was observed) carefully, followed by the addition of freshly degassed THF (70 mL) under argon atmosphere. After the addition, the reaction mixture was stirred at ambient temperature for 3 hours and then cooled to 0 °C. To this mixture, freshly degassed Et_2O (50 mL) was added via addition funnel. Subsequently, 2 N aq. NaOH (140 mL) (freshly degassed) was added via additional funnel over 20 minutes (vigorous gas evolution was observed), followed by the addition of 40 mL saturated aq. NaCl (freshly degassed). The mixture was stirred for additional 20 minutes at room temperature. After the reaction, the organic phase was transferred via cannula to a 500 mL dried round bottom flask, which was pre-charged with $MgSO_4$ under argon. The aqueous phase was again extracted with freshly degassed Et_2O (70 mL) and the organic phase was transferred to the above mentioned 500 mL round bottom flask and stirred for 5 minutes. The organic mixture was filtered through celite, and the solvent was removed in vacuum to obtain semi-solid, the phosphine hydride, which was confirmed by ^{31}P NMR and carried next step without further purification (**L6-S2**) (8.4 g, 90% yield). ^{1}H NMR (300 MHz, CD_3CN) δ 7.49 – 7.42 (m, 4H), 7.38 – 7.32 (m, 4H), 5.21 (d, J = 222.5 Hz, 1H). ^{31}P NMR (122 MHz, CD_3CN) δ -43.84.

Step-3: A 500 mL two-neck round bottom flask was charged with bis(4-chlorophenyl)phosphane (**L6-S2**) (8.4 g, 32.93 mmol), KO^{t}Bu (3.69 g, 32.93 mmol) and freshly degassed DMSO (100 mL) under argon. The dark red mixture was stirred for 30 minutes at ambient temperature. To this mixture, 1,1,1-tris(chloromethyl)ethane (1.44 g, 8.23 mmol) was added dropwise via syringe, and the reaction mixture was stirred at 80 °C for overnight. Discoloration of the dark red solution was observed during the course of the reaction. Then reaction mixture cooled to 0 °C and added degassed water (100 mL) and stirred for 30 minutes. The water/DMSO mixture was extracted with EtOAc (100 mL x 2), the combined organic phase was washed with brine (100 mL) and dried over Na_2SO_4 and filtered. The solvent from the filtrate was removed in vacuum and obtained solid was recrystallized from the mixture of Et_2O and MeOH as a white solid (**L6**) (4 g, 59% yield). ^{1}H NMR (400 MHz, $CDCl_3$) δ 7.33 - 7.28 (m, 12H), 7.24 - 7.19 (m,

12H), 2.27 (d, J = 3.3 Hz, 6H), 1.05 (s, 3H). ^{31}P NMR (162 MHz, $CDCl_3$) δ -27.62. Calculated for [$C_{41}H_{33}Cl_6P_3$]: 831.34953; found: 831.65243.

3. General procedure for the hydrogenative amination of esters or carboxylic acids with ammonia

To an oven dried 8 mL glass vial, a magnetic stir bar, ester or carboxylic acid (0.5 mmol), 8 mol% $Co(BF_4)_2.6H_2O$, 2.1 equiv. of L5 to [Co] and $Al(O\text{-}iPr)_3$ (20 mol%) were added in glove box. Then the vial was fitted with septum, cap and needle and HFIP (1 mL, distilled and degassed) was added via syringe under argon atmosphere. The reaction vials (8 vials with different substrates at a time) were placed into a 300 mL autoclave. The autoclave was flushed with hydrogen 2 times with 20 bar pressure, and it was pressurized with 5 bar ammonia gas and 60 bar hydrogen. The autoclave was placed into an aluminium block, which was pre-heated at 160 °C and the reactions were allowed to progress for the required time under stirring condition. During the course of reaction, the inside temperature of the autoclave was measured to be 140 °C and this temperature was considered as the reaction temperature. After the completion of reactions, the autoclave was cooled to room temperature, remaining hydrogen was slowly discharged and the vials containing reaction products were removed from the autoclave. The reaction products were analyzed by GC and GC-MS. The resulting products (corresponding primary amines) were purified by column chromatography (MeOH/DCM) and converted to their hydrochloride salts and characterized by NMR. For the preparation of the amine-hydrochloride salts, 1-2 mL methanolic HCl (0.5M HCl in methanol) was added to the isolated free amine and stirred at room temperature for 4-5 h. Then, the solvent was removed, and the solid product was washed twice with ethyl acetate and the resulted amine-hydrochloride salt was dried under vacuum. Some primary amines were characterized as free amines by NMR. The yields for selected primary amines were determined by GC. For this purpose, mesitylene (27 μL, 0.2 mmol) as a GC standard was added to the reaction vials and the reaction mixture was diluted with DCM followed by filtration using plug of silica and the filtrate containing product was subjected to GC analysis.

4. Hydrogenative amination of triglycerides and vegetable oils using ammonia

To an 8 mL glass vial, a magnetic stir bar, triglyceride or vegetable oil (0.1 mmol) and $Co(BF_4)_2.6H_2O$ (6 mol% for each ester group), 2.1 equiv. of **L5** to [Co] and $Al(O\text{-}iPr)_3$ (20 mol% for each ester group) were transferred in glove box. Then the vial was fitted with septum, cap and needle and HFIP (2 mL, distilled and degassed) was added via syringe under argon atmosphere. The reaction vials (8 vials with different substrates at a time) were placed into a 300 mL autoclave. The autoclave was flushed with hydrogen 2 times with 20 bar pressure, and it was pressurized with 5 bar ammonia gas and 60 bar hydrogen. The autoclave was placed into an aluminium block, which was pre-heated at 160 °C and the reactions were allowed to progress for the required time under stirring condition. During the course of reaction, the inside temperature of the autoclave was measured to be 140 °C and this temperature was considered as the reaction temperature. After the completion of reactions, the autoclave was cooled to room temperature, remaining hydrogen was slowly discharged and the vials containing reaction products were removed from the autoclave. The reaction products were analyzed by GC and GC-MS. The selected products (corresponding primary amines) were purified by column chromatography (MeOH/DCM) and characterized by NMR.

GC caliberation for Vegetable oils: Vegetable oil contains three fatty ester unit with glycerol, For GC-caliberation 0.3 mmol of one fatty amine unit was considered and then correlated with percentage composition of corresponding oil fatty acid essay (Supporting information, Section 14). For this purpose, after the reaction mesitylene, GC standard was added to the reaction vials and the reaction mixture was diluted with DCM followed by filtration using plug of silica and the filtrate containing product was subjected to GC analysis.

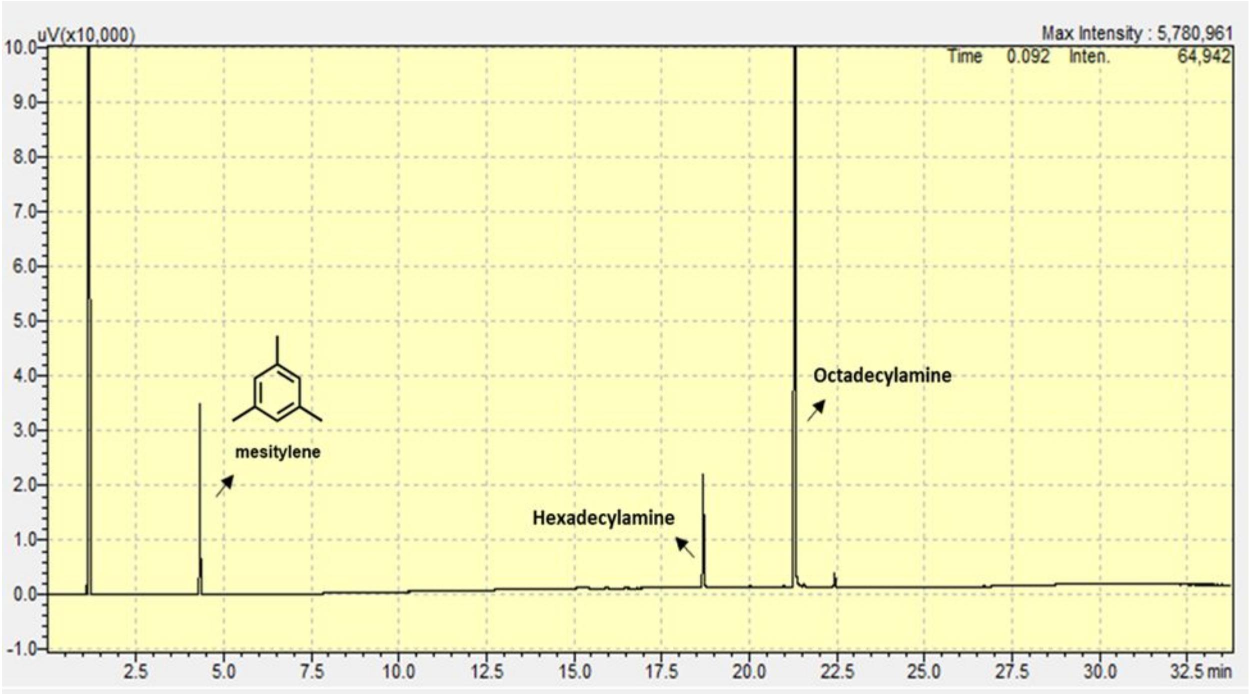

Figure S1. GC-FID spectra after the reaction-Olive oil

5. Reaction optimization

Table S1. Hydrogenative amination of methyl benzoate using *in situ* metal-phosphine complexes: Testing of different metal precursors and ligands.

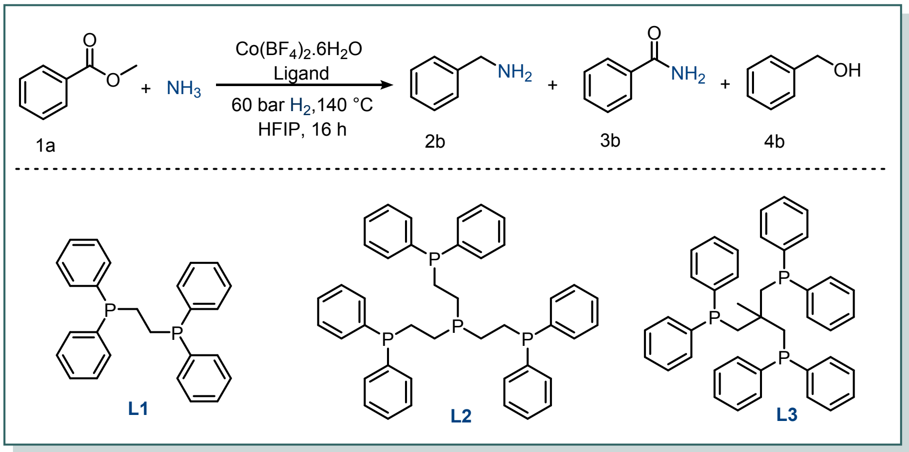

Entry	Metal precursor-Ligand	Conv. (%) 1a	Yield (%) 2b	3b	4b
1	$Fe(BF_4)_2.6H_2O$-**L1**	78	-	63	14
2	$Fe(BF_4)_2.6H_2O$-**L2**	76	-	56	19
3	$Fe(BF_4)_2.6H_2O$-**L3**	84	-	68	15
4	$Mn(OTf)_2$-**L1**	92	-	81	10
5	$Mn(OTf)_2$-**L2**	92	-	76	15
6	$Mn(OTf)_2$-**L3**	96	-	85	10
7	$Co(BF_4)_2.6H_2O$-**L1**	85	-	60	24
8	$Co(BF_4)_2.6H_2O$-**L2**	78	-	58	19
9	$Co(BF_4)_2.6H_2O$-**L3**	89	25	55	8
10	$Ni(BF_4)_2.6H_2O$-**L1**	78	-	62	15
11	$Ni(BF_4)_2.6H_2O$-**L2**	56	-	44	11
12	$Ni(BF_4)_2.6H_2O$-**L3**	60	-	45	14
13	$Cu(BF_4)_2.xH_2O$-**L1**	78	-	58	19
14	$Cu(BF_4)_2.xH_2O$-**L2**	61	-	48	12
15	$Cu(BF_4)_2.xH_2O$-**L3**	69	-	47	21

Reaction conditions: 0.3 mmol methyl benzoate, 5 bar NH_3, 8 mol% metal precursor, 16 mol% ligand, 60 bar H_2, 1 mL HFIP, 140 °C, 16 h. Conversion and yields were determined by GC using mesitylene as standard.

Table S2. Hydrogenative amination of methyl benzoate using *in situ* and molecularly defined cobalt-phosphine complexes: Testing of different triphos ligands.

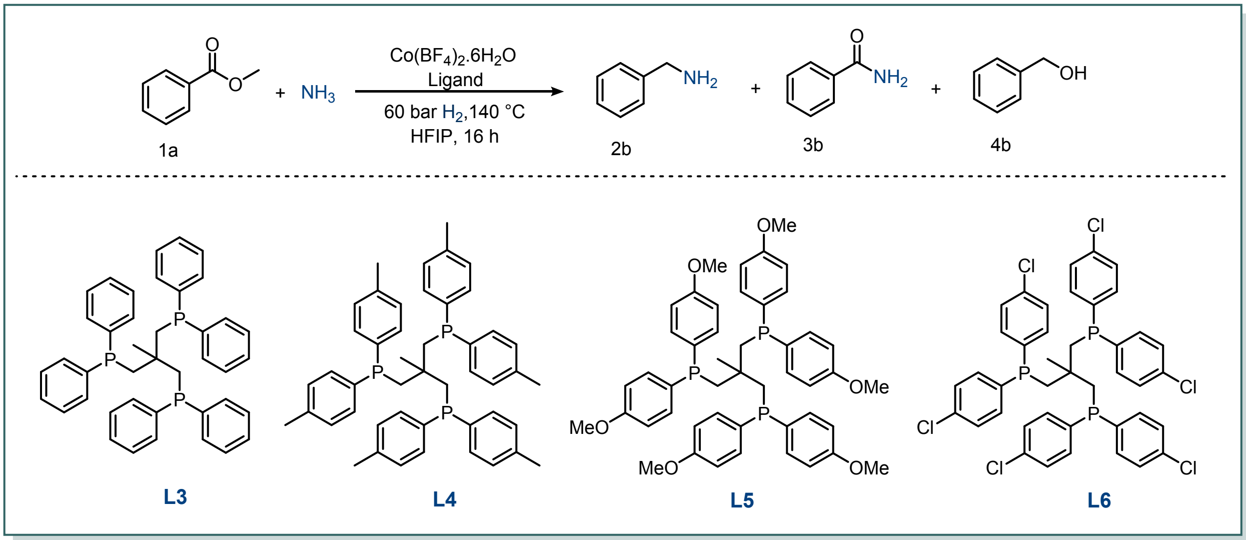

Entry	Co-L	Conv.(%)	Yield (%)		
		1a	2b	3b	4b
1	$Co(BF_4)_2.6H_2O$-**L3**	89	25	55	8
2	$Co(BF_4)_2.6H_2O$-**L4**	85	65	12	7
3	$Co(BF_4)_2.6H_2O$-**L5**	98	79	11	4
4	$Co(BF_4)_2.6H_2O$-**L6**	70	10	48	10
5	Co-**L5** (1:1.1)	54	11	39	2
6	Co-**(L5)** (1:2.1)	>99	82	10	5

Reaction conditions: 0.3 mmol methyl benzoate, 5 bar NH_3, 8 mol% $Co(BF_4)_2.6H_2O$, 16 mol% ligand, 60 bar H_2, 1 mL HFIP, 140 °C, 16 h. Conversions and yields were determined by GC using mesitylene as standard.

Table S3. Hydrogenative amination of methyl benzoate: Screening of different additives.

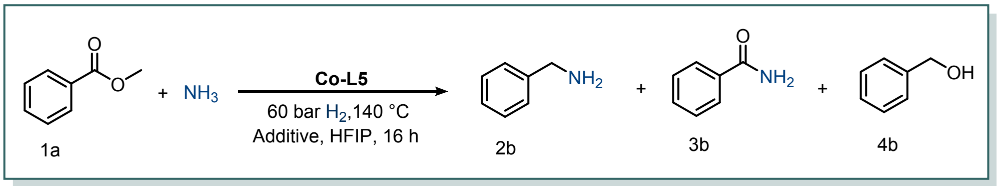

Entry	Additive	Conv. (%) 1a	Yield (%) 2b	3b	4b
1	Without additive	>99	82	10	5
2	K_2CO_3	90	66	4	13
3	KO^{t}Bu	91	70	12	5
4	PTSA	95	83	7	2
5	Al(O-*i*Pr)$_3$	>99	92	5	2
7	$HNTf_2$	90	77	12	-
8	$Al(OTf)_3$	92	78	10	2
9	$AlCl_3$	85	69	8	4

Reaction conditions: 0.3 mmol methyl benzoate, 8 mol% $Co(BF_4)_2.6H_2O$, 2.1 equiv. **L5** to [Co], 5 bar NH_3, 20 mol% additive, 60 bar H_2, 1 mL HFIP, 140 °C, 16 h. Conversion and yields were determined by GC using mesitylene as standard.

Table S4. Hydrogenative amination of methyl benzoate: Screening of solvents.

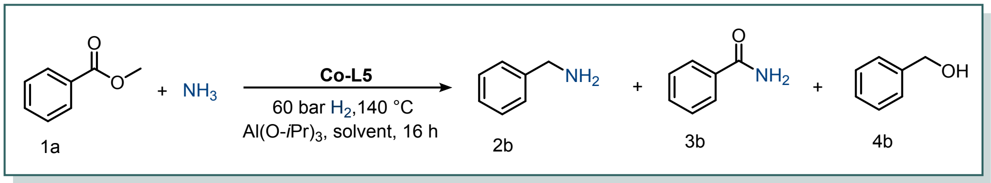

Entry	Solvent	Conv. (%) 1a	Yield (%) 2b	3b	4b
1	Toluene	17	-	9	-
2	THF	22	-	12	-
3	t-amyl alcohol	47	-	28	-
4	MeOH	52	-	39	-
5	i-PrOH	73	-	22	-
6	Trifluoroethanol (TFE)	83	58	22	-
7	Hexafluoroisopropanol (HFIP)	>99	92	5	2

Reaction conditions: 0.3 mmol methyl benzoate, 8 mol% $Co(BF_4)_2.6H_2O$, 2.1 equiv. **L5** to [Co], 20 mol% $Al(O\text{-}iPr)_3$, 5 bar NH_3, 60 bar H_2, 1 mL solvent, 140 °C, 16 h. Conversion and yields were determined by GC using mesitylene as standard.

Table S5. Hydrogenative amination of methyl benzoate: Different catalyst loading

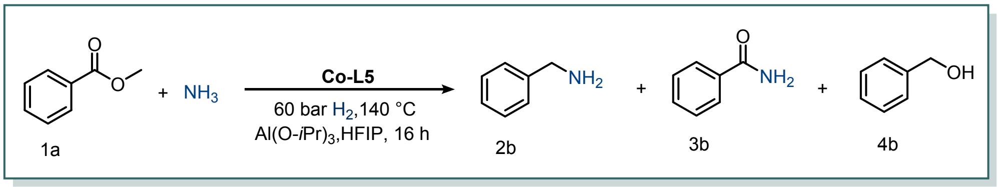

Entry	Catalyst loading [Co]	Conv. (%) 1a	Yield (%)		
			2b	3b	4b
1	4 mol%	60	42	13	-
2	6 mol%	89	68	16	-
3	8 mol%	>99	92	5	2
4	10 mol%	>99	93	4	2

Reaction conditions: 0.3 mmol methyl benzoate, 2.1 equiv. **L5** to [Co], 20 mol% $Al(O\text{-}iPr)_3$, 5 bar NH_3, 60 bar H_2, 1 mL HFIP, 140 °C, 16 h. Conversion and yields were determined by GC using mesitylene as standard.

6. Recycling of HFIP solvent

After the completion of reaction under standard conditions, HFIP solvent was recovered under rotary evaporator. Recovered solvent was distilled twice (40-50 °C, under vacuum (5 millibar)) under molecular sieves (3 Å) and stored under argon. Recycled solvent was used for reactions and final product was obtained in similar yield of reaction performed in fresh (distilled as well as degassed) HFIP solvent[5].

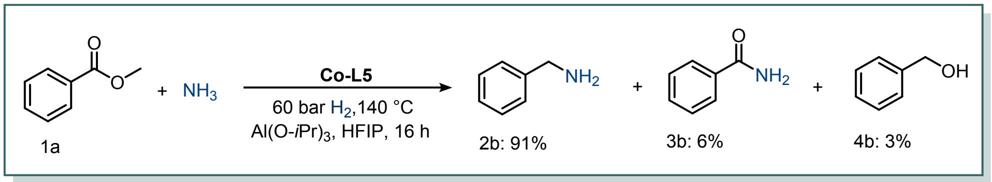

Figure S2. Recycling of solvent

Reaction conditions: 0.3 mmol methyl benzoate, 8 mol% $Co(BF_4)_2.6H_2O$, 2.1 equiv. **L5** to [Co], 20 mol% $Al(O\text{-}iPr)_3$, 5-7 bar NH_3, 60 bar H_2, 1 mL HFIP, 140 °C, 16 h. Conversion and yields were determined by GC using mesitylene as standard.

7. Catalyst stability experiment

Under optimized conditions, the hydrogenative amination of methyl benzoate was performed at 180 °C.

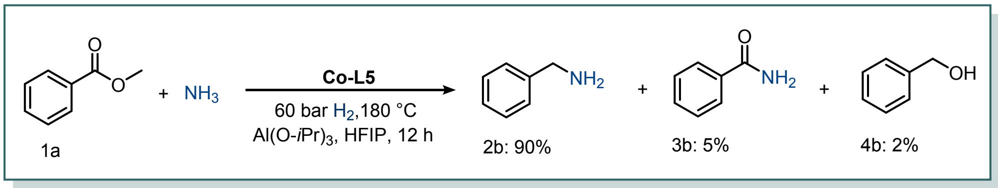

Figure S3. Using Co-L5 at higher temperature.

Reaction conditions: 0.3 mmol methyl benzoate, 8 mol% $Co(BF_4)_2.6H_2O$, 2.1 equiv. L5 to [Co], 5 bar NH_3, 20 mol% Al(O-*i*Pr)$_3$, 60 bar H_2, 1 mL HFIP, 180 °C, 12 h. Conversion and yields were determined by GC using mesitylene as standard.

8. High pressure NMR experiments

The reaction of $Co(BF_4)_2{\cdot}6H_2O$ with **L5** (at a 1:2 metal-to-ligand ratio) was investigated, with the addition of hydrogen (60 bar) under varying temperatures using *in situ* NMR spectroscopy. Initial experiments were conducted in a Young NMR tube under argon atmosphere and the mixture of $Co(BF_4)_2{\cdot}6H_2O$ and **L5** was analyzed. The formation of a $[Co_2(triphos)_2(\mu\text{-}OH)_2](BF_4)_2$ complex, which exhibits antiferromagnetic properties, is reported when $Co(BF4)_2{\cdot}6H_2O$ and triphos are mixed in a metal-to-ligand ratio of 1:1.[6] However, using two equivalents of L5 in the current system resulted in a mixture of complexes. Specifically, the ^{31}P NMR spectrum showed that only a small fraction of the triphos ligand initially coordinated to the metal (**Figure S4**). Moreover, an intense broad resonance corresponding to non-coordinated P-atoms was observed at 31.39 ppm. The broadening of the phosphorus signals can be attributed to the coupling of the quadrupolar Co nucleus. Following the addition of hydrogen at room temperature (297 K) within a sapphire NMR tube, a clear broad signal manifested in the hydride region of the ^{1}H NMR spectrum at -12.9 ppm (**Figure S5a**). The corresponding ^{31}P NMR spectrum shows a decrease in the intensity of the signal attributed to non-coordinating P-atoms of L5 at -31.3 ppm, and the resonance at -0.5 ppm decreases (**Figure S5 b**). At the same time, a new broad resonance in the region for coordinated P ligand was detected at 20.4 ppm (**Figure S5b**). This suggests the formation of a monohydride Co complex [HCo(triphos(p-anisole))(L)], by heterolytic cleavage of the H-H bond, where L could be a P atom from another triphos ligand, given the addition of excess equivalents of ligand were added with respect to Co.[7] The performance of NMR experiments at 120 °C (393 K) under 60 bar

hydrogen revealed complications with the lock and shimming, indicating the potential formation of paramagnetic species, particularly at elevated temperatures.

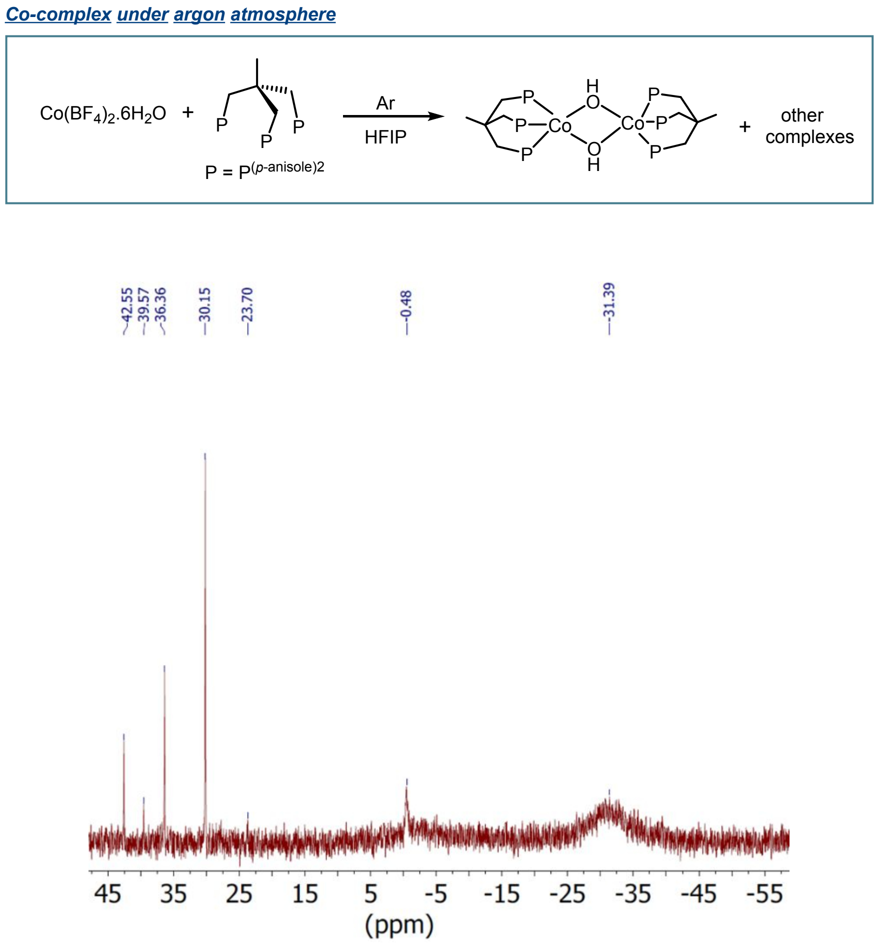

Figure S4. $^{31}P\{^{1}H\}$-NMR spectrum of initially formed cobalt complexes in a 5 mm J. Young NMR tube under argon. Conditions: $Co(BF_4)_2.6H_2O$ = 20 mM, **L5** = 40 mM, solvent: HFIP-d_2/HFIP (1:1).

As the temperature increased, a doublet in the hydride region of the ^{1}H NMR spectrum was detected at -12.8 ppm. $^{1}H\{^{31}P\}$ measurements confirmed the 2J(^{31}P,^{1}H) coupling as illustrated in **Figure S5**. The value of 74.6 Hz corresponds to trans coupling in a distorted geometry and is assigned to a dihydrogen cobalt complex $[Co(H_2)(triphos(p\text{-}anisole))(L)]^+$. We assume the formation of this complex could happen during the catalytic reaction through the oxidative addition of H_2 to $[Co_2(triphos)_2(\mu\text{-}OH)_2](BF_4)_2$. Such complexes have been previously reported for a similar system.[8] In the ^{31}P NMR spectrum at 120 °C, most of the signals of coordinated phosphorus could not be detected most likely due to broadening of the phosphorus signals, which is a known feature at increased temperatures.[7] Intriguingly, a signal at -17.4 ppm, corresponding to non-coordinating P-atoms became discernible at 40 °C. After cooling down the mixture to rt (297 K), a new broad signal in the ^{31}P NMR spectrum at -5.8 ppm was detected.

Concurrently, the doublet in the hydride region of the ^{1}H NMR spectrum became more pronounced (**Figure S5b**). No further signals were detected in the region for non-coordinated phosphorus species.

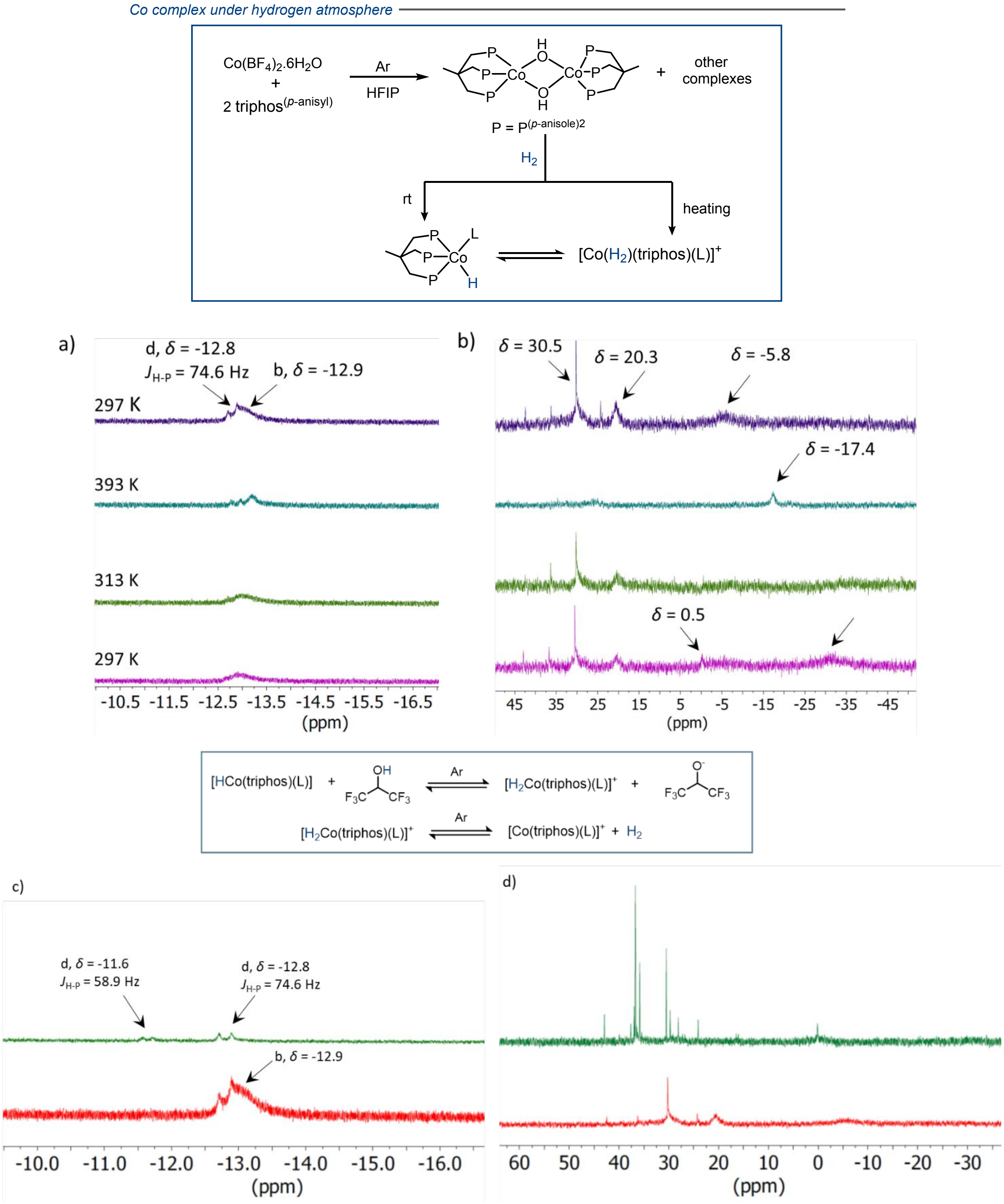

Figure S5. a) ^{1}H-NMR spectra of cobalt hydride complexes in a 5 mm sapphire tube after pressurizing with H_2 under variation of the temperature. b) ^{31}P{^{1}H}-NMR spectra of cobalt hydride complexes in a 5 mm sapphire tube after pressurizing

with H_2 under variation of the temperature. c) ^{1}H-NMR, hydride region and d) $^{31}P\{^1H\}$-NMR spectra of cobalt hydride complexes under H_2 (down, red) and argon (up, green). Conditions: $Co(BF_4)_2.6H_2O$ = 20 mM, **L5** = 40 mM, 60 bar H_2 or argon atmosphere, solvent: HFIP-d_2/HFIP (1:1).

Furthermore, we performed experiments to investigate the stability of in situ formed Co-H complex. Subsequent to the high-pressure measurements, hydrogen was released, and the solution was transferred into a Young NMR tube under argon atmosphere. Then, the broad signal of the main complex in the hydride region of the ^{1}H NMR spectrum at -12.9 ppm, attributed to [HCo(triphos(p-anisole))(L)] vanished completely (**Figure S5c and d**). The lability of this type of complex can be explained by its tendency to be protonated under acidic conditions to form the Co(III) dihydride [H_2Co(triphos(p-anisole))(L)], followed by the irreversible loss of H_2.[7] This process is favored under an inert atmosphere but is inhibited under hydrogen. Furthermore, the doublet assigned to the stable hydrogen complex [Co(H_2)(triphos(p-anisole))(L)]$^+$ at -12.8 ppm decreased notably in intensity and a new doublet appeared at δ= -11.6 ppm (2JP-H = 58.9 Hz), which can be attributed to an isomeric form.

9. EPR measurements

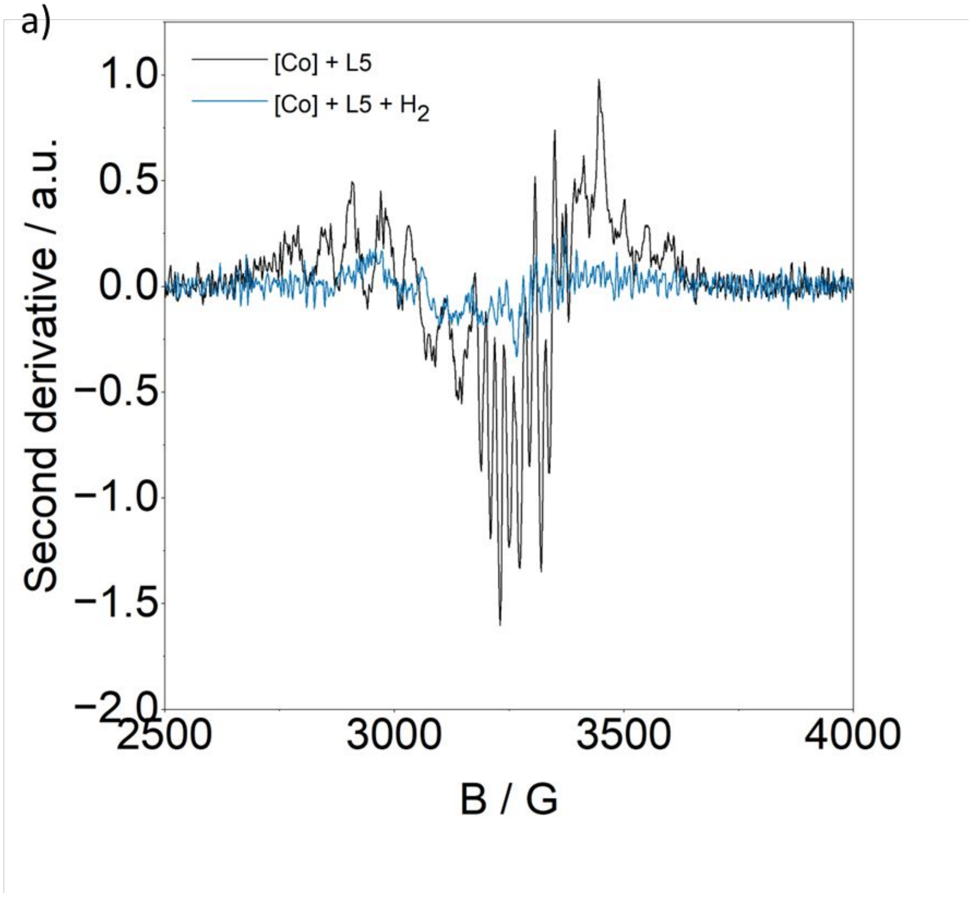

b)

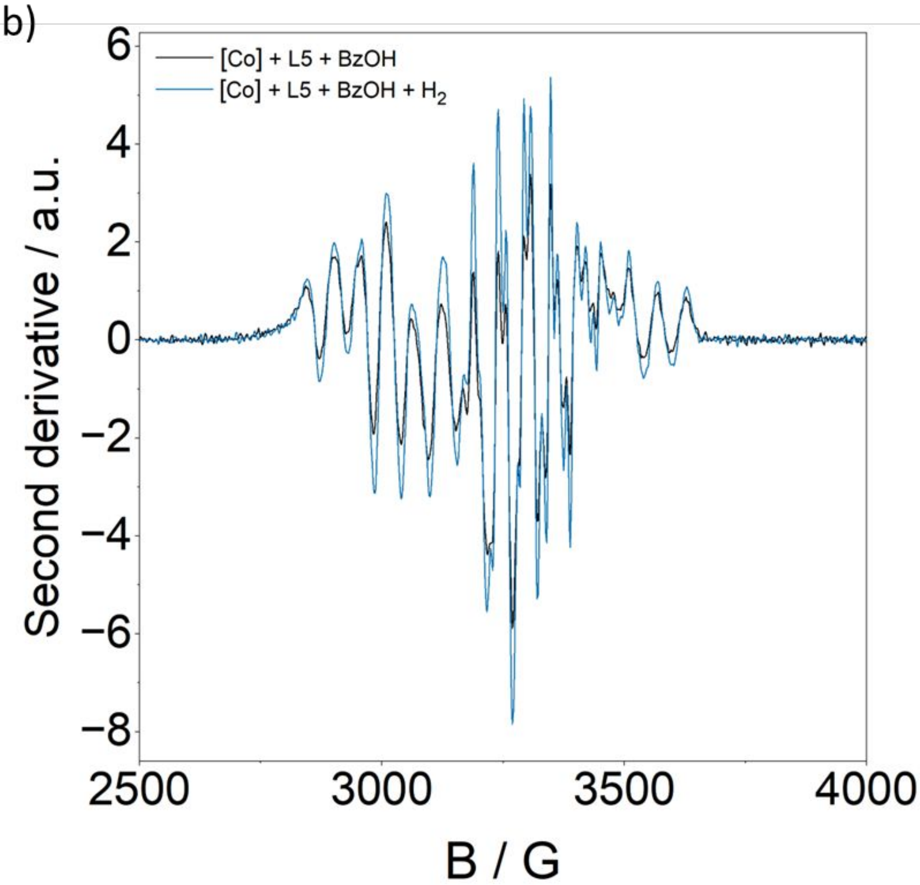

Figure S6. a) Second derivative EPR spectra of [Co] + **L5** and [Co] + **L5 +** hydrogen. b) Second derivative EPR spectra of [Co] + **L5** + benzoic acid and [Co] + **L5** + benzoic acid + hydrogen.

10. ESI-MS analysis

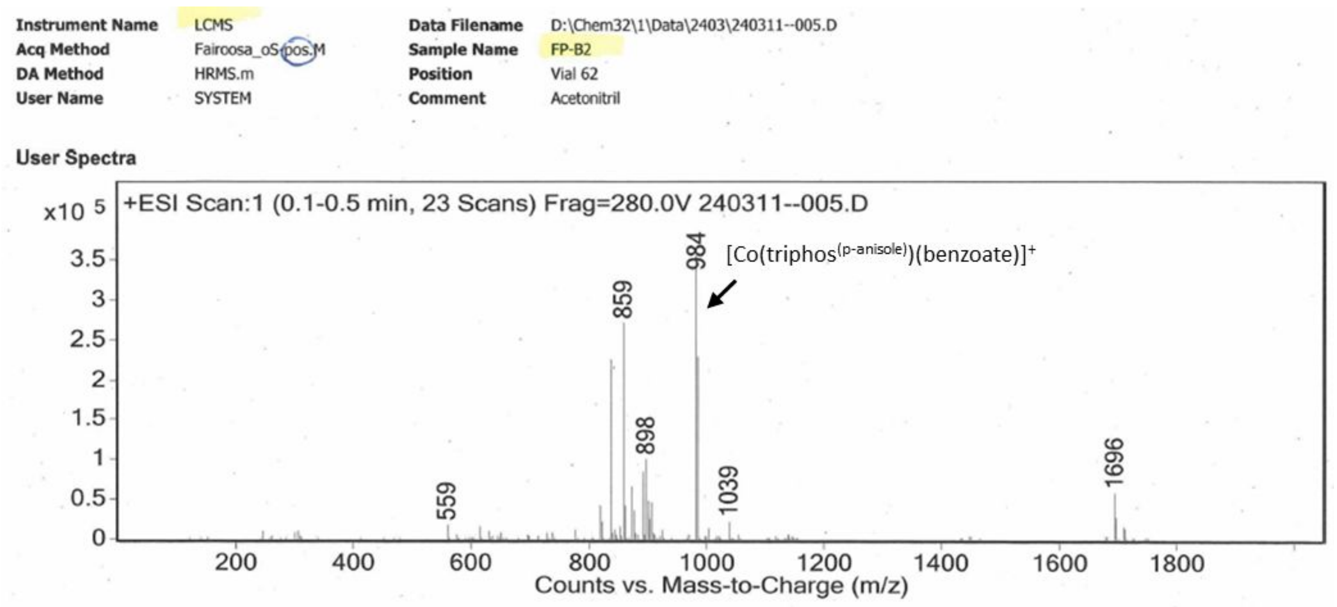

Figure S7. ESI-MS spectra of reaction sample after 1 hour

11. Catalyst poisoning experiment

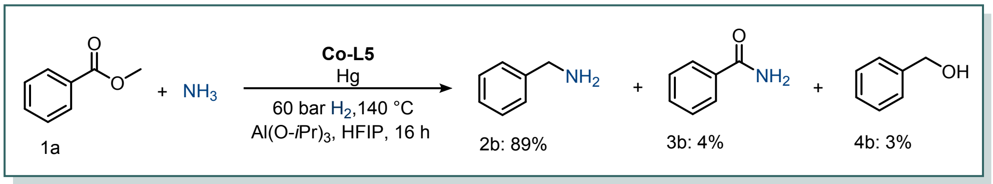

Figure S8. Catalyst poisoning experiment.

Reaction conditions: 0.3 mmol methyl benzoate, 5-7 bar NH_3, 8 mol% $Co(BF_4)_2.6H_2O$, 2.1 equiv. L5 to [Co], 20 mol% Al(O-iPr)$_3$, 2 equiv. of Hg, 60 bar H_2, 1 mL HFIP, 140 °C, 16 h. Conversion and yields were determined by GC using mesitylene as standard.

12. Extended substrate scope for hydrogenative amination of triglycerides

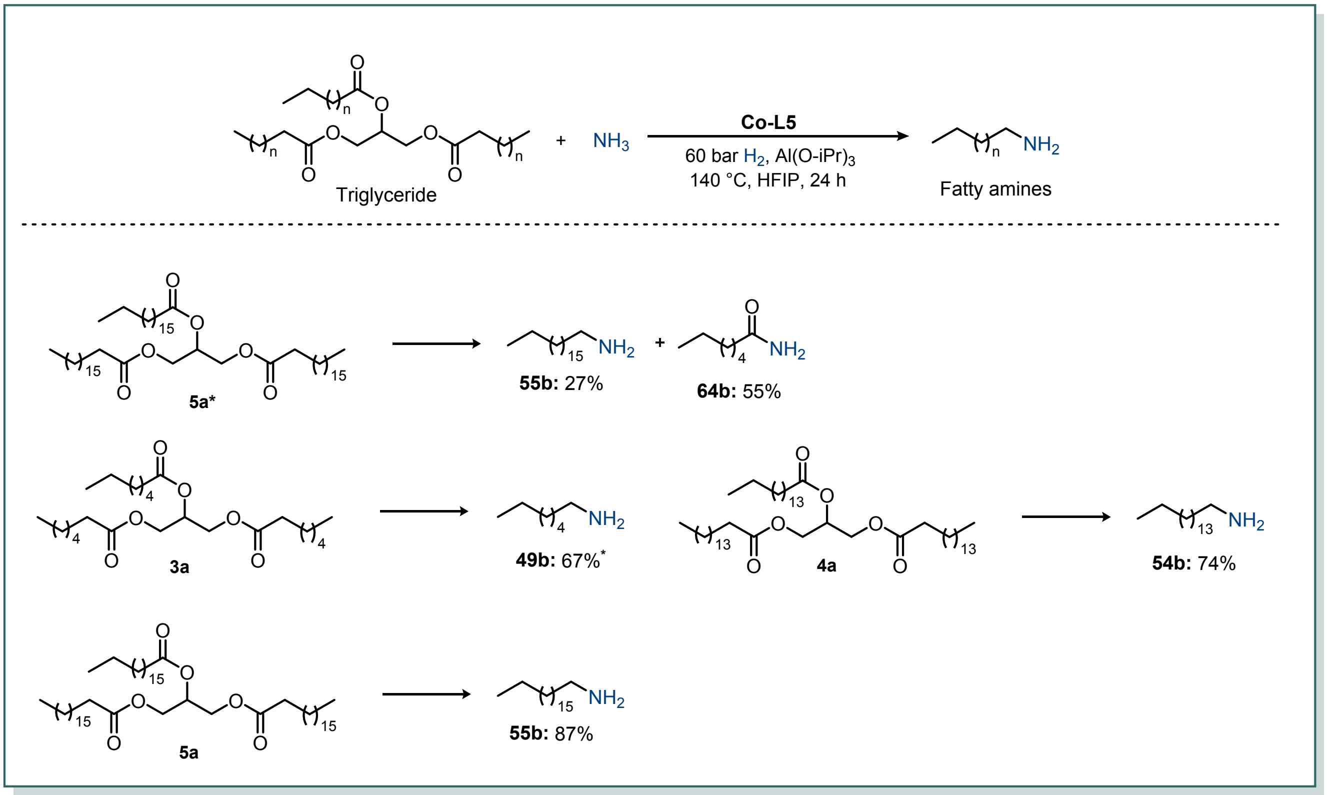

Figure S9. Hydrogenative amination of triglycerides using ammonia.

Reaction conditions. *0.1 mmol triglyceride, 5 bar NH_3, 8 mol% $Co(BF4)_2.6H_2O$, 2.1 equiv. **L5** to [Co], 20 mol% Al(O-iPr)$_3$, 60 bar H_2, 2 mL HFIP, 140 °C, 16 h, isolated yields.

0.1 mmol triglyceride, 5 bar NH_3, 6 mol% $Co(BF_4)_2.6H_2O$ for each ester group, 2.1 equiv. **L5** to [Co], 20 mol% Al(O-iPr)$_3$ for each ester group, 60 bar H_2, 2 mL HFIP, 140 °C, 24 h, isolated yields. *GC yields were determined by using mesitylene as standard.

13. Extended control experiments

The control experiments were performed using methyl benzoate and sunflower oil. The reaction performed using methyl benzoate in the absence of hydrogen and cobalt catalyst produced 88% primary amide with excellent conversion (Figure 1a). However, in case of vegetable oil (sunflower oil) very little conversion (11%) and only traces of the corresponding amide were observed (Figure 1b). The hydrogenation of methyl benzoate and sunflower oil in the absence of ammonia, gave the desired alcohol was obtained in a very good yield (Figures 1c and 1d). 88% of total fatty alcohol yield was obtained when sunflower oil was hydrogenated under mentioned conditions suggesting the possibility of an aldehyde intermediate forming during the reaction (Figure 1d).

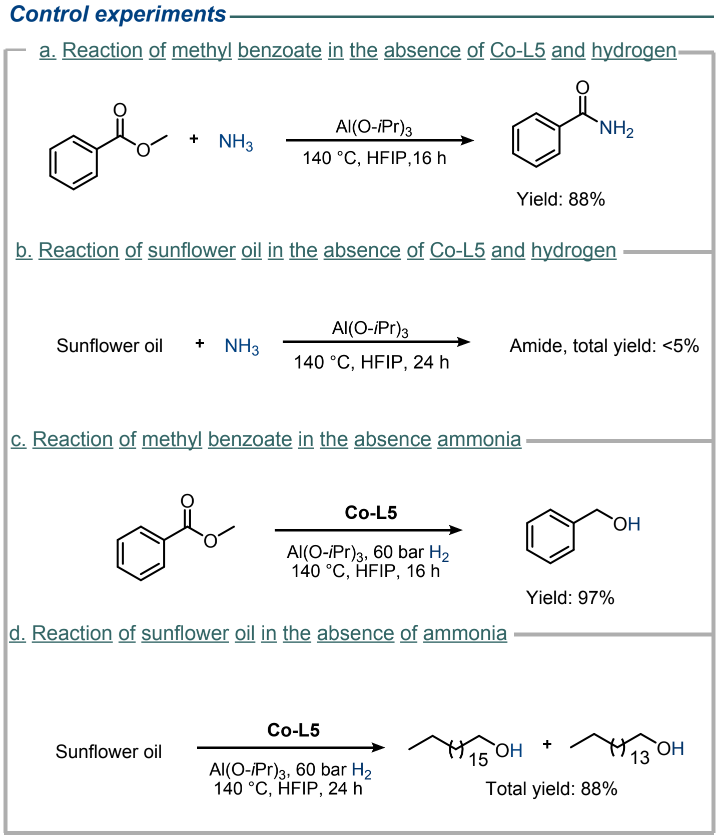

Figure S10. Control experiments using methyl benzoate and sunflower oil.

Reaction conditions. a) 0.5 mmol methyl benzoate, 5 bar NH_3, 20 mol% Al(O-iPr)$_3$, 2 mL HFIP, 140 °C, 16 h. b) 0.1 mmol sunflower oil, 5 bar NH_3, 20 mol% Al(O-iPr)$_3$ for each ester unit, 2 mL HFIP, 140 °C, 24 h. c) 0.5 mmol methyl benzoate, 8 mol% Co(BF$_4$)$_2$.6H_2O, 2.1 equiv. L5 to [Co], 20 mol% Al(O-iPr)$_3$, 60 bar H_2, 2 mL HFIP, 140 °C, 16 h. d) 0.1 mmol sunflower oil, 6 mol% Co(BF$_4$)$_2$.6H_2O for each ester group, 2.1 equiv. **L5** to [Co], 20 mol% Al(O-iPr)$_3$ for each ester group, 60 bar H_2, 2 mL HFIP, 140 °C, 24 h. *GC yields were determined by using mesitylene as standard.

14. Composition of vegetable oils

Table S6.

Entry	Vegetable oil	Fatty acid essay
1	Palm oil	39.4% C16 fatty acid (39.2% saturated, 0.2% unsaturated)<br>58% C18 fatty acid (5.4% saturated, 52.6% unsaturated)
2	Sunflower oil	5.9% C16 fatty acid (3.1% saturated, 2.8% unsaturated)<br>92.4% C18 fatty acid (3.1% saturated, 89.3% unsaturated)
3	Rapeseed oil	6.1% C16 fatty acid (6.0% saturated, 0.1% unsaturated)<br>91.9% C18 fatty acid (29.9% saturated, 58.9% unsaturated)

4	Olive oil	15.1% C16 fatty acid (14.3% saturated, 0.8% unsaturated)<br>83.4% C18 fatty acid (3.4% saturated, 80% unsaturated)
5	Coconut oil	47.7% C12 fatty acid<br>19.4% C14 fatty acid<br>7.7% C16 fatty acid<br>8.9% C18 fatty acid<br>(3.1% saturated, 5.8% unsaturated)
6	Pumpkin seed oil	11.2% C16 fatty acid (11.1% saturated, 0.1% unsaturated)<br>87.8% C18 fatty acid (5.5% saturated, 82% unsaturated)
7	Walnut oil	6.7% C16 fatty acid (6% saturated, 0.1% unsaturated)<br>92.51% C18 fatty acid (2.6% saturated, 89.91% unsaturated)

Composition analysis was carried out by the hydrolysis of 100g of each vegetable oil to corresponding carboxylic acids.

15. Analysis report of vegetable oils

Palm oil

**Probennummer:**	**2330013677**	Probeneingang:	17.02.2023
Probenart:	Pflanzliches Öl	Fettgehalt:	99 g/100g (laut Spezifikation)
Kennzeichnung:	Palm oil www.ktc-edibles.com		
Beginn der Prüfung:	20.02.2023	Ende der Prüfung:	23.02.2023

	C-Zahl	Stellung	Besonderheit	Fettsäure-Anteil in %	Fettsäure-Anteil in 100g Produkt in %	Fettsäure-Anteil in 100g Produkt als Triglycerid in %
Buttersäure	C4:0			< 0,1	< 0,09	< 0,10
Capronsäure	C6:0			< 0,1	< 0,09	< 0,10
Önanthsäure	C7:0			< 0,1	< 0,09	< 0,10
Caprylsäure	C8:0			< 0,1	< 0,09	< 0,10
Caprinsäure	C10:0			< 0,1	< 0,09	< 0,10
Undecansäure	C11:0			< 0,1	< 0,09	< 0,10
Laurinsäure	C12:0			0,2	0,14	0,15
Tridecansäure	C13:0			< 0,1	< 0,09	< 0,10
Myristinsäure	C14:0			0,8	0,70	0,74
Myristoleinsäure	C14:1	c-9		< 0,1	< 0,09	< 0,10
Myristelaidinsäure	C14:1	t-9	trans-Fettsäure	< 0,10	< 0,09	< 0,10
Pentadecansäure	C15:0			0,1	0,06	0,06
Pentadecensäure	C15:1	c-10		< 0,1	< 0,09	< 0,10
Palmitinsäure	C16:0			39,2	36,78	38,80
Palmitoleinsäure	C16:1	c-9		0,2	0,15	0,15
Palmitelaidinsäure	C16:1	t-9	trans-Fettsäure	< 0,10	< 0,09	< 0,10
Margarinsäure	C17:0			0,1	0,10	0,11
Heptadecensäure	C17:1	c-10		< 0,1	< 0,09	< 0,10
Stearinsäure	C18:0			5,4	5,08	5,33
Ölsäure	C18:1	c-9		41,6	39,26	41,20
Vaccensäure	C18:1	c-11		0,6	0,59	0,61
Elaidinsäure	C18:1	t-9	trans-Fettsäure	< 0,10	< 0,09	< 0,10
Summe trans-Ölsäure	C18:1	t-6,t-10,t-11	trans-Fettsäure	< 0,10	< 0,09	< 0,10
Linolsäure	C18:2	c-9,c-12	ω-6 Fettsäure	10,8	10,22	10,74
Linolsäure	C18:2	t-9,t-12	trans-Fettsäure	< 0,10	< 0,09	< 0,10
Linolsäure	C18:2	c-9,t-12	trans-Fettsäure	< 0,10	< 0,09	< 0,10
Linolsäure	C18:2	t-9,c-12	trans-Fettsäure	< 0,10	< 0,09	< 0,10
Linolsäure	C18:2	c-9, t-11 CLA	CLA	< 0,10	< 0,09	< 0,10
Linolsäure	C18:2	t-10, c-12 CLA	CLA	< 0,10	< 0,09	< 0,10
alpha-Linolensäure	C18:3	c-9,c-12,c-15	ω-3 Fettsäure	0,3	0,29	0,31
gamma-Linolensäure	C18:3	c-6,c-9,c-12	ω-6 Fettsäure	< 0,1	< 0,09	< 0,10
Arachinsäure	C20:0			0,4	0,39	0,41
Gondosäure	C20:1	c-11		0,2	0,16	0,17
Eicosadiensäure	C20:2	c-11, c-14	ω-6 Fettsäure	< 0,1	< 0,09	< 0,10
Eicosatriensäure	C20:3	c-8, c-11, c-14	ω-6 Fettsäure	< 0,1	< 0,09	< 0,10
Eicosatriensäure	C20:3	c-11,c-14,c-17	ω-3 Fettsäure	< 0,1	< 0,09	< 0,10
Arachidonsäure	C20:4	c-5,c-8,c-11,c-14	ω-6 Fettsäure	< 0,1	< 0,09	< 0,10
Eicosapentaensäure	C20:5	c-5,c-8,c-11,c-14,c-17	ω-3 Fettsäure	< 0,1	< 0,09	< 0,10
Hareicosansäure	C21:0			< 0,1	< 0,09	< 0,10
Behensäure	C22:0			0,1	0,08	0,08
Erucasäure	C22:1	c-13		< 0,1	< 0,10	< 0,10
Docosahexaensäure	C22:6	c-4,c-7,c-10,c-13,c-16,c-19	ω-3 Fettsäure	< 0,1	< 0,09	< 0,10
Tricosansäure	C23:0			< 0,1	< 0,10	< 0,10
Lignocerinsäure	C24:0			0,1	0,08	0,08
Nervonsäure	C24:1	c-15		< 0,1	< 0,10	< 0,10
**Fettsäuren gesättigte, Summe**				46,26	**43,44**	**45,80**
**Fettsäuren einfach ungesättigte, Summe**				42,56	**40,15**	**42,13**
**Fettsäuren mehrfach ungesättigte, Summe**				11,18	**10,54**	**11,07**
**Fettsäuren trans, Summe**				0,02	**0,02**	**0,02**
**Omega-3-Fettsäuren**				0,31	**0,29**	**0,31**
**Omega-6-Fettsäuren**				10,85	**10,22**	**10,74**
**Konjugierte Linolsäure-Isomere (CLA-Isomere), Summe**				0,02	**0,02**	**0,02**

Fettsäureverteilung in 100 % Fettsäuren

Sunflower oil

**Probennummer:**	**2330013684**	Probeneingang:	17.02.2023
Probenart:	Pflanzliches Öl	Fettgehalt:	99 g/100g (laut Spezifikation)
Kennzeichnung:	Sunflower oil www.kaufland.com		
Beginn der Prüfung:	20.02.2023	Ende der Prüfung:	23.02.2023

	C-Zahl	Stellung	Besonderheit	Fettsäure-Anteil in %	Fettsäure-Anteil in 100g Produkt in %	Fettsäure-Anteil in 100g Produkt als Triglycerid in %
Buttersäure	C4:0			< 0,1	< 0,09	< 0,10
Capronsäure	C6:0			< 0,1	< 0,09	< 0,10
Önanthsäure	C7:0			< 0,1	< 0,09	< 0,10
Caprylsäure	C8:0			< 0,1	< 0,09	< 0,10
Caprinsäure	C10:0			< 0,1	< 0,09	< 0,10
Undecansäure	C11:0			< 0,1	< 0,09	< 0,10
Laurinsäure	C12:0			< 0,1	< 0,09	< 0,10
Tridecansäure	C13:0			< 0,1	< 0,09	< 0,10
Myristinsäure	C14:0			0,1	0,12	0,13
Myristoleinsäure	C14:1	c-9		< 0,1	< 0,09	< 0,10
Myristelaidinsäure	C14:1	t-9	trans-Fettsäure	< 0,10	< 0,09	< 0,10
Pentadecansäure	C15:0			< 0,1	< 0,09	< 0,10
Pentadecensäure	C15:1	c-10		< 0,1	< 0,09	< 0,10
Palmitinsäure	C16:0			5,9	5,49	5,79
Palmitoleinsäure	C16:1	c-9		0,1	0,09	0,10
Palmitelaidinsäure	C16:1	t-9	trans-Fettsäure	< 0,10	< 0,09	< 0,10
Margarinsäure	C17:0			< 0,1	< 0,09	< 0,10
Heptadecensäure	C17:1	c-10		< 0,1	< 0,09	< 0,10
Stearinsäure	C18:0			3,1	2,88	3,02
Ölsäure	C18:1	c-9		28,0	26,46	27,76
Vaccensäure	C18:1	c-11		0,7	0,69	0,72
Elaidinsäure	C18:1	t-9	trans-Fettsäure	< 0,10	< 0,09	< 0,10
Summe trans-Ölsäure	C18:1	t-6,t-10,t-11	trans-Fettsäure	0,05	0,05	0,05
Linolsäure	C18:2	c-9,c-12	ω-6 Fettsäure	60,2	56,78	59,64
Linolsäure	C18:2	t-9,t-12	trans-Fettsäure	< 0,10	< 0,09	< 0,10
Linolsäure	C18:2	c-9,t-12	trans-Fettsäure	0,09	0,09	0,09
Linolsäure	C18:2	t-9,c-12	trans-Fettsäure	< 0,10	< 0,09	< 0,10
Linolsäure	C18:2	c-9, t-11 CLA	CLA	< 0,10	< 0,09	< 0,10
Linolsäure	C18:2	t-10, c-12 CLA	CLA	< 0,10	< 0,09	< 0,10
alpha-Linolensäure	C18:3	c-9,c-12,c-15	ω-3 Fettsäure	0,1	0,06	0,07
gamma-Linolensäure	C18:3	c-6,c-9,c-12	ω-6 Fettsäure	< 0,1	< 0,09	< 0,10
Arachinsäure	C20:0			0,2	0,21	0,22
Gondosäure	C20:1	c-11		0,2	0,16	0,16
Eicosadiensäure	C20:2	c-11, c-14	ω-6 Fettsäure	< 0,1	< 0,09	< 0,10
Eicosatriensäure	C20:3	c-8, c-11, c-14	ω-6 Fettsäure	< 0,1	< 0,09	< 0,10
Eicosatriensäure	C20:3	c-11,c-14,c-17	ω-3 Fettsäure	< 0,1	< 0,09	< 0,10
Arachidonsäure	C20:4	c-5,c-8,c-11,c-14	ω-6 Fettsäure	< 0,1	< 0,09	< 0,10
Eicosapentaensäure	C20:5	c-5,c-8,c-11,c-14,c-17	ω-3 Fettsäure	< 0,1	< 0,09	< 0,10
Hareicosansäure	C21:0			< 0,1	< 0,09	< 0,10
Behensäure	C22:0			0,8	0,76	0,80
Erucasäure	C22:1	c-13		< 0,1	< 0,10	< 0,10
Docosahexaensäure	C22:6	c-4,c-7,c-10,c-13,c-16,c-19	ω-3 Fettsäure	< 0,1	< 0,09	< 0,10
Tricosansäure	C23:0			< 0,1	< 0,10	< 0,10
Lignocerinsäure	C24:0			0,3	0,28	0,29
Nervonsäure	C24:1	c-15		< 0,1	< 0,10	< 0,10
**Fettsäuren gesättigte, Summe**				10,49	**9,87**	**10,38**
**Fettsäuren einfach ungesättigte, Summe**				29,09	**27,45**	**28,80**
**Fettsäuren mehrfach ungesättigte, Summe**				60,42	**56,94**	**59,82**
**Fettsäuren trans, Summe**				0,16	**0,16**	**0,16**
**Omega-3-Fettsäuren**				0,07	**0,06**	**0,07**
**Omega-6-Fettsäuren**				60,24	**56,78**	**59,64**
**Konjugierte Linolsäure-Isomere (CLA-Isomere), Summe**				0,02	**0,02**	**0,02**

Fettsäureverteilung in 100 % Fettsäuren

Rapeseed oil

**Probennummer:**	**2330013678**	Probeneingang:	17.02.2023
Probenart:	Pflanzliches Öl	Fettgehalt:	99 g/100g (laut Spezifikation)
Kennzeichnung:	Rapseed oil www.lidl.com		
Beginn der Prüfung:	20.02.2023	Ende der Prüfung:	23.02.2023

	C-Zahl	Stellung	Besonderheit	Fettsäure-Anteil in %	Fettsäure-Anteil in 100g Produkt in %	Fettsäure-Anteil in 100g Produkt als Triglycerid in %
Buttersäure	C4:0			< 0,1	< 0,09	< 0,10
Capronsäure	C6:0			< 0,1	< 0,09	< 0,10
Önanthsäure	C7:0			< 0,1	< 0,09	< 0,10
Caprylsäure	C8:0			0,1	0,06	0,06
Caprinsäure	C10:0			< 0,1	< 0,09	< 0,10
Undecansäure	C11:0			< 0,1	< 0,09	< 0,10
Laurinsäure	C12:0			< 0,1	< 0,09	< 0,10
Tridecansäure	C13:0			< 0,1	< 0,09	< 0,10
Myristinsäure	C14:0			0,1	0,13	0,14
Myristoleinsäure	C14:1	c-9		< 0,1	< 0,09	< 0,10
Myristelaidinsäure	C14:1	t-9	trans-Fettsäure	< 0,10	< 0,09	< 0,10
Pentadecansäure	C15:0			< 0,1	< 0,09	< 0,10
Pentadecensäure	C15:1	c-10		< 0,1	< 0,09	< 0,10
Palmitinsäure	C16:0			6,0	5,62	5,93
Palmitoleinsäure	C16:1	c-9		0,1	0,10	0,11
Palmitelaidinsäure	C16:1	t-9	trans-Fettsäure	< 0,10	< 0,09	< 0,10
Margarinsäure	C17:0			< 0,1	< 0,09	< 0,10
Heptadecensäure	C17:1	c-10		< 0,1	< 0,09	< 0,10
Stearinsäure	C18:0			3,1	2,91	3,05
Ölsäure	C18:1	c-9		29,9	28,19	29,58
Vaccensäure	C18:1	c-11		0,8	0,79	0,83
Elaidinsäure	C18:1	t-9	trans-Fettsäure	< 0,10	< 0,09	< 0,10
Summe trans-Ölsäure	C18:1	t-6,t-10,t-11	trans-Fettsäure	0,06	0,06	0,06
Linolsäure	C18:2	c-9,c-12	ω-6 Fettsäure	57,7	54,36	57,10
Linolsäure	C18:2	t-9,t-12	trans-Fettsäure	< 0,10	< 0,09	< 0,10
Linolsäure	C18:2	c-9,t-12	trans-Fettsäure	0,10	0,09	0,10
Linolsäure	C18:2	t-9,c-12	trans-Fettsäure	< 0,10	< 0,09	< 0,10
Linolsäure	C18:2	c-9, t-11 CLA	CLA	< 0,10	< 0,09	< 0,10
Linolsäure	C18:2	t-10, c-12 CLA	CLA	< 0,10	< 0,09	< 0,10
alpha-Linolensäure	C18:3	c-9,c-12,c-15	ω-3 Fettsäure	0,4	0,34	0,36
gamma-Linolensäure	C18:3	c-6,c-9,c-12	ω-6 Fettsäure	< 0,1	< 0,09	< 0,10
Arachinsäure	C20:0			0,3	0,25	0,26
Gondosäure	C20:1	c-11		0,2	0,21	0,22
Eicosadiensäure	C20:2	c-11, c-14	ω-6 Fettsäure	< 0,1	< 0,09	< 0,10
Eicosatriensäure	C20:3	c-8, c-11, c-14	ω-6 Fettsäure	< 0,1	< 0,09	< 0,10
Eicosatriensäure	C20:3	c-11,c-14,c-17	ω-3 Fettsäure	< 0,1	< 0,09	< 0,10
Arachidonsäure	C20:4	c-5,c-8,c-11,c-14	ω-6 Fettsäure	< 0,1	< 0,09	< 0,10
Eicosapentaensäure	C20:5	c-5,c-8,c-11,c-14,c-17	ω-3 Fettsäure	< 0,1	< 0,09	< 0,10
Hareicosansäure	C21:0			< 0,1	< 0,09	< 0,10
Behensäure	C22:0			0,8	0,74	0,77
Erucasäure	C22:1	c-13		< 0,1	< 0,10	< 0,10
Docosahexaensäure	C22:6	c-4,c-7,c-10,c-13,c-16,c-19	ω-3 Fettsäure	< 0,1	< 0,09	< 0,10
Tricosansäure	C23:0			< 0,1	< 0,10	< 0,10
Lignocerinsäure	C24:0			0,3	0,27	0,28
Nervonsäure	C24:1	c-15		< 0,1	< 0,10	< 0,10
**Fettsäuren gesättigte, Summe**				10,72	**10,09**	**10,61**
**Fettsäuren einfach ungesättigte, Summe**				31,13	**29,37**	**30,82**
**Fettsäuren mehrfach ungesättigte, Summe**				58,15	**54,81**	**57,57**
**Fettsäuren trans, Summe**				0,20	**0,19**	**0,20**
**Omega-3-Fettsäuren**				0,36	**0,34**	**0,36**
**Omega-6-Fettsäuren**				57,67	**54,36**	**57,10**
**Konjugierte Linolsäure-Isomere (CLA-Isomere), Summe**				0,02	**0,02**	**0,02**

Fettsäureverteilung in 100 % Fettsäuren

Olive oil

Probennummer:	2330013683	Probeneingang:	17.02.2023
Probenart:	Pflanzliches Öl	Fettgehalt:	99 g/100g (laut Spezifikation)
Kennzeichnung:	Olive oil www.laespanolaoliveoil.de		
Beginn der Prüfung:	20.02.2023	Ende der Prüfung:	23.02.2023

	C-Zahl	Stellung	Besonderheit	Fettsäure-Anteil in %	Fettsäure-Anteil in 100g Produkt in %	Fettsäure-Anteil in 100g Produkt als Triglycerid in %
Buttersäure	C4:0			< 0,1	< 0,09	< 0,10
Capronsäure	C6:0			< 0,1	< 0,09	< 0,10
Önanthsäure	C7:0			< 0,1	< 0,09	< 0,10
Caprylsäure	C8:0			< 0,1	< 0,09	< 0,10
Caprinsäure	C10:0			< 0,1	< 0,09	< 0,10
Undecansäure	C11:0			< 0,1	< 0,09	< 0,10
Laurinsäure	C12:0			< 0,1	< 0,09	< 0,10
Tridecansäure	C13:0			< 0,1	< 0,09	< 0,10
Myristinsäure	C14:0			0,1	0,05	0,06
Myristoleinsäure	C14:1	c-9		< 0,1	< 0,09	< 0,10
Myristelaidinsäure	C14:1	t-9	trans-Fettsäure	< 0,10	< 0,09	< 0,10
Pentadecansäure	C15:0			< 0,1	< 0,09	< 0,10
Pentadecensäure	C15:1	c-10		< 0,1	< 0,09	< 0,10
Palmitinsäure	C16:0			14,3	13,40	14,13
Palmitoleinsäure	C16:1	c-9		0,8	0,80	0,84
Palmitelaidinsäure	C16:1	t-9	trans-Fettsäure	< 0,10	< 0,09	< 0,10
Margarinsäure	C17:0			0,2	0,16	0,17
Heptadecensäure	C17:1	c-10		< 0,1	< 0,09	< 0,10
Stearinsäure	C18:0			3,4	3,19	3,34
Ölsäure	C18:1	c-9		66,4	62,63	65,72
Vaccensäure	C18:1	c-11		2,1	1,95	2,05
Elaidinsäure	C18:1	t-9	trans-Fettsäure	< 0,10	< 0,09	< 0,10
Summe trans-Ölsäure	C18:1	t-6,t-10,t-11	trans-Fettsäure	< 0,10	< 0,09	< 0,10
Linolsäure	C18:2	c-9,c-12	ω-6 Fettsäure	10,8	10,14	10,65
Linolsäure	C18:2	t-9,t-12	trans-Fettsäure	< 0,10	< 0,09	< 0,10
Linolsäure	C18:2	c-9,t-12	trans-Fettsäure	< 0,10	< 0,09	< 0,10
Linolsäure	C18:2	t-9,c-12	trans-Fettsäure	< 0,10	< 0,09	< 0,10
Linolsäure	C18:2	c-9, t-11 CLA	CLA	< 0,10	< 0,09	< 0,10
Linolsäure	C18:2	t-10, c-12 CLA	CLA	< 0,10	< 0,09	< 0,10
alpha-Linolensäure	C18:3	c-9,c-12,c-15	ω-3 Fettsäure	0,7	0,65	0,68
gamma-Linolensäure	C18:3	c-6,c-9,c-12	ω-6 Fettsäure	< 0,1	< 0,09	< 0,10
Arachinsäure	C20:0			0,6	0,56	0,58
Gondosäure	C20:1	c-11		0,3	0,31	0,32
Eicosadiensäure	C20:2	c-11, c-14	ω-6 Fettsäure	< 0,1	< 0,09	< 0,10
Eicosatriensäure	C20:3	c-8, c-11, c-14	ω-6 Fettsäure	< 0,1	< 0,09	< 0,10
Eicosatriensäure	C20:3	c-11,c-14,c-17	ω-3 Fettsäure	< 0,1	< 0,09	< 0,10
Arachidonsäure	C20:4	c-5,c-8,c-11,c-14	ω-6 Fettsäure	< 0,1	< 0,09	< 0,10
Eicosapentaensäure	C20:5	c-5,c-8,c-11,c-14,c-17	ω-3 Fettsäure	< 0,1	< 0,09	< 0,10
Hareicosansäure	C21:0			< 0,1	< 0,09	< 0,10
Behensäure	C22:0			0,2	0,18	0,18
Erucasäure	C22:1	c-13		< 0,1	< 0,10	< 0,10
Docosahexaensäure	C22:6	c-4,c-7,c-10,c-13,c-16,c-19	ω-3 Fettsäure	< 0,1	< 0,09	< 0,10
Tricosansäure	C23:0			< 0,1	< 0,10	< 0,10
Lignocerinsäure	C24:0			0,1	0,12	0,12
Nervonsäure	C24:1	c-15		< 0,1	< 0,10	< 0,10
**Fettsäuren gesättigte, Summe**				18,87	**17,74**	**18,68**
**Fettsäuren einfach ungesättigte, Summe**				69,66	**65,72**	**68,96**
**Fettsäuren mehrfach ungesättigte, Summe**				11,47	**10,81**	**11,36**
**Fettsäuren trans, Summe**				0,06	**0,06**	**0,06**
**Omega-3-Fettsäuren**				0,69	**0,65**	**0,68**
**Omega-6-Fettsäuren**				10,76	**10,14**	**10,65**
**Konjugierte Linolsäure-Isomere (CLA-Isomere), Summe**				0,02	**0,01**	**0,02**

Fettsäureverteilung in 100 % Fettsäuren

Coconut oil

**Probennummer:**	**2330013681**	Probeneingang:	17.02.2023
Probenart:	Pflanzliches Öl	Fettgehalt:	99 g/100g (laut Spezifikation)
Kennzeichnung:	Coconut oil www.kaufland.com		
Beginn der Prüfung:	20.02.2023	Ende der Prüfung:	23.02.2023

	C-Zahl	Stellung	Besonderheit	Fettsäure-Anteil in %	Fettsäure-Anteil in 100g Produkt in %	Fettsäure-Anteil in 100g Produkt als Triglycerid in %
Buttersäure	C4:0			< 0,1	< 0,09	< 0,10
Capronsäure	C6:0			0,8	0,73	0,82
Önanthsäure	C7:0			< 0,1	< 0,09	< 0,10
Caprylsäure	C8:0			9,2	8,32	9,13
Caprinsäure	C10:0			6,0	5,49	5,94
Undecansäure	C11:0			< 0,1	< 0,09	< 0,10
Laurinsäure	C12:0			47,7	44,11	47,18
Tridecansäure	C13:0			< 0,1	< 0,09	< 0,10
Myristinsäure	C14:0			19,4	18,08	19,19
Myristoleinsäure	C14:1	c-9		< 0,1	< 0,09	< 0,10
Myristelaidinsäure	C14:1	t-9	trans-Fettsäure	< 0,10	< 0,09	< 0,10
Pentadecansäure	C15:0			< 0,1	< 0,09	< 0,10
Pentadecensäure	C15:1	c-10		< 0,1	< 0,09	< 0,10
Palmitinsäure	C16:0			7,7	7,22	7,61
Palmitoleinsäure	C16:1	c-9		< 0,1	< 0,09	< 0,10
Palmitelaidinsäure	C16:1	t-9	trans-Fettsäure	< 0,10	< 0,09	< 0,10
Margarinsäure	C17:0			< 0,1	< 0,09	< 0,10
Heptadecensäure	C17:1	c-10		< 0,1	< 0,09	< 0,10
Stearinsäure	C18:0			3,1	2,94	3,08
Ölsäure	C18:1	c-9		4,9	4,65	4,88
Vaccensäure	C18:1	c-11		< 0,1	< 0,09	< 0,10
Elaidinsäure	C18:1	t-9	trans-Fettsäure	< 0,10	< 0,09	< 0,10
Summe trans-Ölsäure	C18:1	t-6,t-10,t-11	trans-Fettsäure	< 0,10	< 0,09	< 0,10
Linolsäure	C18:2	c-9,c-12	ω-6 Fettsäure	0,9	0,81	0,85
Linolsäure	C18:2	t-9,t-12	trans-Fettsäure	< 0,10	< 0,09	< 0,10
Linolsäure	C18:2	c-9,t-12	trans-Fettsäure	< 0,10	< 0,09	< 0,10
Linolsäure	C18:2	t-9,c-12	trans-Fettsäure	< 0,10	< 0,09	< 0,10
Linolsäure	C18:2	c-9, t-11 CLA	CLA	< 0,10	< 0,09	< 0,10
Linolsäure	C18:2	t-10, c-12 CLA	CLA	< 0,10	< 0,09	< 0,10
alpha-Linolensäure	C18:3	c-9,c-12,c-15	ω-3 Fettsäure	< 0,1	< 0,09	< 0,10
gamma-Linolensäure	C18:3	c-6,c-9,c-12	ω-6 Fettsäure	< 0,1	< 0,09	< 0,10
Arachinsäure	C20:0			0,1	0,08	0,08
Gondosäure	C20:1	c-11		< 0,1	< 0,09	< 0,10
Eicosadiensäure	C20:2	c-11, c-14	ω-6 Fettsäure	< 0,1	< 0,09	< 0,10
Eicosatriensäure	C20:3	c-8, c-11, c-14	ω-6 Fettsäure	< 0,1	< 0,09	< 0,10
Eicosatriensäure	C20:3	c-11,c-14,c-17	ω-3 Fettsäure	< 0,1	< 0,09	< 0,10
Arachidonsäure	C20:4	c-5,c-8,c-11,c-14	ω-6 Fettsäure	< 0,1	< 0,09	< 0,10
Eicosapentaensäure	C20:5	c-5,c-8,c-11,c-14,c-17	ω-3 Fettsäure	< 0,1	< 0,09	< 0,10
Hareicosansäure	C21:0			< 0,1	< 0,09	< 0,10
Behensäure	C22:0			< 0,1	< 0,10	< 0,10
Erucasäure	C22:1	c-13		< 0,1	< 0,10	< 0,10
Docosahexaensäure	C22:6	c-4,c-7,c-10,c-13,c-16,c-19	ω-3 Fettsäure	< 0,1	< 0,09	< 0,10
Tricosansäure	C23:0			< 0,1	< 0,10	< 0,10
Lignocerinsäure	C24:0			< 0,1	< 0,10	< 0,10
Nervonsäure	C24:1	c-15		< 0,1	< 0,10	< 0,10
**Fettsäuren gesättigte, Summe**				94,11	**87,10**	**93,17**
**Fettsäuren einfach ungesättigte, Summe**				5,02	**4,74**	**4,97**
**Fettsäuren mehrfach ungesättigte, Summe**				0,86	**0,81**	**0,85**
**Fettsäuren trans, Summe**				0,00	**0,00**	**0,00**
**Omega-3-Fettsäuren**				0,00	**0,00**	**0,00**
**Omega-6-Fettsäuren**				0,86	**0,81**	**0,85**
**Konjugierte Linolsäure-Isomere (CLA-Isomere), Summe**				0,00	**0,00**	**0,00**

Fettsäureverteilung in 100 % Fettsäuren

Pumpkin oil

**Probennummer:**	**2330013680**	Probeneingang:	17.02.2023
Probenart:	Pflanzliches Öl	Fettgehalt:	99 g/100g (laut Spezifikation)
Kennzeichnung:	Pumpkin oil www.kunella-feinkost.com		
Beginn der Prüfung:	20.02.2023	Ende der Prüfung:	23.02.2023

	C-Zahl	Stellung	Besonderheit	Fettsäure-Anteil in %	Fettsäure-Anteil in 100g Produkt in %	Fettsäure-Anteil in 100g Produkt als Triglycerid in %
Buttersäure	C4:0			< 0,1	< 0,09	< 0,10
Capronsäure	C6:0			< 0,1	< 0,09	< 0,10
Önanthsäure	C7:0			< 0,1	< 0,09	< 0,10
Caprylsäure	C8:0			< 0,1	< 0,09	< 0,10
Caprinsäure	C10:0			< 0,1	< 0,09	< 0,10
Undecansäure	C11:0			< 0,1	< 0,09	< 0,10
Laurinsäure	C12:0			< 0,1	< 0,09	< 0,10
Tridecansäure	C13:0			< 0,1	< 0,09	< 0,10
Myristinsäure	C14:0			0,1	0,13	0,14
Myristoleinsäure	C14:1	c-9		< 0,1	< 0,09	< 0,10
Myristelaidinsäure	C14:1	t-9	trans-Fettsäure	< 0,10	< 0,09	< 0,10
Pentadecansäure	C15:0			< 0,1	< 0,09	< 0,10
Pentadecensäure	C15:1	c-10		< 0,1	< 0,09	< 0,10
Palmitinsäure	C16:0			11,1	10,46	11,03
Palmitoleinsäure	C16:1	c-9		0,1	0,12	0,12
Palmitelaidinsäure	C16:1	t-9	trans-Fettsäure	< 0,10	< 0,09	< 0,10
Margarinsäure	C17:0			0,1	0,06	0,06
Heptadecensäure	C17:1	c-10		< 0,1	< 0,09	< 0,10
Stearinsäure	C18:0			5,5	5,19	5,45
Ölsäure	C18:1	c-9		28,8	27,15	28,49
Vaccensäure	C18:1	c-11		0,8	0,72	0,75
Elaidinsäure	C18:1	t-9	trans-Fettsäure	< 0,10	< 0,09	< 0,10
Summe trans-Ölsäure	C18:1	t-6,t-10,t-11	trans-Fettsäure	< 0,10	< 0,09	< 0,10
Linolsäure	C18:2	c-9,c-12	ω-6 Fettsäure	52,5	49,47	51,96
Linolsäure	C18:2	t-9,t-12	trans-Fettsäure	< 0,10	< 0,09	< 0,10
Linolsäure	C18:2	c-9,t-12	trans-Fettsäure	0,06	0,06	0,06
Linolsäure	C18:2	t-9,c-12	trans-Fettsäure	< 0,10	< 0,09	< 0,10
Linolsäure	C18:2	c-9, t-11 CLA	CLA	< 0,10	< 0,09	< 0,10
Linolsäure	C18:2	t-10, c-12 CLA	CLA	< 0,10	< 0,09	< 0,10
alpha-Linolensäure	C18:3	c-9,c-12,c-15	ω-3 Fettsäure	0,2	0,17	0,17
gamma-Linolensäure	C18:3	c-6,c-9,c-12	ω-6 Fettsäure	< 0,1	< 0,09	< 0,10
Arachinsäure	C20:0			0,4	0,34	0,36
Gondosäure	C20:1	c-11		0,1	0,09	0,10
Eicosadiensäure	C20:2	c-11, c-14	ω-6 Fettsäure	< 0,1	< 0,09	< 0,10
Eicosatriensäure	C20:3	c-8, c-11, c-14	ω-6 Fettsäure	< 0,1	< 0,09	< 0,10
Eicosatriensäure	C20:3	c-11,c-14,c-17	ω-3 Fettsäure	< 0,1	< 0,09	< 0,10
Arachidonsäure	C20:4	c-5,c-8,c-11,c-14	ω-6 Fettsäure	< 0,1	< 0,09	< 0,10
Eicosapentaensäure	C20:5	c-5,c-8,c-11,c-14,c-17	ω-3 Fettsäure	< 0,1	< 0,09	< 0,10
Hareicosansäure	C21:0			< 0,1	< 0,09	< 0,10
Behensäure	C22:0			0,1	0,13	0,14
Erucasäure	C22:1	c-13		< 0,1	< 0,10	< 0,10
Docosahexaensäure	C22:6	c-4,c-7,c-10,c-13,c-16,c-19	ω-3 Fettsäure	< 0,1	< 0,09	< 0,10
Tricosansäure	C23:0			< 0,1	< 0,10	< 0,10
Lignocerinsäure	C24:0			0,1	0,07	0,08
Nervonsäure	C24:1	c-15		< 0,1	< 0,10	< 0,10
**Fettsäuren gesättigte, Summe**				17,48	**16,43**	**17,30**
**Fettsäuren einfach ungesättigte, Summe**				29,80	**28,12**	**29,51**
**Fettsäuren mehrfach ungesättigte, Summe**				52,72	**49,69**	**52,19**
**Fettsäuren trans, Summe**				0,10	**0,09**	**0,10**
**Omega-3-Fettsäuren**				0,18	**0,17**	**0,17**
**Omega-6-Fettsäuren**				52,49	**49,47**	**51,96**
**Konjugierte Linolsäure-Isomere (CLA-Isomere), Summe**				0,00	**0,00**	**0,00**

Fettsäureverteilung in 100 % Fettsäuren

Walnut oil

**Probennummer:**	**2330013679**	Probeneingang:	17.02.2023
Probenart:	Pflanzliches Öl	Fettgehalt:	99 g/100g (laut Spezifikation)
Kennzeichnung:	Walnut oil www.kunella-feinkost.com		
Beginn der Prüfung:	20.02.2023	Ende der Prüfung:	23.02.2023

	C-Zahl	Stellung	Besonderheit	Fettsäure-Anteil in %	Fettsäure-Anteil in 100g Produkt in %	Fettsäure-Anteil in 100g Produkt als Triglycerid in %
Buttersäure	C4:0			< 0,1	< 0,09	< 0,10
Capronsäure	C6:0			< 0,1	< 0,09	< 0,10
Önanthsäure	C7:0			< 0,1	< 0,09	< 0,10
Caprylsäure	C8:0			< 0,1	< 0,09	< 0,10
Caprinsäure	C10:0			< 0,1	< 0,09	< 0,10
Undecansäure	C11:0			< 0,1	< 0,09	< 0,10
Laurinsäure	C12:0			< 0,1	< 0,09	< 0,10
Tridecansäure	C13:0			< 0,1	< 0,09	< 0,10
Myristinsäure	C14:0			0,1	0,06	0,07
Myristoleinsäure	C14:1	c-9		< 0,1	< 0,09	< 0,10
Myristelaidinsäure	C14:1	t-9	trans-Fettsäure	< 0,10	< 0,09	< 0,10
Pentadecansäure	C15:0			< 0,1	< 0,09	< 0,10
Pentadecensäure	C15:1	c-10		< 0,1	< 0,09	< 0,10
Palmitinsäure	C16:0			6,6	6,22	6,56
Palmitoleinsäure	C16:1	c-9		0,1	0,09	0,10
Palmitelaidinsäure	C16:1	t-9	trans-Fettsäure	< 0,10	< 0,09	< 0,10
Margarinsäure	C17:0			0,1	0,05	0,06
Heptadecensäure	C17:1	c-10		< 0,1	< 0,09	< 0,10
Stearinsäure	C18:0			2,6	2,41	2,53
Ölsäure	C18:1	c-9		16,1	15,23	15,98
Vaccensäure	C18:1	c-11		0,9	0,84	0,88
Elaidinsäure	C18:1	t-9	trans-Fettsäure	< 0,10	< 0,09	< 0,10
Summe trans-Ölsäure	C18:1	t-6,t-10,t-11	trans-Fettsäure	0,07	0,07	0,07
Linolsäure	C18:2	c-9,c-12	ω-6 Fettsäure	61,4	57,91	60,83
Linolsäure	C18:2	t-9,t-12	trans-Fettsäure	< 0,10	< 0,09	< 0,10
Linolsäure	C18:2	c-9,t-12	trans-Fettsäure	0,31	0,30	0,31
Linolsäure	C18:2	t-9,c-12	trans-Fettsäure	< 0,10	< 0,09	< 0,10
Linolsäure	C18:2	c-9, t-11 CLA	CLA	< 0,10	< 0,09	< 0,10
Linolsäure	C18:2	t-10, c-12 CLA	CLA	< 0,10	< 0,09	< 0,10
alpha-Linolensäure	C18:3	c-9,c-12,c-15	ω-3 Fettsäure	11,2	10,54	11,07
gamma-Linolensäure	C18:3	c-6,c-9,c-12	ω-6 Fettsäure	< 0,1	< 0,09	< 0,10
Arachinsäure	C20:0			0,1	0,08	0,08
Gondosäure	C20:1	c-11		0,2	0,23	0,24
Eicosadiensäure	C20:2	c-11, c-14	ω-6 Fettsäure	< 0,1	< 0,09	< 0,10
Eicosatriensäure	C20:3	c-8, c-11, c-14	ω-6 Fettsäure	< 0,1	< 0,09	< 0,10
Eicosatriensäure	C20:3	c-11,c-14,c-17	ω-3 Fettsäure	< 0,1	< 0,09	< 0,10
Arachidonsäure	C20:4	c-5,c-8,c-11,c-14	ω-6 Fettsäure	< 0,1	< 0,09	< 0,10
Eicosapentaensäure	C20:5	c-5,c-8,c-11,c-14,c-17	ω-3 Fettsäure	< 0,1	< 0,09	< 0,10
Hareicosansäure	C21:0			< 0,1	< 0,09	< 0,10
Behensäure	C22:0			0,1	0,07	0,07
Erucasäure	C22:1	c-13		< 0,1	< 0,10	< 0,10
Docosahexaensäure	C22:6	c-4,c-7,c-10,c-13,c-16,c-19	ω-3 Fettsäure	< 0,1	< 0,09	< 0,10
Tricosansäure	C23:0			< 0,1	< 0,10	< 0,10
Lignocerinsäure	C24:0			< 0,1	< 0,10	< 0,10
Nervonsäure	C24:1	c-15		< 0,1	< 0,10	< 0,10
**Fettsäuren gesättigte, Summe**				9,56	**8,98**	**9,46**
**Fettsäuren einfach ungesättigte, Summe**				17,44	**16,46**	**17,27**
**Fettsäuren mehrfach ungesättigte, Summe**				73,00	**68,80**	**72,27**
**Fettsäuren trans, Summe**				0,41	**0,38**	**0,40**
**Omega-3-Fettsäuren**				11,19	**10,54**	**11,07**
**Omega-6-Fettsäuren**				61,48	**57,95**	**60,87**
**Konjugierte Linolsäure-Isomere (CLA-Isomere), Summe**				0,02	**0,02**	**0,02**

Fettsäureverteilung in 100 % Fettsäuren

16. NMR data

Note: In case of some products, peaks of secondary N-H are not properly observed in the corresponding

[1,1'-biphenyl]-4-ylmethanamine

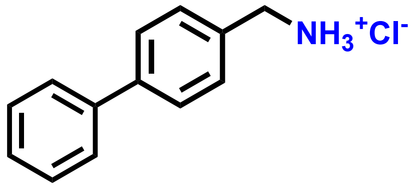

^{1}H NMR (300 MHz, DMSO) δ 8.62 (bs, 3H), 7.75 – 7.56 (m, 6H), 7.52 – 7.43 (m, 2H), 7.42 – 7.33 (m, 1H), 4.14 – 3.94 (m, 2H).

^{13}C NMR (75 MHz, DMSO) δ 140.19, 139.54, 133.30, 129.63, 129.00, 127.69, 126.77, 126.71, 41.81.

(4-(tert-butyl)phenyl)methanamine hydrochloride

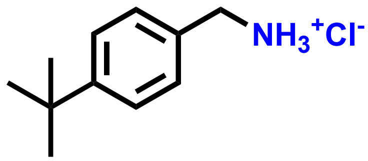

^{1}H NMR (400 MHz, DMSO) δ 8.55 (bs, 3H), 7.48 – 7.36 (m, 4H), 3.94 (q, J = 5.8 Hz, 2H), 1.27 (s, 9H).

^{13}C NMR (101 MHz, DMSO) δ 150.93, 131.12, 128.82, 125.29, 41.85, 34.34, 31.09.

Naphthalen-1-ylmethanamine hydrochloride

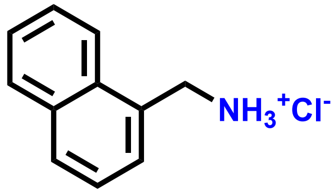

^{1}H NMR (300 MHz, DMSO) δ 8.84 (bs, 3H), 8.15 (dt, J = 8.2, 1.6 Hz, 1H), 8.06 – 7.89 (m, 2H), 7.76 – 7.48 (m, 4H), 4.52 (q, J = 5.6 Hz, 2H).

^{13}C NMR (75 MHz, DMSO) δ 133.23, 130.69, 129.99, 129.01, 128.66, 127.31, 126.77, 126.24, 125.39, 123.52, 39.12.

(4-fluorophenyl)methanamine hydrochloride

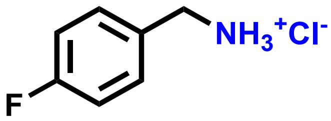

^{1}H NMR (300 MHz, DMSO) δ 8.55 (bs, 3H), 7.62 – 7.50 (m, 2H), 7.24 (td, *J* = 9.0, 0.9 Hz, 2H), 3.99 (q, *J* = 5.8 Hz, 2H).

^{13}C NMR (75 MHz, DMSO) δ 162.09 (d, *J* = 244.5 Hz), 131.40 (d, *J* = 8.4 Hz), 130.43 (d, *J* = 3.1 Hz), 115.37 (d, *J* = 21.5 Hz), 41.37.

(2-chlorophenyl)methanamine hydrochloride

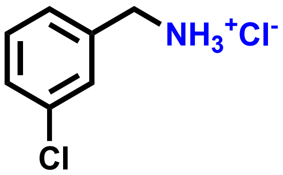

^{1}H NMR (300 MHz, DMSO) δ 8.67 (bs, 3H), 7.65 (dd, *J* = 1.7, 1.0 Hz, 1H), 7.55 – 7.35 (m, 3H), 4.02 (q, *J* = 5.5 Hz, 2H).

^{13}C NMR (75 MHz, DMSO) δ 136.58, 133.04, 130.41, 128.94, 128.29, 127.78, 41.45.

(3-methoxyphenyl)methanamine hydrochloride

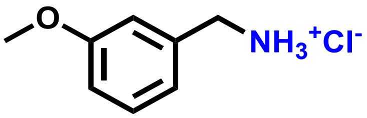

^{1}H NMR (300 MHz, DMSO) δ 8.68 (bs, 3H), 7.29 (t, *J* = 7.9 Hz, 1H), 7.24 – 7.13 (m, 1H), 7.06 (d, *J* = 7.7 Hz, 1H), 6.91 (dd, *J* = 8.1, 2.8 Hz, 1H), 4.09 – 3.86 (m, 2H), 3.76 (s, 3H).

^{13}C NMR (75 MHz, DMSO) δ 159.32, 135.61, 129.65, 121.01, 114.54, 113.96, 55.24, 42.08.

(6-methoxynaphthalen-2-yl)methanamine hydrochloride

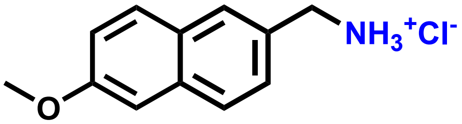

^{1}H NMR (300 MHz, DMSO) δ 8.58 (bs, 3H), 7.96 – 7.76 (m, 3H), 7.60 (dd, *J* = 8.4, 1.8 Hz, 1H), 7.35 (d, *J* = 2.7 Hz, 1H), 7.20 (dd, *J* = 8.9, 2.6 Hz, 1H), 4.12 (q, *J* = 5.7 Hz, 2H), 3.87 (s, 3H).

^{13}C NMR (75 MHz, DMSO) δ 157.69, 134.07, 129.34, 129.18, 128.02, 127.97, 127.16, 127.12, 119.19, 105.91, 55.28, 42.34.

(3-(benzyloxy)phenyl)methanamine hydrochloride

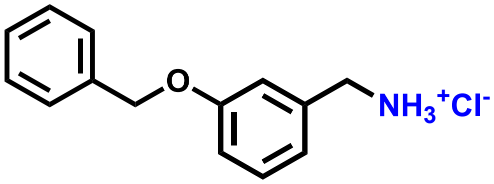

^{1}H NMR (300 MHz, DMSO) δ 8.70 (s, 3H), 7.50 – 7.23 (m, 7H), 7.14 – 6.95 (m, 2H), 5.11 (s, 2H), 3.97 (s, 2H).

^{13}C NMR (75 MHz, DMSO) δ 158.40, 136.93, 135.70, 129.68, 128.49, 127.93, 127.79, 121.24, 115.56, 114.57, 69.30, 42.07.

(4-(trifluoromethoxy)phenyl)methanamine hydrochloride

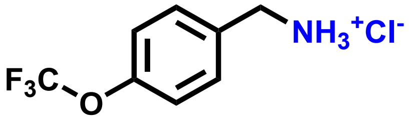

^{1}H NMR (300 MHz, DMSO) δ 8.71 (bs, 3H), 7.68 (d, *J* = 8.7 Hz, 2H), 7.40 (dd, *J* = 8.8, 1.0 Hz, 2H), 4.04 (q, *J* = 5.7 Hz, 2H).

^{13}C NMR (75 MHz, DMSO) δ 148.27 (q, *J* = 1.8 Hz), 133.70, 131.26, 121.11, 120.08 (q, *J* = 256.5 Hz), 41.32.

(4-(aminomethyl)phenyl)methanol hydrochloride

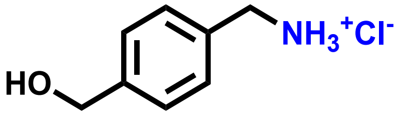

^{1}H NMR (300 MHz, DMSO) δ 8.55 (bs, 3H), 7.44 (d, *J* = 8.3 Hz, 2H), 7.33 (d, *J* = 8.3 Hz, 2H), 4.49 (s, 2H), 4.03 – 3.98 (m, 2H).

^{13}C NMR (75 MHz, DMSO) δ 142.86, 132.30, 128.74, 126.48, 62.46, 41.93.

(4-(methylthio)phenyl)methanamine hydrochloride

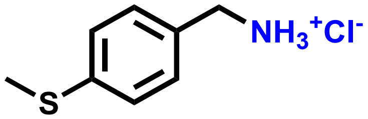

^{1}H NMR (300 MHz, DMSO) δ 8.62 (bs, 3H), 7.46 (d, *J* = 8.6 Hz, 2H), 7.27 (d, *J* = 8.6 Hz, 2H), 3.94 (s, 2H), 2.46 (s, 3H).

^{13}C NMR (75 MHz, DMSO) δ 138.57, 130.54, 129.72, 125.79, 41.65, 14.65.

(3-chloro-4-methylphenyl)methanamine hydrochloride

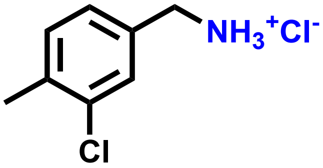

^{1}H NMR (400 MHz, DMSO) δ 8.57 (s, 3H), 7.61 (t, *J* = 1.0 Hz, 1H), 7.38 (d, *J* = 1.1 Hz, 2H), 3.97 (d, *J* = 5.5 Hz, 2H), 2.32 (s, 3H).

^{13}C NMR (101 MHz, DMSO) δ 135.54, 133.76, 133.14, 131.25, 129.36, 127.84, 41.20, 19.29.

(3,4-dichlorophenyl)methanamine hydrochloride

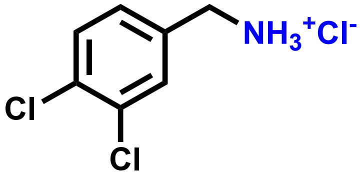

^{1}H NMR (300 MHz, DMSO) δ 8.73 (bs, 3H), 7.87 (d, *J* = 2.0 Hz, 1H), 7.67 (d, *J* = 8.3 Hz, 1H), 7.53 (dd, *J* = 8.3, 2.1 Hz, 1H), 4.03 (q, *J* = 5.6 Hz, 2H).

^{13}C NMR (75 MHz, DMSO) δ 135.24, 131.27, 131.05, 130.98, 130.64, 129.58, 40.89.

(4-(benzyloxy)-3-methoxyphenyl)methanamine hydrochloride

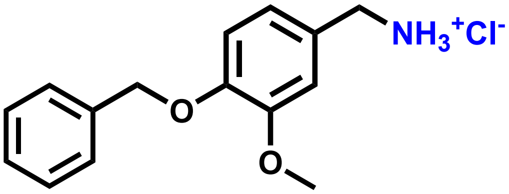

^{1}H NMR (300 MHz, DMSO) δ 8.54 (bs, 3H), 7.42 – 7.27 (m, 6H), 7.07 – 6.91 (m, 2H), 5.08 (s, 2H), 3.89 (t, *J* = 5.6 Hz, 2H), 3.78 (s, 3H).

^{13}C NMR (75 MHz, DMSO) δ 148.78, 147.49, 136.85, 128.22, 127.64, 127.51, 126.55, 121.16, 113.14, 113.08, 69.61, 55.50, 41.82.

(3,5-bis(trifluoromethyl)phenyl)methanamine hydrochloride

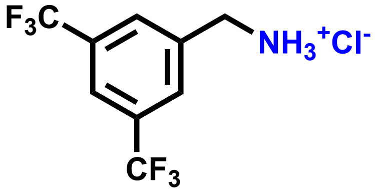

^{1}H NMR (300 MHz, DMSO) δ 8.80 (bs, 3H), 8.37 – 8.30 (m, 2H), 8.10 (tt, *J* = 1.7, 0.8 Hz, 1H), 4.25 (s, 2H).

^{13}C NMR (75 MHz, DMSO) δ 137.54, 130.43, 130.22 (q, *J* = 36.0 Hz), 123.28 (q, *J* = 272.7 Hz), 122.05 (q, *J* = 3.8 Hz), 41.10.

__pyridin-3-ylmethanamine hydrochloride__

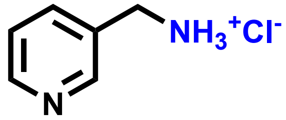

^{1}H NMR (300 MHz, D_2O) δ 9.00 (d, *J* = 2.3 Hz, 1H), 8.90 (d, *J* = 5.9 Hz, 1H), 8.78 (dt, *J* = 8.3, 1.7 Hz, 1H), 8.20 (dd, *J* = 8.3, 5.7 Hz, 1H), 4.53 (s, 2H).

^{13}C NMR (75 MHz, D_2O) δ 147.76, 141.94, 141.71, 133.01, 127.96, 39.68.

__(6-(trifluoromethyl)pyridin-3-yl)methanamine hydrochloride__

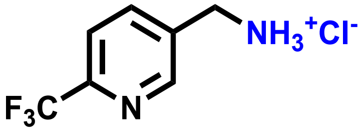

^{1}H NMR (300 MHz, DMSO) δ 8.90 (d, *J* = 2.5 Hz, 1H), 8.82 (s, 3H), 8.29 (dd, *J* = 8.2, 2.4 Hz, 1H), 7.98 (d, *J* = 8.2 Hz, 1H), 4.19 (q, *J* = 5.3 Hz, 2H).

^{13}C NMR (75 MHz, DMSO) δ 150.83, 146.17 (q, *J* = 34.0 Hz), 139.13, 133.94, 121.59 (q, *J* = 272.3 Hz), 120.44 (q, *J* = 2.8 Hz), 39.28.

__Benzo[d][1,3]dioxol-5-ylmethanamine hydrochloride__

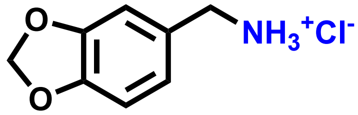

^{1}H NMR (300 MHz, DMSO) δ 8.46 (bs, 3H), 7.13 (d, *J* = 1.7 Hz, 1H), 7.04 – 6.85 (m, 2H), 6.03 (s, 2H), 3.90 (s, 2H).

^{13}C NMR (75 MHz, DMSO) δ 147.29, 147.26, 127.70, 122.89, 109.50, 108.24, 101.20, 41.95.

(2,3-dihydrobenzo[b][1,4]dioxin-6-yl)methanamine hydrochloride

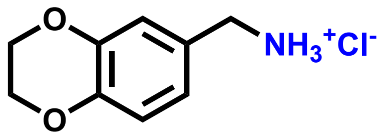

^{1}H NMR (400 MHz, DMSO) δ 8.55 (bs, 3H), 7.06 (d, *J* = 2.1 Hz, 1H), 6.95 (dd, *J* = 8.3, 2.1 Hz, 1H), 6.84 (d, *J* = 8.2 Hz, 1H), 4.22 (s, 4H), 3.85 (q, *J* = 5.4 Hz, 2H).

^{13}C NMR (101 MHz, DMSO) δ 143.49, 143.15, 127.00, 122.11, 117.98, 117.02, 64.13, 64.09, 41.59.

(1-methyl-1H-benzo[d][1,2,3]triazol-5-yl)methanamine hydrochloride

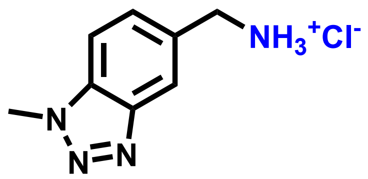

^{1}H NMR (300 MHz, DMSO) δ 8.62 (bs, 3H), 8.17 (s, 1H), 7.90 (d, *J* = 8.6 Hz, 1H), 7.69 (d, *J* = 8.5 Hz, 1H), 4.29 (s, 3H), 4.16 (q, *J* = 5.6 Hz, 2H).

^{13}C NMR (75 MHz, DMSO) δ 129.65, 128.07, 119.27, 110.46, 41.71, 34.02.

furan-3-ylmethanamine hydrochloride

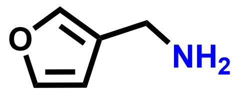

^{1}H NMR (300 MHz, DMSO) δ 8.48 (bs, 3H), 7.77 (dq, *J* = 1.7, 0.9 Hz, 1H), 7.68 (t, *J* = 1.7 Hz, 1H), 6.69 (dd, *J* = 1.8, 0.9 Hz, 1H), 3.85 (q, *J* = 5.9 Hz, 2H).

^{13}C NMR (75 MHz, DMSO) δ 143.77, 141.86, 118.53, 111.03, 33.35.

3-phenylpropan-1-amine hydrochloride

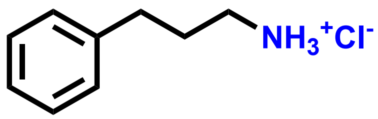

¹H NMR (300 MHz, DMSO) δ 8.25 (bs, 3H), 7.34 – 7.13 (m, 5H), 2.74 (dt, J = 8.7, 5.9 Hz, 2H), 2.64 (t, J = 7.7 Hz, 2H), 1.88 (p, J = 7.7 Hz, 2H).

¹³C NMR (75 MHz, DMSO) δ 140.94, 128.41, 128.29, 126.01, 38.27, 31.90, 28.70.

2-(3,4-dimethoxyphenyl)ethan-1-amine hydrochloride

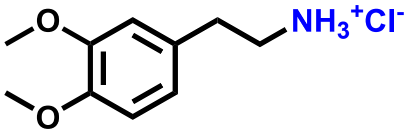

¹H NMR (300 MHz, DMSO) δ 8.30 (bs, 3H), 6.92 – 6.81 (m, 2H), 6.74 (dd, J = 8.1, 2.1 Hz, 1H), 3.73 (s, 3H), 3.70 (s, 3H), 2.92 (ddd, J = 36.0, 9.1, 5.7 Hz, 4H).

¹³C NMR (75 MHz, DMSO) δ 148.81, 147.63, 129.87, 120.55, 112.55, 112.04, 55.57, 55.50, 40.11, 32.50.

2-(6-methoxynaphthalen-2-yl)propan-1-amine hydrochloride

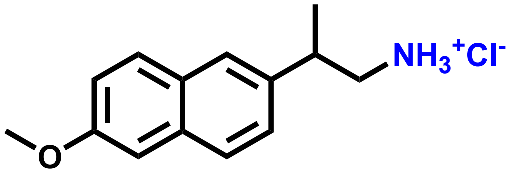

¹H NMR (300 MHz, DMSO) δ 8.08 (bs, 3H), 7.79 (dd, J = 8.8, 4.9 Hz, 2H), 7.71 (d, J = 2.0 Hz, 1H), 7.42 (dd, J = 8.5, 1.9 Hz, 1H), 7.30 (d, J = 2.7 Hz, 1H), 7.15 (dd, J = 9.0, 2.6 Hz, 1H), 3.86 (s, 3H), 3.21 (h, J = 6.9 Hz, 1H), 3.06 (s, 2H), 1.33 (d, J = 6.9 Hz, 3H).

¹³C NMR (75 MHz, DMSO) δ 157.12, 137.84, 133.45, 129.13, 128.55, 127.23, 126.07, 125.49, 118.67, 105.75, 55.20, 44.90, 37.39, 19.42.

2-phenylpropan-1-amine hydrochloride

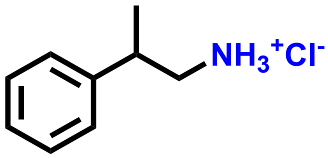

^{1}H NMR (300 MHz, DMSO) δ 8.26 (bs, 3H), 7.41 – 7.12 (m, 5H), 3.12 (dt, *J* = 8.2, 6.8 Hz, 1H), 2.94 (p, *J* = 5.8 Hz, 2H), 1.26 (d, *J* = 6.9 Hz, 3H).

^{13}C NMR (75 MHz, DMSO) δ 143.07, 128.66, 127.15, 126.86, 44.95, 37.31, 19.34

2,2-diphenylethan-1-amine hydrochloride

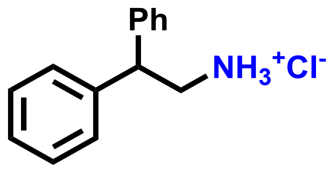

^{1}H NMR (300 MHz, DMSO) δ 8.34 (bs, 3H), 7.40 – 7.18 (m, 10H), 4.47 (t, *J* = 7.9 Hz, 1H), 3.53 (d, *J* = 5.2 Hz, 2H).

^{13}C NMR (75 MHz, DMSO) δ 141.19, 128.78, 127.89, 126.99, 48.43, 42.43.

Cyclopentylmethanamine hydrochloride

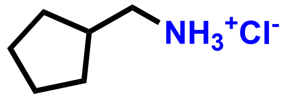

^{1}H NMR (300 MHz, DMSO) δ 8.17 (bs, 3H), 2.69 (dq, *J* = 7.6, 5.9 Hz, 2H), 2.10 (hept, *J* = 7.7 Hz, 1H), 1.83 – 1.65 (m, 2H), 1.64 – 1.39 (m, 4H), 1.31 – 1.10 (m, 2H).

^{13}C NMR (75 MHz, DMSO) δ 43.28, 37.49, 29.88, 24.61.

Cyclohexylmethanamine hydrochloride

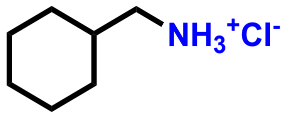

^{1}H NMR (300 MHz, DMSO) δ 7.96 (bs, 3H), 2.61 (p, *J* = 6.1 Hz, 2H), 1.80 – 1.47 (m, 6H), 1.29 – 1.04 (m, 3H), 1.01 – 0.80 (m, 2H).

^{13}C NMR (75 MHz, DMSO) δ 44.38, 35.43, 29.75, 25.65, 25.05.

Octan-1-amine hydrochloride

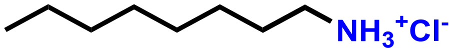

^{1}H NMR (300 MHz, DMSO) δ 8.03 (bs, 3H), 2.82 – 2.63 (m, 2H), 1.53 (q, *J* = 7.7 Hz, 2H), 1.37 – 1.16 (m, 10H), 0.94 – 0.76 (m, 3H).

^{13}C NMR (75 MHz, $CDCl_3$) δ 38.69, 31.17, 28.50, 26.93, 25.86, 22.08, 13.96.

Decan-1-amine hydrochloride

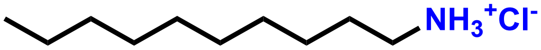

^{1}H NMR (300 MHz, DMSO) δ 8.08 (bs, 3H), 2.71 (dt, *J* = 9.3, 5.8 Hz, 2H), 1.54 (p, *J* = 7.5 Hz, 2H), 1.22 (s, 14H), 0.95 – 0.73 (m, 3H).

^{13}C NMR (75 MHz, DMSO) δ 38.88, 31.44, 29.07, 29.01, 28.85, 28.72, 27.01, 26.05, 22.24, 14.06.

Dodecan-1-amine hydrochloride

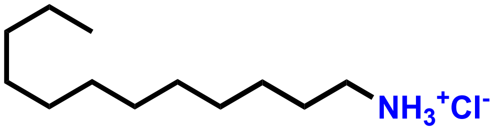

^{1}H NMR (300 MHz, DMSO) δ 8.10 (bs, 3H), 2.71 (t, *J* = 7.6 Hz, 2H), 1.55 (p, *J* = 7.3 Hz, 2H), 1.38 – 1.12 (m, 18H), 0.94 – 0.74 (m, 3H).

^{13}C NMR (75 MHz, DMSO) δ 38.67, 31.30, 29.05, 29.02, 28.95, 28.86, 28.71, 28.56, 26.90, 25.88, 22.09, 13.93.

Tetradecan-1-amine

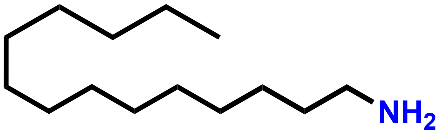

^{1}H NMR (300 MHz, $CDCl_3$) δ 3.32 (s, 2H), 2.71 (t, *J* = 7.2 Hz, 2H), 1.47 (q, *J* = 7.3 Hz, 2H), 1.27 – 1.19 (m, 22H), 0.86 (t, *J* = 6.7 Hz, 3H).

^{13}C NMR (75 MHz, $CDCl_3$) δ 41.71, 32.49, 32.05, 29.82, 29.78, 29.73, 29.54, 29.48, 26.97, 22.81, 14.22.

<u>Hexadecan-1-amine</u>

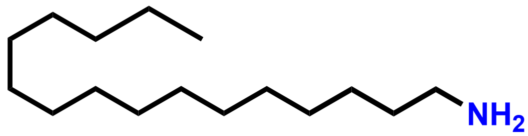

^{1}H NMR (300 MHz, $CDCl_3$) δ 2.74 – 2.60 (m, 2H), 1.51 – 1.39 (m, 2H), 1.25 (s, 28H), 0.95 – 0.79 (m, 3H).

^{13}C NMR (75 MHz, $CDCl_3$) δ 42.45, 34.07, 32.07, 29.84, 29.80, 29.78, 29.67, 29.51, 27.05, 22.84, 14.26.

<u>Octadecan-1-amine</u>

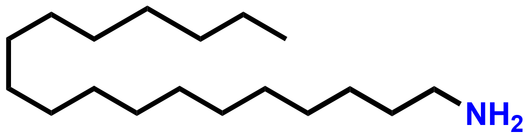

^{1}H NMR (300 MHz, $CDCl_3$) δ ^{1}H NMR (300 MHz, $CDCl_3$) δ 2.83 – 2.48 (m, 2H), 1.40 – 1.18 (m, 32H), 0.95 – 0.79 (m, 3H).

^{13}C NMR (75 MHz, $CDCl_3$) δ ^{13}C NMR (75 MHz, $CDCl_3$) δ 42.44, 34.06, 32.07, 29.84, 29.82, 29.80, 29.78, 29.67, 29.51, 27.06, 22.84, 14.26.

17. NMR spectra

230623.319.10.fid
Fairoosa Poovan FPH-749-PA
Au1H DMSO {C:\Bruker\TopSpin3.6.2} 2306 19

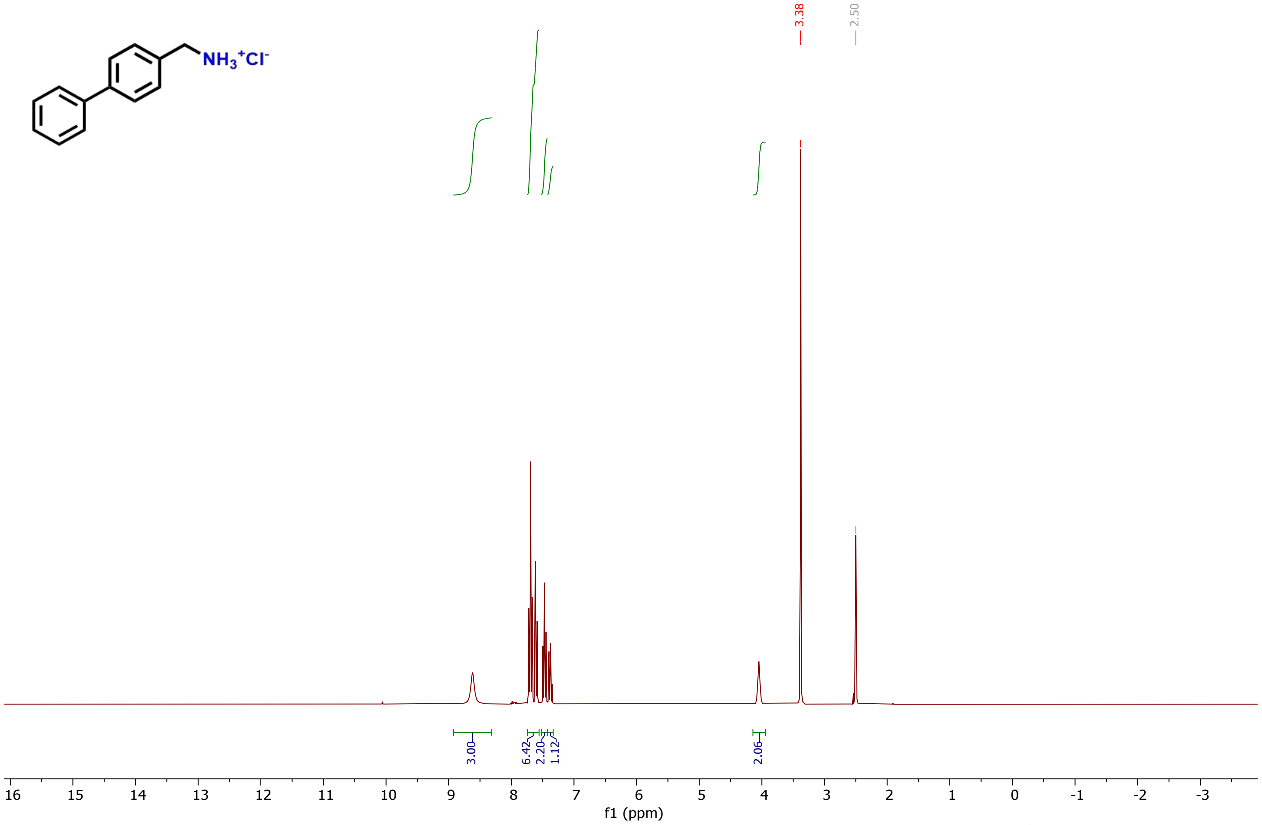

230623.319.11.fid
Fairoosa Poovan FPH-749-PA
Au13C DMSO {C:\Bruker\TopSpin3.6.2} 2306 19

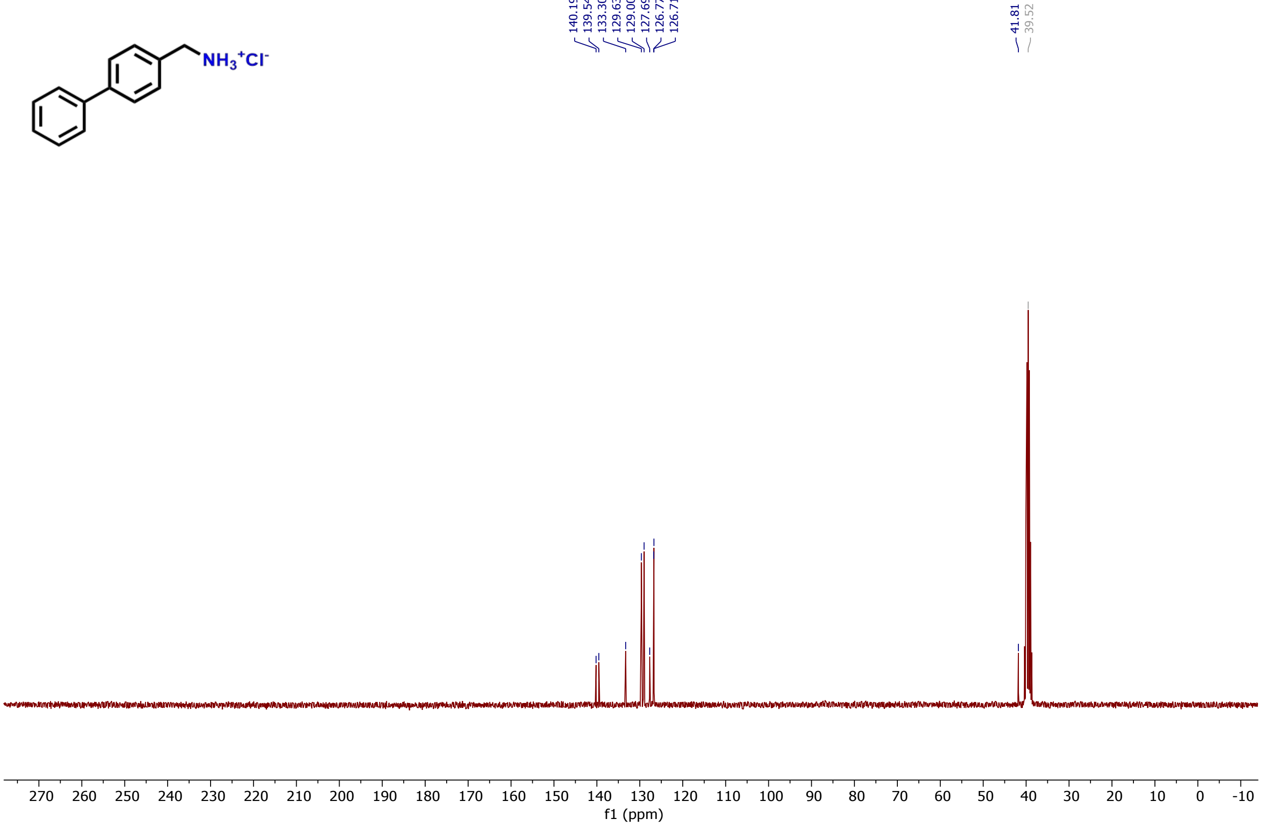

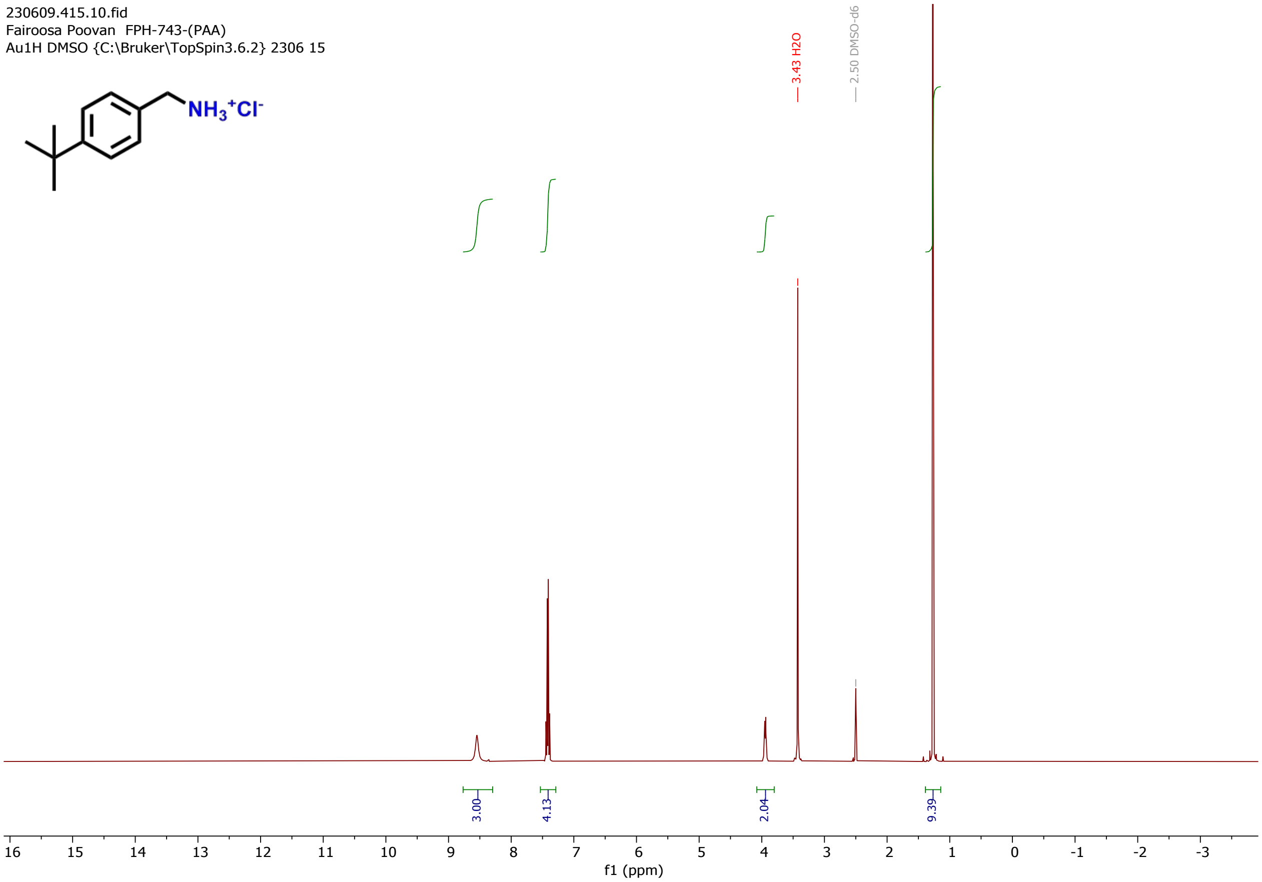
230609.415.10.fid
Fairoosa Poovan FPH-743-(PAA)
Au1H DMSO {C:\Bruker\TopSpin3.6.2} 2306 15
NH3+Cl-
3.43 H2O
2.50 DMSO-d6
3.00
4.13
2.04
9.39
f1 (ppm)

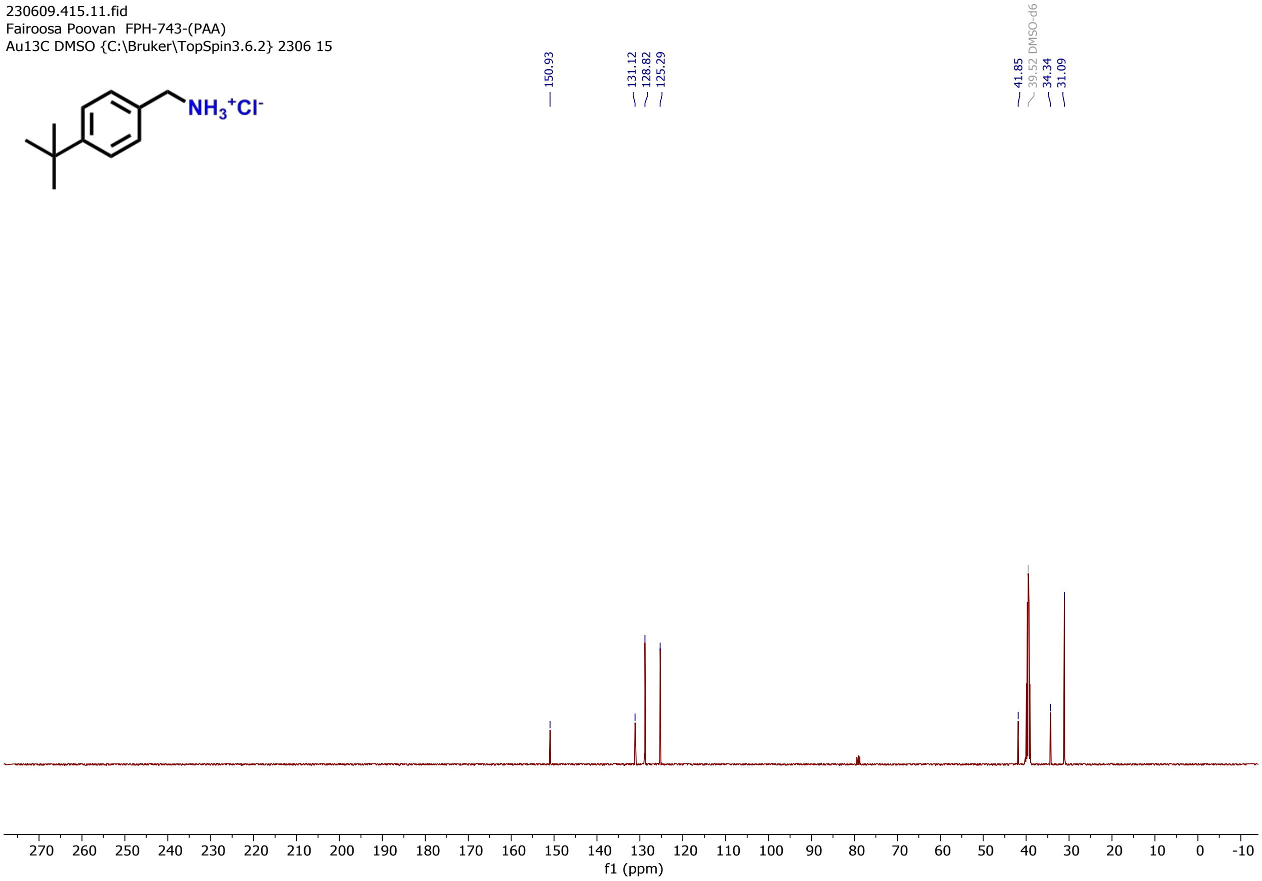
230609.415.11.fid
Fairoosa Poovan FPH-743-(PAA)
Au13C DMSO {C:\Bruker\TopSpin3.6.2} 2306 15
NH3+Cl-
150.93
131.12
128.82
125.29
41.85
39.52 DMSO-d6
34.34
31.09
f1 (ppm)

230623.322.10.fid
Fairoosa Poovan FPH-519-PA
Au1H DMSO {C:\Bruker\TopSpin3.6.2} 2306 22

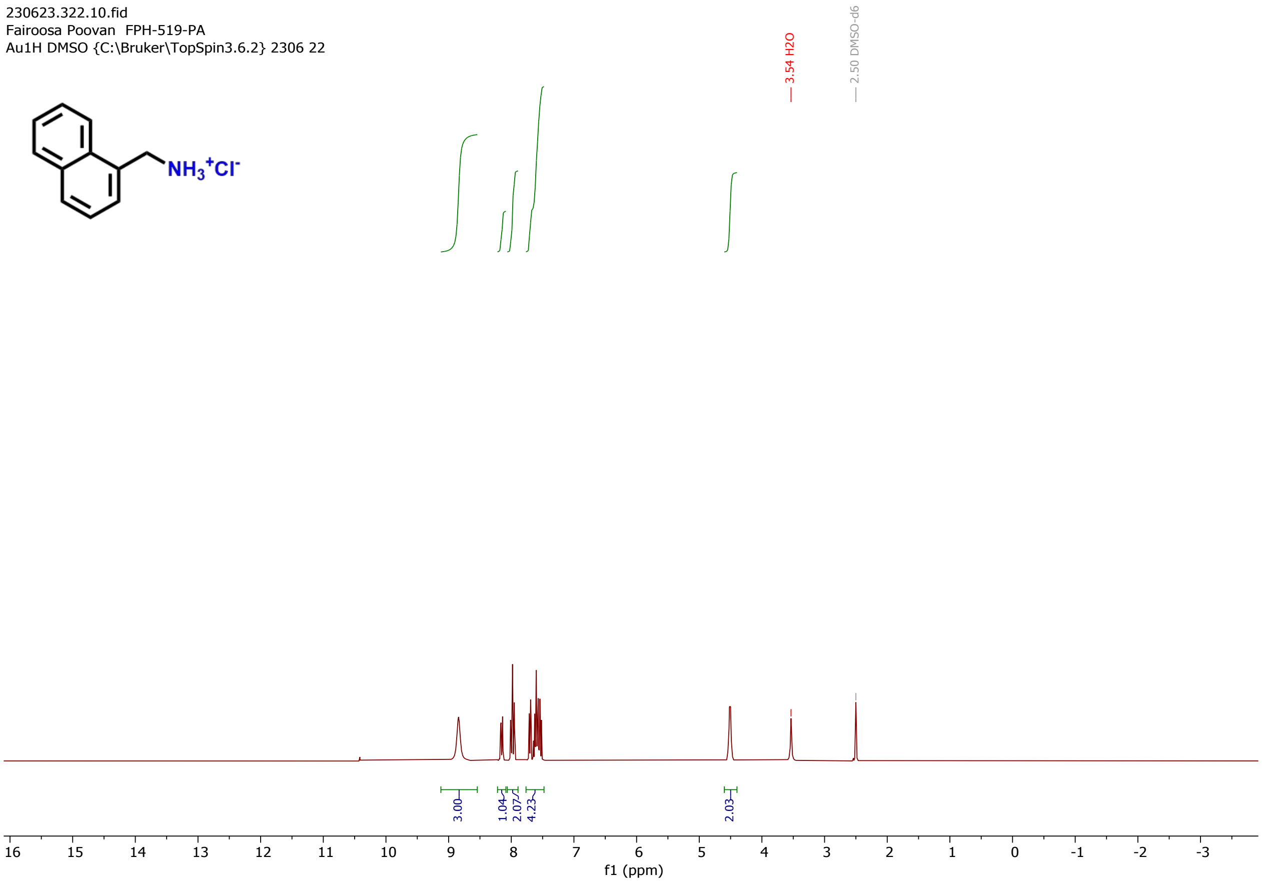

230623.322.11.fid
Fairoosa Poovan FPH-519-PA
Au13C DMSO {C:\Bruker\TopSpin3.6.2} 2306 22

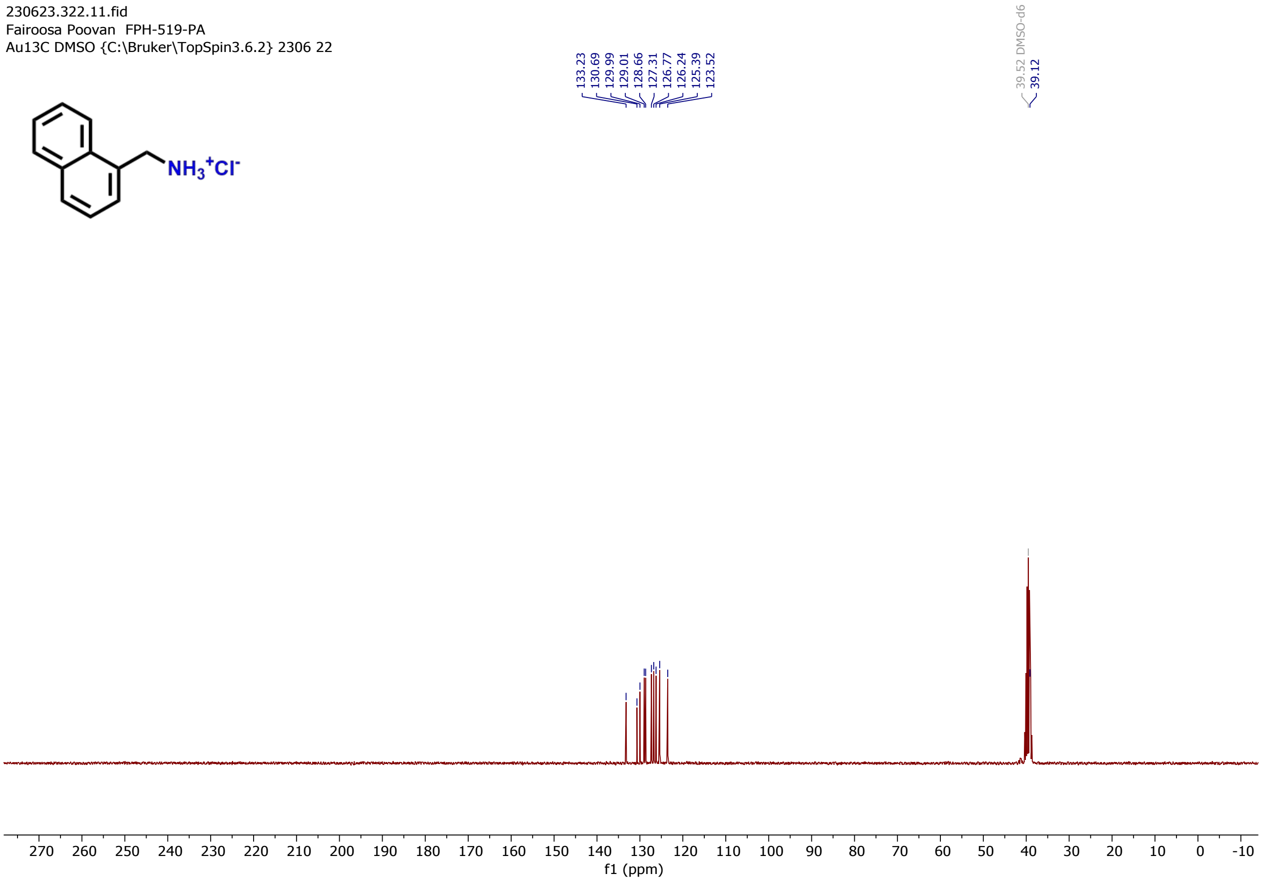

230526.f318.10.fid
Fairoosa Poovan FPH-361-(N)
PROTON DMSO {C:\Bruker\TopSpin3.6.2} 2305 18

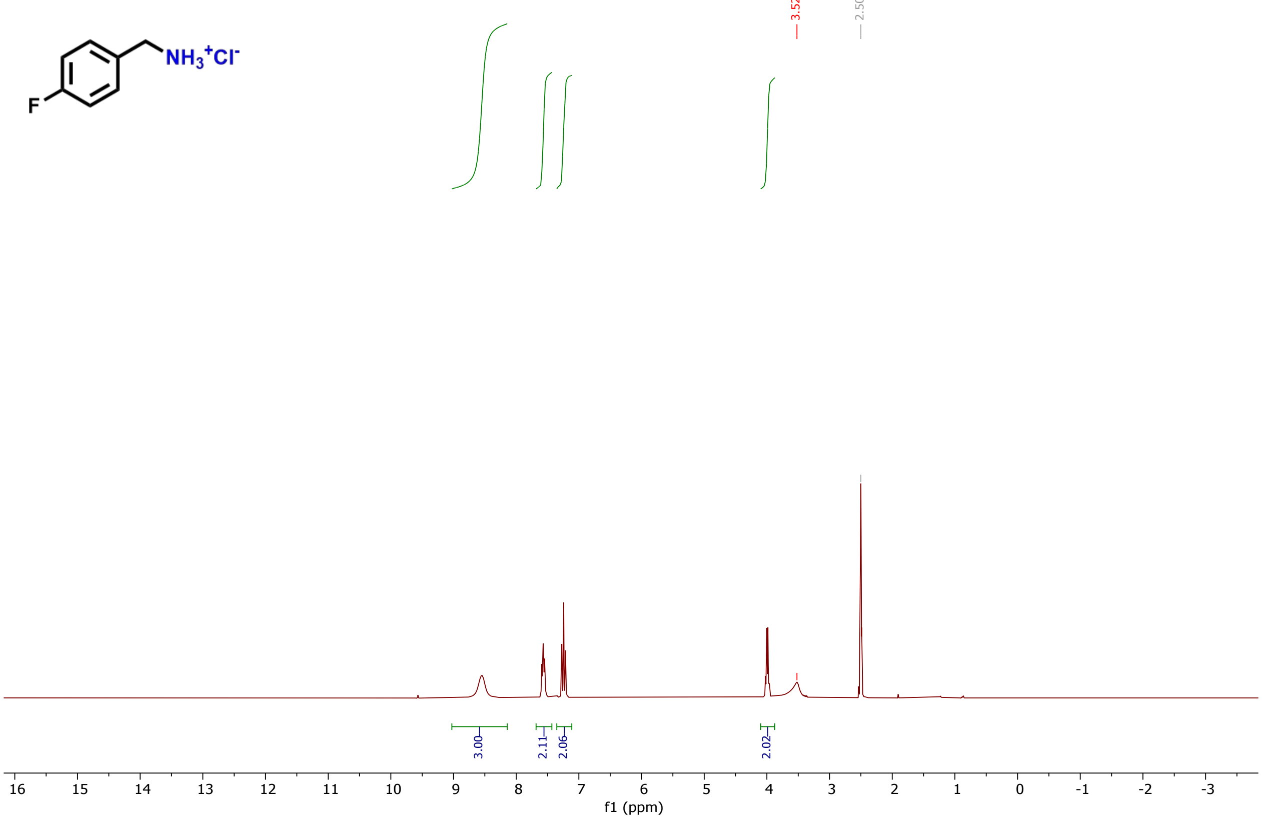

230615.311.10.fid
Fairoosa Poovan FPH-361-PA
Au13C DMSO {C:\Bruker\TopSpin3.6.2} 2306 11

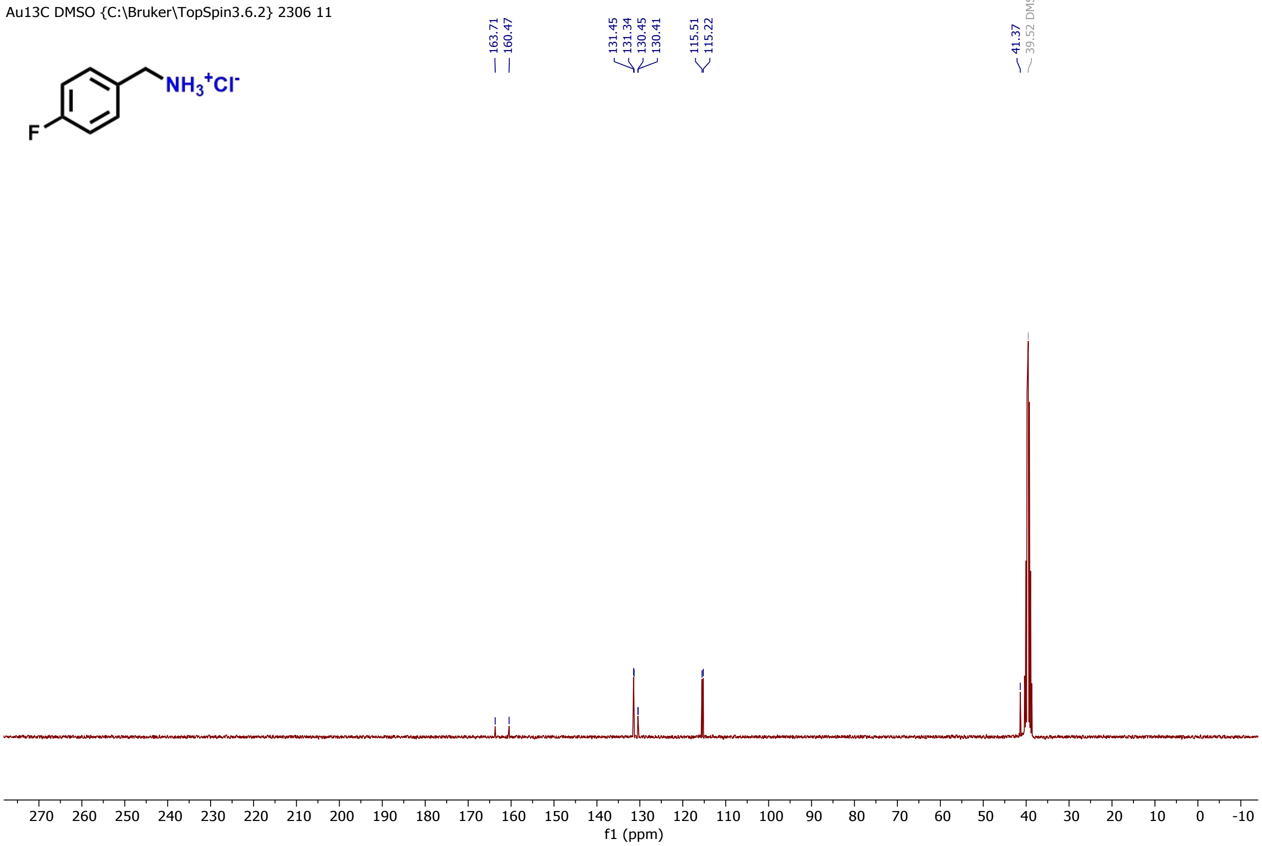

230615.317.10.fid
Fairoosa Poovan FPH-386-PA
Au1H DMSO {C:\Bruker\TopSpin3.6.2} 2306 17

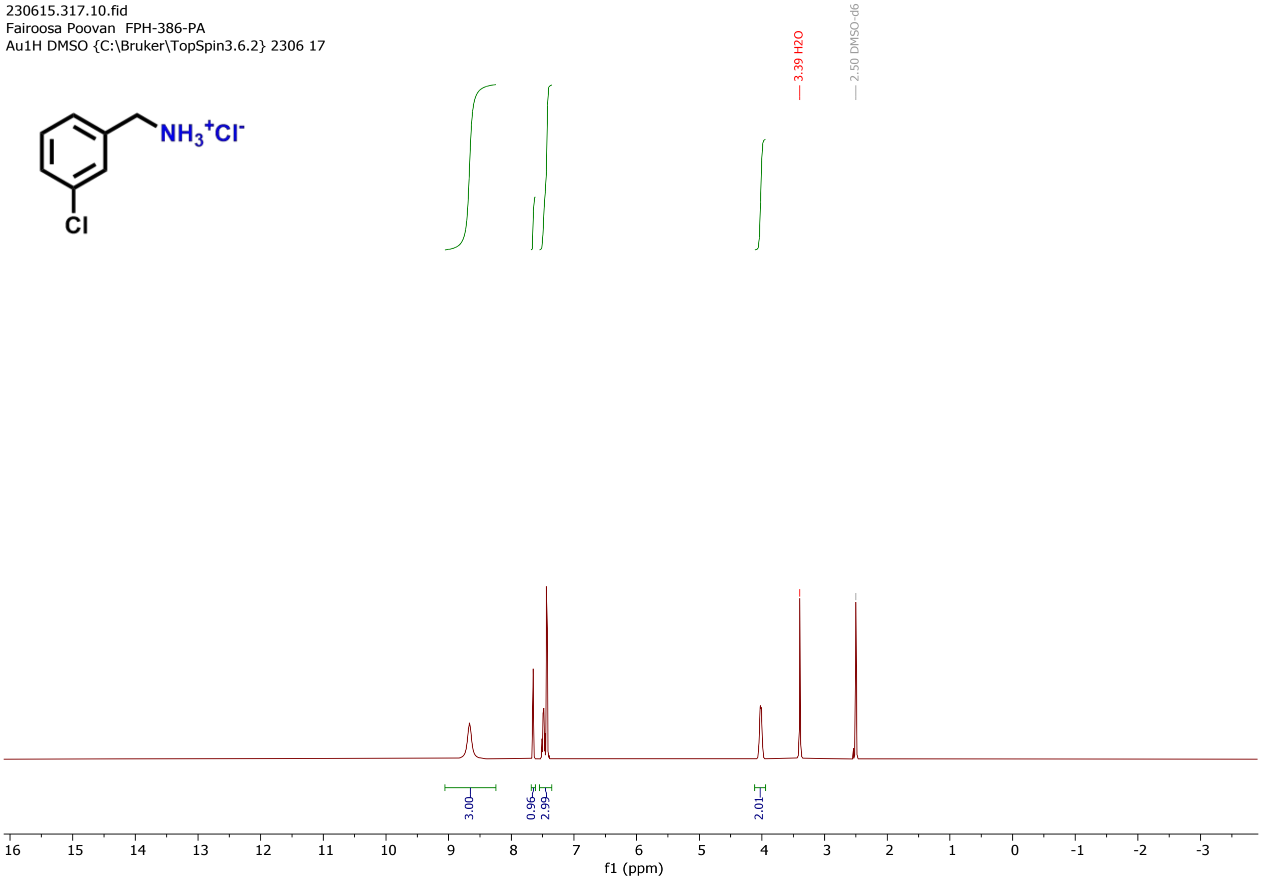

230615.317.11.fid
Fairoosa Poovan FPH-386-PA
Au13C DMSO {C:\Bruker\TopSpin3.6.2} 2306 17

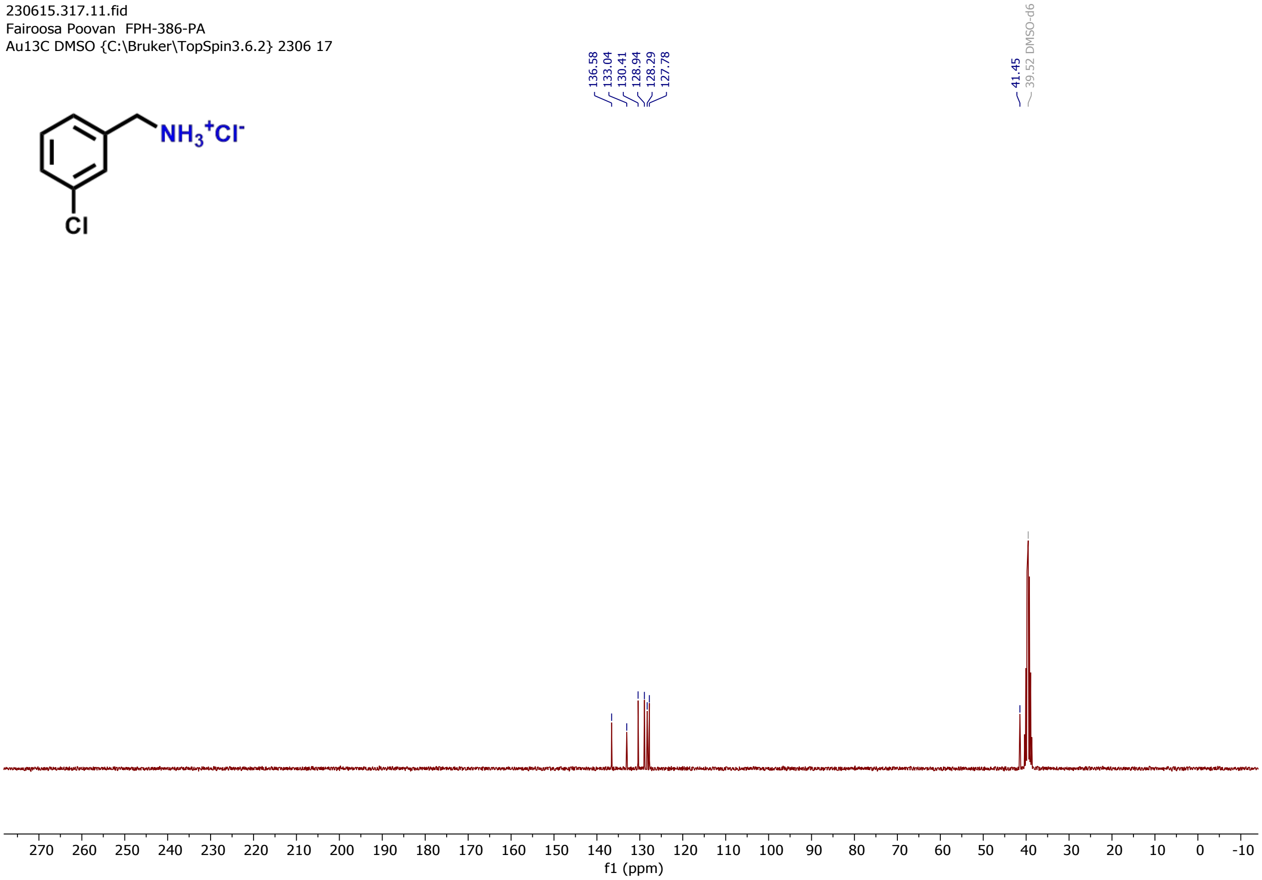

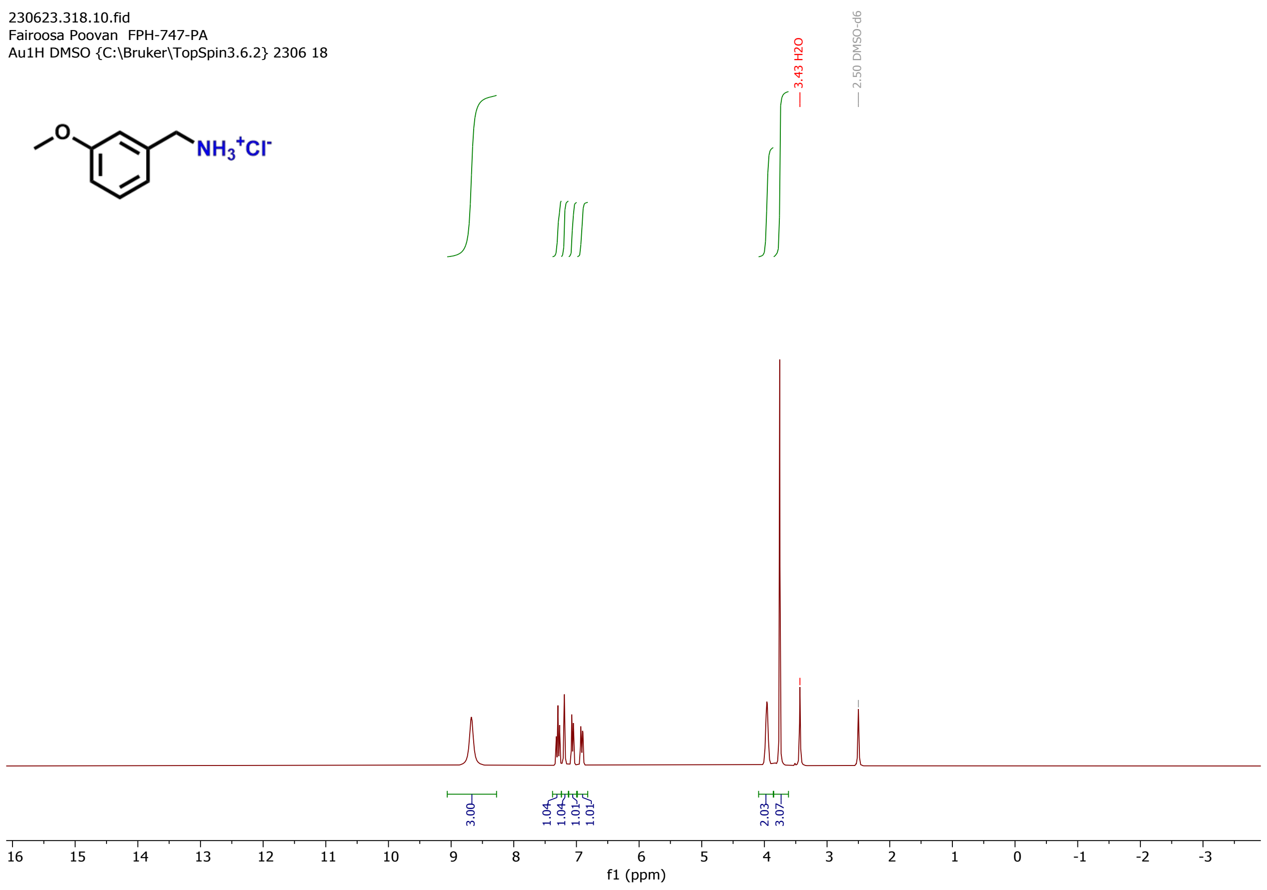
230623.318.10.fid
Fairoosa Poovan FPH-747-PA
Au1H DMSO {C:\Bruker\TopSpin3.6.2} 2306 18
3.43 H2O
2.50 DMSO-d6
NH3+Cl-
O
3.00
1.04
1.04
1.01
1.01
2.03
3.07
f1 (ppm)

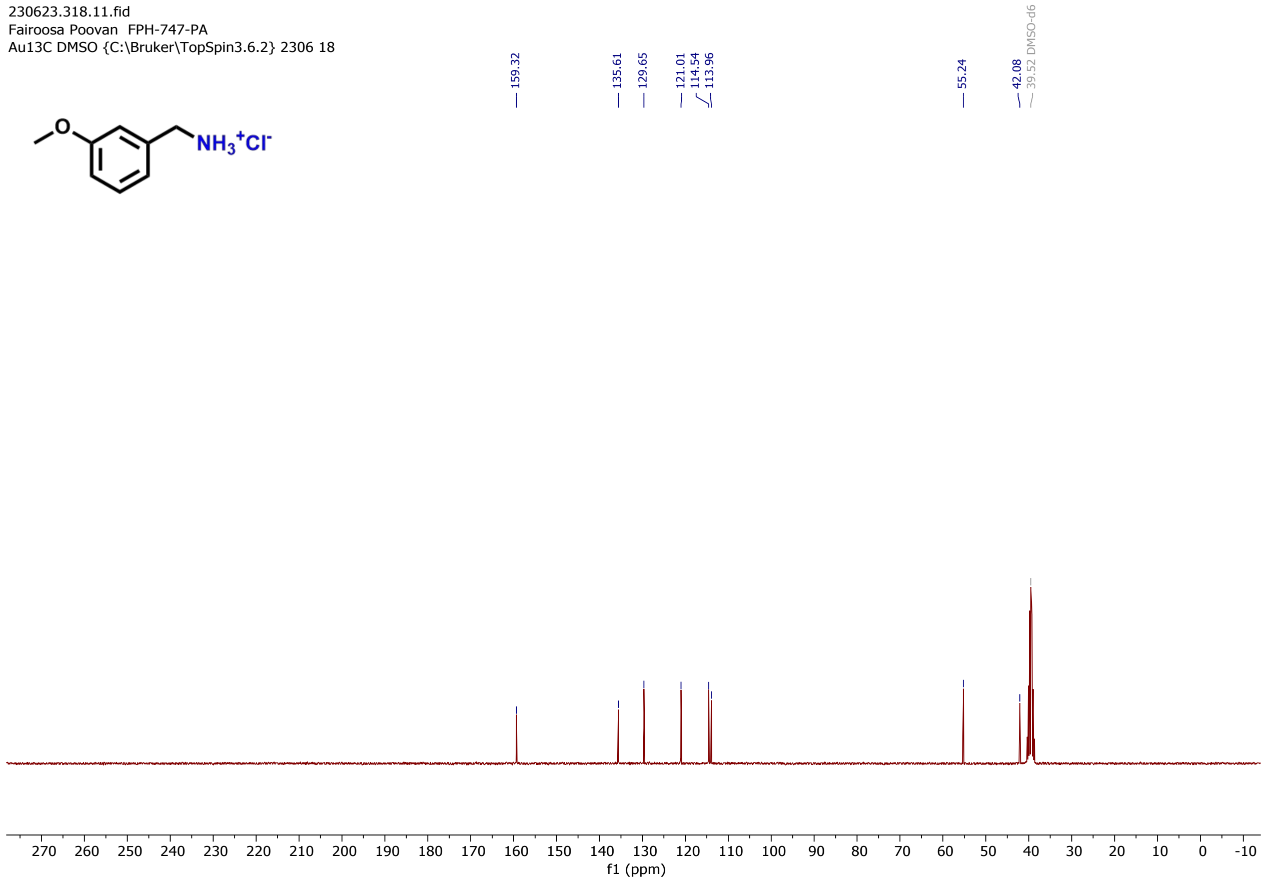
230623.318.11.fid
Fairoosa Poovan FPH-747-PA
Au13C DMSO {C:\Bruker\TopSpin3.6.2} 2306 18
159.32
135.61
129.65
121.01
114.54
113.96
55.24
42.08
39.52 DMSO-d6
NH3+Cl-
O
f1 (ppm)

230703.311.12.fid
Fairoosa Poovan FPH-772-PA Wdhl.
Au1H DMSO {C:\Bruker\TopSpin3.6.2} 2307 13

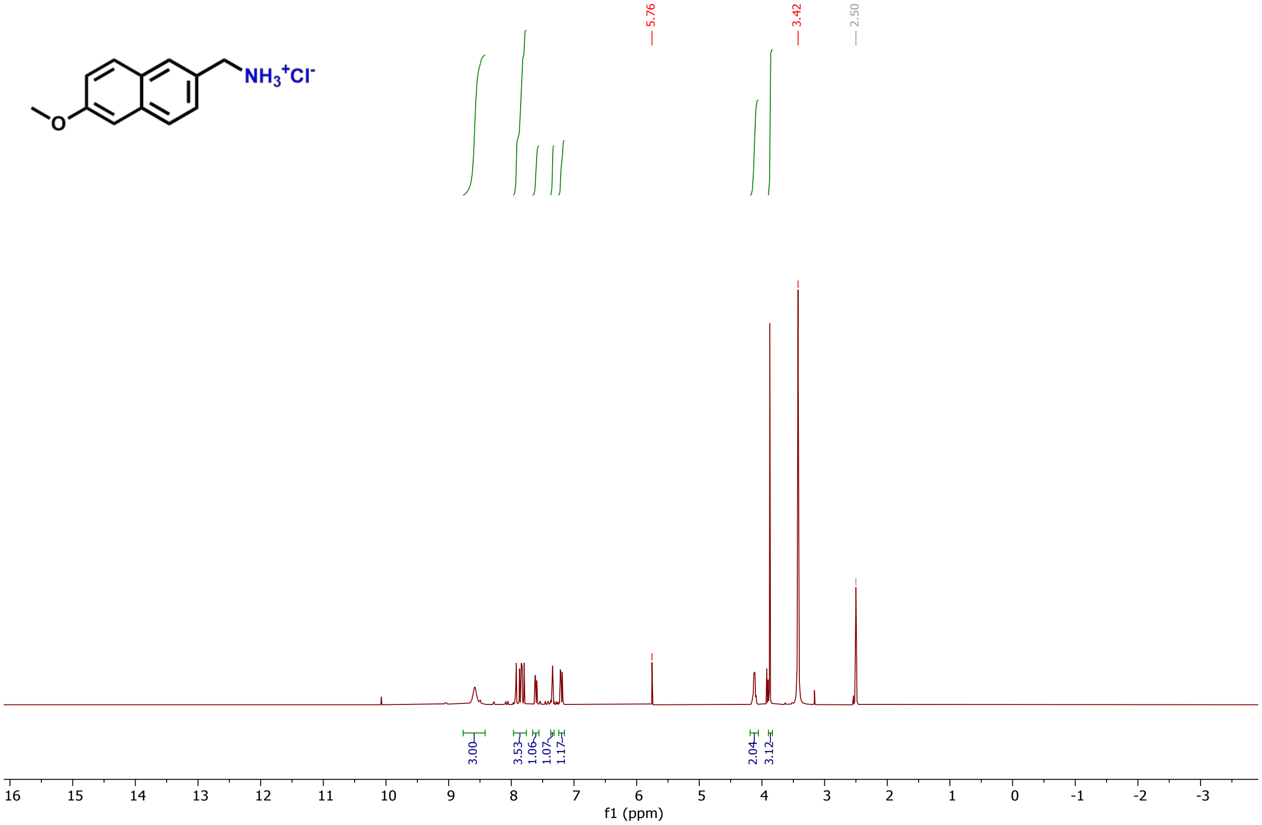

230703.311.13.fid
Fairoosa Poovan FPH-772-PA Wdhl.
Au13C DMSO {C:\Bruker\TopSpin3.6.2} 2307 13

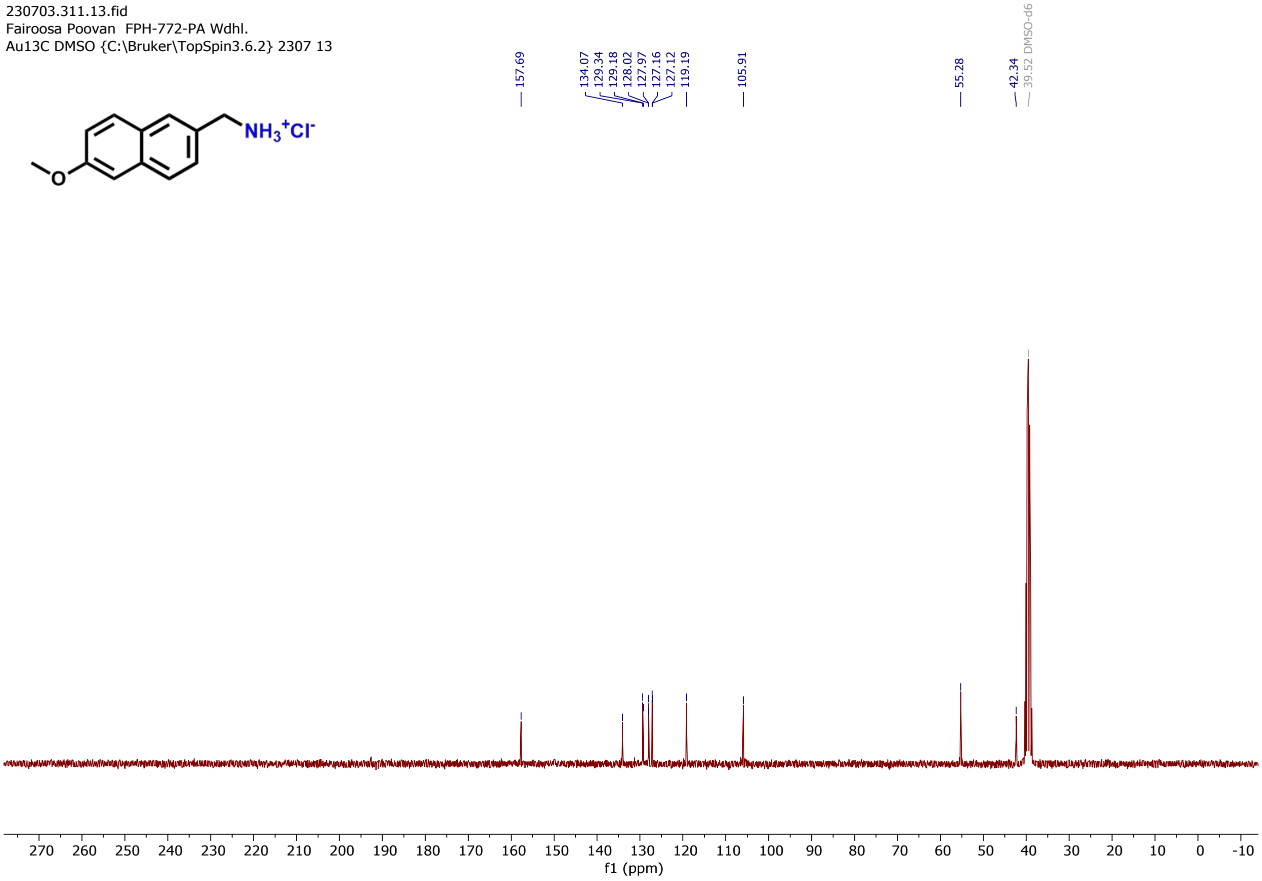

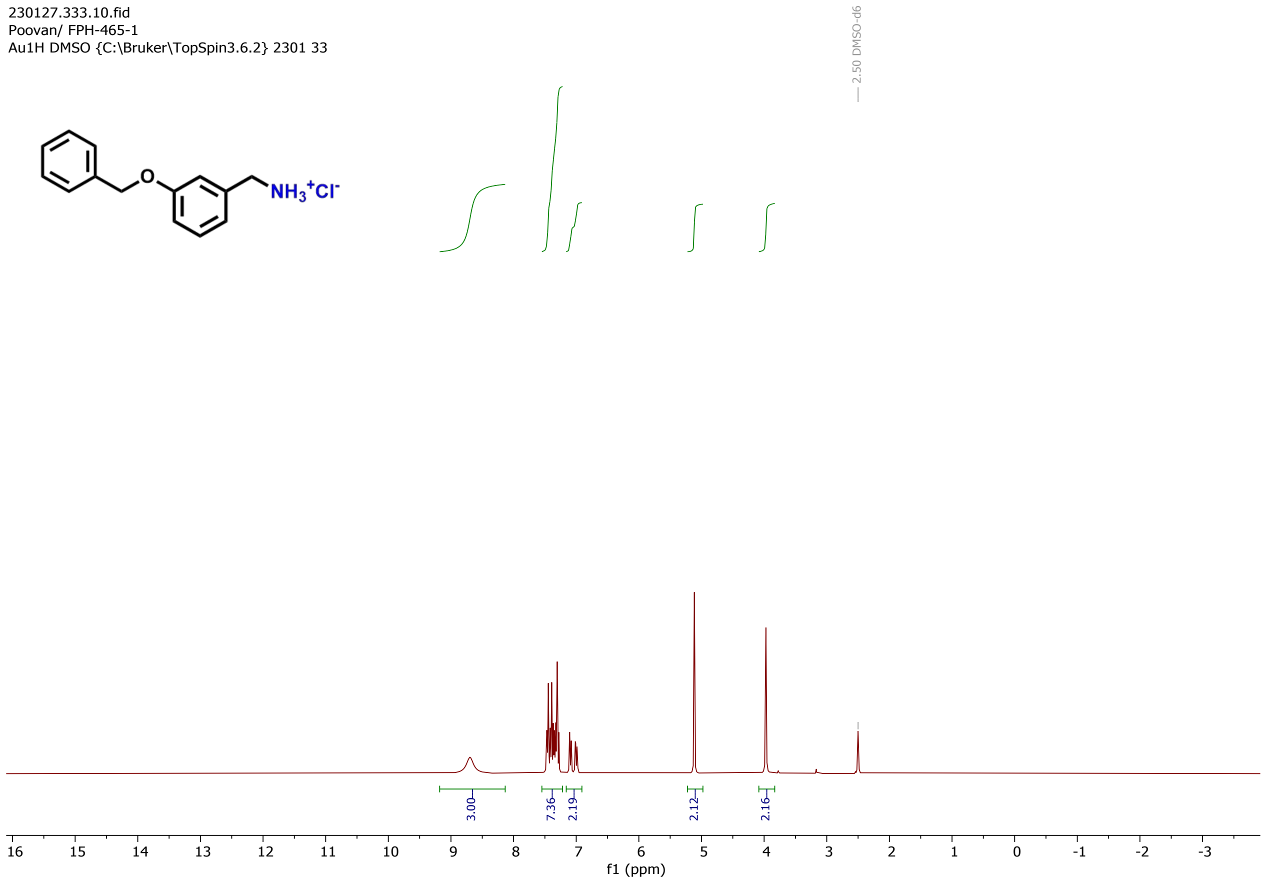
230127.333.10.fid
Poovan/ FPH-465-1
Au1H DMSO {C:\Bruker\TopSpin3.6.2} 2301 33
2.50 DMSO-d6
NH3+Cl-
3.00
7.36
2.19
2.12
2.16
16 15 14 13 12 11 10 9 8 7 6 5 4 3 2 1 0 -1 -2 -3
f1 (ppm)

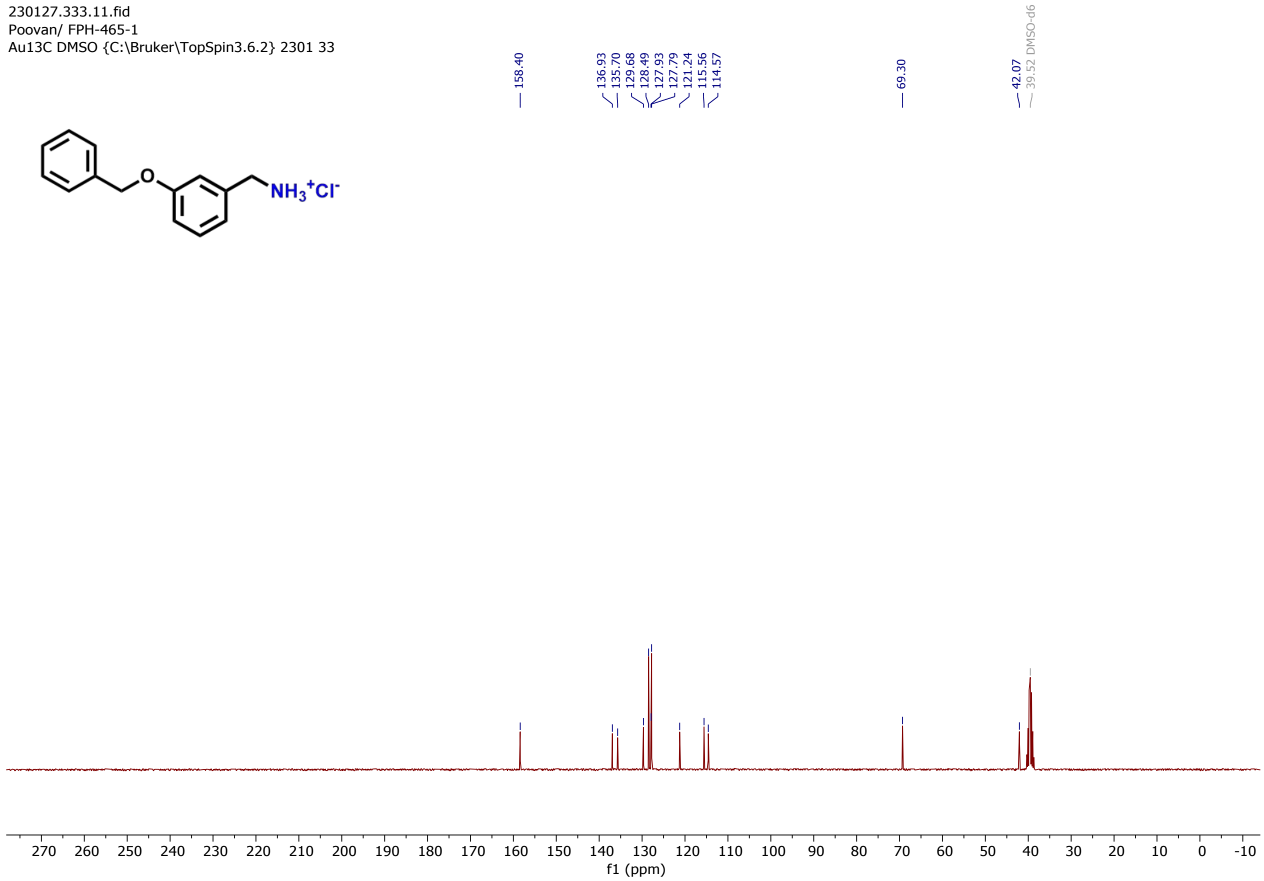
230127.333.11.fid
Poovan/ FPH-465-1
Au13C DMSO {C:\Bruker\TopSpin3.6.2} 2301 33
158.40
136.93
135.70
129.68
128.49
127.93
127.79
121.24
115.56
114.57
69.30
42.07
39.52 DMSO-d6
NH3+Cl-
270 260 250 240 230 220 210 200 190 180 170 160 150 140 130 120 110 100 90 80 70 60 50 40 30 20 10 0 -10
f1 (ppm)

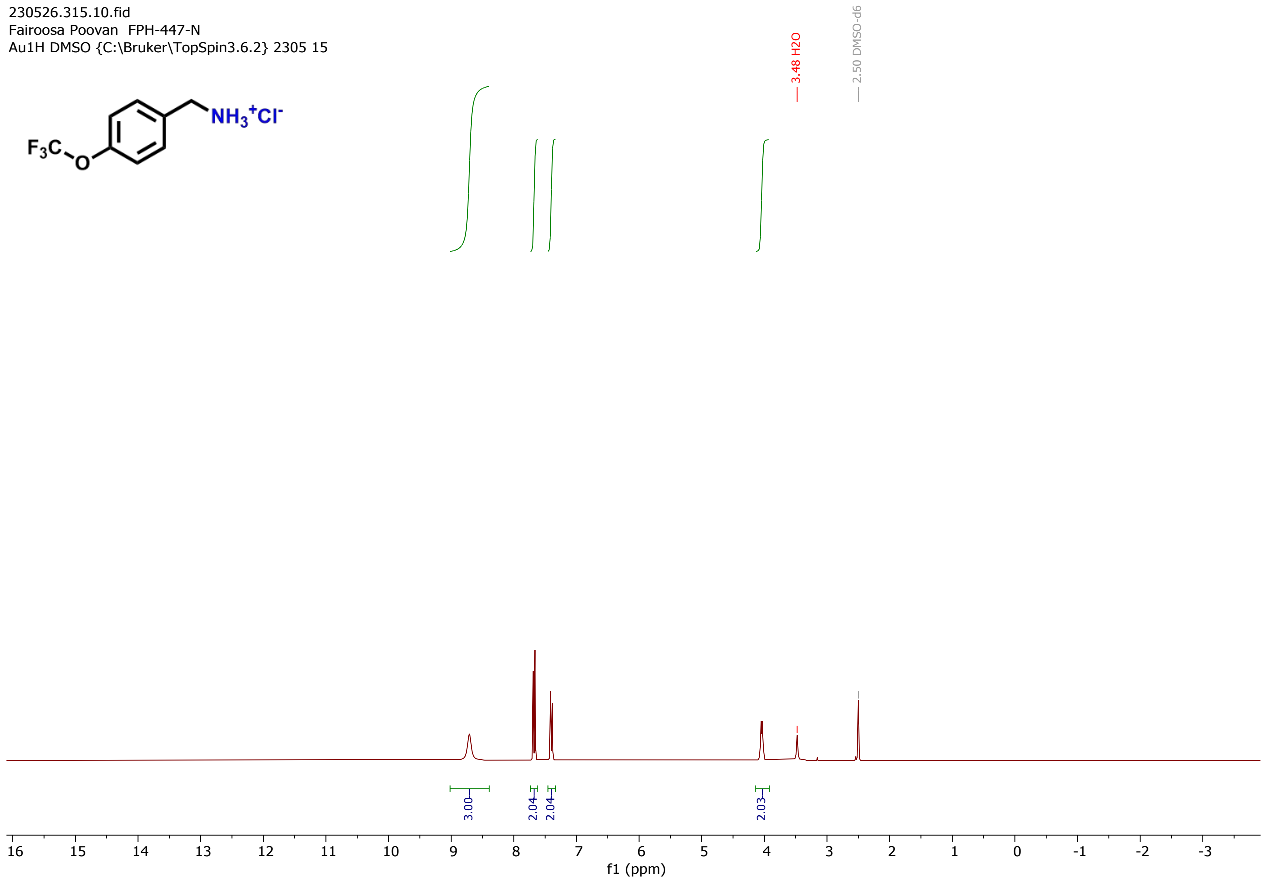
230526.315.10.fid
Fairoosa Poovan FPH-447-N
Au1H DMSO {C:\Bruker\TopSpin3.6.2} 2305 15
$NH_3^+Cl^-$
F_3C
O
3.48 H2O
2.50 DMSO-d6
3.00
2.04
2.04
2.03
16 15 14 13 12 11 10 9 8 7 6 5 4 3 2 1 0 -1 -2 -3
f1 (ppm)

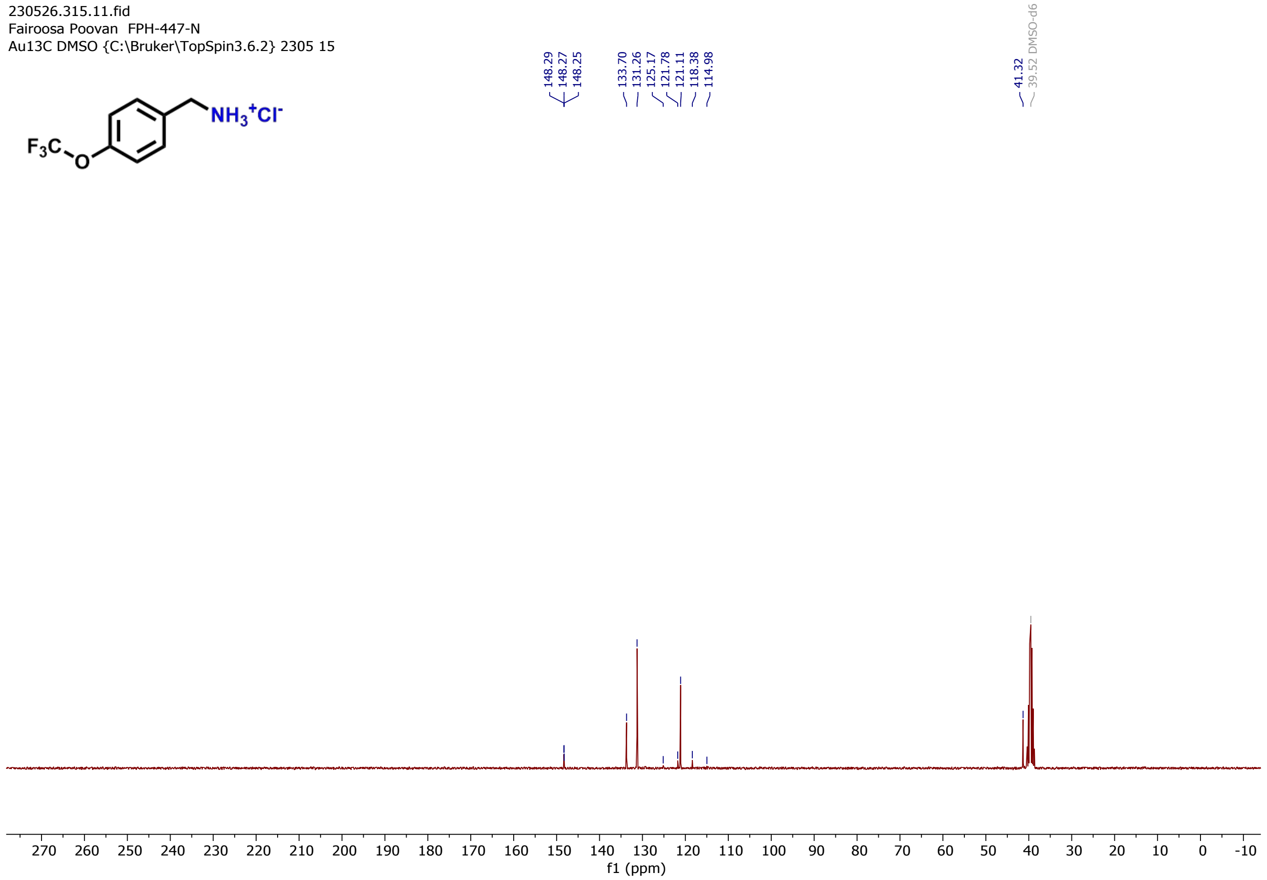
230526.315.11.fid
Fairoosa Poovan FPH-447-N
Au13C DMSO {C:\Bruker\TopSpin3.6.2} 2305 15
$NH_3^+Cl^-$
F_3C
O
148.29
148.27
148.25
133.70
131.26
125.17
121.78
121.11
118.38
114.98
41.32
39.52 DMSO-d6
270 260 250 240 230 220 210 200 190 180 170 160 150 140 130 120 110 100 90 80 70 60 50 40 30 20 10 0 -10
f1 (ppm)

230701.f350.10.fid
Fairoosa Poovan
FPH-644-PA
PROTON DMSO {C:\Bruker\TopSpin3.6.2} 2306 50

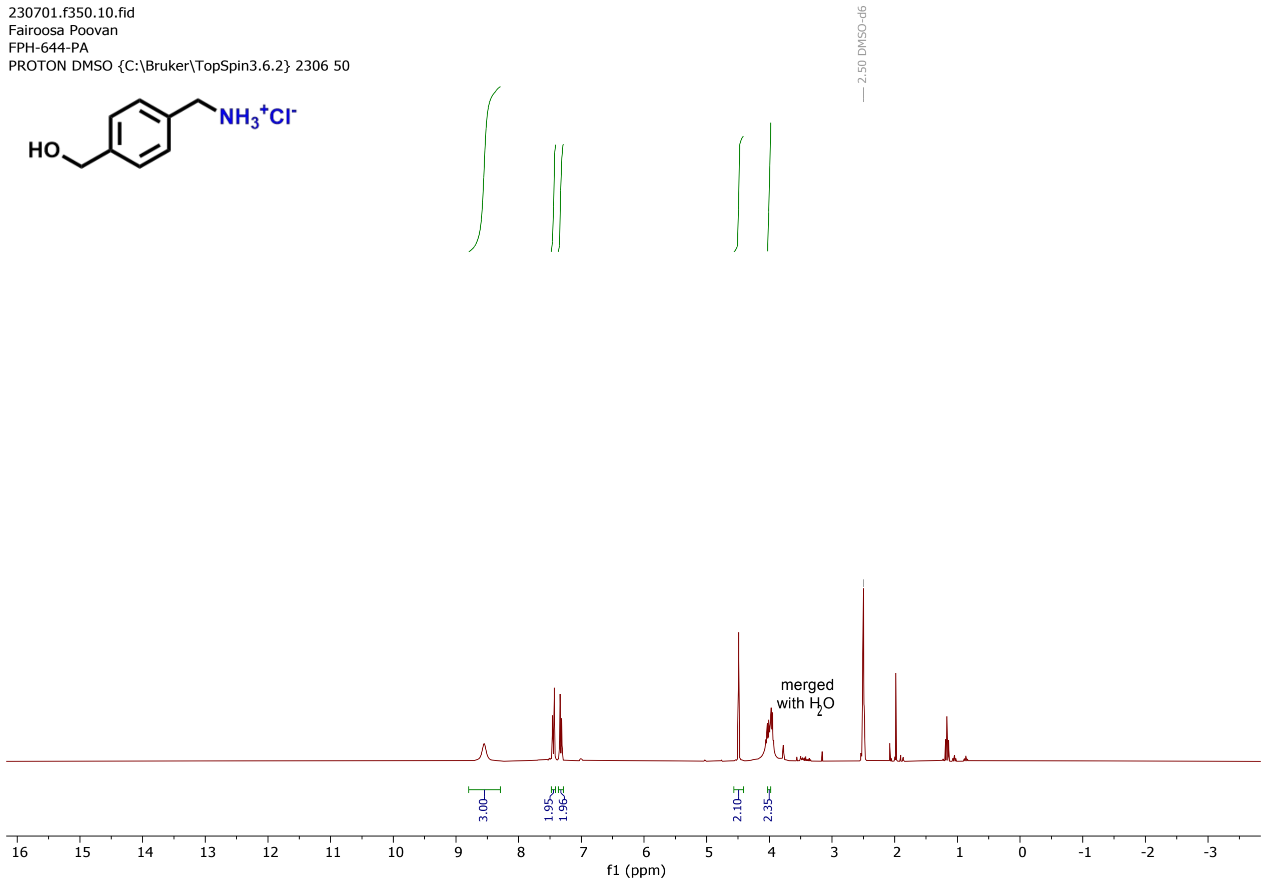

230701.f350.11.fid
Fairoosa Poovan
FPH-644-PA
C13CPD DMSO {C:\Bruker\TopSpin3.6.2} 2306 50

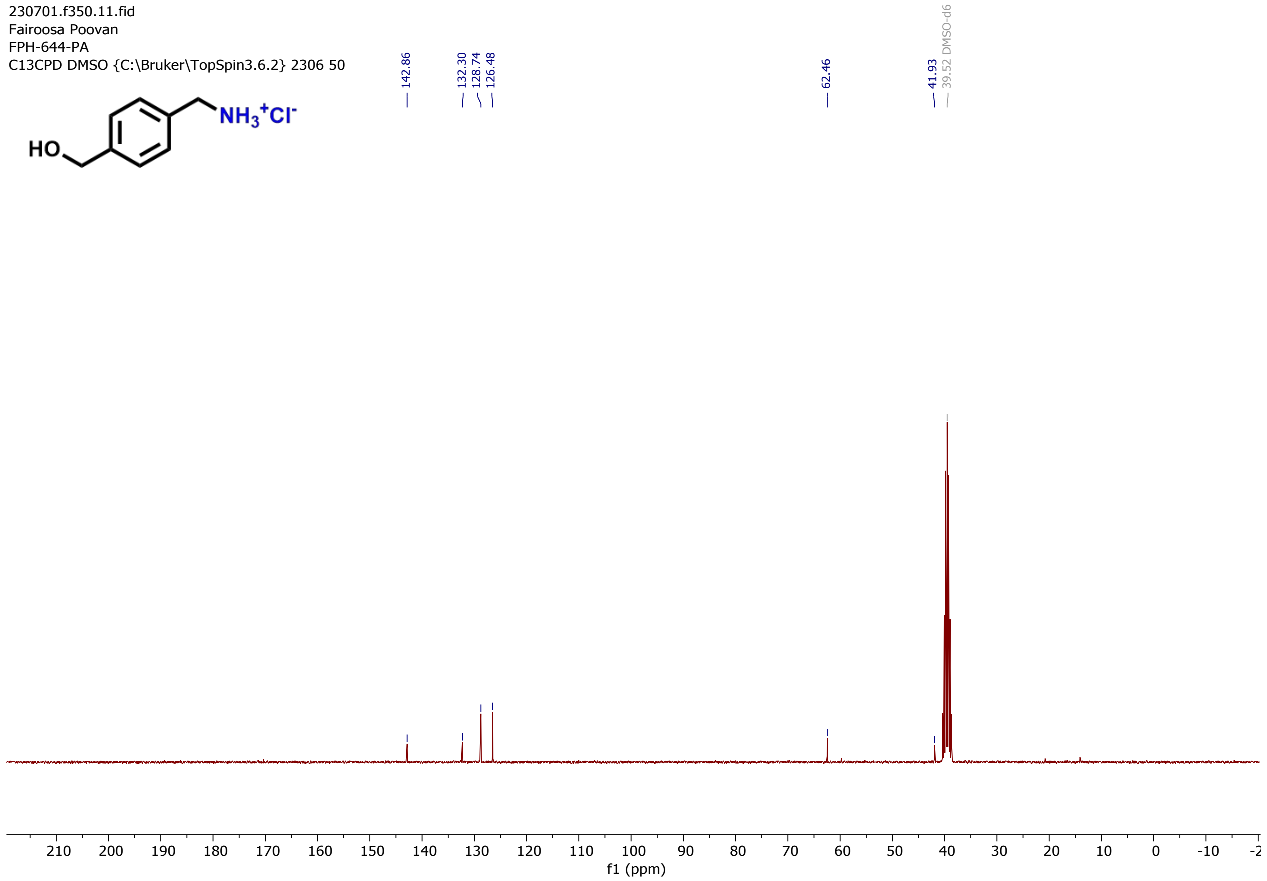

230615.316.10.fid
Fairoosa Poovan FPH-596-1-PA
Au1H DMSO {C:\Bruker\TopSpin3.6.2} 2306 16

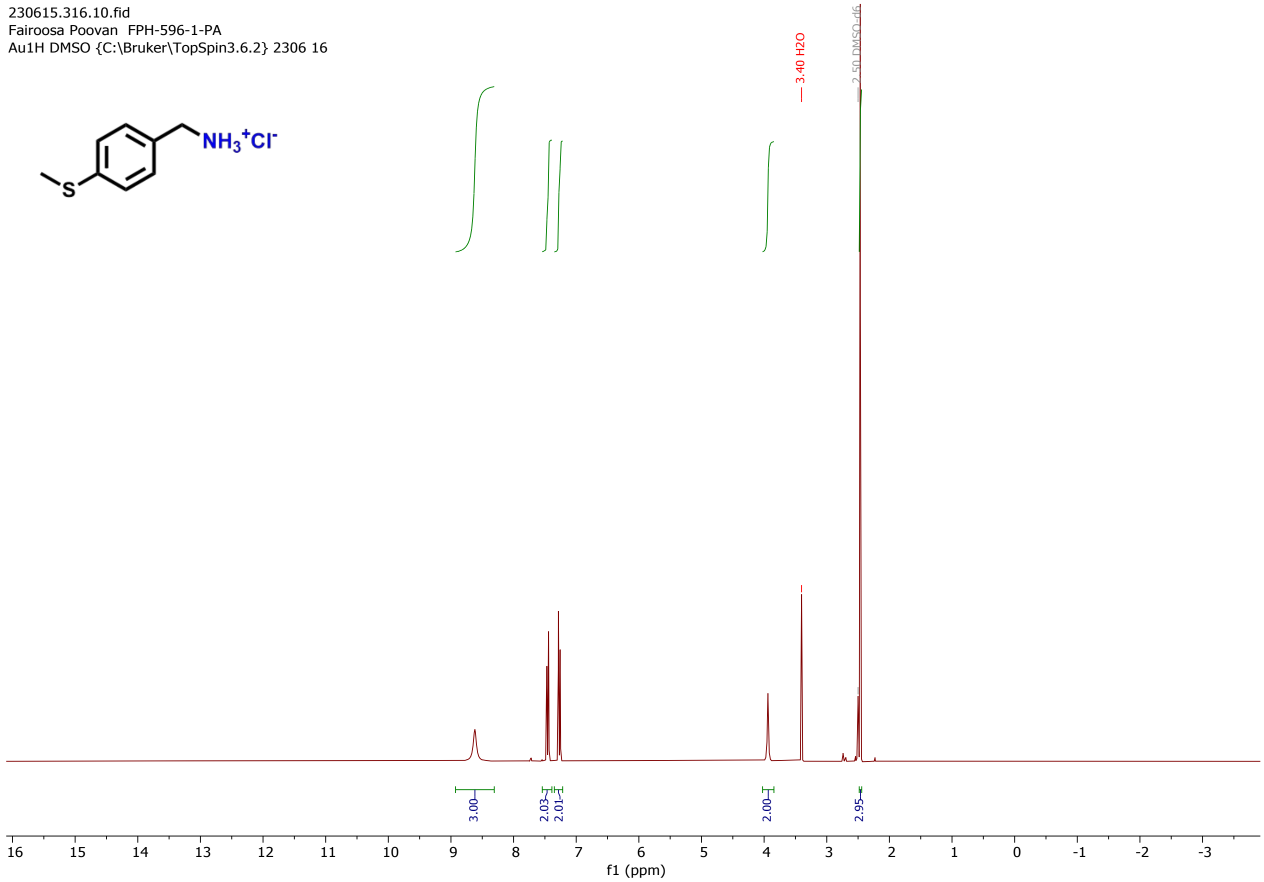

230615.316.11.fid
Fairoosa Poovan FPH-596-1-PA
Au13C DMSO {C:\Bruker\TopSpin3.6.2} 2306 16

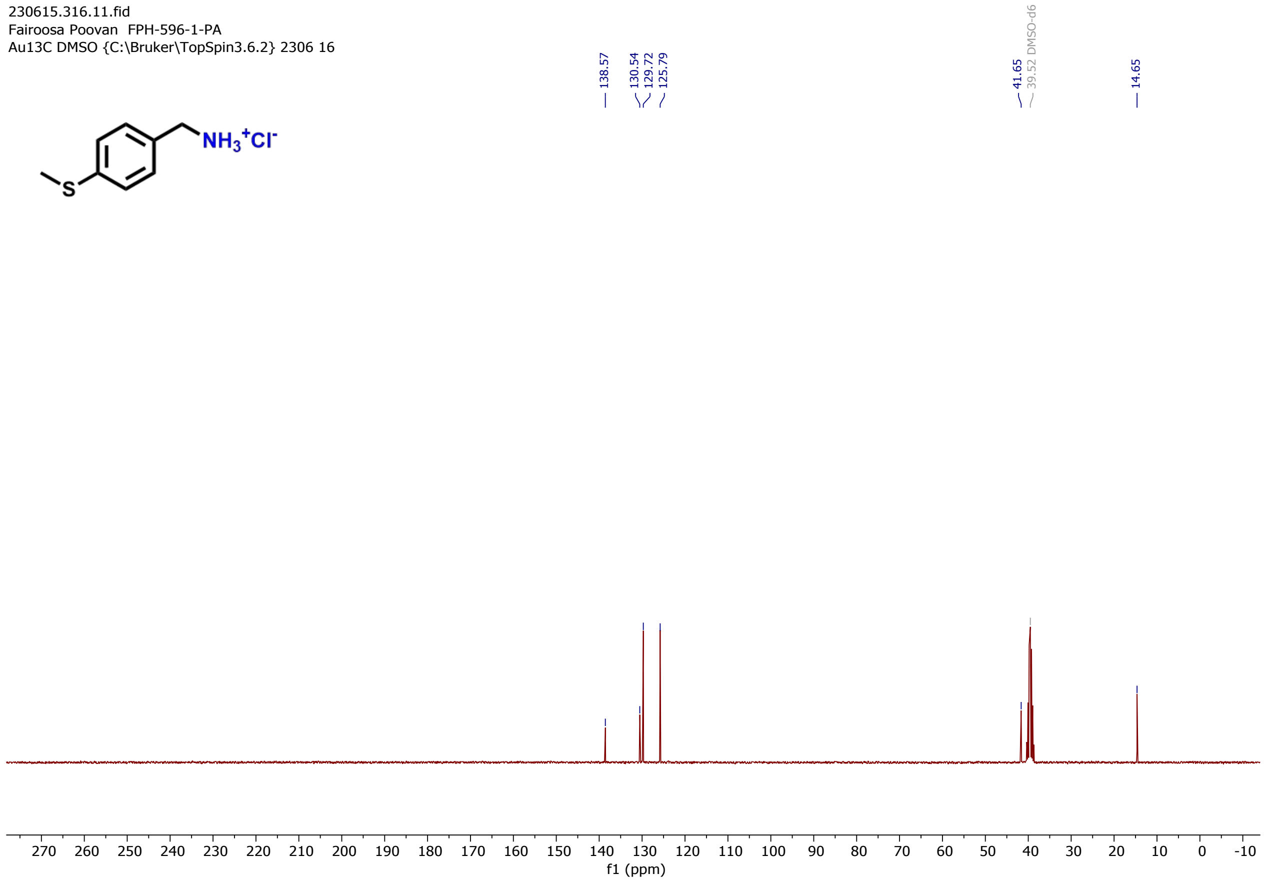

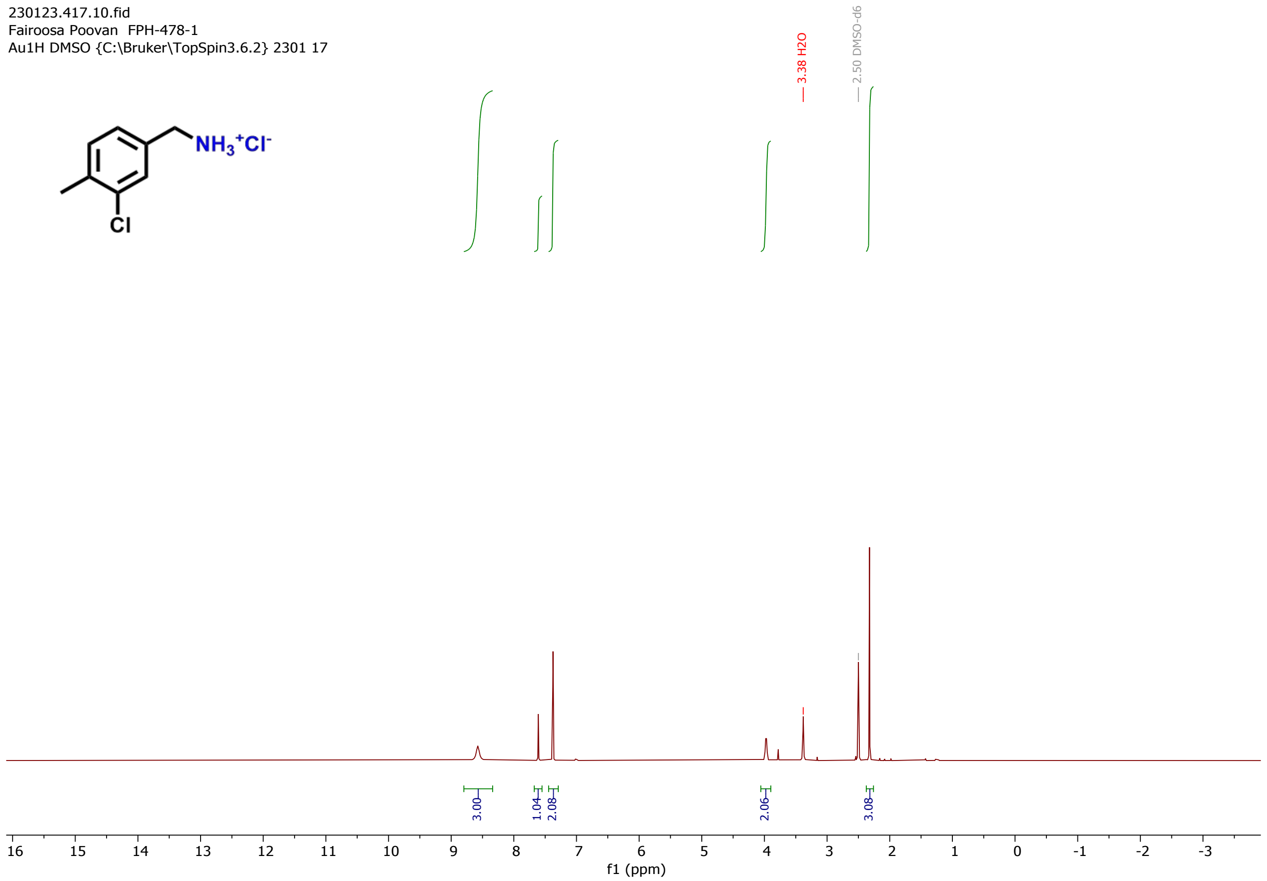
230123.417.10.fid
Fairoosa Poovan FPH-478-1
Au1H DMSO {C:\Bruker\TopSpin3.6.2} 2301 17
3.38 H2O
2.50 DMSO-d6
NH3+Cl-
Cl
3.00
1.04
2.08
2.06
3.08
f1 (ppm)

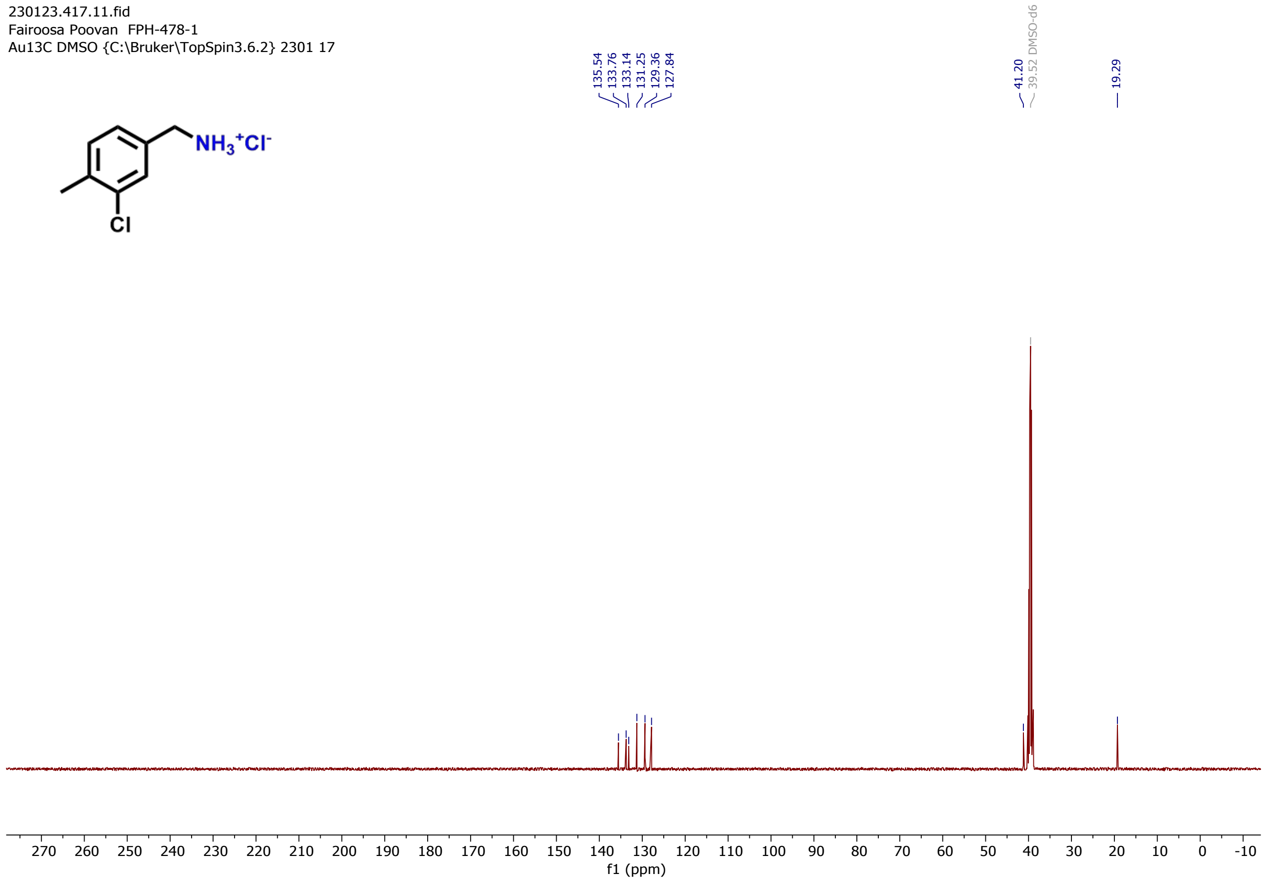
230123.417.11.fid
Fairoosa Poovan FPH-478-1
Au13C DMSO {C:\Bruker\TopSpin3.6.2} 2301 17
135.54
133.76
133.14
131.25
129.36
127.84
41.20
39.52 DMSO-d6
19.29
NH3+Cl-
Cl
f1 (ppm)

230615.314.10.fid
Fairoosa Poovan FPH-745-PA
Au1H DMSO {C:\Bruker\TopSpin3.6.2} 2306 14

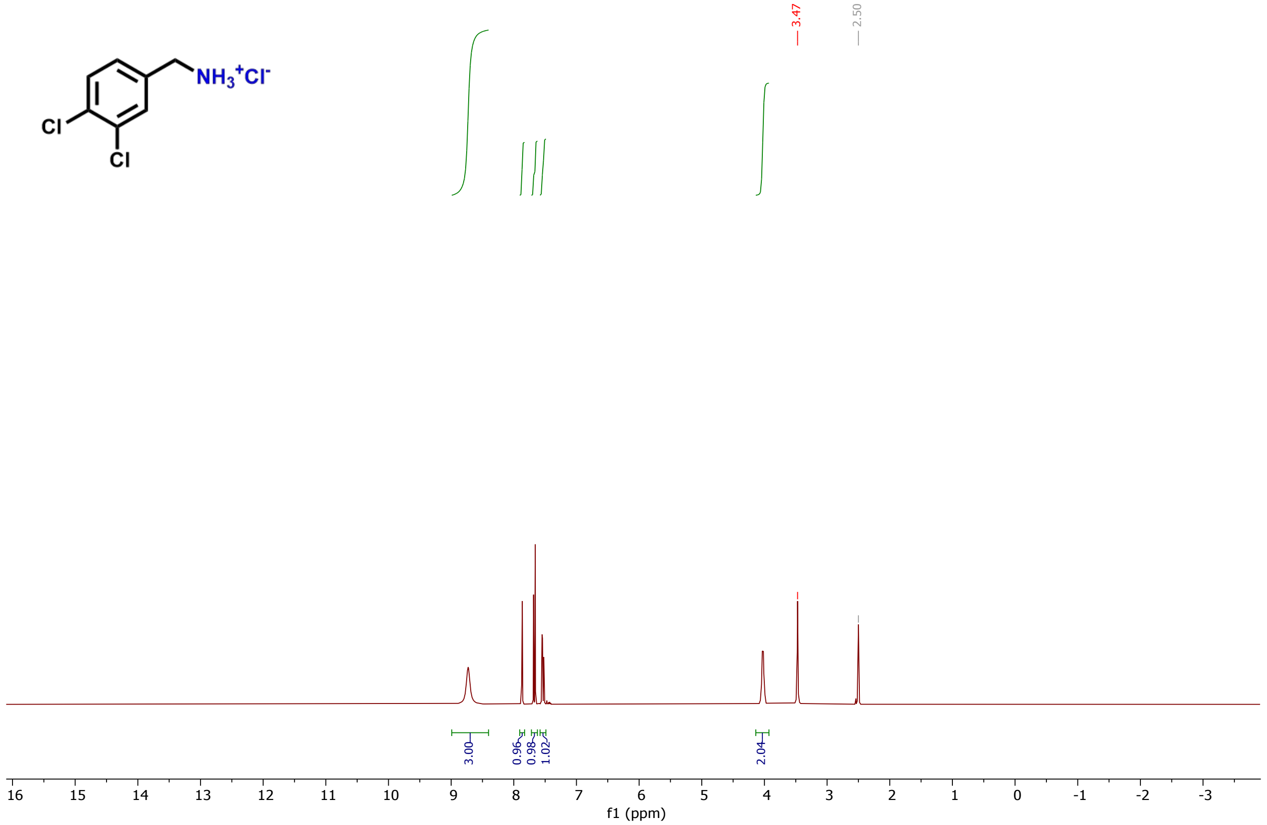

230615.314.11.fid
Fairoosa Poovan FPH-745-PA
Au13C DMSO {C:\Bruker\TopSpin3.6.2} 2306 14

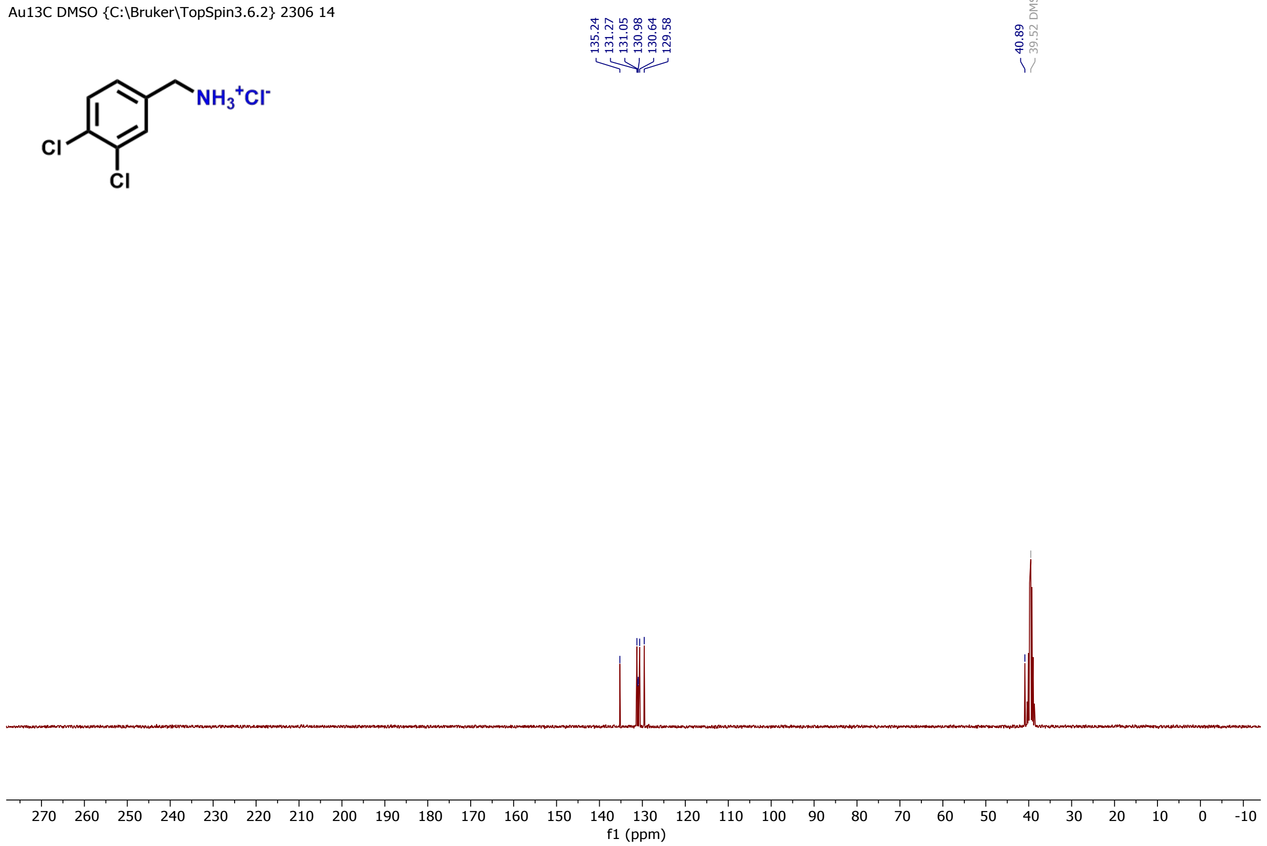

230703.310.12.fid
Fairoosa Poovan FPH-599-PA Wdhl.
Au1H DMSO {C:\Bruker\TopSpin3.6.2} 2307 12

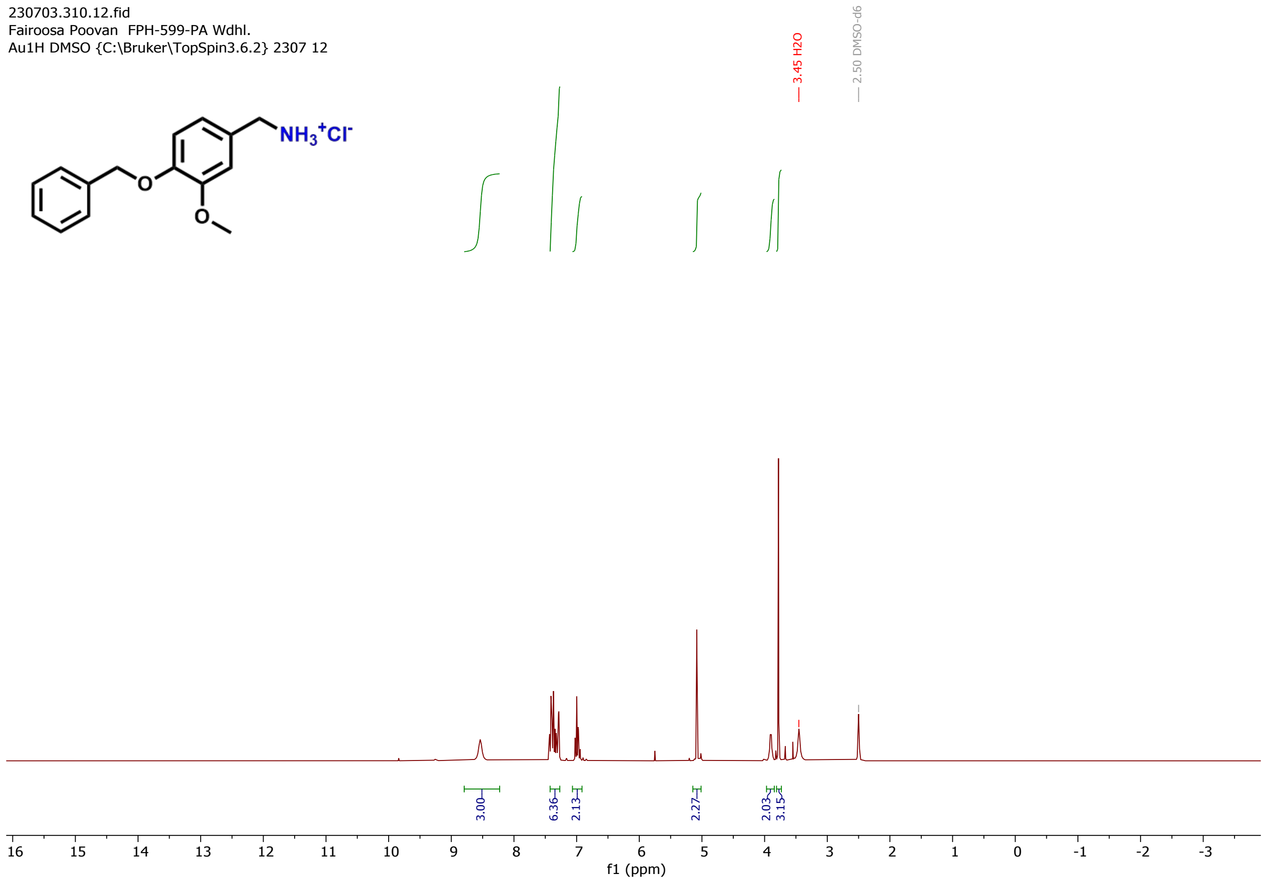

230703.310.13.fid
Fairoosa Poovan FPH-599-PA Wdhl.
Au13C DMSO {C:\Bruker\TopSpin3.6.2} 2307 12

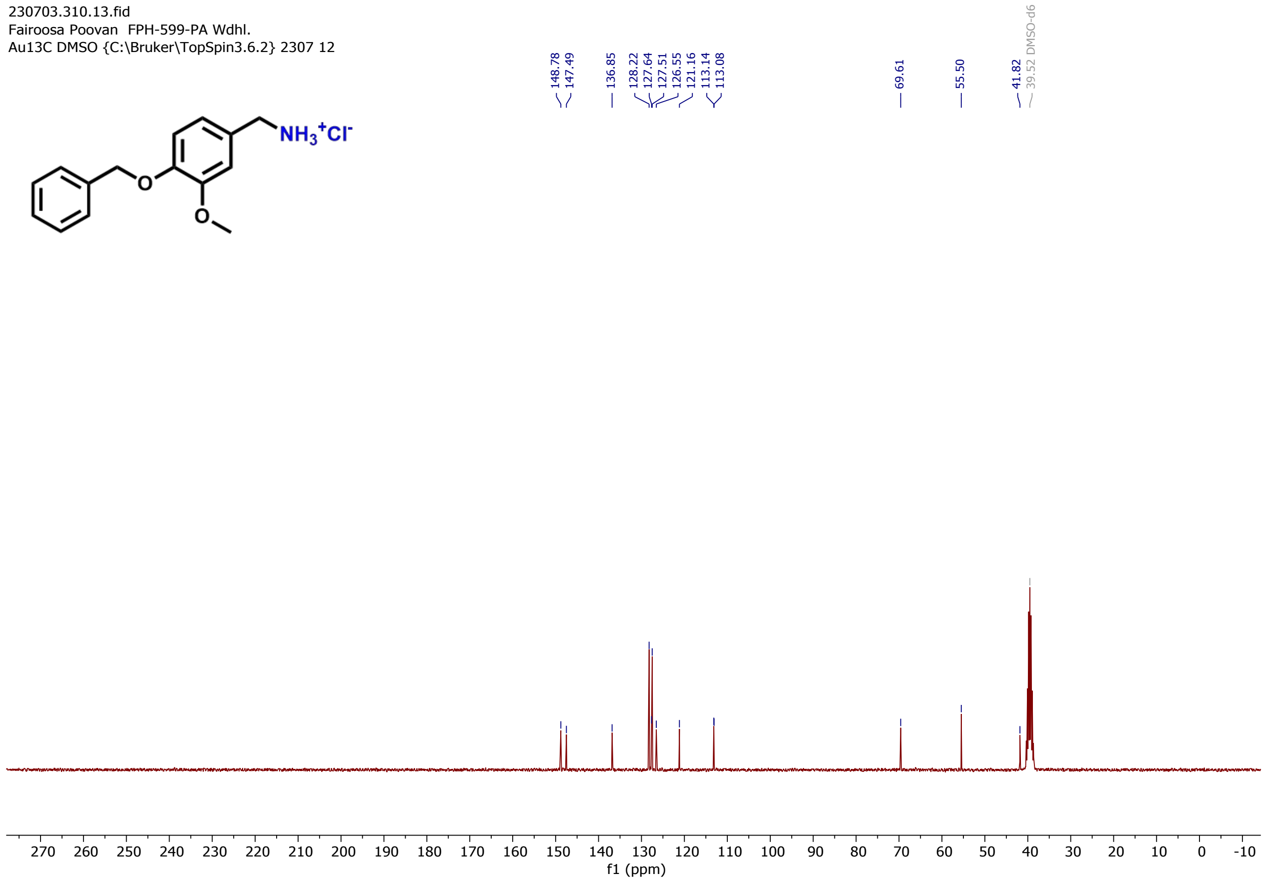

230526.f319.10.fid
Fairoosa Poovan FPH-736-(PA)-N
PROTON DMSO {C:\Bruker\TopSpin3.6.2} 2305 19

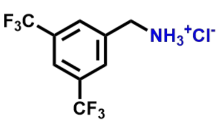

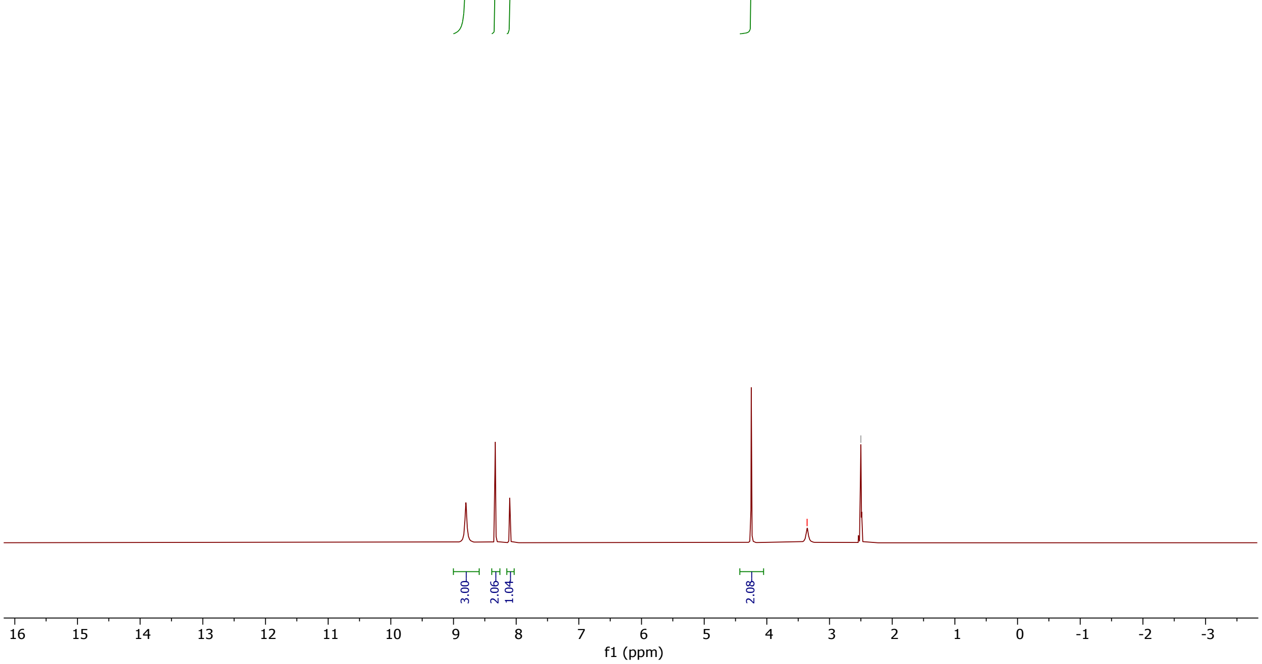

230615.312.10.fid
Fairoosa Poovan FPH-736-PA
Au13C DMSO {C:\Bruker\TopSpin3.6.2} 2306 12

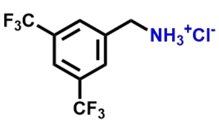

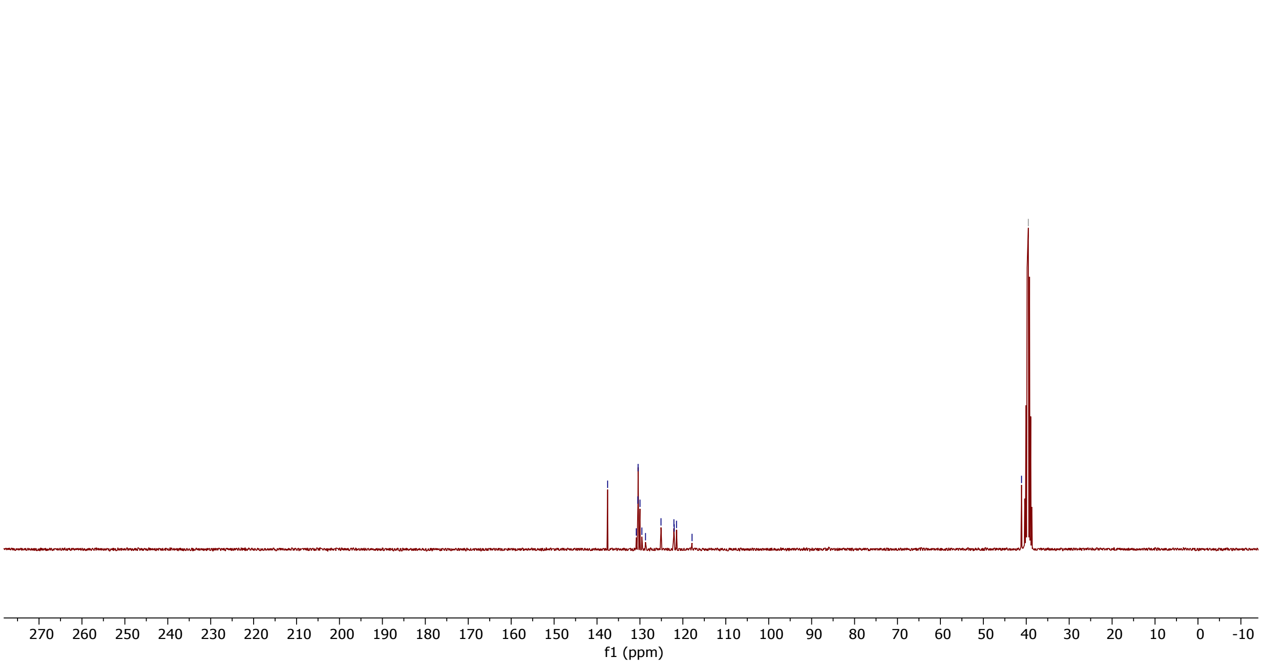

230724.f351.10.fid
Poovan
FPH-346-PA
PROTON D2O {C:\Bruker\TopSpin3.6.2} 2307 51

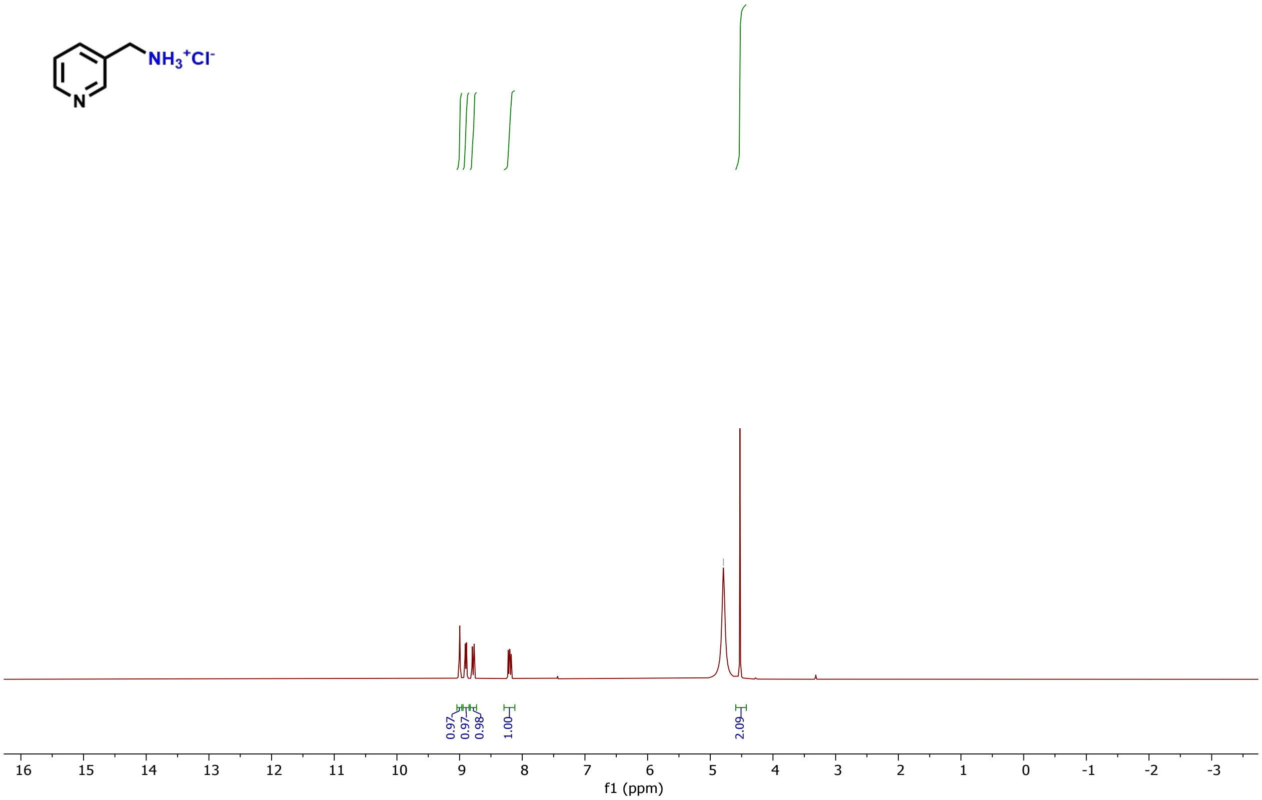

230724.f351.11.fid
Poovan
FPH-346-PA
C13CPD D2O {C:\Bruker\TopSpin3.6.2} 2307 51

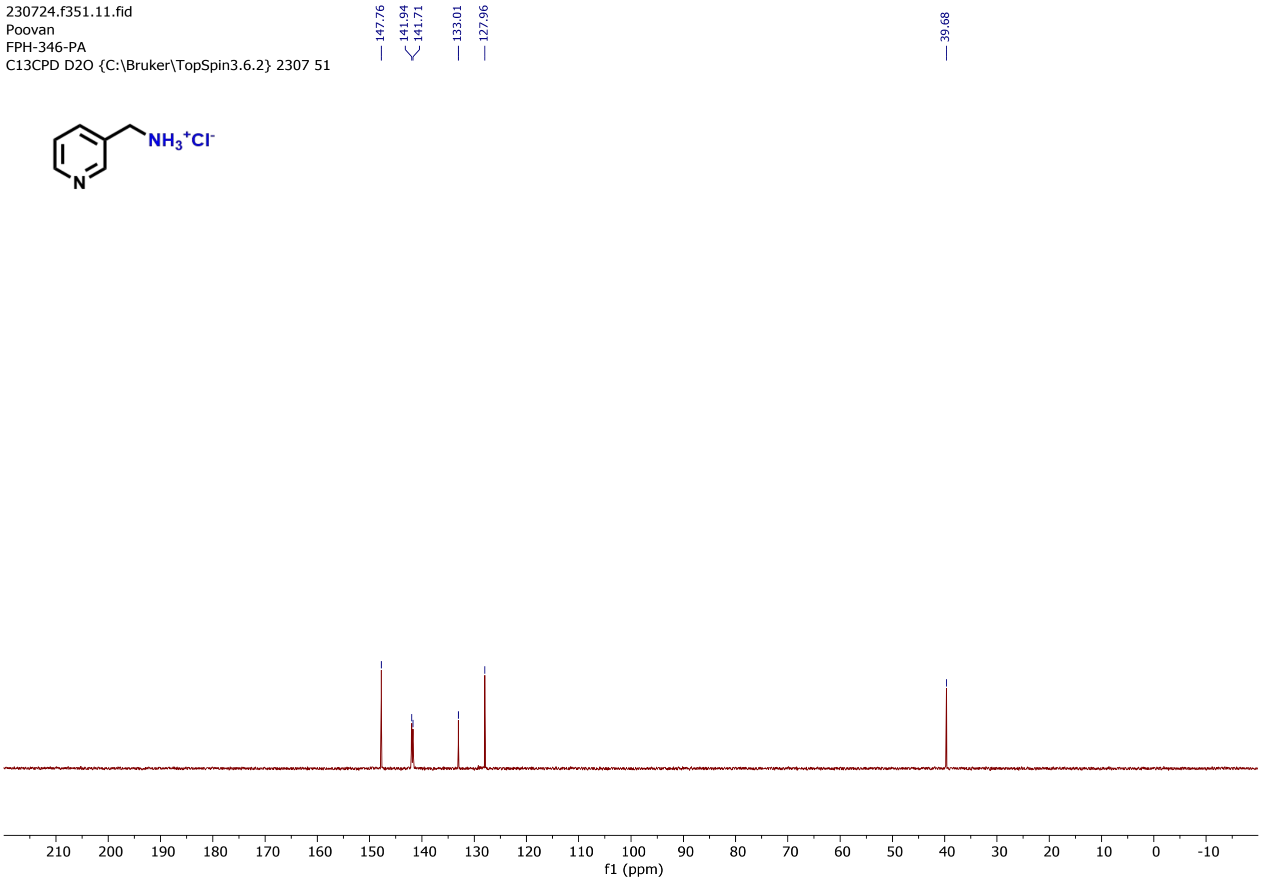

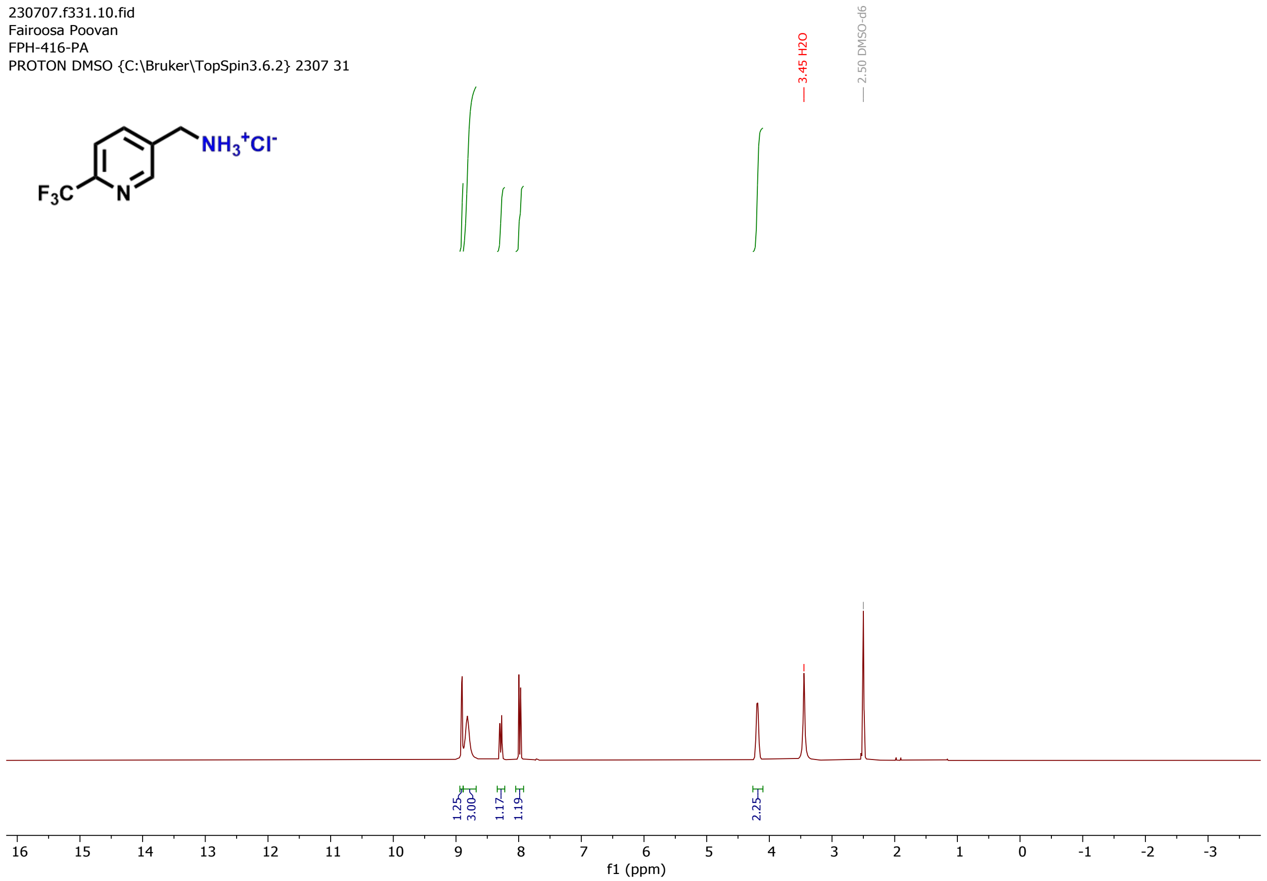
230707.f331.10.fid
Fairoosa Poovan
FPH-416-PA
PROTON DMSO {C:\Bruker\TopSpin3.6.2} 2307 31
NH3+Cl-
F3C
N
3.45 H2O
2.50 DMSO-d6
1.25
3.00
1.17
1.19
2.25
f1 (ppm)

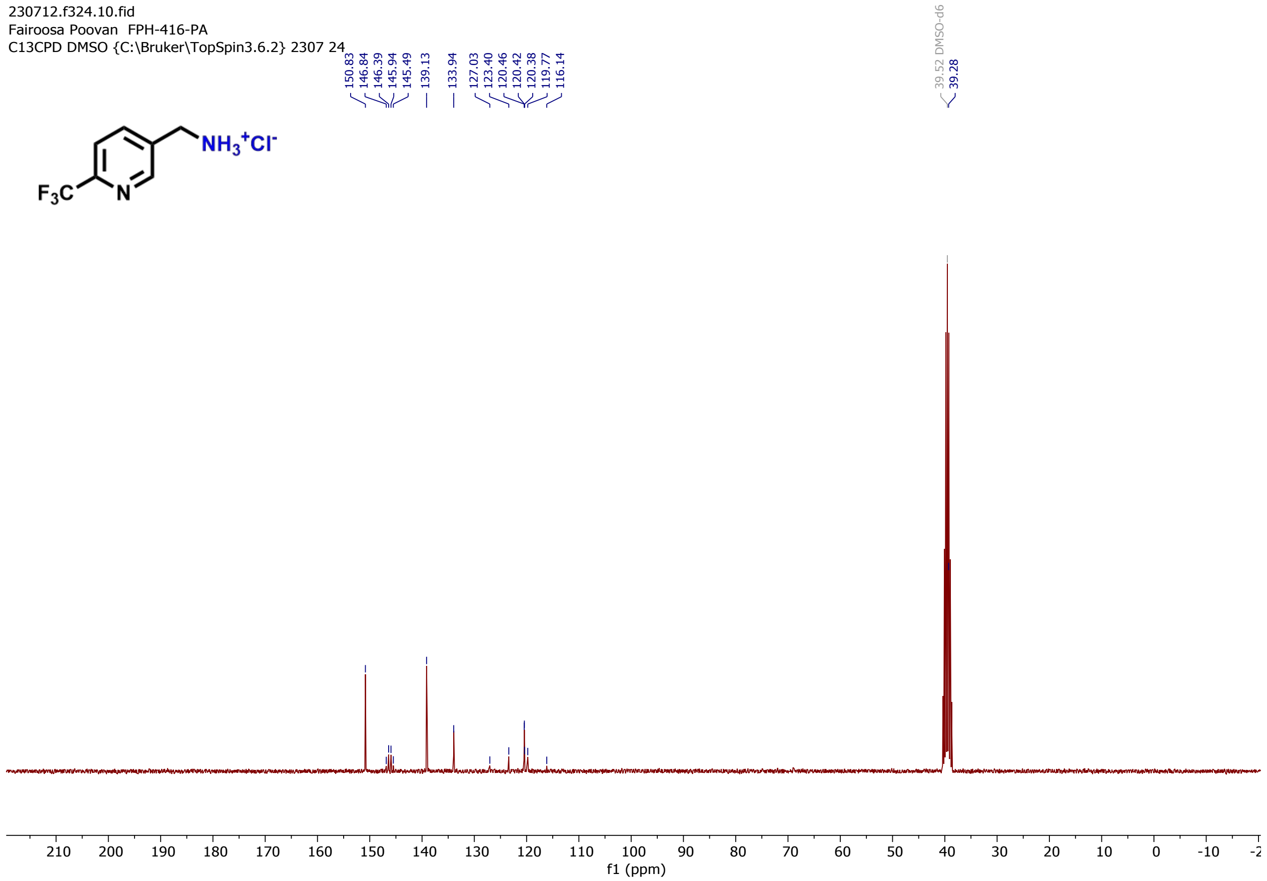
230712.f324.10.fid
Fairoosa Poovan FPH-416-PA
C13CPD DMSO {C:\Bruker\TopSpin3.6.2} 2307 24
NH3+Cl-
F3C
N
150.83
146.84
146.39
145.94
145.49
139.13
133.94
127.03
123.40
120.46
120.42
120.38
119.77
116.14
39.52 DMSO-d6
39.28
f1 (ppm)

230615.315.10.fid
Fairoosa Poovan FPH-477-PA
Au1H DMSO {C:\Bruker\TopSpin3.6.2} 2306 15

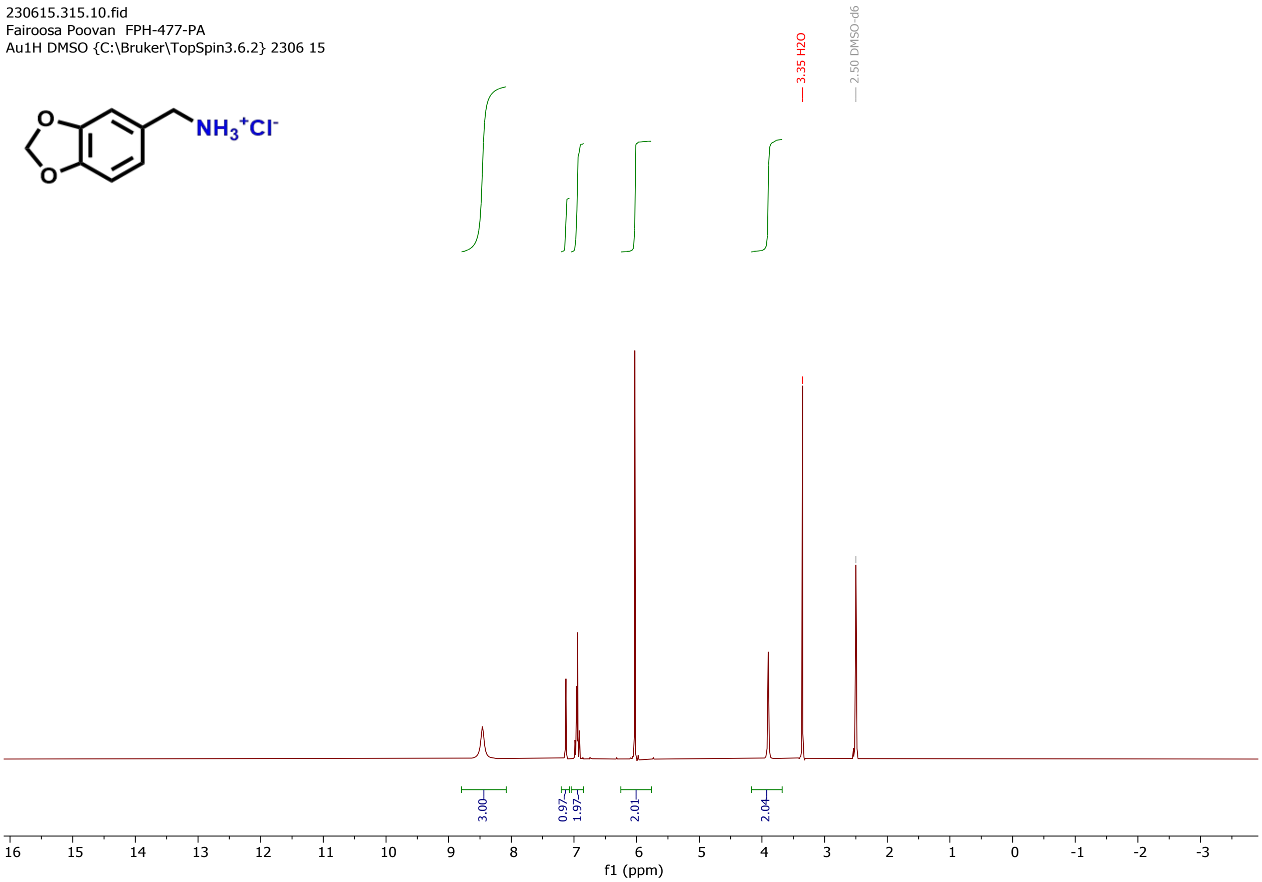

230615.315.11.fid
Fairoosa Poovan FPH-477-PA
Au13C DMSO {C:\Bruker\TopSpin3.6.2} 2306 15

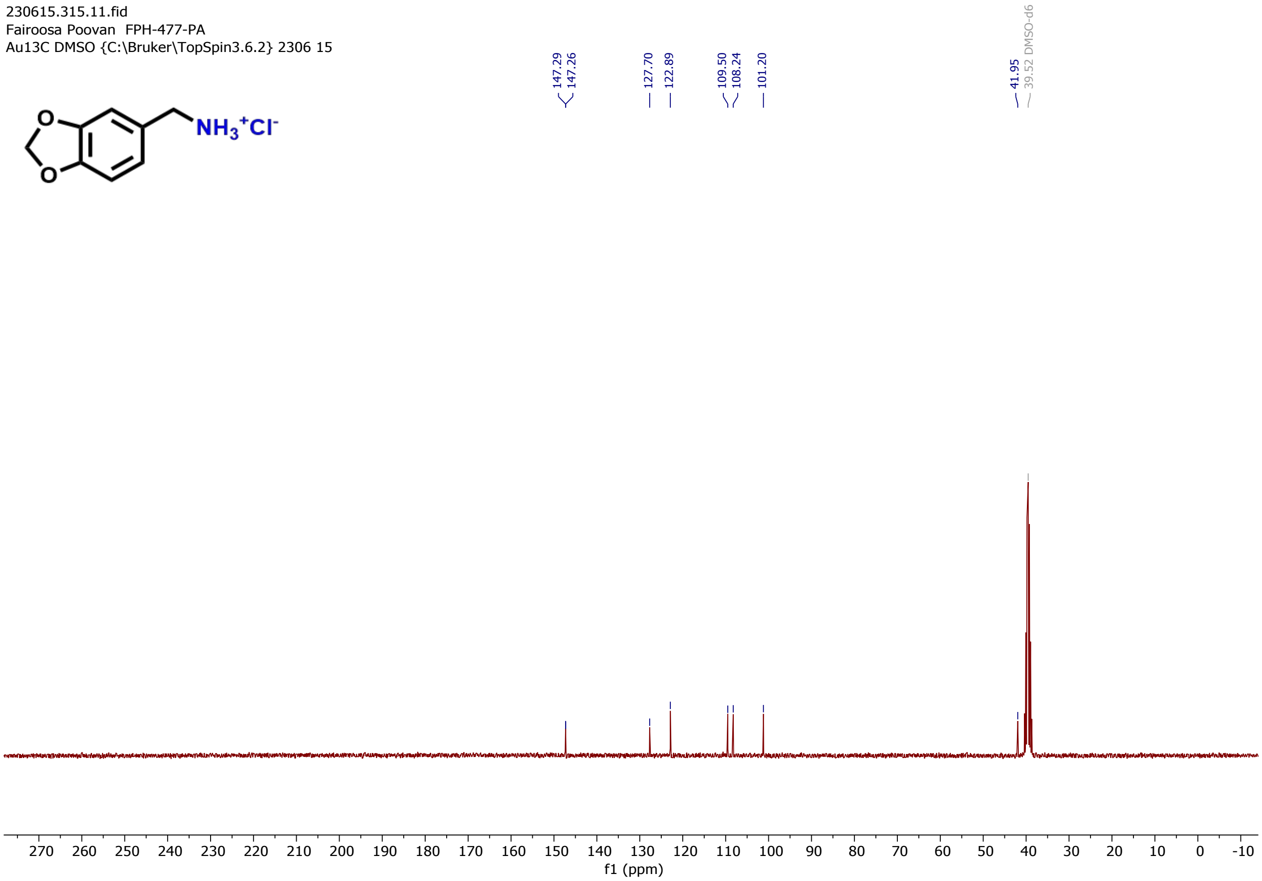

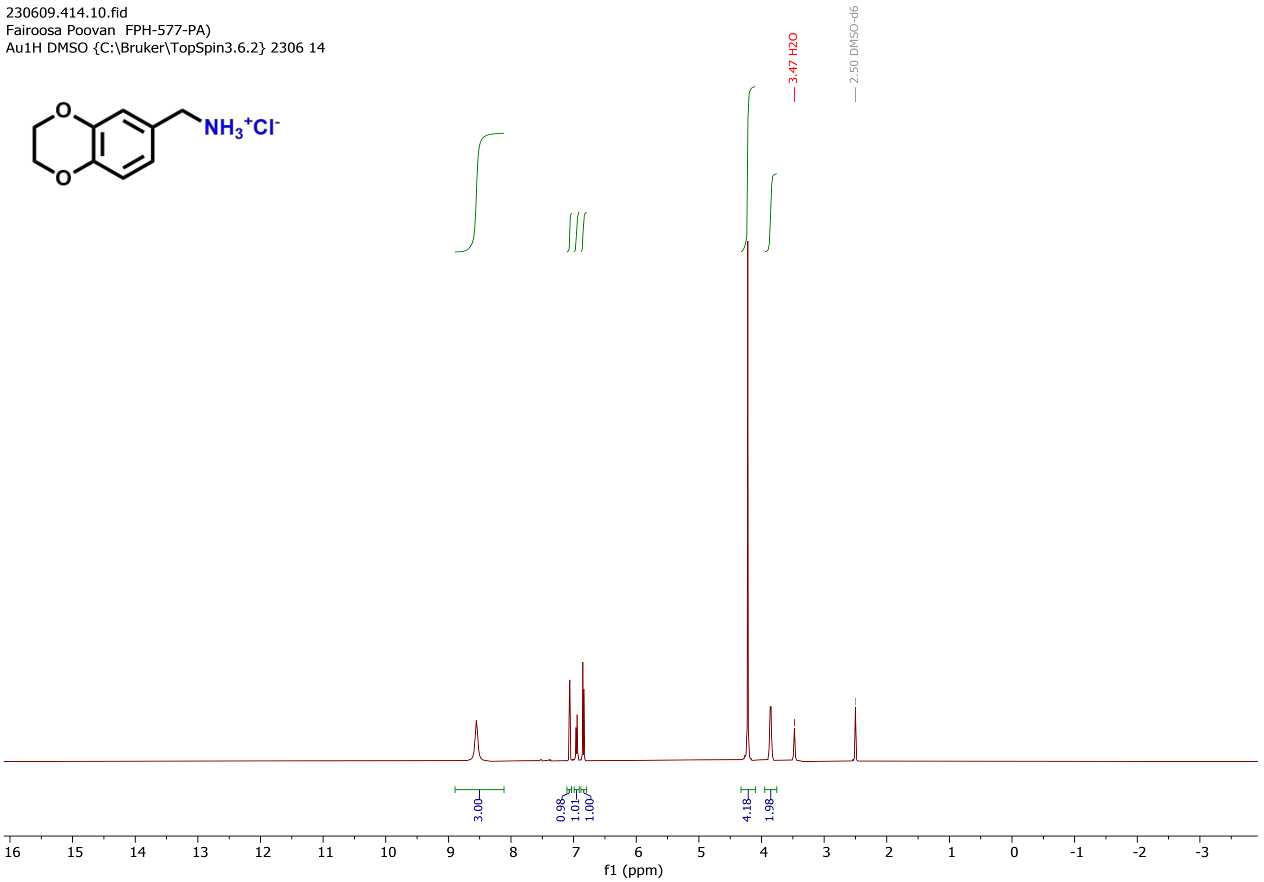
230609.414.10.fid
Fairoosa Poovan FPH-577-PA)
Au1H DMSO {C:\Bruker\TopSpin3.6.2} 2306 14
NH3+Cl-
3.47 H2O
2.50 DMSO-d6
3.00
0.98
1.01
1.00
4.18
1.98
f1 (ppm)

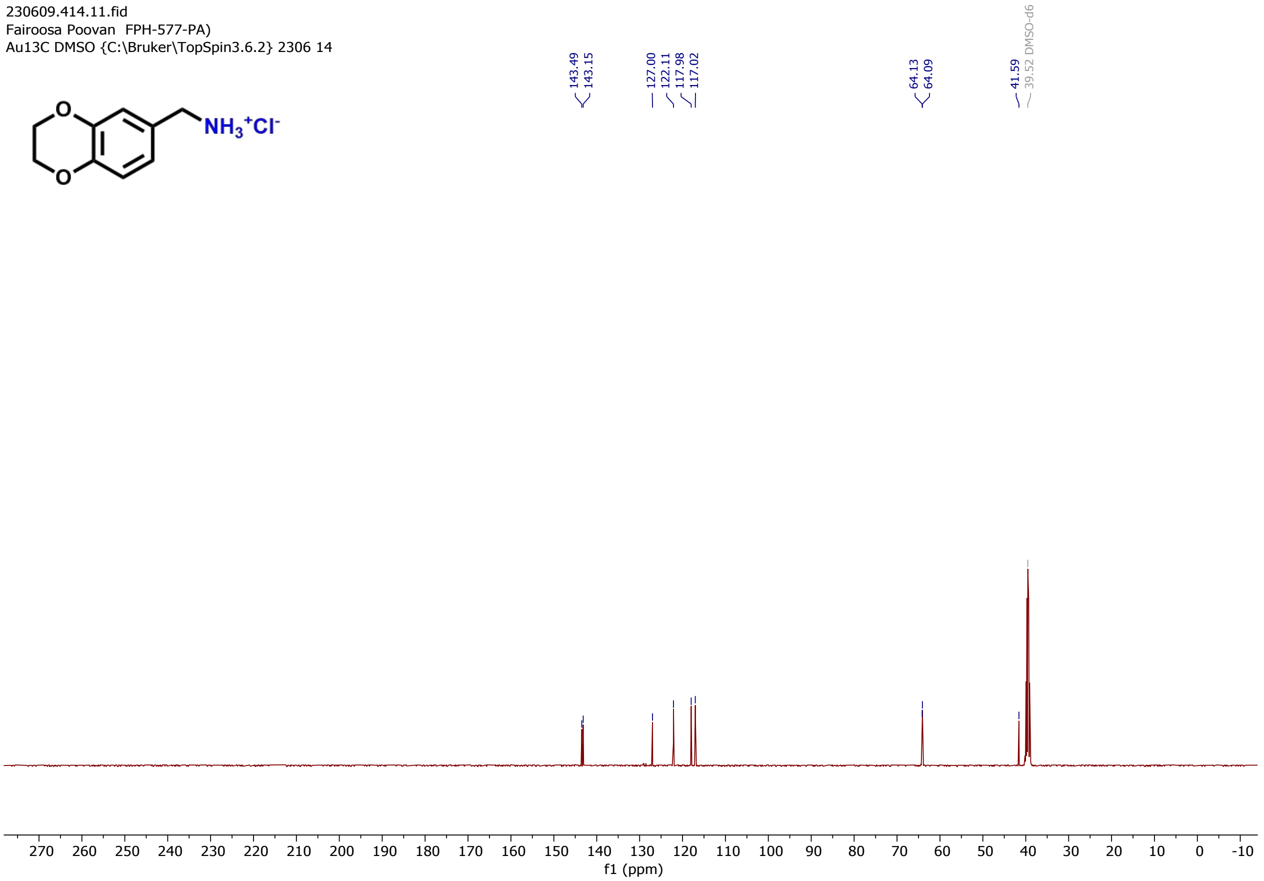
230609.414.11.fid
Fairoosa Poovan FPH-577-PA)
Au13C DMSO {C:\Bruker\TopSpin3.6.2} 2306 14
NH3+Cl-
143.49
143.15
127.00
122.11
117.98
117.02
64.13
64.09
41.59
39.52 DMSO-d6
f1 (ppm)

230606.308.10.fid
Fairoosa Poovan FPH-557
Au1H DMSO {C:\Bruker\TopSpin3.6.2} 2306 8

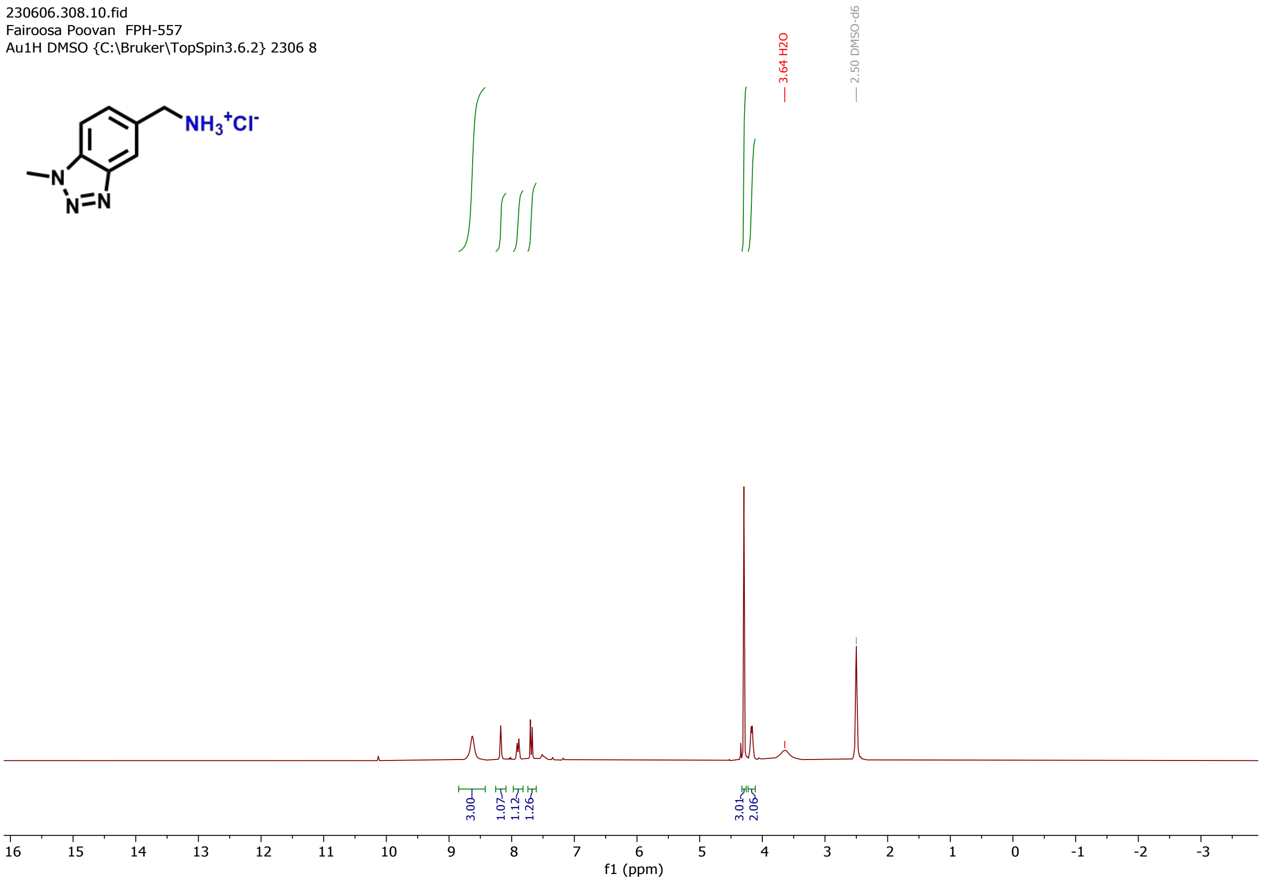

230606.308.11.fid
Fairoosa Poovan FPH-557
Au13C DMSO {C:\Bruker\TopSpin3.6.2} 2306 8

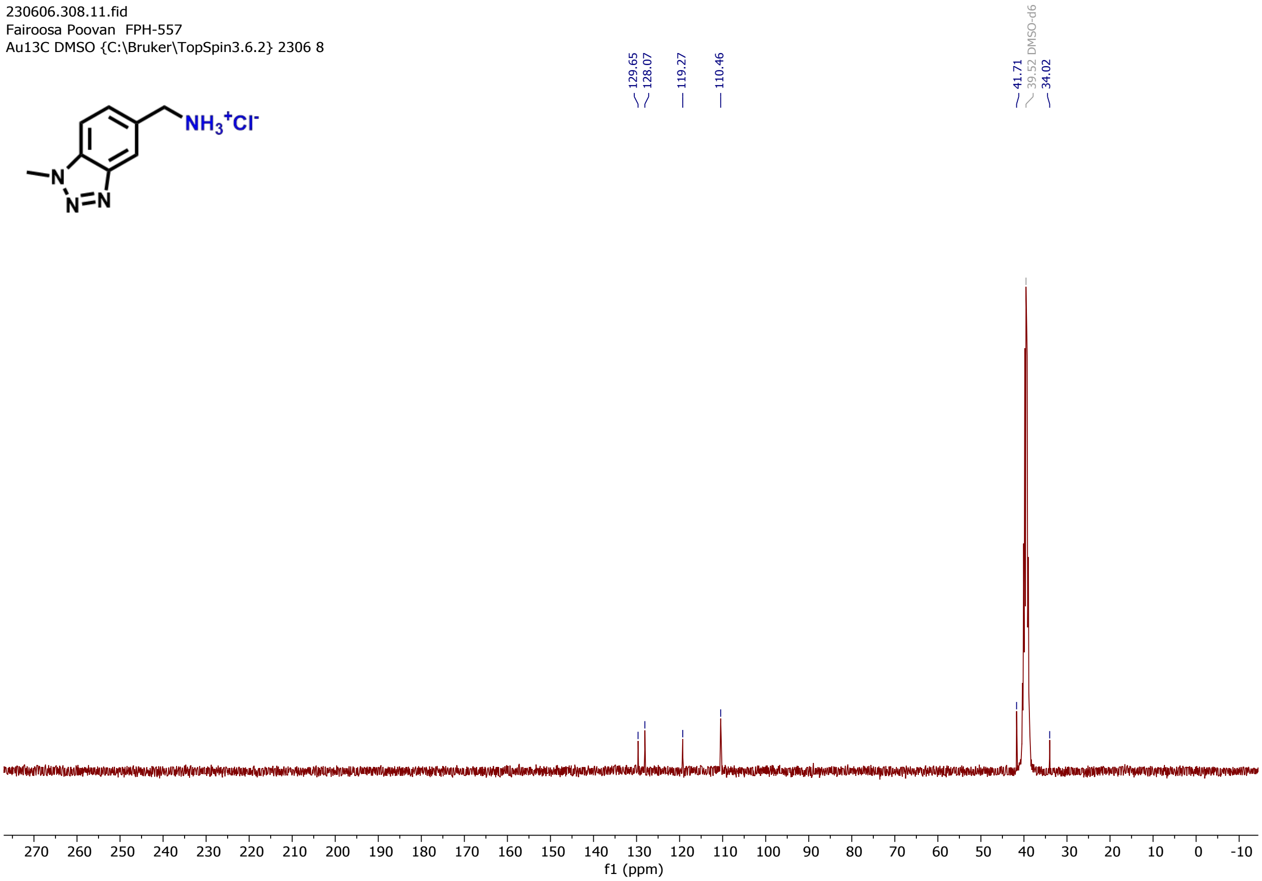

230606.309.10.fid
Fairoosa Poovan FPH-417-1
Au1H DMSO {C:\Bruker\TopSpin3.6.2} 2306 9

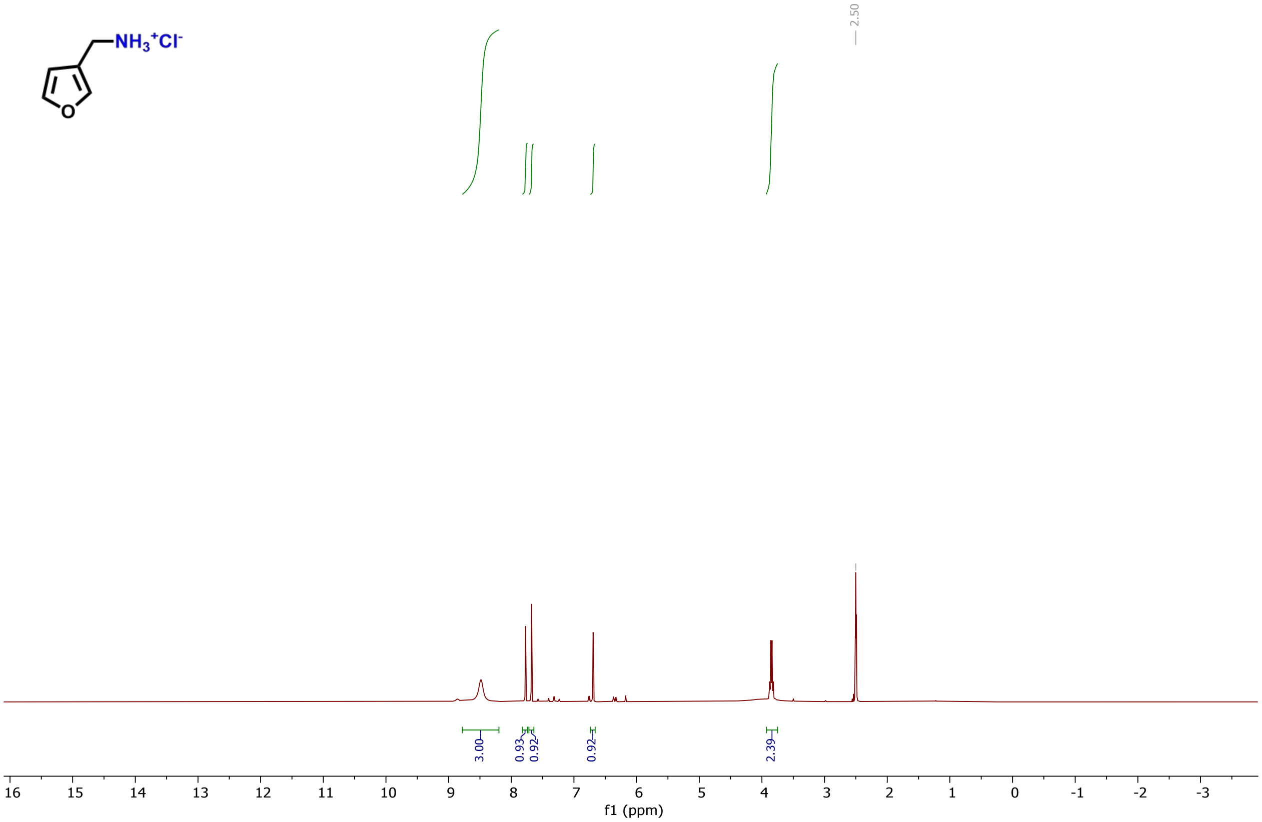

230606.309.11.fid
Fairoosa Poovan FPH-417-1
Au13C DMSO {C:\Bruker\TopSpin3.6.2} 2306 9

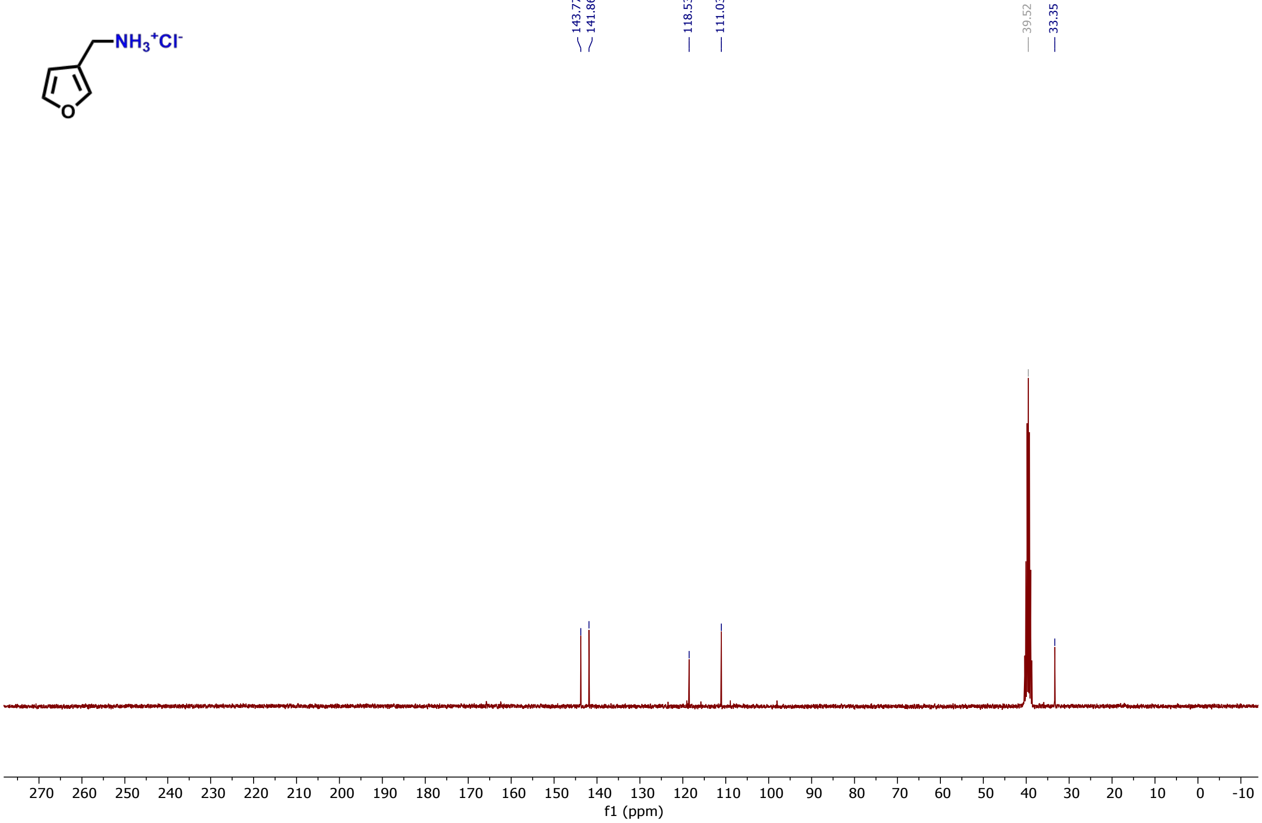

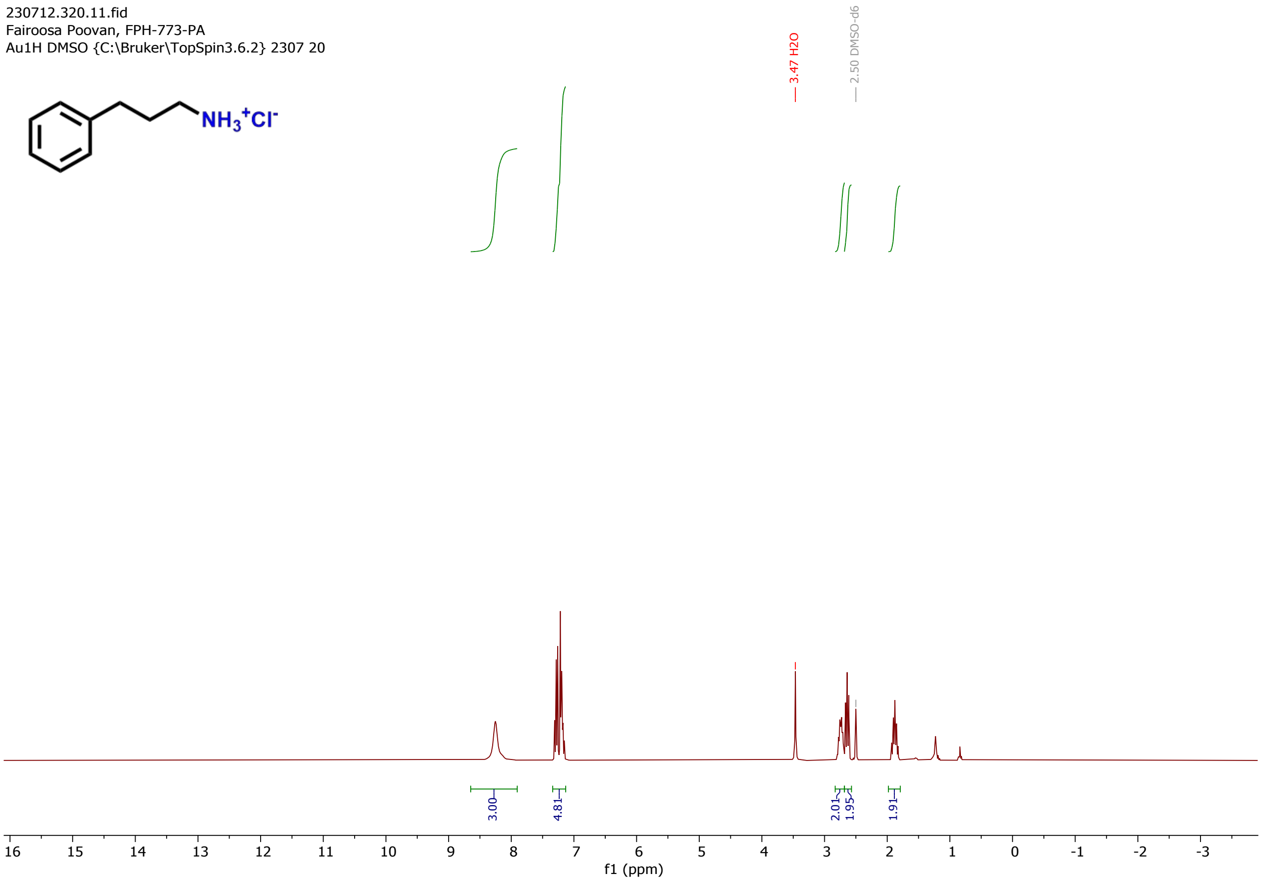
230712.320.11.fid
Fairoosa Poovan, FPH-773-PA
Au1H DMSO {C:\Bruker\TopSpin3.6.2} 2307 20
NH3+Cl-
3.47 H2O
2.50 DMSO-d6
3.00
4.81
2.01
1.95
1.91
f1 (ppm)

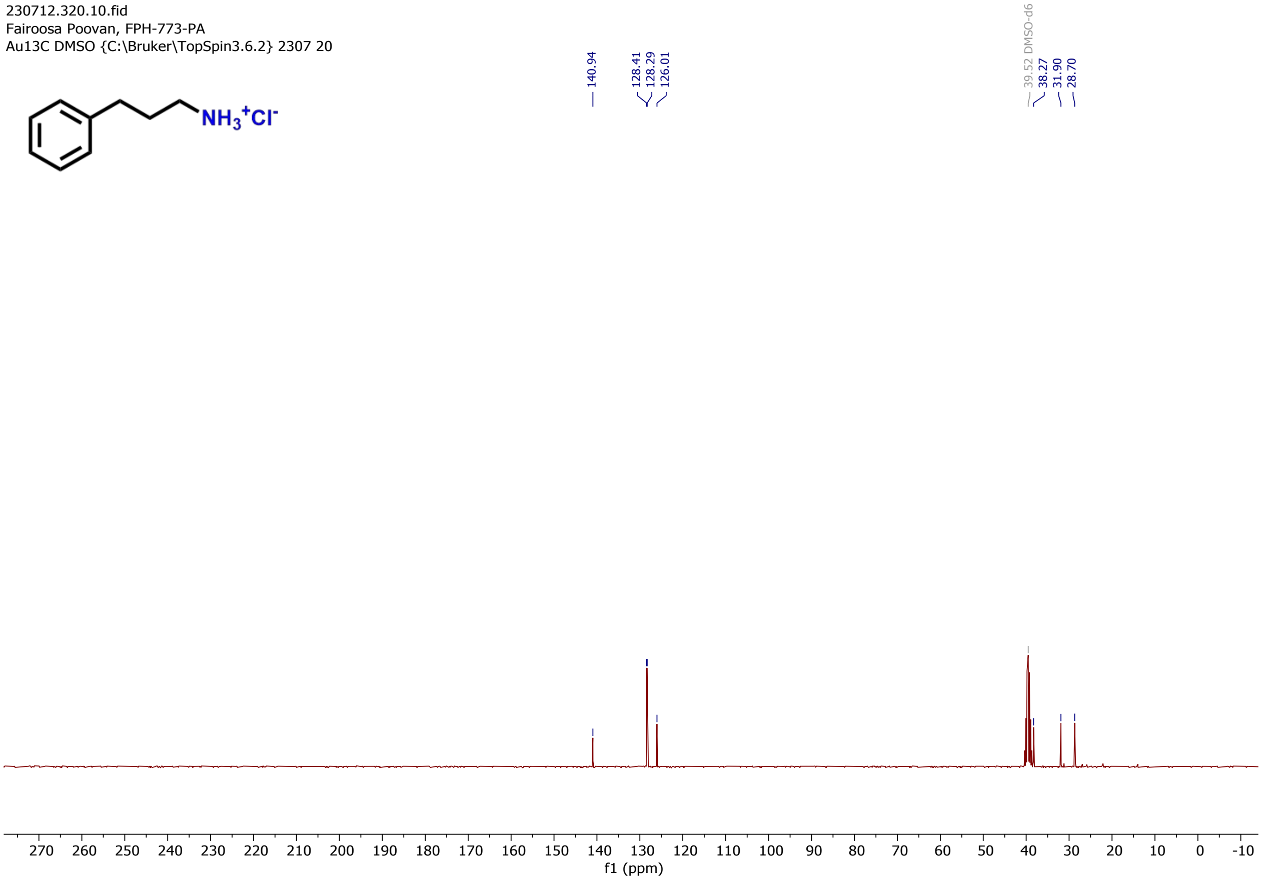
230712.320.10.fid
Fairoosa Poovan, FPH-773-PA
Au13C DMSO {C:\Bruker\TopSpin3.6.2} 2307 20
NH3+Cl-
140.94
128.41
128.29
126.01
39.52 DMSO-d6
38.27
31.90
28.70
f1 (ppm)

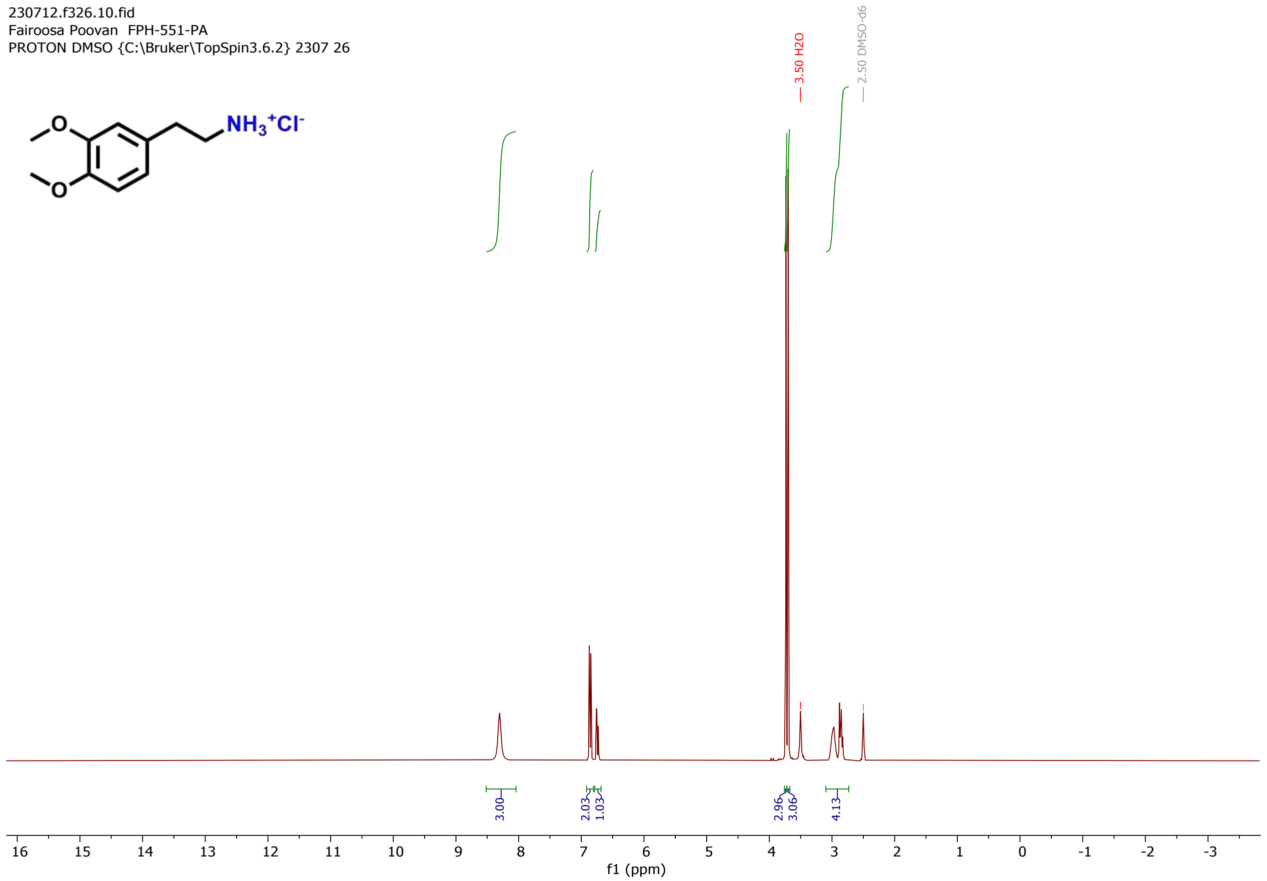
230712.f326.10.fid
Fairoosa Poovan FPH-551-PA
PROTON DMSO {C:\Bruker\TopSpin3.6.2} 2307 26
NH3+Cl-
3.50 H2O
2.50 DMSO-d6
3.00
2.03
1.03
2.96
3.06
4.13
16 15 14 13 12 11 10 9 8 7 6 5 4 3 2 1 0 -1 -2 -3
f1 (ppm)

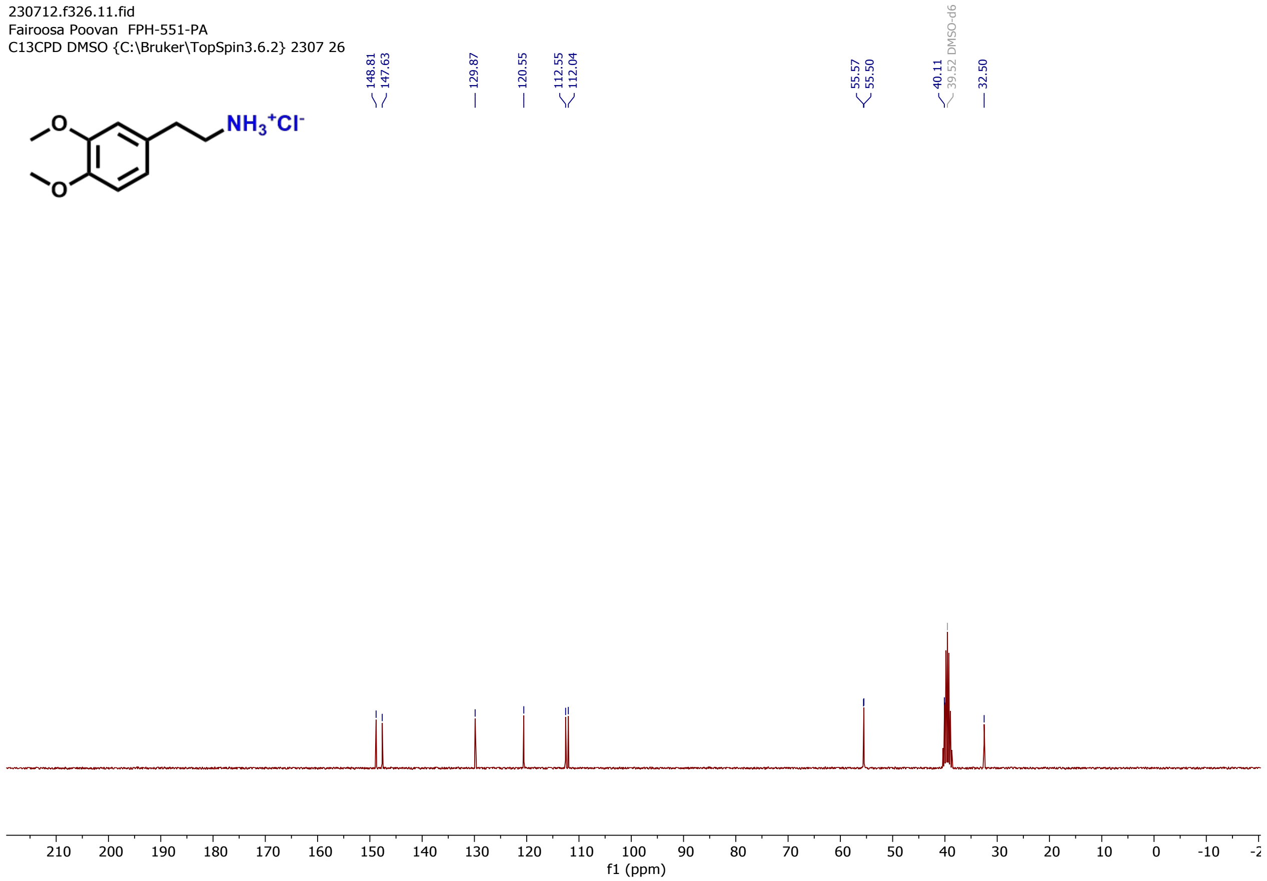
230712.f326.11.fid
Fairoosa Poovan FPH-551-PA
C13CPD DMSO {C:\Bruker\TopSpin3.6.2} 2307 26
NH3+Cl-
148.81
147.63
129.87
120.55
112.55
112.04
55.57
55.50
40.11
39.52 DMSO-d6
32.50
210 200 190 180 170 160 150 140 130 120 110 100 90 80 70 60 50 40 30 20 10 0 -10
f1 (ppm)

230706.309.10.fid
Fairoosa Poovan FPH-640-PA
Au1H DMSO {C:\Bruker\TopSpin3.6.2} 2307 9

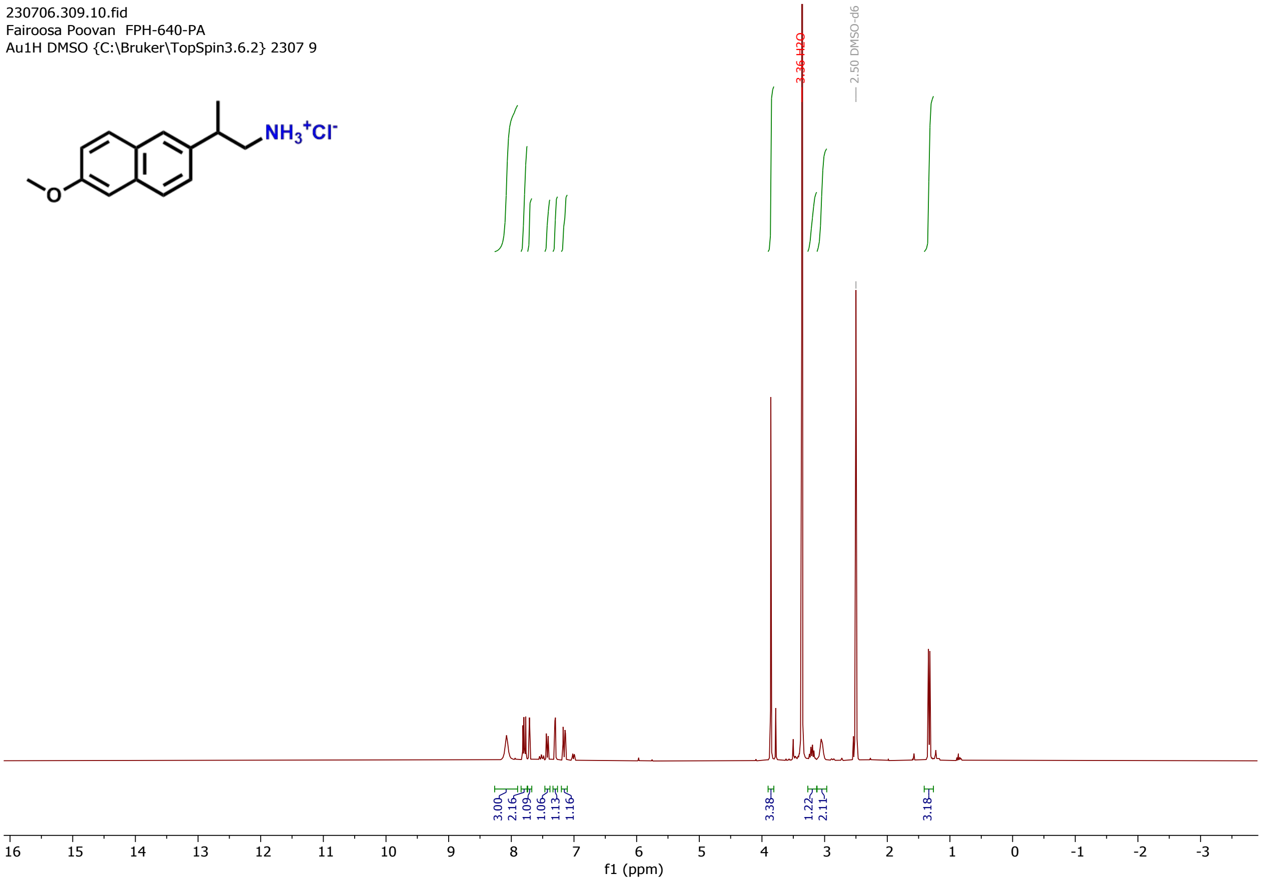

230706.309.11.fid
Fairoosa Poovan FPH-640-PA
Au13C DMSO {C:\Bruker\TopSpin3.6.2} 2307 9

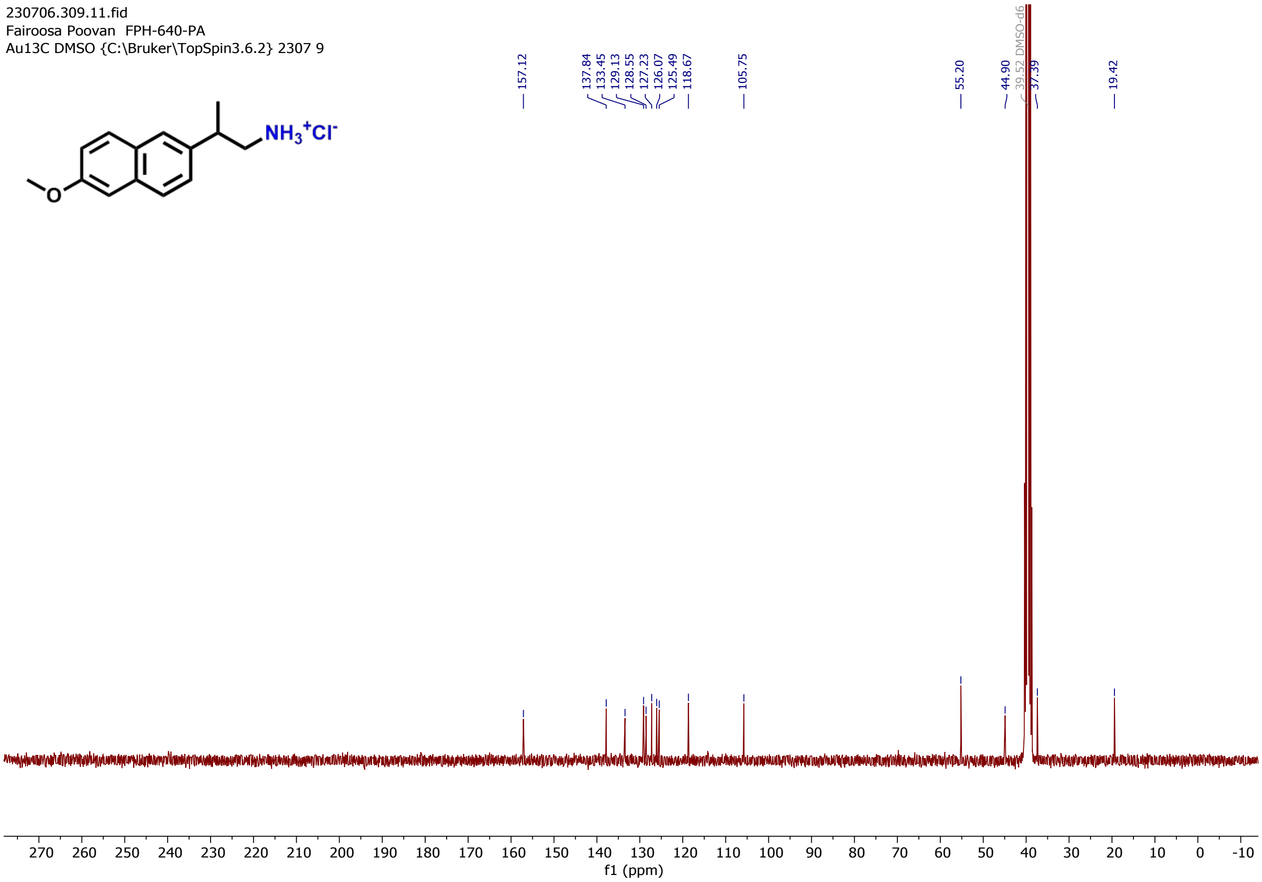

230623.321.10.fid
Fairoosa Poovan FPH-751-PA
Au1H DMSO {C:\Bruker\TopSpin3.6.2} 2306 21

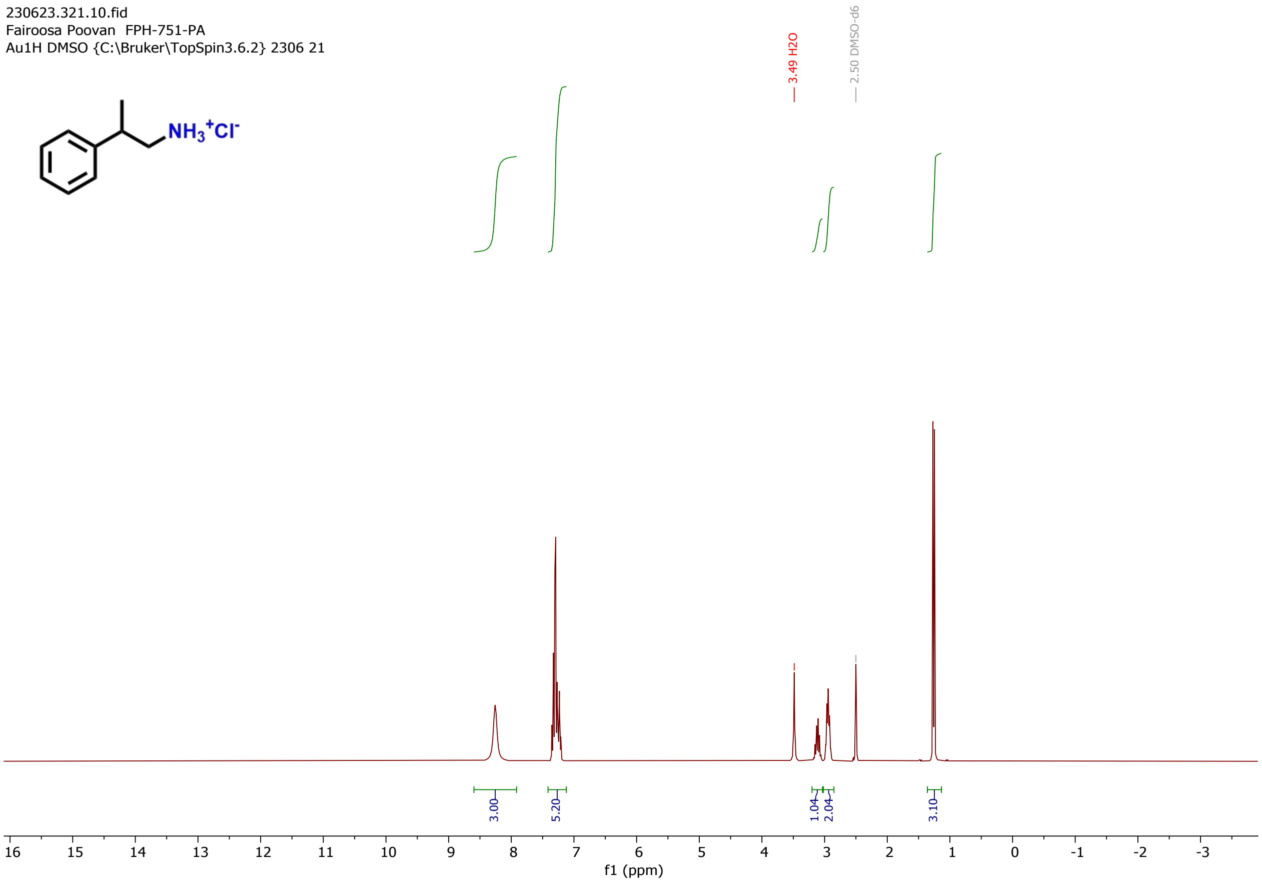

230623.321.11.fid
Fairoosa Poovan FPH-751-PA
Au13C DMSO {C:\Bruker\TopSpin3.6.2} 2306 21

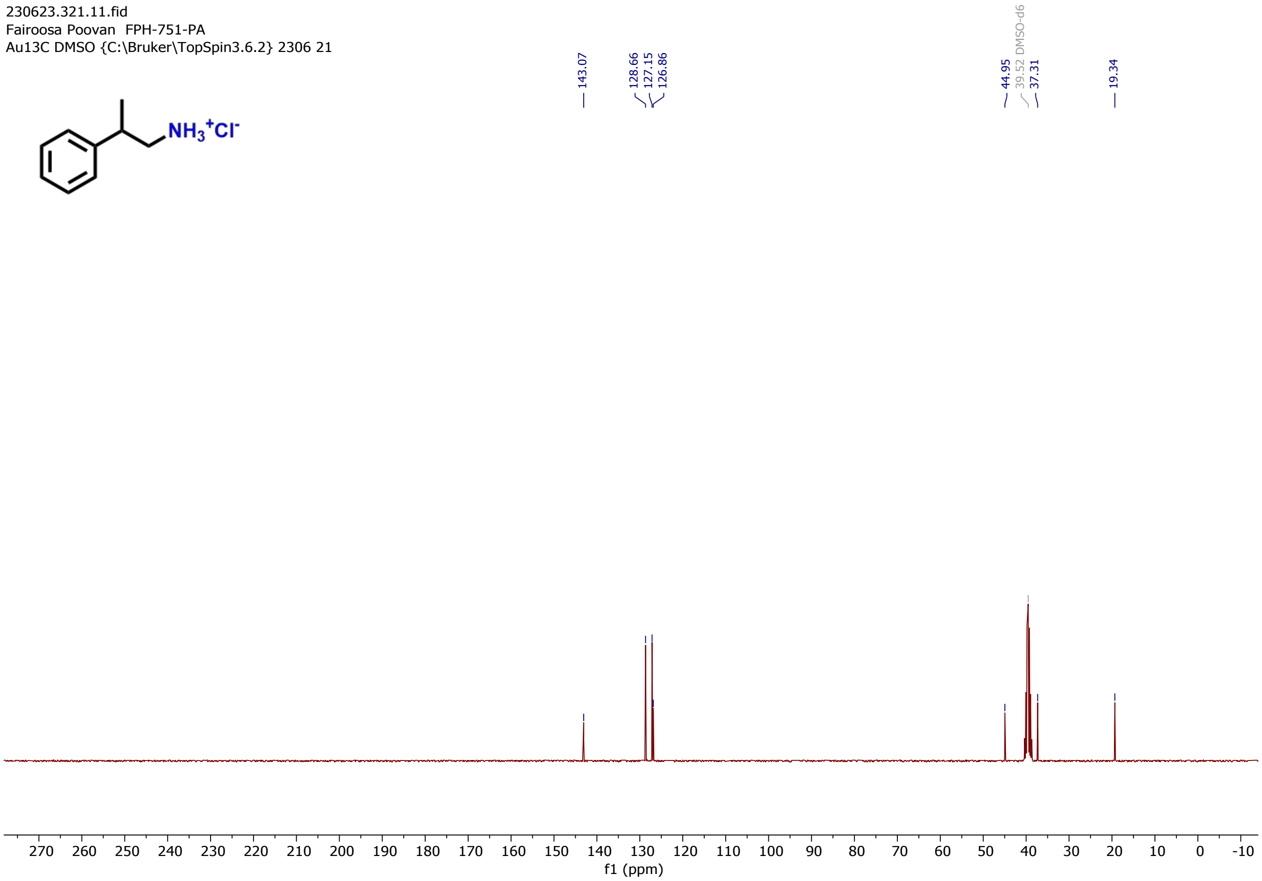

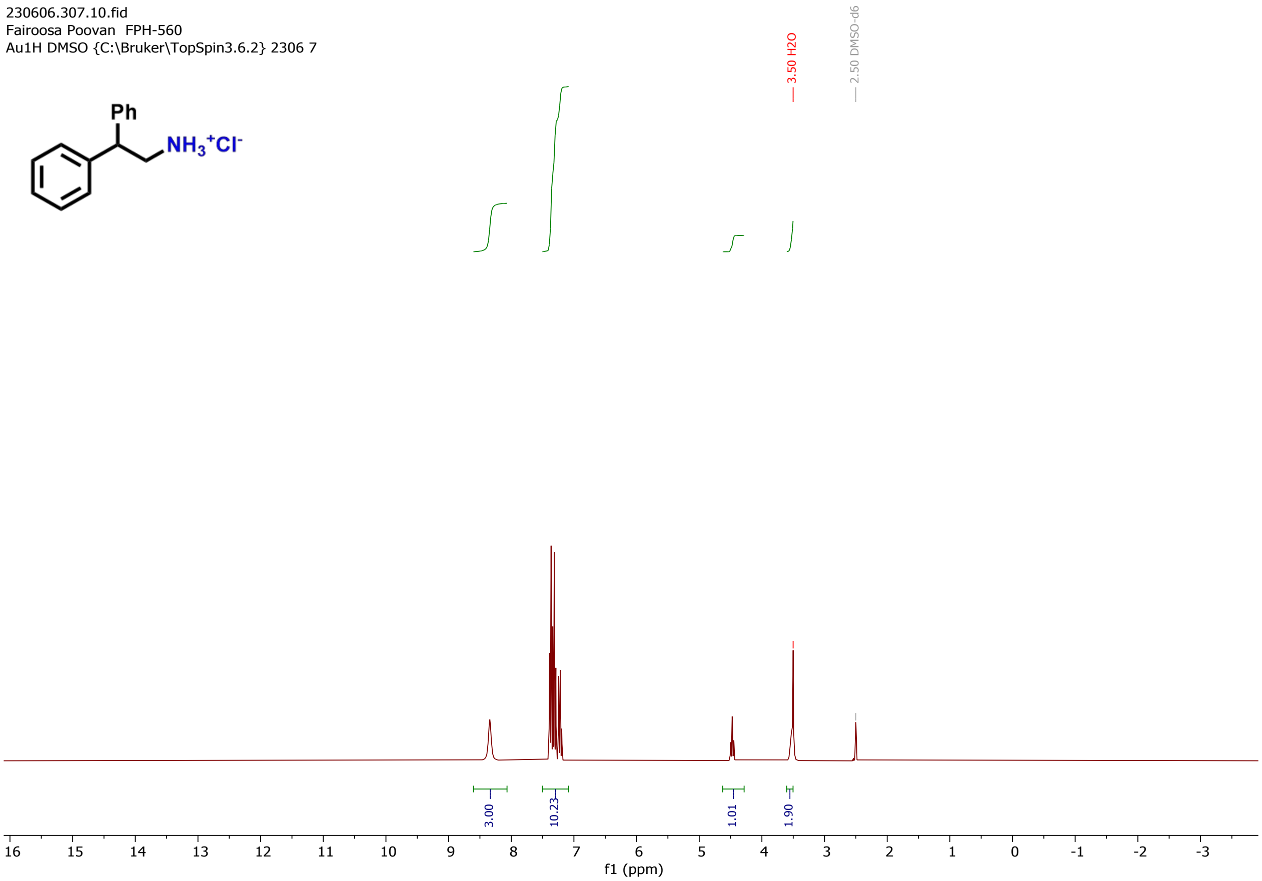
230606.307.10.fid
Fairoosa Poovan FPH-560
Au1H DMSO {C:\Bruker\TopSpin3.6.2} 2306 7
3.50 H2O
2.50 DMSO-d6
Ph
$NH_3^+Cl^-$
3.00
10.23
1.01
1.90
16 15 14 13 12 11 10 9 8 7 6 5 4 3 2 1 0 -1 -2 -3
f1 (ppm)

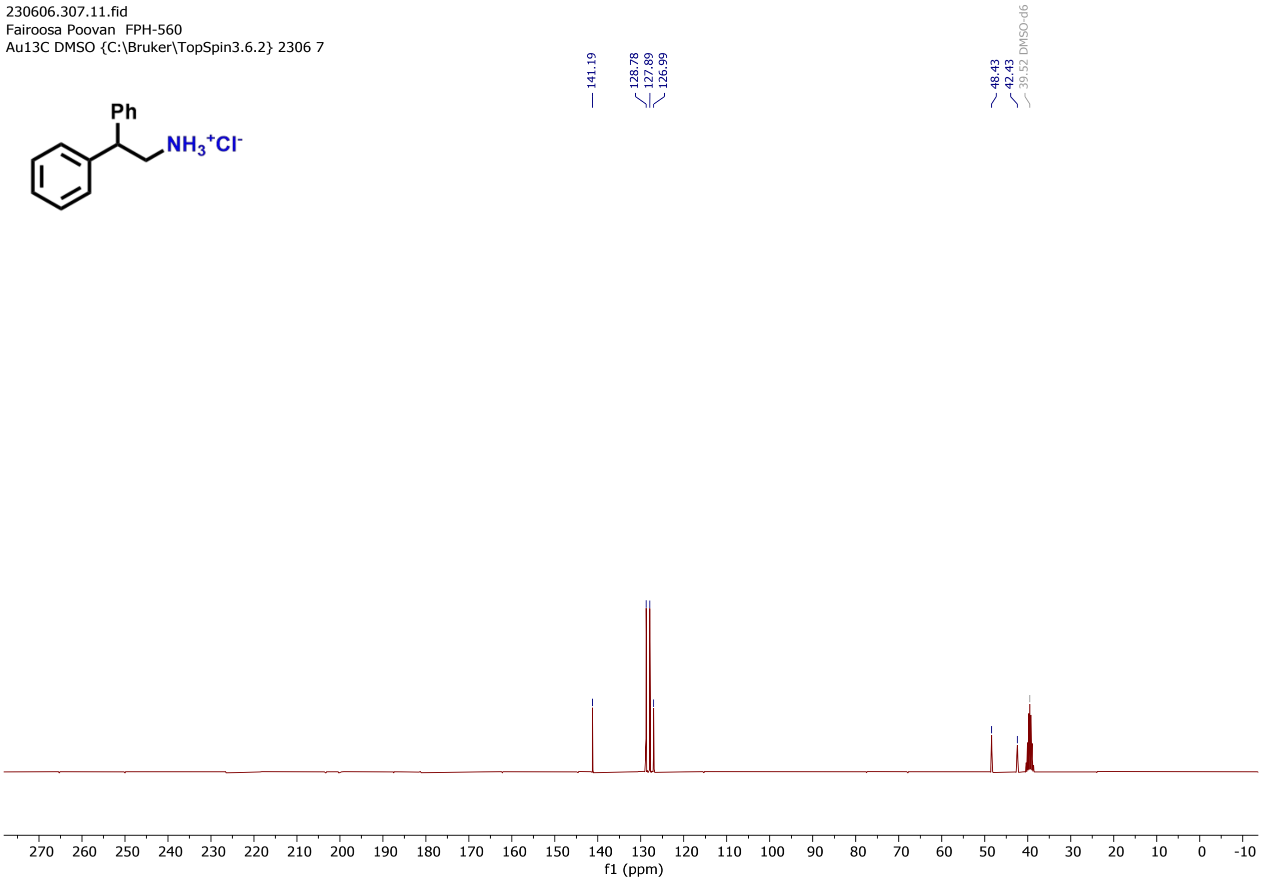
230606.307.11.fid
Fairoosa Poovan FPH-560
Au13C DMSO {C:\Bruker\TopSpin3.6.2} 2306 7
141.19
128.78
127.89
126.99
48.43
42.43
39.52 DMSO-d6
Ph
$NH_3^+Cl^-$
270 260 250 240 230 220 210 200 190 180 170 160 150 140 130 120 110 100 90 80 70 60 50 40 30 20 10 0 -10
f1 (ppm)

230712.318.11.fid
Fairoosa Poovan, FPH-503-PA
Au1H DMSO {C:\Bruker\TopSpin3.6.2} 2307 18

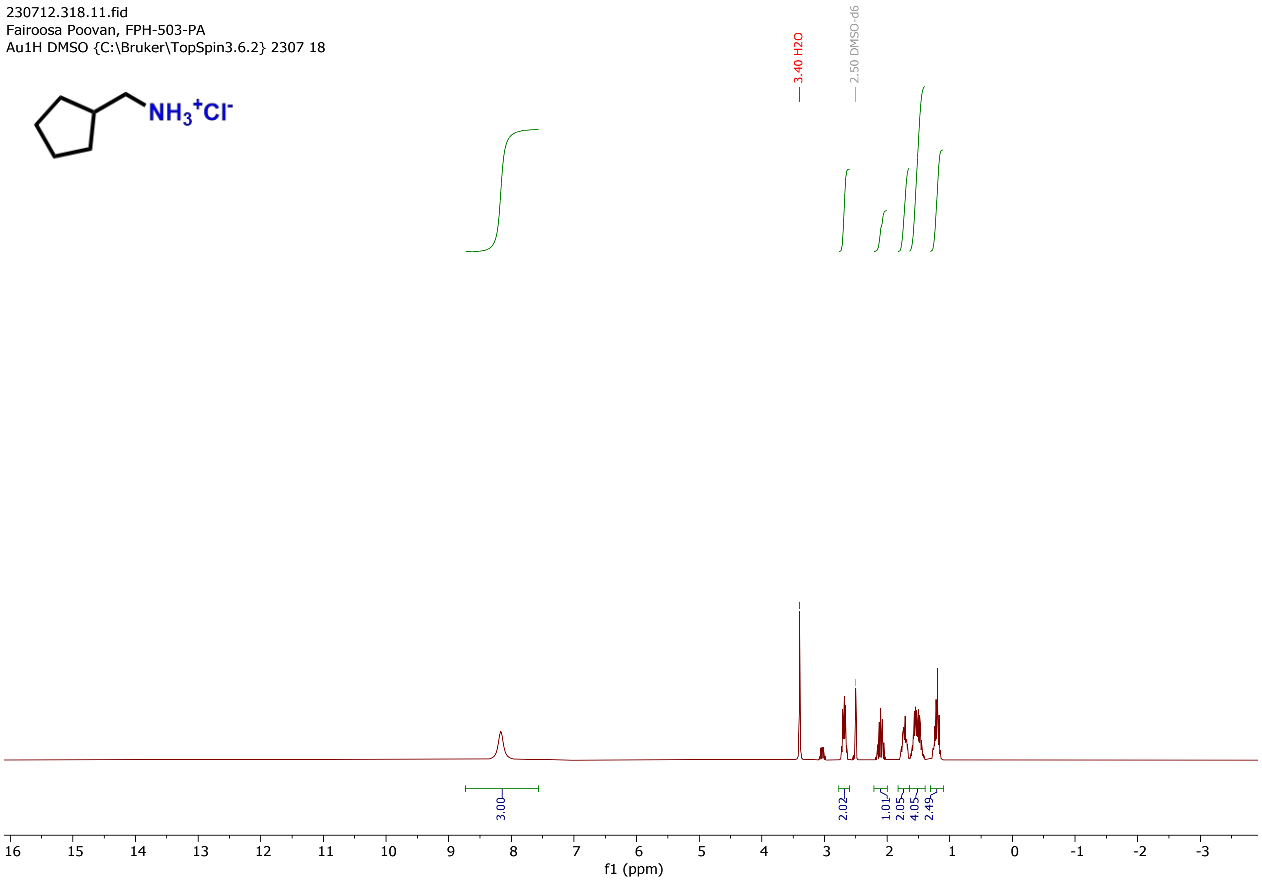

230712.318.10.fid
Fairoosa Poovan, FPH-503-PA
Au13C DMSO {C:\Bruker\TopSpin3.6.2} 2307 18

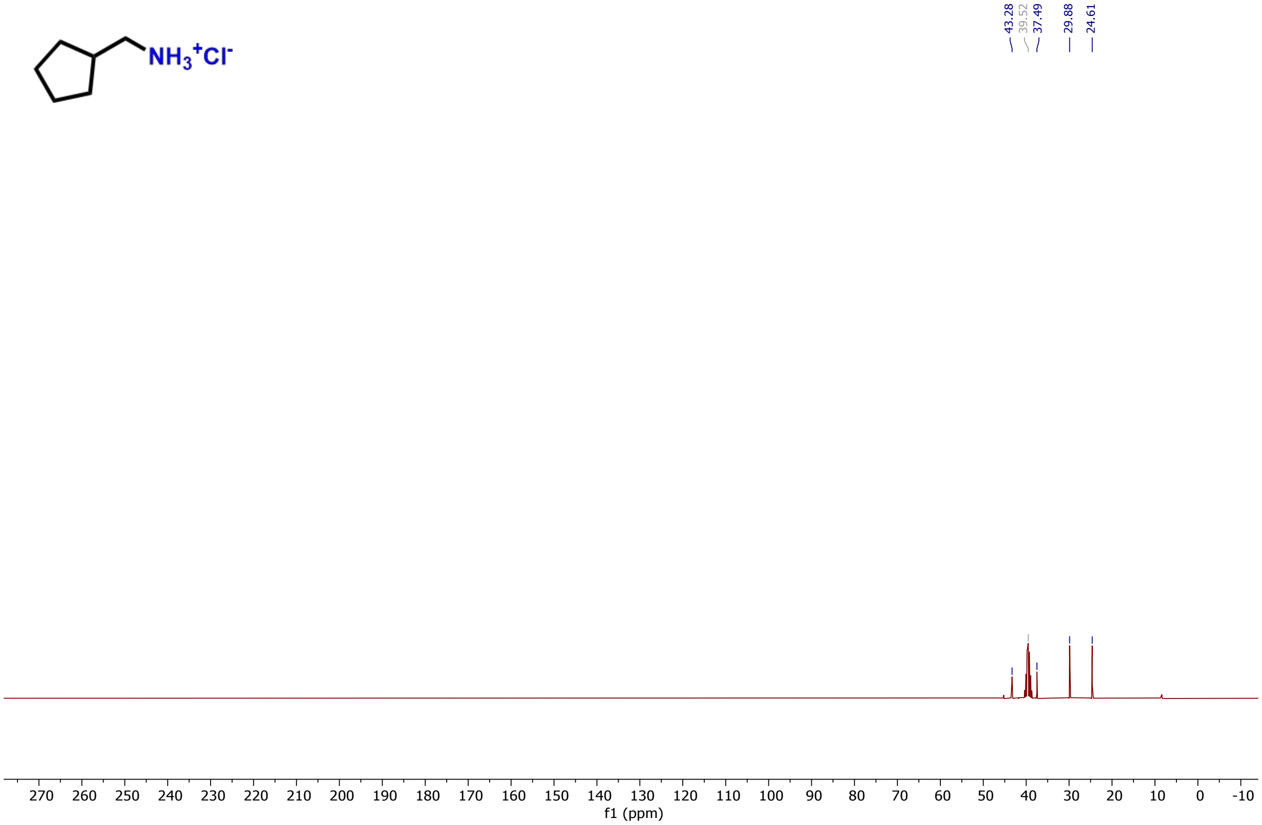

230712.317.11.fid
Fairoosa Poovan, FPH-420-R-PA
Au1H DMSO {C:\Bruker\TopSpin3.6.2} 2307 17

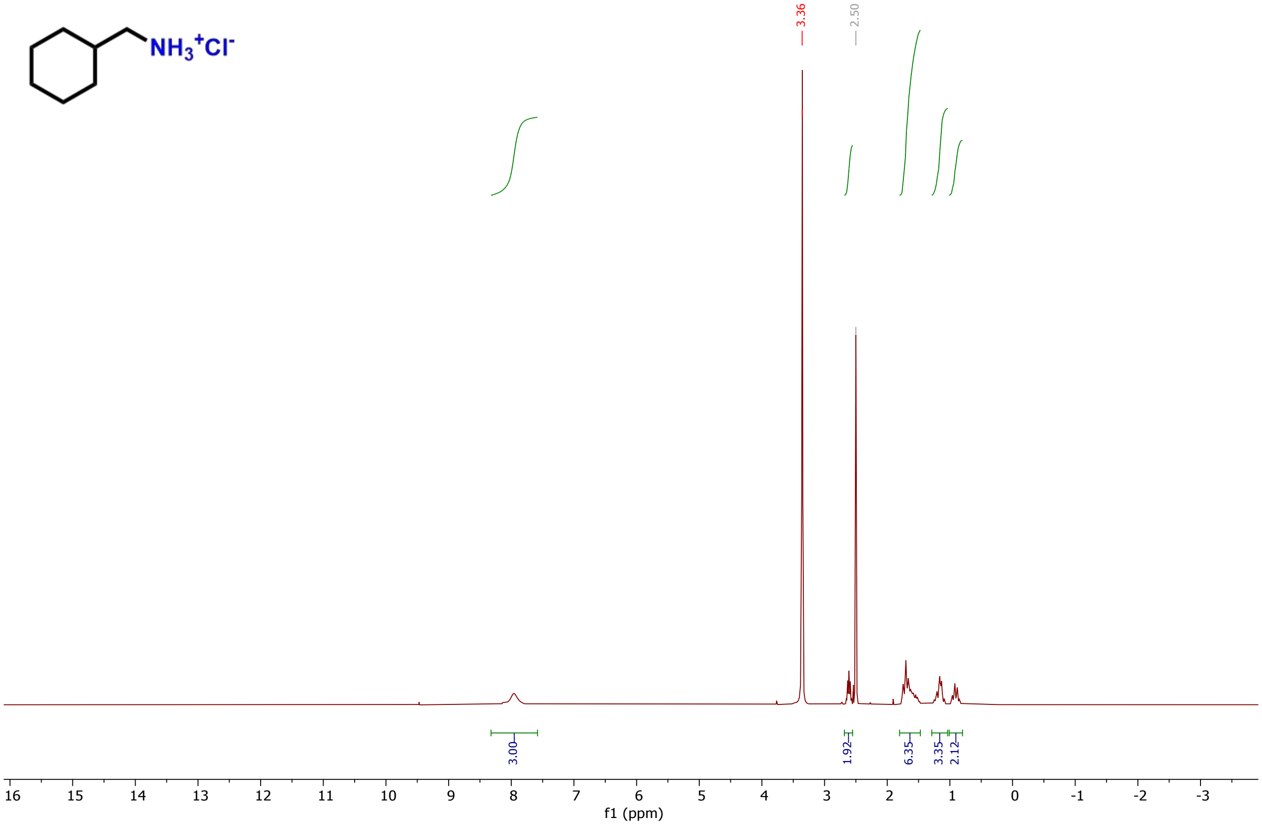

230712.317.10.fid
Fairoosa Poovan, FPH-420-R-PA
Au13C DMSO {C:\Bruker\TopSpin3.6.2} 2307 17

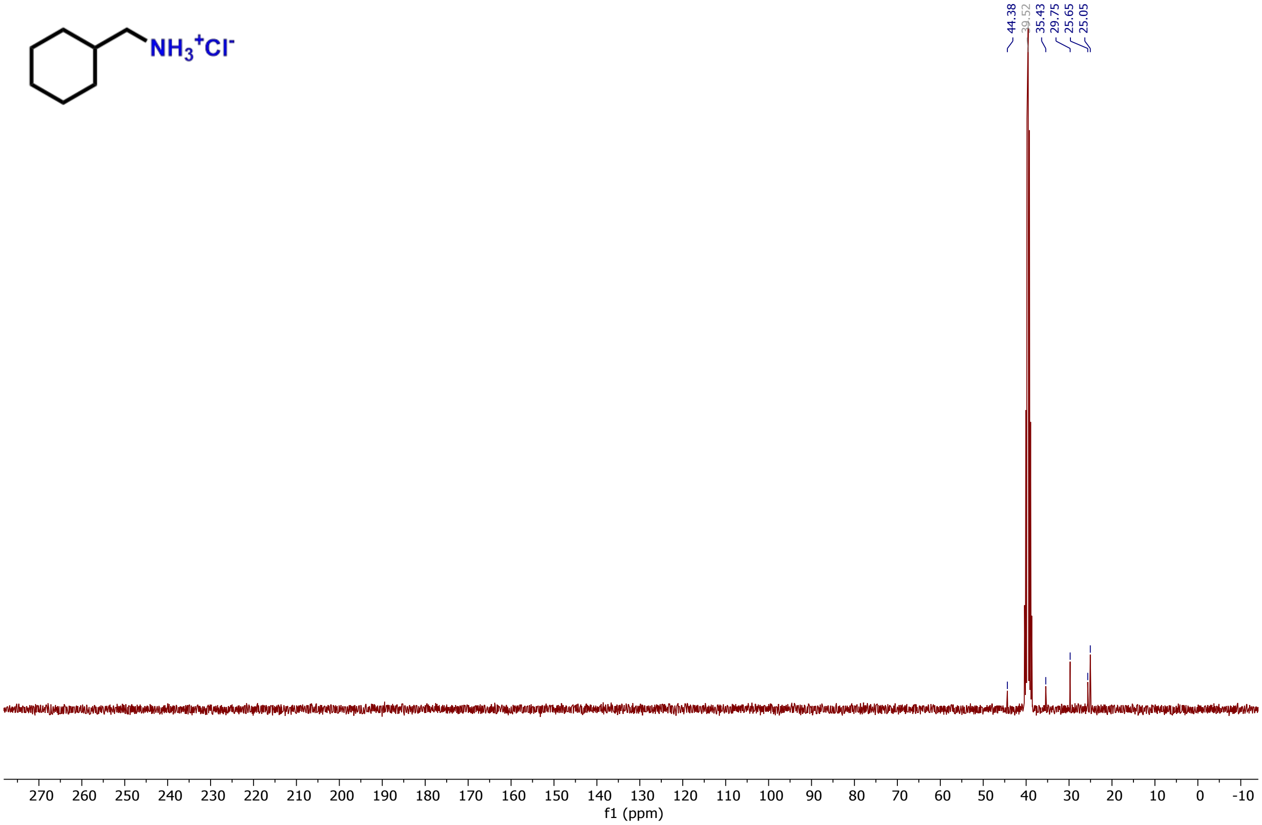

230703.315.12.fid
Fairoosa Poovan FPH-344-PA Wdhl.
Au1H DMSO {C:\Bruker\TopSpin3.6.2} 2307 16

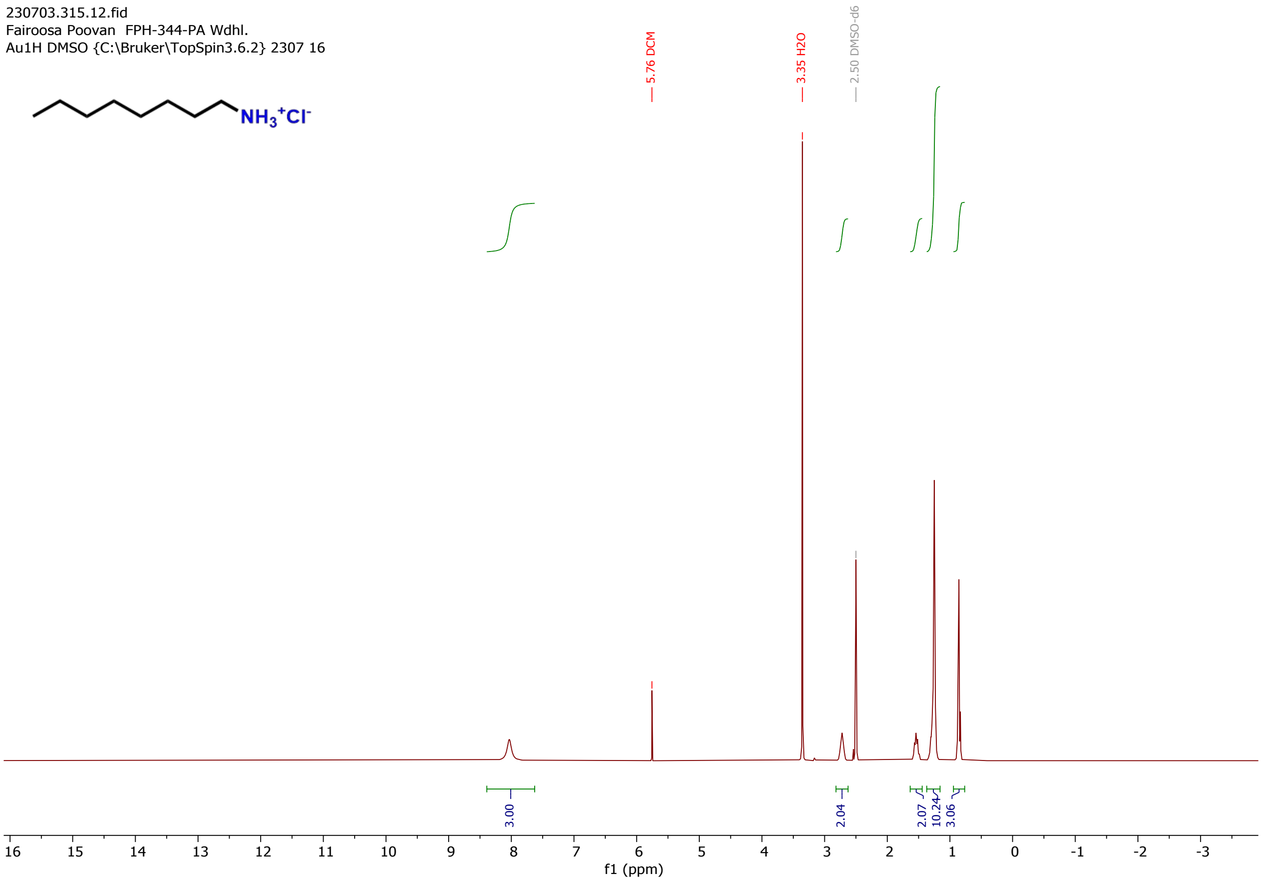

230703.315.11.fid
Fairoosa Poovan FPH-344-PA
Au13C CDCl3 {C:\Bruker\TopSpin3.6.2} 2307 15

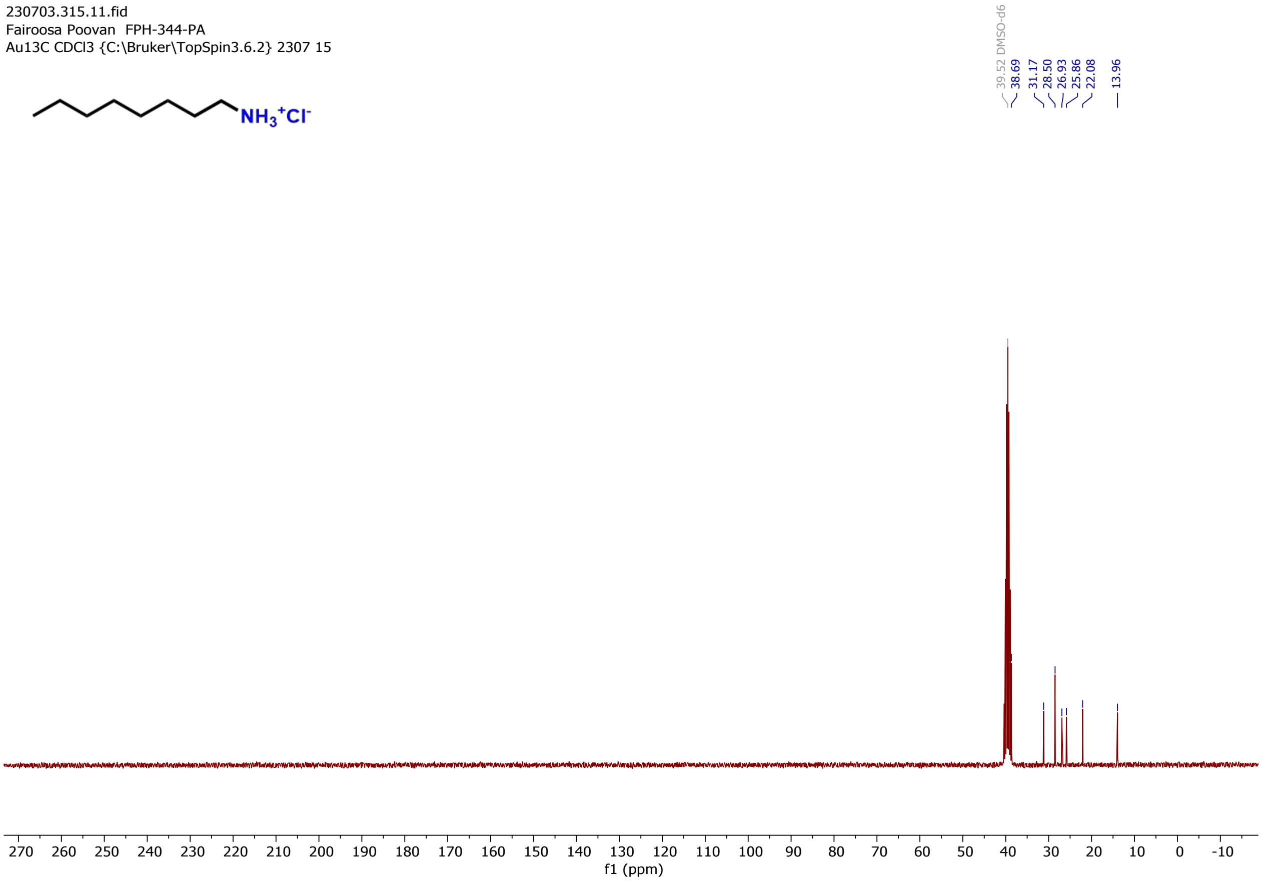

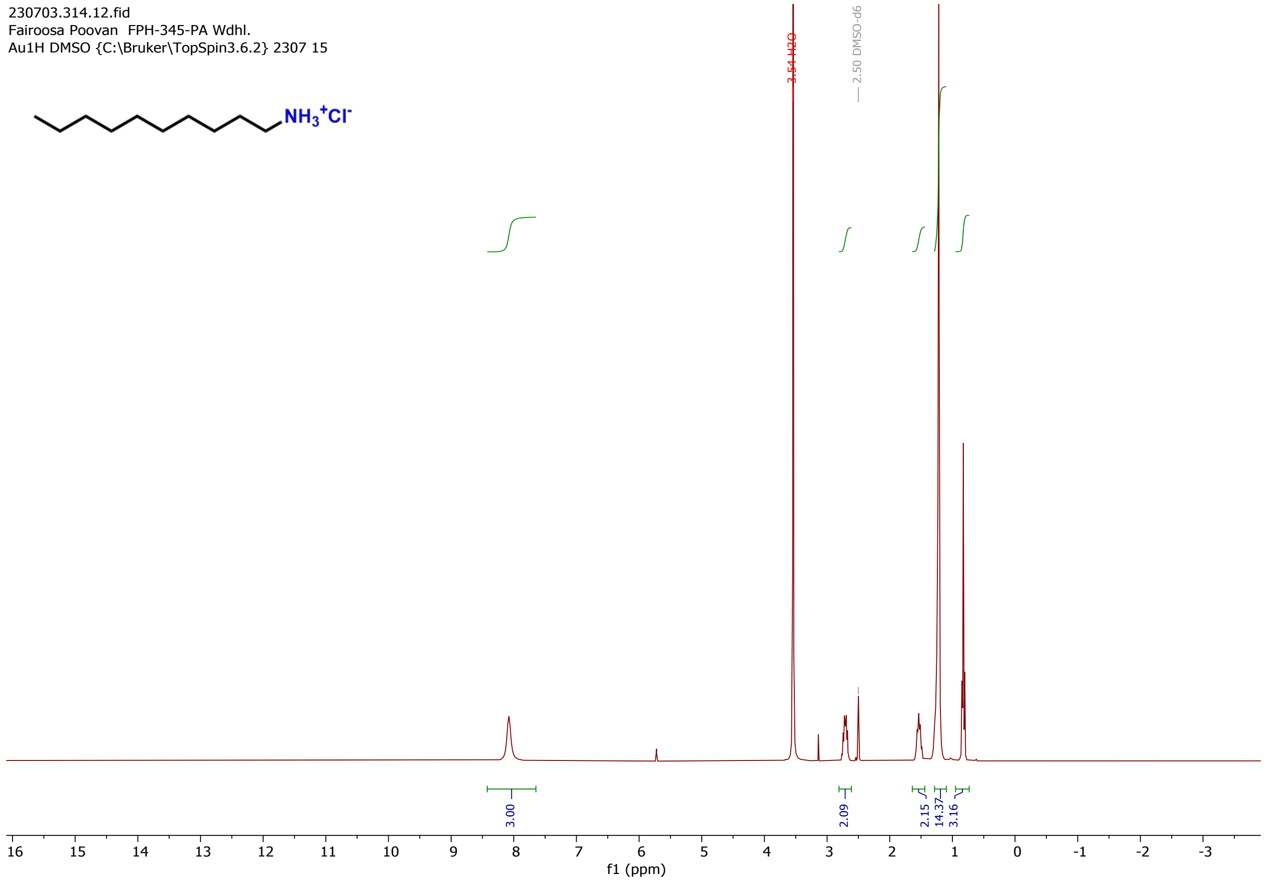
230703.314.12.fid
Fairoosa Poovan FPH-345-PA Wdhl.
Au1H DMSO {C:\Bruker\TopSpin3.6.2} 2307 15
NH3+Cl-
3.54 H2O
2.50 DMSO-d6
3.00
2.09
2.15
14.37
3.16
f1 (ppm)

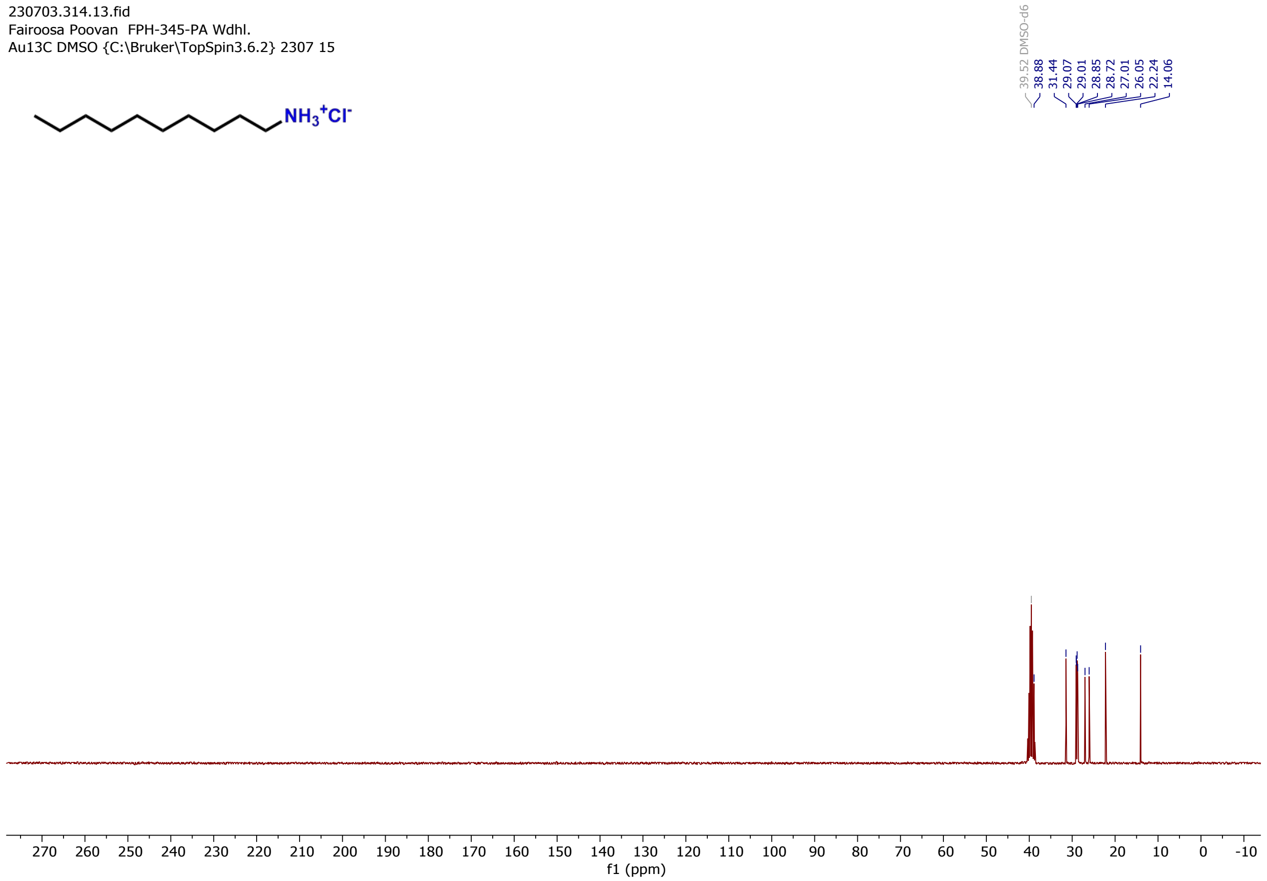
230703.314.13.fid
Fairoosa Poovan FPH-345-PA Wdhl.
Au13C DMSO {C:\Bruker\TopSpin3.6.2} 2307 15
NH3+Cl-
39.52 DMSO-d6
38.88
31.44
29.07
29.01
28.85
28.72
27.01
26.05
22.24
14.06
f1 (ppm)

230703.f328.10.fid
Fairoosa Poovan
FPH-775-PA
PROTON DMSO {C:\Bruker\TopSpin3.6.2} 2307 28

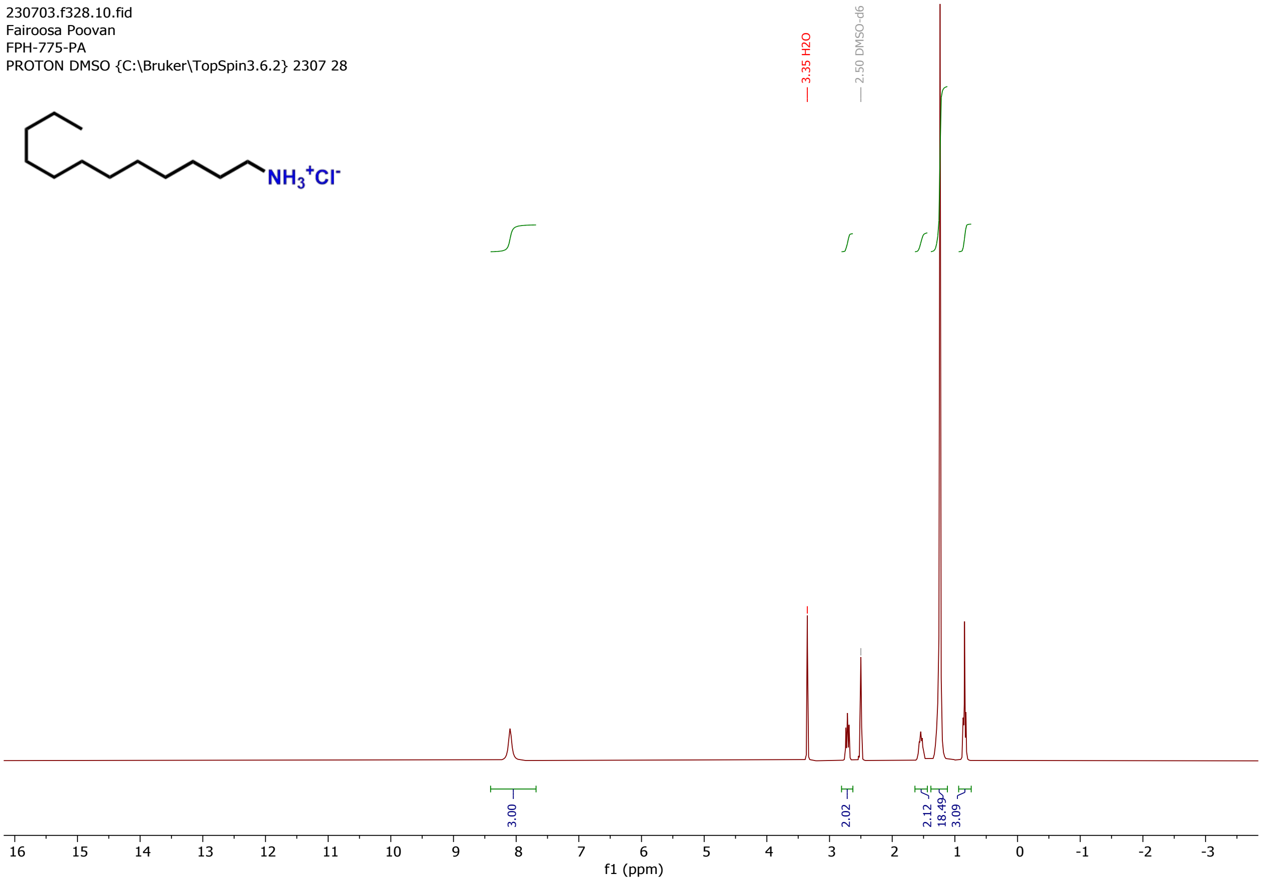

230703.f328.11.fid
Fairoosa Poovan
FPH-775-PA
C13CPD DMSO {C:\Bruker\TopSpin3.6.2} 2307 28

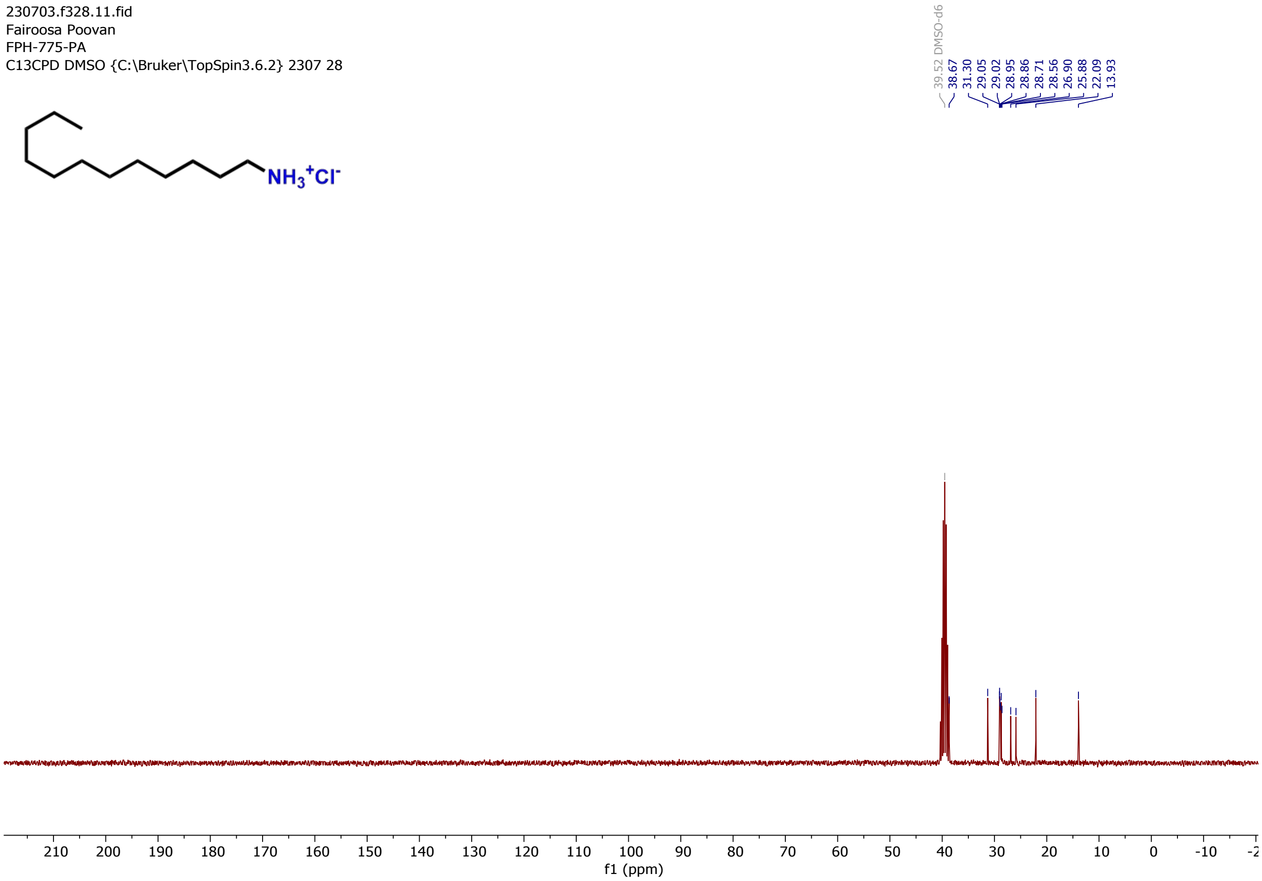

230703.f331.10.fid
Fairoosa Poovan
FPH-776-PA
PROTON CDCl3 {C:\Bruker\TopSpin3.6.2} 2307 31

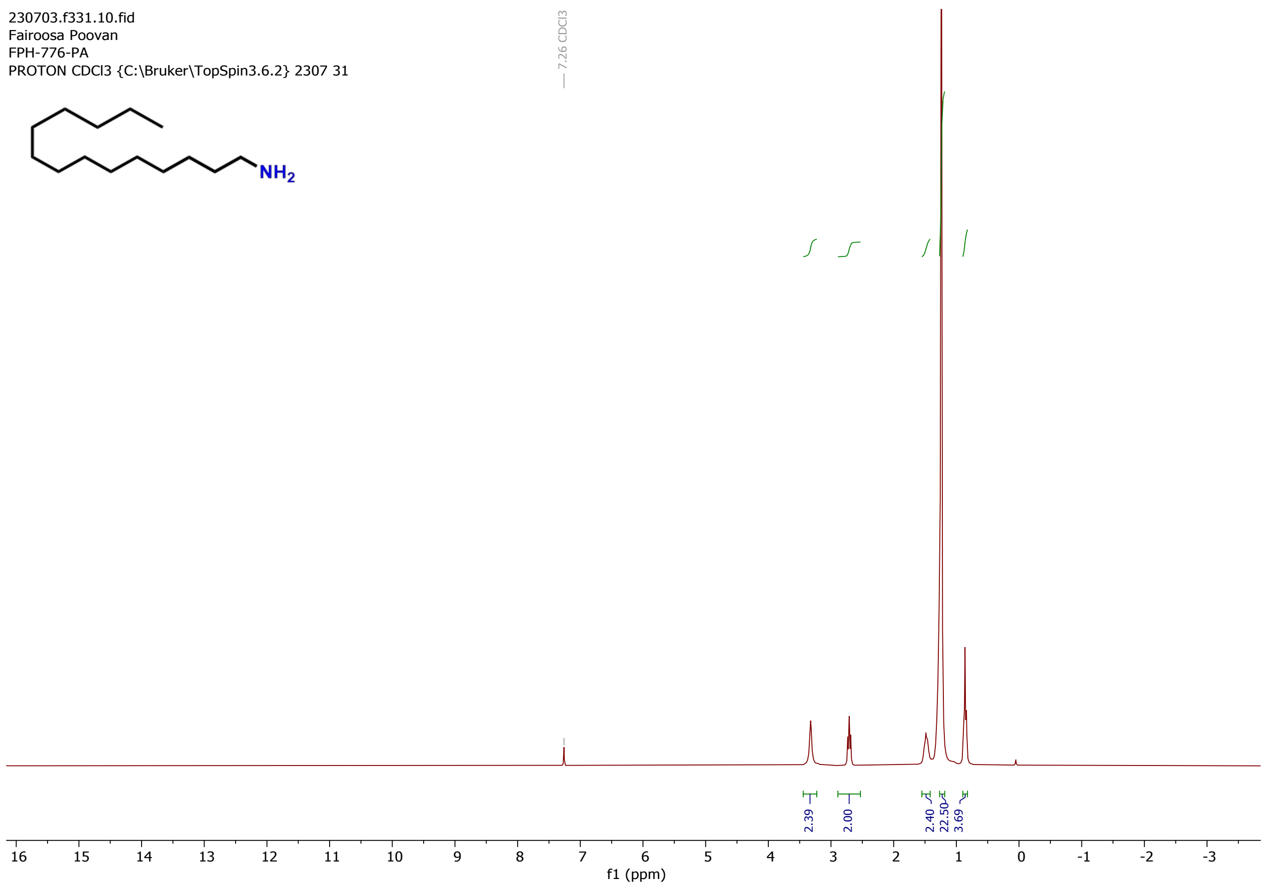

230703.f331.11.fid
Fairoosa Poovan
FPH-776-PA
C13CPD CDCl3 {C:\Bruker\TopSpin3.6.2} 2307 31

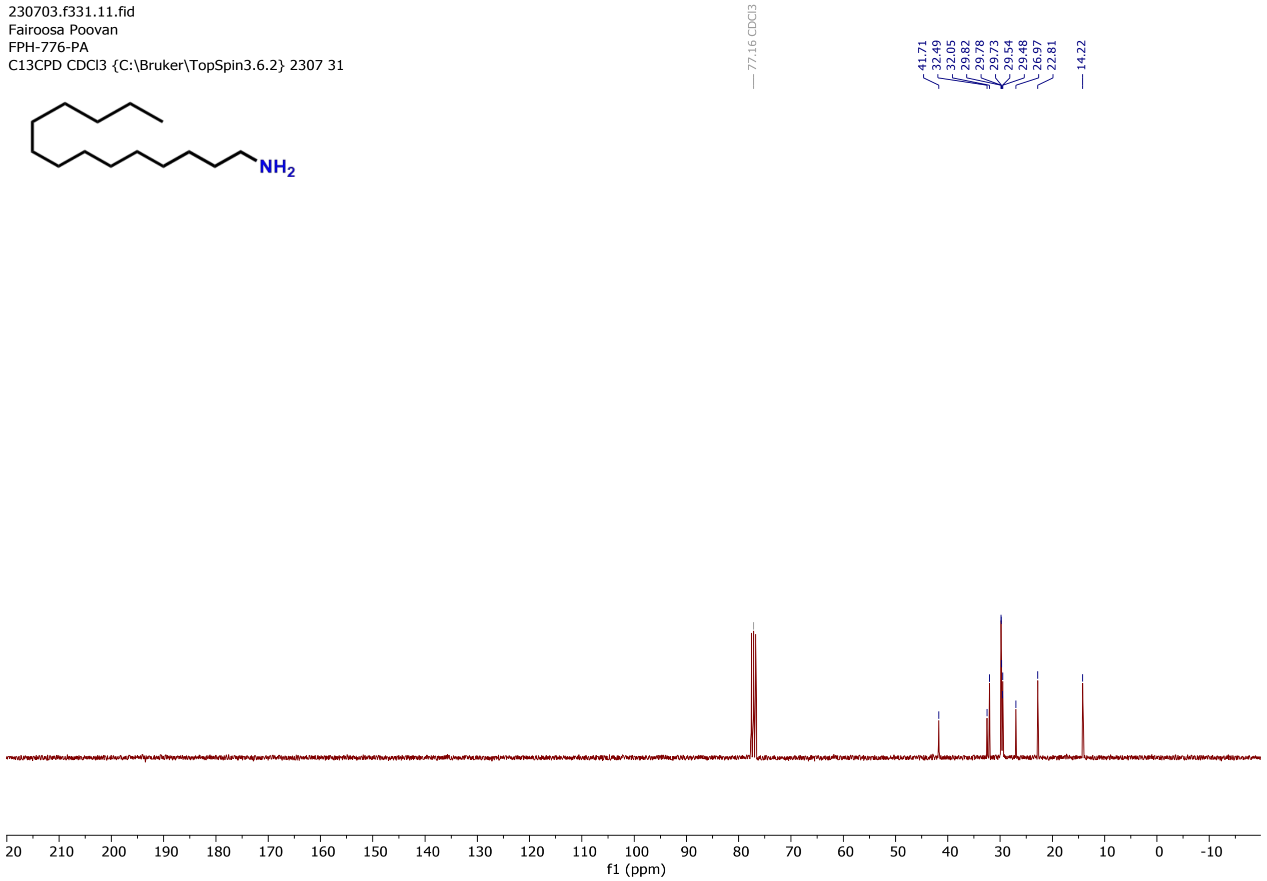

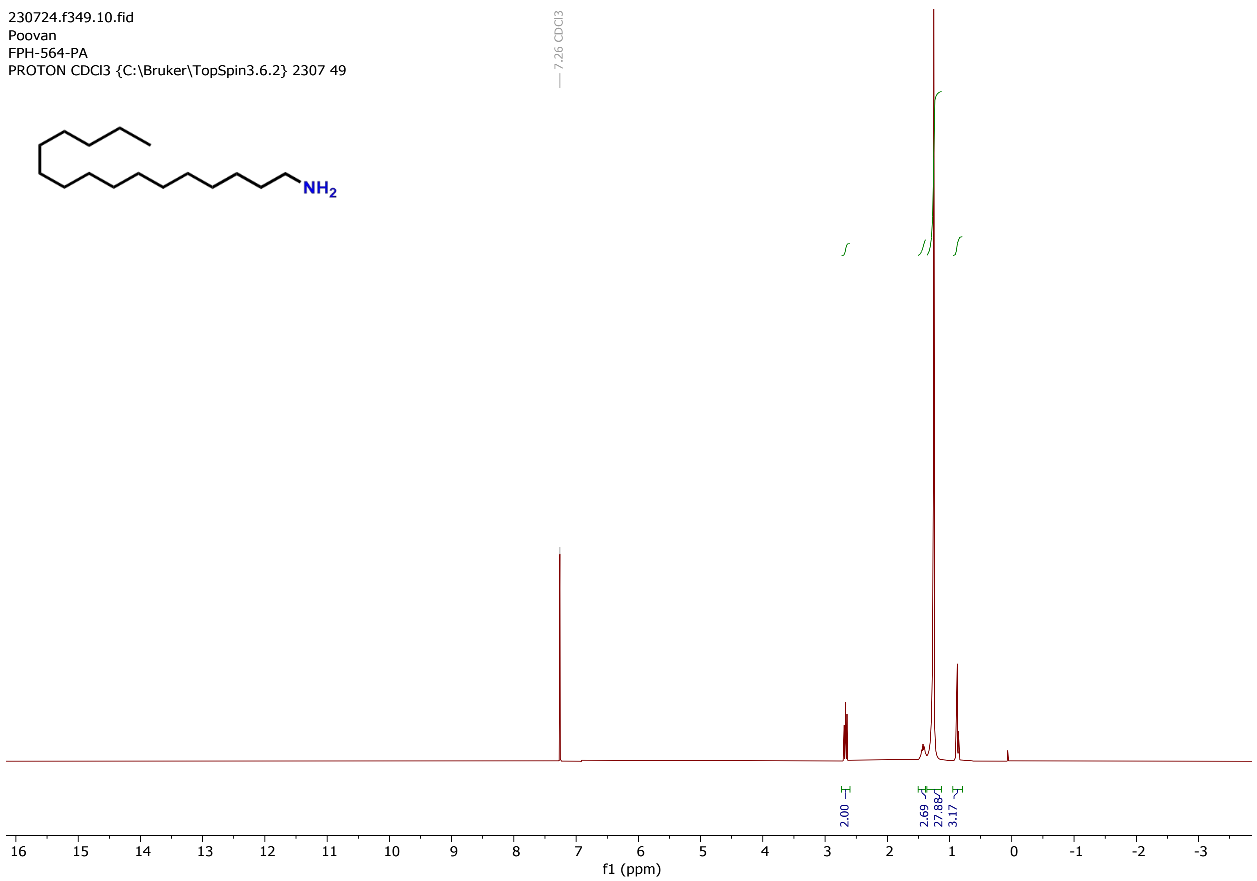
230724.f349.10.fid
Poovan
FPH-564-PA
PROTON CDCl3 {C:\Bruker\TopSpin3.6.2} 2307 49
NH2
7.26 CDCl3
2.00
2.69
27.88
3.17
16 15 14 13 12 11 10 9 8 7 6 5 4 3 2 1 0 -1 -2 -3
f1 (ppm)

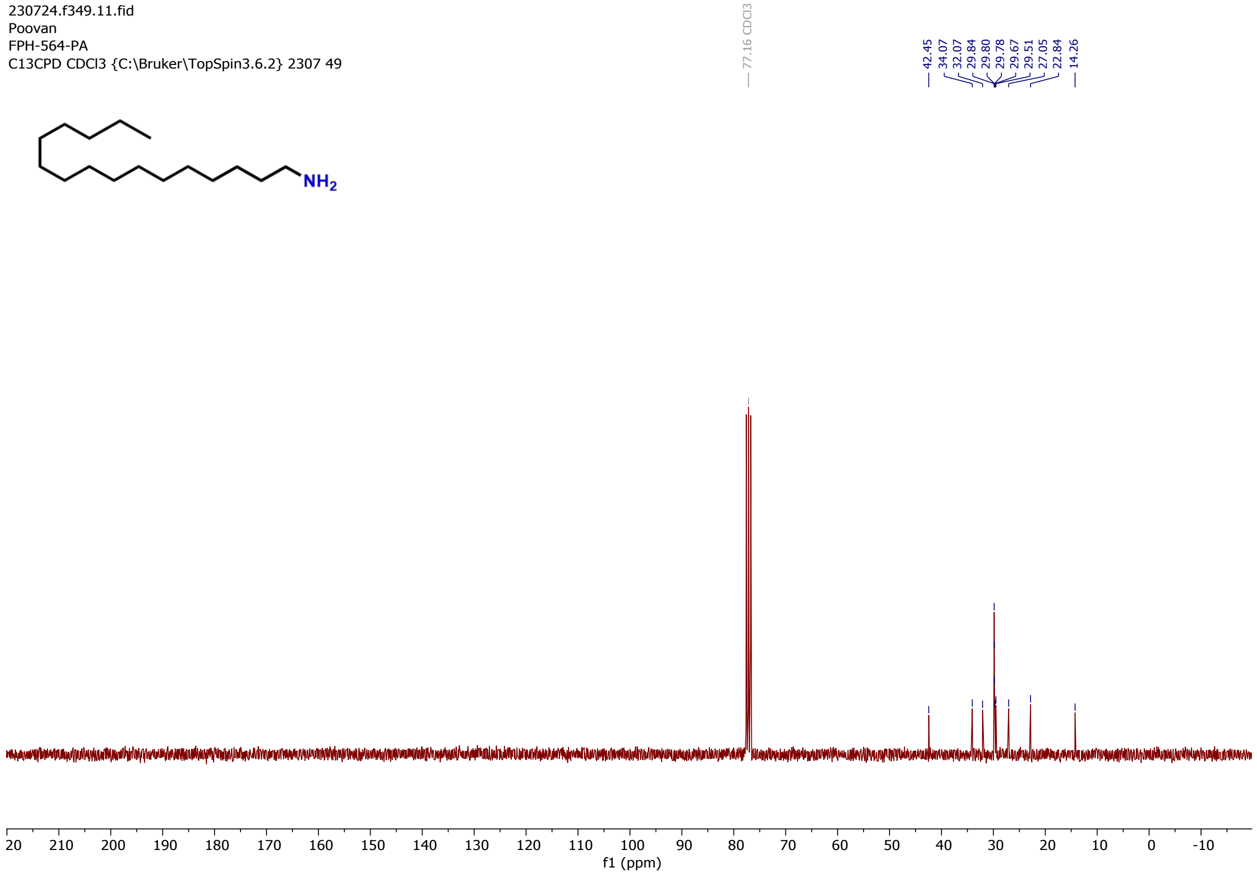
230724.f349.11.fid
Poovan
FPH-564-PA
C13CPD CDCl3 {C:\Bruker\TopSpin3.6.2} 2307 49
NH2
77.16 CDCl3
42.45
34.07
32.07
29.84
29.80
29.78
29.67
29.51
27.05
22.84
14.26
20 210 200 190 180 170 160 150 140 130 120 110 100 90 80 70 60 50 40 30 20 10 0 -10
f1 (ppm)

230726.f333.10.fid
Fairoosa Poovan FPH-515-PA
PROTON CDCl3 {C:\Bruker\TopSpin3.6.2} 2307 33

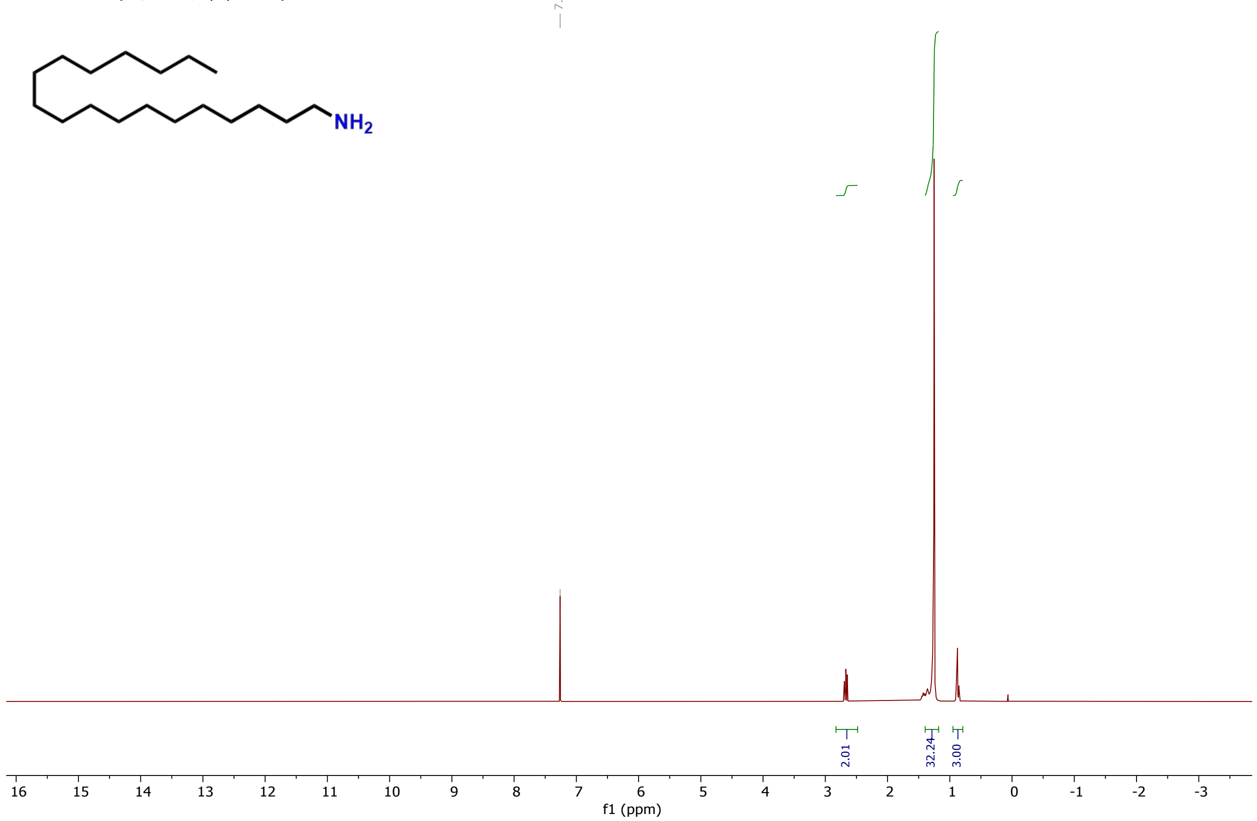

230726.f333.11.fid
Fairoosa Poovan FPH-515-PA
C13CPD CDCl3 {C:\Bruker\TopSpin3.6.2} 2307 33

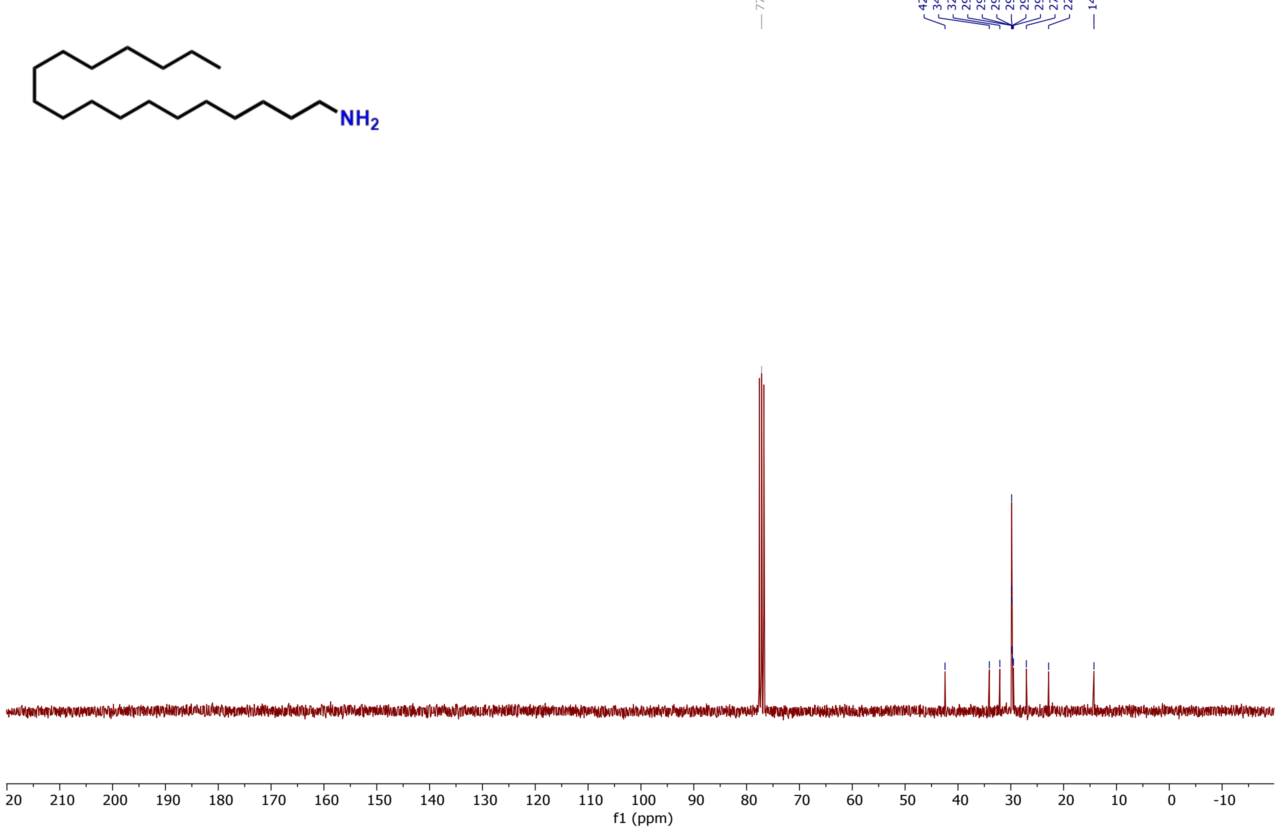

18. References

1. Busacca, C. A.; Grossbach, D.; Ponti, A.; Saha, A.; Grinberg, N.; Haddad, N.; Lee, H.; Lorenz, J. C.; Reeves, D. C.; Sarvestani, M.; Senanayake, C. H. A Superior Method for the Reduction of Secondary Phosphine Oxides. *Org. Lett.* **2005**, *7*, 4277−4280.
2. Wesselbaum, S.; Vom Stein, T.; Klankermayer, J.; Leitner, W. Hydrogenation of Carbon Dioxide to Methanol Using a Homogeneous Ruthenium–Triphos Catalyst: From Mechanistic Investigations to Multiphase Catalysis. *Chem. Sci.* **2015**, *6*, 693−704.
3. Westhues, N.; Klankermayer, J. Transfer Hydrogenation of Carbon Dioxide to Methanol Using Molecular Ruthenium–Phosphine Catalyst. *ChemCatChem* **2019**, *11*, 3371−3375.
4. Molitor, S.; Becker, J.; Gessner, V. H. Selective Dehydrocoupling of Phosphines by Lithium Chloride Carbenoids. *J. Am. Chem. Soc.* **2014**, *136*, 15517−15520.
5. Ramos-Villaseñor, J. M.; Rodríguez-Cárdenas, E.; Barrera Díaz, C. E.; Bernardo, M.; Frontana-Uribe, B. A. Review—Use of 1,1,1,3,3,3–Hexafluoro–2–Propanol (HFIP) Co-Solvent Mixtures in Organic Electrosynthesis. *J. Electrochem. Soc*. **2020**, *167*, 155509.
6. Mealli, C.; Midollini, S.; Sacconi, L. Synthesis and Structure of Some Cobalt (II) Complexes with the Tridentate Ligand 1,1,1-Tris(diphenylphosphinomethyl)ethane. Inorg. Chem. **1975**, *14*, 2513–2521.
7. Kuehn, M. A.; Fernandez, W.; Zall, C. M. Structure and Thermodynamic Hydricity in Co-balt(Triphosphine)(Monophosphine) Hydrides. *Inorg. Chem*. **2023**, *62*, 8505–8518.
8. Federse, C.; Ziebart, C.; Jackstell, R.; Baumann, W.; Beller, M. Catalytic Hydrogenation of Carbon Dioxide and Bicarbonates with a Well-Defined Cobalt Dihydrogen Complex. Chem. Eur. J. **2012**, 72-75.
